# Supplementary material for: Identification of a blaVIM-1-Carrying IncA/C2 Multiresistance Plasmid in an Escherichia coli Isolate Recovered from the German Food Chain
Source: Microorganisms. 2020 Dec 24;9(1):29. doi: 10.3390/microorganisms9010029 (PMC7824508; doi:10.3390/microorganisms9010029)
Supplement: Supplementary file 1 [file microorganisms-09-00029-s001.pdf]

>2 length=190205 depth=2.01x circular=true
ATGGACCACCAGCTAGAAAGTATCGACGGAACAATCATGAGCAAGAGAACCAAAGACAAA
GACCTGGAGAAACTCGACGTAATCAAAGACTCACCGCAAATGAGCCTGTTTGAGATCATT
GAATCTCCGGCCAAGAAAGACGACTACTCCAACACCATCGAGATCTACGATGCGCTGCCG
AAGTACATTTGGGACCAAAAGCGTGAGCATGAAGATTTATCCAACGCTGTAGTGACACGA
CAATGCACCATCAGAGGCCAGCATTTCACGGTGAAGGTGAAGCCAGCCATCATCGAGAAG
GATGACGGAAGAACCGTGCTGATCTACGCGGGACAGCGAGAGGAAATCCTTGAGGATGCT
CTACGCAAGCTCGCAGTGAACGGGAAAGGCCATATCATCGAGGGCAAGGCTGGAGTCATG
TTCACTCTGTACGAACTCCAGAAAGAGCTCTCGAAGATGGGTCACGGGTACAACCTGAAC
GAAATCAAGGAAGCAATCCAGGTTTGTCGTGGCGCAACACTCGAATGTATCAGTGATGAC
GGTGAAGCCTTCATCAGCTCCAGCTTCTTCCCGATGGTGGGACTGACCACCAGAGGTGAG
TTTCGCAAGAAAGGCGGGAACGCCAGGTGCTATGTGCAGTTCAACCCGCTGGTAAACGAA
TCGATCATGAATCTGTCGTTTCGTCAGTACAACTACAAAATCGGAATGCAAATCCGCTCC
CCTCTTGCACGGTACATCTACAAGCGAATGAGCCACTACTGGACTCAAGCATCACCAGAT
TCGCCGTACACGCCATCGCTTATCAGCTTCCTGACCCAGAGCCCTCGTGAATTGAGCCCA
CGGATGCCGGAGAACGTCAGAGCCATGAAGCTCGCTCTGGAGGCCCTCATCAAACAAGAG
GTCATAAGCGACTACGACGCGAACCAGATCAAGGATGGCCGCAGAGTCATCGACGTGCGG
TACGTCATAAGGCCTCATGAGAACTTCGTGAAGCAGGTGATGGCGTCCAACAAGCGTAAG
CAGCAGACAGAGCTACGGGCCATCAAGCACGGCATGATCGACCACGACATCATTGATGAA
CCCCAACGTAAGGGGAGATAGCCACCAGAATCTCCCACCCCCCAGGGAAAAGAAAGATAG
GCGCGTCAGCGCCTTTTTTTATGCCAGAAGAAAGCGCACAAGGTACGCCCACCAGGCAGG
ACCGGAAACGGAATGCCTCCGCATATGGGGGAGCTGCACGGATAGAGCGAACGTGACCCC
AGATGCAGCGTGGGCTGGGGCTCTATATATGGGATCGCTGCACGGTAGTCGCAAGCTGAG
TCTCAGTATCGTGCAAGCGTAAGCCACGACGAAGGATAAGTGCCATGCTGCTCCAGTGGT
TCCCGTAGAGTGGTTCCCGTAAGGTATTGATAGTTAAAGGGTGAGGATGTGGGGGACCTG
CACGTTATGAGATTTGATATGTGGGTACGTTGCACGAAAGCAGGTCCTGAACCTCTCGAT
ATATGGGAACTCTGCACGATTGACGAAGCAGAACAAAGATTGACAGATCAGGACAAACCT
AAAAAAGCAGTCAAAATGACTCTAATAGTCCATAAAGTGATCACGCGCACACAAAAAGAG
GCCAAAAGTTATCCACATTTCCTGTGCATAAACGGGGTTTTGGTATATGGGAACGCTGCA
CGATCATATGTGGGTACGCTGCACGGTGCAAAAAACGACCAAAATTGACATGTGGGTACG
TTGCACGTTCAGAAAATTGTATATGGGGCGACTGCACGTATGACCTTAGACATGTGGGAA
TACTGCACGAACACCAGATCTGATATGTGGGAACGCTGCACGAAAGCTCTGTTTTGTAAG
GATTTTGTGGAAAAAAGAGGGGTTCCGTGTGGATAACAGTGGATAAGATCCAAGGGTATG
TGGGGACGTTGCACGGCAACCTATGGGAACGCTGCACGGCAACCTATGGGTTTGTTGCAC
GGAATAAATATGAAAACACCAGTAATATCAATGTGTTGAGTGGTGATTTAGAACGCCTAA
CCTGTCTTTAACCGTTTTTAATCCTTTATACAAAGATCTAAAGATCTTTATATTTATCCT
TTTGATCACTGAACAAAAAATAATCAACACTGTTTTTTTAGAAAAAAGTCTCGTATGCAA
GGCCGAACAGGACGAGTATCACCAACAAGATAAGCATCGACAGCACAAAGTAACCAGACT
CGTACATCACAGCTTTGATCACAGAAACGACGCTGATCGATCTTCCAGAGATCCTCCCGA
TGATCCACTGAAACGCAACGAACGAACCAAGCCCACAGATCAGAACCACCGCTACGGAGA
CAAGTGCATGTTCCCTCAGGACAGACTCACTCAGGAAACCATACTGCATCCCCATCAATA
CAAGCCTGCCAATGCCAAAACCCAGGCCCAATGCAACAGCTCCACGAACCAGCACGACCT
TCAACGACTGGAGCCATGTCAGCTCAAGATCCATCCCCTTACGGGAAGACCGACGCACCA
GCACCGTAGATATGCCCAACCCCAAAAACACCAAAAAAACAAAATCCAACATTGCCACCT
CTGCACAAATCAATCAGAACCACAGGCCTCTCGGCCAAATGCGCCACCAGGGAGAACCCA
CTTCTGCCCCTCCGGCACAAAAATCGGGAGCGTGTAATCTTCTCTGGATACCTGCTCCAA
TGCCTCAACATCACTAATCTGTATCGGCGCAACAGCAGAACCTAAATCTATTACGTCGAT
TTTTTTTCTTGATACCGTCACACCAAAGGTTTGTTCATACTGAGCAATTTTCCCAGCTCG
TTCAGGCCAGTAGTGACGGATCGTGGACCAGATACGCTGTGAGTTGTAGATGCAGGTCAT
ACACGACGACCGGCTCCAGCCAAGGCGATAAGGGACTGGGGCCAGGATGCGGTGACGCTC
GATTACTTCCCATACCTCCTCCTCAGTCCAATGAAGAACGGGCCTCCAGGCATCAACTAA
CCGTGCAGTCTTCCCATATCTTCTGTCACAGGCATGAGCCTCAAGCTGGTTGTACTTGGA
TCTGTTTGCACTTTCCTCCCGGCGCTCACCAGTGATGAAAAGGATCTTCTTCCCCTTGAA
GCGCTCCTGGTTATTAAGAGCTCTGCGGCCAACATCGATCTTCAAGGCTGATGAGCACCA

CCTTGTTTGGAGTGATGGCGATTGCTGAGGGAAACGAAGGCGCGTACCAGGCTTAGAGCG
CTTATGGTCTCTAGGCAGCACCAGAAGCCCCTCAGGCGTCTCTACACGATGGGGGTGGCT
ATAGGCATTGTCTTTGAGCATTTCGCCCTCAAAGCCGCCCTCAAGCCACGAGAAGTACAT
TGGGATACCCAGTTCTTCCCCGAGCTGACGACAGTAATCACGCATGAAGGCCCAATCCAT
CAACGAACTGCCTTCCTGACCATCAACATCATGGTGCCAGAACTCCACCTTTGACTTATC
AACACCCATGTCCACCAGCCGCAAGTACGCAGCAATCGAGTCTTTGCCTCCAGACAGGCA
AACAATGATGTGATCGTACAAACTCAGGTCCACATCCGGAGCGGAGAAGTACGTTGTTCT
ATCGTCACAACGCTGACTGGAAGATATGCCGGTAAGAACCGACTCCAGTGATGGCAACAC
CACCTGGTCATCGAACAAATCTCCTTGGTTTTCTCGTCTCAACATAAGTCGCTCTTTTTT
CTTTACTGGGTTGTTTTTATGATTTACAGTATTATAAAATGATACTGTGAAAAGTAAAGT
TTTCAGAACGACTAATCACTCAGGAGAACTTGATAAGCACCAGAAAGCAATCTAAAGGTG
GTTTTTCGGTGAGGTTAGTTCAGGGGCAACCTGCCGCTGCTGATTGGAAATCATCAAGAA
GTGGCTTTTTGGTGGGGTTACGGTTTGAAGGGGTGAGTATGTGGGAACGTTGCACGGTTA
AAGGGGGCCGCACATGAAAACGATAAAGCGGTTCATCGTATGGGTGAATTACGGTCTTGA
GGGTTGGAGCATTTTCGGATCGAGCGATGACTGGGATGAAGCGGTATCCATCCGTTCGGA
AGCGATAGACGAATGCAATATTGACGAGGAAGACATCATCTTGGCAGAAAACAAAAACGA
GCTGGTGGTAAAACCTGCGGCCAAGCAAATGACGGAGTGGCACCGGGAACTGGAAGCCGT
CCTTATGACCTTGGATGACTGCCAGATGGAATGTGACGGTATGACATGGGCAGTAAGCCA
CCTACTCAATGAAGCGGGTGTACCTCATGACTGCATGTATGGCTTTGTGCGAAACGAACA
GACCAAGGACATCGTGACACCGCATTTCTGGGTTGTCCTGGATGATGGGTGGCTGGTGGA
TCTGAGGCTACGCATGTGGCTGGGCGATCACGACAACATTCCTCACGGGGTATTCCATCC
CGATAACGAGCCAGGACTTTTCTACAAGGGAGACCCGGTTCAAAACCATAAAGGGATGAG
ACTGGGCAAAGCCGTCCTGGACATCATGACCGACGGAAAACTTTCACATGTGAAAGTTCC
TGAACGACAAGACGGAGAGTAATAGCGTGGAATTTGATTACGACAAGTCAGTATCAAACG
CACATCTTGAGGCCGCAGGCTGGGGTATGGATGCCTTTAACCACTCCAACCCATTTGAGA
GTCATGTCATCTATGTGCGTGACTACCGCAACGATCACATCCGCCTGTTCACCATCAAGC
AGGCTGACTTCGACACGATAAAGCTGCCGCTTCACCTTACCTCCGACATGCTGGCTTTGG
TGATCGCTGAGTTCGTATCGAAGGCAGCAAAAGGGAAGTTGAATACGAAGGAGTCCGACA
CGCTGGCTCCTGCCTTGGTTGGCTATGCCAAATCAACAGAAACCTACCGGAGCTGGCGGA
GAGTTTCTGGAGCAACAGAGCGATTGCATATGGTCATCAATATCTATGCGGGTTCTGAAT
TGCTGAGACCTTTCATCGCTCGGGCTCCTGAGACCGTCCTGACGACCCAAGAGCTACTGG
TCTTCTCATCACAGGTTAAGAGCATGGATGTATCCAATCATCCAGAGTGGTTTAGGGGCC
GGAGGTAGTCATGAAGCCAGTTCTCTGGATATTCGTCCTGATCATCGCCCCTTTCGTCAT
TGCGAAAGTGGACCAATGGCGCAAACGCGGCATTGGAGACACCTGGGCGTGGTGGAAATC
AGAGAACATGCCTTATGAGCTGCGCTCGGCCACTCTGTTTTTGTCCGAACAGGACATATC
AACCACACAGCCGGTGCCCATGCACGGCAGGGTTGATCAGGTGTACCAAACCAAGAATGG
GGTTCTCATTCCCTTGGACACCAAGCTGCGCCAGGTAAACCACATCTATGAGTCGGACAT
TATCCAGCTCTCGGTTTACCGGGTAATTCTGTCACACAAGTACAAGGCACCCGTTGCCAA
GTACGGCTATGTCCGAACCGTTGTTGAGACAGCGGACGGGGATAGAGTCCGTTACATCAA
GACAAACCTTCTCAGCGAAAAAGAAGTGGTCAAGCTGTGGCACCGCTATCAATCCATCCG
TTCTGGTCAGGTAAAAACCTCCTGTTCCTGTGGCGGAAAGTTTCACATGTGAAAGTTCTG
GATTGAATCAGGGTTGCCCGAATTGGGCAGCTAGGCGGGGTTGCATTTTTGAAAAGTCCT
GTACGCTGGTTGGTATAGGAGGTGAATAGTGGCCCAGCTTAAACATAACCGCTTCGTCAT
GTTGGGGTCCTTGGTAGCGACGGTCGTAGCTGCCGCCAATGGCTTGAGTTTTCTCACCCT
TATGGCTGGTGGAATGACCCTTCTCCAGTTCATGGCTTGGAAGCAACAAGATGCTGTCCG
GAGCCGGTTTAAAGCTGCGAAGGATGCGTTTGAAGCGTTGAACGTCATTGCGTTCGACAA
GCACTGGGTAGGTTCAACCGCGACGGTCGCCAAAGTGTCGAACATGATCACTCCGCCCGA
ACGACTGGATAAGCCCTGGGCGGTGCAAGTTCTGGCCGTGACCAAAGGTGGGACGTGGTT
CGCGGTGGATCTGCAAGTGACCGGCACTGACAAGGTTCAGATGCTTAGCCTCCACCAGCT
TAGTGAAAAGGCGGCAAAGACCATGCTGGCCTTCGACCTCGAAGTGTATGAGAAATTTTT
CGGAAAACCTGATGTTGCATGAGGACAATGTGATGGAAAAAGTCAAAAGCGTAGATGCCG
TGGAGTTCACGGCAGCAGATAAGAAGGCGATGACCCATGCGGCATACGCCCAGTGTTTCT
TCATTCTGGCCCAAGTGATGGCATTCCCTTCCCTGGGGGTGGCTGGCGCTATGGTTGCTG
CTCTGTGCGGCATGATGCCGTGGTTGACCAAGTTCGCCAAAGAGGCTCCATCGAAAGCCT

TTGGCATGGTGATGGCTTCACTCTGCCTTGCTCCTGTGTACGGGAAACTCTGTGAGCTGG
TGGTCCACGCGATTCAGGGAGCTTGAGCTTATGGGGGTAGTACGAGTTCCCTACCTCTTG
GCTGAGCTGAAAGAACGCGGATGTGCTGATGAATCTGCTCTGGCGCAGGTTATGCAGCCT
GGATGCCGAATTGGAGAGGAAGACCTGCGGAAGTTGGCCGCAAATTTAGGGCTTGAAGTG
TCCGAGCTGGCACCAGCGCCAGAGAACGCGGCCAACACCCGGTTCAAAGCAAAGCTGAGA
GGAGGTTTGGCCTCCTTTCTTTTTGAGTACGACGGCTGTTTCCGACATGCAGAAGGCTCC
AGCCATGCAGAAATGTTGGGTATCGAGCAAGAAGACGACATAGGTCTTCCGAGTCGAGCT
GCTGACGCCATGCTCCTGGAGAAGACGCTTTACCAGGTAATCGCTCGGGCTAAGTACATG
CTCGGCAAAATCGACAGCAAGTTTGTCAGGTCAGAACAAGCAATCGAGTTCCGTGAGCAA
CTTGCTCCTGGGATCTTCAAACCAGGTTACAGAGGTTTCCGATTCAAGGAGGCTGCCGCT
GGTGATTTGCCTACTGTCATGATTGACGGTCGGAAGTTCAACTGCGTTGCCAGCATAGCC
AGAGCACATGGACTTGATCCGGTAACGGTGCGTAGGCGCATTGCGGACACCGGGAAGGCC
GCAGACAAACTCTCCAATGATGAGTGGAAGCTCATCCTCGCCAAGAAAAAAGGTAAAGGG
AAACCCTTCACCTATCTGGACAGGACCTACAGCAACATTGCTCAGTTCTGTCGTGAGCAT
CAGCTCAACACCAACCTTGTTTACCAGAAAGTCAAAGACCGGGCTGATAGCGCTGATGAA
GAGTTCTGGGGACTGATAATCGAAACATGCAAAAGGAAAAACTAATGGCAGAAAACAAAG
AGATCCCCTGGGAGAAAGAACTCATTGAGAAGTACATGTTCACCCTGCATAAAGAGCAGG
TGAAAGACCGGCGCTGGCGGACCATGTTGCGTGTGCTTCGCGCATCTGGTTTCGTGCTTC
TCATGATCGGCTTCATCATTTTGGCATCTAATCCGGGTGGGATGCCGTGGCAAAGCGCCA
AGGCAGGAGCCCCTCACACCGCGTATATCAACATCCGTGGTGAAATTGCTGCTGGCACTC
TGGCCGATGCTGATCACCTTATCCCGTCCATCCAAGCTGCATTCGACAACCCGAACTCAC
AAGCTATCGTGCTTCGCATAAACAGCCCTGGCGGTAGCCCGGTTCAAGCAGGACGGATTT
ATGACGAAGTGAAGGCGCAGCGAGCCCTTCATCCGGAGAAAAAGGTCTACGCCATCATTG
ATGACATCGGTGCCTCTGGCGGTTACTACATCGCCTCTGCTGCGGATGAAATCTATGCTG
ACCGCGCCAGCCTTGTCGGTTCTATCGGCGTCATCAGCTCGGGGTTTGGATTCACCGGCT
TGATGGACAAGCTCGGCATCGAGCGCCGGGCTATCACTTCCGGAGAGCACAAAGCGCTTC
TCGACCCATTCTCCCCTCTTACCTCTGACATGAAGAAATTCTGGGAGGGCGTTCTATCGA
AAACCCACCAGCAGTTCATCGAACGAGTGAAGGCTGGGCGGGGTGATCGACTGAAAGACG
ACCCAGAGGTGTTTTCTGGATTGCTCTGGAACGGGGAGCAGGCCAAAGACATTGGGCTGA
TTGATGGCCTGGGTAGTTTGAACTCCGTGGCGCGAGACGTCATCCACCAGAGCAACTTGG
TGGACTACACACCAACCGAAGACATCATCCGGCGACTGACCCAACGAGCGAAGCTCGAAG
CCAGTTCCTTCGTGCAAGAACTCAGCGCTGTGAAAGTTTACTGATAGGAGCAGTTTGATA
TGACAGAACAAAGTACCAAATCACTACGTCTCGGGGTTGTTGCCGCCATTCTTTTGGGGC
TCGTAGGAACCGGGTTCGGGATCTACCAGTTCGTGAAAGAGAAGGATCTGGCGCAAGAGA
TCGCCAACGTGAAGTCTACGGTCAACCAGGTGAAGGATGCCGAAGGCGTCACCTTCAAAA
GCAAGGCAGAGTTTGAAGCTGCTGTGGCCGAAAGCATCAACAAGTTTGTCGCGCAGAAAC
AACAAGCCGACATCGATCAGAAGTATGCCCAGTTCGAGGCAGCACCTGAGAAGGTCGAAG
AAGGCAAACACATCTACGGGGACCTTGGTGCTCGGTTCACGCTGGTGGAGTTCTCCGATA
TGGAGTGCCCATTCTGCAAGCGATTCCACGACACACCCAAACAAATTGTGGATGCCAGCA
AAGGCAATGTGAATTGGCAGTGGAAGCACATGCCTCTCGATTTCCATAACCCGGCAGCTC
ACAAGGAAGCTCTGGCCGCTGAATGTATTGCTGAGCAGAAGGGCAACCGTGGCTTCTGGG
TCTTCGTTAACGACATTTTCCACCATACCCAGGGGAATGGTGGCGGTGTTGCTGACCTGG
CCTCTGTTGTCACTGGTGTTGGTGCTGATCTGGATGCGTTCCGGGAGTGCCTCGGCTCAG
GAAAGTACGAAGACAAGGTTGAAGCTGACATCCAGAAAGCCAAGAGCTATGGCGTCAACG
GGACACCAGCCACTTTTGTAGTGGACAACCATACCGGCAAGAGCCAGCTTCTCGGCGGCG
CTCAACCGGCGCAAGCCATCATGGCCGTGATGCGAAAAATGATGATTGAGTCGCAACAAG
ACGACTCCGCGAACCAATAAAAACTTTCACATGTGAAAGTGGAGTGAGAACTGATGATCA
AGATTACAACTAAATTGGGATGCCTTCTGGCGGGGCTGCTTGTCCTGTCGGCTTGCTCAA
GCGTCCCTCAGCCAAACAACGAATACGCGAAGGCCTTGGATGATACCAAACAGGTATGCG
CTGCCTGTGCCCTGGTTGGTAATGACCTGCTGGTTGCCCTCAACAAGTCATGCGACACCC
CAATGACCCCCGAGACTCTTACAAGCGTCATGAACAGTAACCCGATGTTTGCCGCCATGA
TGGCAATTAACTCCATAGGTGGAACCGACTTCTATCAGGTTTACCGTGATGCGGCCATAG
ACACCCTGCGGTGCAATGAAATGGACAGTTGGCCTGATCGGACCAAGGTGCGTTTCCAGC
AGCCCGACATGCAAAAGGCGCTGGCCTTGAGGGTTTCTGCCAGACAGCAGAAGGCAAATT

AACTTTCACATGTGAAAGTCAGGCTCTCCGGGGCCTGATTTCACTATGAGGTGAATCTAT
GGAGTTACAAGAAGCAAAAGAGGCTCTCGATAGCCTTCATCCACACAAGGCCTCAGCGCC
TTTGAGGCTTGTCATCCACCAGCCAGGTGGGATTGGTGGAACCCCTACAGTGGGGGTGAA
AGCTATTCACGCAGGCTTTGACTGGGACAGCAACACCATCCTGATCTATCCGGAAGAGCA
GCTCACCCGGTTGACGCCGGATGAGGTCGCCGCCATCACGAAGTCAGTATCGAAAGGACA
GTCCTGGCATTCATATCAGCAGTTCAAGAAGTATCGGGAGCAGTTGGCCGAAGCCACGGA
AGAAATTAATAGGCTCAGGGCTGAGCTGGGCAGGTATCAGAATAACGGGAGGGGGTAATG
CTAAAACGCGGAATTATCAATCTCGCTGCTAGTTACATCATTGTTGATGCCCTACTCCGG
AATGCAGCAATTTGGATCTTTGGCTTGTCCTTCTCCGTTGGTGGCACTTACGTCACTGGC
GAGGCCAGTACATGGGTCGTTTACCTAGCCACTTCTGGTGCCATGACACTCTGTTCTGTC
GTAACAGCCTACCTGCTCGTGACCTATCACCGCTGGGGGCTGATGACGGCCAGGGTCTGG
TTGCTATTGAGTGCCTGCCTAAACGGCTATGCCGTCTATTTGAGTAGCCACAACATCCAG
TTGGTGGTGGCACTGCTTTCCAGCCTGTTTATTGCTTTGTGGATGCTCAAGACCCTTGAG
CAACCAGCAGTGAAGGGGACGTACAAGGTAATCGCTGATCTTCACCGTCAGCTATGGGGA
ATGTTGAAGGGGCAAACACAATGACGACGAATACTCAAAACGCGAACGCCAACCAGGTTC
GGTCTTTACGCGACATGCTTGTCCCCGCCCTGTTGTTCTATGTGGTGATGACGGCCATGT
TTGTGGGTCTTGACGCATTCATGGATAAACCCACCAGCATGAACCTGCCATTTATGCCGT
TCCTTGTCTCGATGGTGAGTTTTACCAGTGACGCACGTCGAGCGTGGGACTGGCGAAACG
GGACCAAGGTTGTAGCTGTATTGACTGCGGTAACCATGCTGTTGGCATTCATCTATCAGC
TCGCGGTCGGAGAGATGAATCTCTTAGGGGTTGGTATATACCCGGCCACAGCCATCCTTC
TGTTGGCAATCTCCTGGGTGATTCGCGCTATCGGAAAGACAGCACCTTTCCAGTTCCTGG
GGAGACACCTGGCGCGGTTTGGTGCATCGAAGTGGGTCCAGCGTACCGCCGCAGTCATTG
TGCTCGCGGGAGGCCTTGCCATCACTATCTATGCCTACTGGCTTAACCACGGGAGCTGAT
GATGATCATTGCAACGAAGAACGGCTTTCTGGTGGCCGCAGAACTAATCAGGGAAGAGGC
CGGGTACTGGCTACTACAGCCTCGTGACCAAAAGACGCCGGTCAGGGTGAATAAGCAGGA
TAACAATAAACGCGCTTTCACGCATATGGGAGACGCCCTTCGCTGGGCCGGTGATCCTGA
GCTTGCAAAGCAATTCGATGCCGAGGGGGAAGAACATGCAAATTCGTGACTACATGACAA
AGTTGTTTGACGCATTTGGTGATGTGGAGGAAGTCACCCGAGAAATGCTTCTGGAGCAGG
CGGAGCTCATTCATACGATCAGCGATAAGTGTCAGAGCACAGGCCTGTTTCTGGATAGTC
AGGTTCGTTTCAACCAGTTCGTTCAAGAGATTGAGGCTGACGACAAGGTAGAGGATCGGT
TGCTTCATGCTTGGTGCTGGGTAATGGACCGAATAGTGAAGGCTCCAACATCCTTTCACA
TGGATGGGGCTGTGATTTTGACAATGCCTCTGGTCGCCAGATACCTGCCACCGGTTGAAC
AGGAGCCGGAAACCATCGTGGTGAATCTCGATGAGGACTACAAGGCTCCTGTAGGCAACC
AAACGCTCTGCGAGCTCGTTATGGAACGGAGGCATTGGCCGCAAGGTGCAACCTGTGCGA
CCCAAGAAGCGGACGGGGGAGTCCTCTACTGGGACGCCCCGGTTGATGTGGTAGAGGAAG
GCAGAAAGGTCGCCGGTAAGCACGGCATGATGGCCGAGATCGGATTGAAACATCAGGTTG
ATGCCTGGTATGCAGATATGGACGAAACACGGCTCGCAACCGATTGGAACACCGCCGTCA
TTACGCCTCACTGCTTGCTGCTTTCCTATCTGGATGTGCTTCAAAAGAACAAAGTTCCCT
TTGATGAAGGTGTGCAGCTCGCTGCCGAATGGGTGAAGCAACTTGGCGGGGAATTTCGTG
AAGACACTGAGGAAGCGCCGGAAGCTGAGGCTTCAGTGCTTTCCCTGGGGAGAGCCACGG
CTCATTGCTTTAAACCCTATCCGGACACAAAAAATTTCTATTACGAGGCCTAAGCCTCAC
CCAAACAGGAGGATCAACATGCGATACCAGGTATTTAAAACGAAGGAAGGGGGCCAGCCG
GTGTTTACGGCACCGTGGTACTGGTTGGCCTCTGCCATTGCTCACTGGTCATCTCTTAAC
TGGGATGCCTGTCGAATCGTAGACAGCAAGGTGGATGAAACAAGGCTCTGTTGGGCCAAG
GCTCTACCGGCTGGGAAAAAAAAGTAATTTGGATGGAGTGATTATGAGGTTAGGTTTTGT
GAAAAATACGGGGGTTTCAGCGTTCCTGGTAGCCATGCTGGTGGTGGCCCCTCAGGTATG
GGCAGAAACCTTTACTGCGAAGGTTGTGGGGGTGTCTGACGGTGATACGGTCAAGGTCCT
GACTGAACAAAGCTGTGACTCCGGGAAAGATTGCCGGAGCGGCAAAATCCAGTACCGGGT
AAGGCTGGCGGAAATCGACACCCCAGAGAAGAAACAACCCTACGGCTCAAAGGCGAAACA
GGCGTTGTCCGATCTGGTGTTTGGTCGAATGATTAAAGTCGAGCAAATCGACAAAGACCG
TTATGGCCGTCTGGTCGCCAACCTTTATGTCGATGGCAAATGGGTCAATGCCGAAATGGT
CCGTTCTGGGAGTGCGTGGGTGTACCGGCAGTACGCCAAAACACCGGAGCTGTTCAAGCT
GGAGGCCGAAGCCAAAGCCGATAAGCGAGGTCTCTGGGCATTACCGGAATCGGAGAGAAC
TCCCCCTTGGGAGTGGCGAAGAAAGCACTAACCACACCCCAGAGTATGCAGGTGTAAACA

GTAACCAAGAAAACAAACAGGAATAACGATGAACAAAAGTGAACTGATTATGAAAGTGGC
CGAAGACGCTGACATTAGCAAAGCAAAGGCCGAAGCTGCGGTAAATGCGCTGATCAACTC
AGTGACAGAGGAGCTTAAAGCGGGTGGGACAGTAGCGCTCACTGGGTTTGGTACTTTCCA
CGTTAAGGAACGCGCAGCGCGAACCGGGCGGAATCCCCAGACTGGAGAGAACATCCAGAT
CGCGGCGGCCAACATTCCTGGGTTCAAAGCTGGTAAGGGGTTGAAAGACTCCGTGAACTA
GATCTTAATCATGTGACACATGAATAAGGTTTACATAAGGCTCCTTCGTGGGAGCCTTAT
TCATATCAGACATGAATAAGGGGAAGGGATGAGATTGTCGTCAACTCAAAAAGACGTTTT
GTTCATTCTATACGCAATTGAGGCCGGTGGAAAAGCCGAGCCTGTACCAGGTGTAAAAAT
ACTGGAGATGATCAATTCAGCTCGCCAGAGCGGTATTCATGGAACGAACTTCCGAACGTC
ATGCCACACGCTGGTGGAGAACGGCCTGTTGAACAAGTACCGGAATGCTTCTCTAAAGCT
GGCCTTTAGGCTGACCGACGATGGCAGGGAACGTGCAGGAGAGATATACCGAAAACGGCT
GGAAGAAGAGCAAGAGAAGTAATACGCCCCATAACGGGGCGTATCGAGTTAAACGGCAAA
GCCGTAGTGGGTGCCGTCCGGAAGTTCAACATCGAGGCGTAGCTTGCCACCGGAGGCCTC
AACGTACCGCTTAATGGACGACAGCTTGAGATCTCGTCCAGGTTTTTCCATCTCGGAGAC
TGTCGGCTGTCTCACACCCAGAGATGCAGCAATTTCCCCCTGGGTAAGGTTCATGCGATC
ACGAAGCTCTGCCAAGTGGATGTTCAGTAACATCTCAGTGGCCGCTTTCTGTGCTTTGGC
AACAACCTCAGGCTTTTCTGTTGCCAGCATTTGGTCAAGAGTTCTTGCCATAACGTCACT
CCTTCTTCAATTTATCCAAGTGCGCCGCAAACTCACGGTCTGCAATTGGGATCATTACTT
CATAAAACCTTTTCTCGTCCCCGGTCTTGTTACCGGCGCAGAGAAGAATCCCCTTACGCT
TTGGATCGAACGCAAAGAACGCTCTGATAGGATCTCCTTTGCTTTGGACCCGAAGCTCTT
TCATGTTGCTGTAGGATGAACCGTTAACAGTATCCGCGTATGGCCTCGACAGCATGGGGC
CTCTATCTCGCAGCACCATCATCGAAGCCAGCACGTTTGCTCTATCGGTATCATCCAGAG
CATCGAACCACTCATCAAAGGTGTCGGTTGTCTCGATGACCCACATGATTTCTCCTTACT
TAATATAGGTGCAATCCTATATAGATTCAAACCTATAATCAAGAAAAATCGGCGGCGATG
GGGGCCGAAATGTCAGTAAGTAGTCATGTGACTAAAATAGGGGGTAGAATAAGCCTCGAT
ATAGTCATGTGACTAAAAGGTGTCCTGAATGAACAACCTACCCCTACTGCTCGATGCAAG
AGAAGCCATTGACTACTACCACCAGCATCCAGGCATGACTGATGCAGAAAAGGCCTATGT
GGTCGCGTTCCTAAGCGGAGAGGGGCGTTCAAACAGTCAGATCAGAGAGGATCTGGGTAT
TGAGAAGGTCTATACGGTTACACACCTGAAACGCGCCGGTACGCTATCGGAGGAAGAGCT
AACACTCTGGCTCAGAAATCCACGCAAGATCACCCTGGGGCATGTGAGGGCCGTGGCTAA
GTTACCTTTCAGCAAGAGAGAAAAGCTCCTGAGGGATCTCCTGCATACCAGGACACCGGT
TCATAAATTTGAGGCGATAGCGAAGGGCAAAGAAGTGGACAGGGATGCAGACATTAAGCG
CCTGGAGACCCTGATGAGCGATGCTACGGGTAGGCCTATCAAAGTTCGCTATAACCCCGC
GAAGCGCTCCGGGGAACTGACGCTGGGATTCTTCACGCTCGATGACCTGGATGACGTGTG
CAAGGCTTTGGGGTTTGATCCAAGCGAGCAGATGTAAACCAGAAAACTTTCACATGTGAA
AGTCTTGCAAGGTGTCTTTTTATTGTTTACAGTATCATAAAATGAATCTGTAAAGGTGCG
CGATGAATGTGCTTCCAACGAAAAGCTGGCCTTGGGCGTTATAACTCAACCAGACCTGAG
TGGCTGGCGGCGTACCGAAACGCAAGATGTATGAGAAGCTCAGGTAAAGAACCTGATGCT
TCTCTTTCCGGGATAGCTTGGAAGGCGCAACTCATTGTTGCCCATGAAAGAGGCAGAATT
GACAGCCTGGCTGTCCCTTTGGATGCCAGATTGATAGCAAAGAACCTCATCGACGAGATC
CTTGCTGAGGAGAACTGATGGACGAAAAAATTACCTACGAAGAAATGCTTGAACAACTCG
ACCAGAAAGGCATCCGCGTCACCAACGGGGCGAGACGGCTTTATGTCGCGTTGAACAACG
GAGTCAAAGCTGAGGTGCTGGGTAACTGCGGTCCCGCCACAATCAGCTTGGTTGACGGGA
TGATTGTTGTGGAAGAGCAGACTCTCCACTAAGGGCAGCAGCATGGGCAAAAAAGTACAC
ATCATTTGTGGAAAGTGCGGCAGTGACGAGATGAACTTTGTCATCAATGAACACTGTCCA
GACGATCCCCAGAATGTTGCCAGTATGTCCTGCTCAAATTGTTGCGAACTGACAGGCATA
GCCGAGTGGTCGGAATTTAATGGACGTGAGCTGAAAGGTGAAGCCGTCGCCCTATCAACA
CCGGCAAACGTGAATGCTCTTCTAGGATTGCTTCGCCAGGCGATGGACAGCGTTGAGTAT
CGCCTCTATGGCGGGGACATGTCATTGAGCGGTAATGATGCTGCCGAACTCATAGACCTC
CAGGAGCGAGCTGAGAAGGTCTTGTCTGTTCTTCGGAGTGATCAGCATGAGTGATTTATA
CGAACCGCTTGAGTTTGTGTTCTGCGGCTTTAGGAAAGGGGACGCTGGGCTATTTATCTC
GGTAGCCACTTTGCGCGATGGTGTTTTAGGCCGTGAGATGTATTTCTCAAAGGGAAAAAG
CAAAAGGCGCTGGGTTGTCGGTGGCATTTACTCAGGGGCCTCGTTTTCGGACAACGGAGC
AAAAGGTCTGGATGATGCTCATTACGTTAAAGCATGGGAAGTGCAAGGCGACAAGATAGA

GTGGCAAGCAAAAAGCGAGCAAGCCGAAGCCTTGGCGCGAAGCGAGAAACTTGAAGCCGA
CGATAGGAAGCGCAATGAACTCGAAGAGCTGATGCTACCCATCAGGAAGCAATATGGGGC
ATTAACAAAGCGACGTGATAGGGCAGGGGCCGCAGCTCTTGAAGAAGCTGTCCTGAGAGC
GCTGAGAGCGCCCATCAGGAAGGCTGAGGAGAAATAACCGTGGAGCCAGTCTTAGCTAAA
GCATTCGCCGGTTTTAAAGTCGTAGCTGTAAGTGCCCATCAGATTGGCTTTTCGGCTTTT
CTTGCCATTGGTGTTTGCTGGCGCTTTAGGGGTCGCAAGTACGCCATAGAACAGGATACC
AACAAGTGCCGCTTGCCACGCGAAAACAGCAACCAGAATCCAAAAAGCAGCAGCCAGAAC
CGGCAGTTTGTGCAGAAGGTTGAGCACCGACTTGGCCCAAATCACTGCCGGGATAGCCAG
CACTGCGTAACCCCAGTTGGTGGCCTCAAGCATAGCCAGGCCAAGAATAACAACAGCCAT
CAGCGCGATATTCAGTACATGTTTCATGGTCATCTCTCCTTTTCAACTTACACTTTTATC
CTATCACAGCATTAAAAAATGCAACTGTGAAAGGGCATTTTTCATTCAAAAAGAGGTGAC
ACTTTGAGTGCAATTATCAGCAAGTGCGGCATGTACAGATACCGTCTGGAAAGGGATGTG
CAACCCGAAGGCCTTGTGTTCGGTTATTTTGGTGTAAACGGCTCTACGGCCACGGCCACC
GAGGACGATCACACGGTCAGAAAGTGGATAGGGTTTACCAAGGTCAACGGAGGGAGGCGA
TTCATAGTCGGTAATGCTTTTGCTTTTCGGGCGACCGACGTTCGAGAGCTGGCTACGGCG
GTTGATCCTGTCGGGCCTGAAAATGAAATCCACTTGGAAAGGATTATTCGGGATGCTGAT
GTCTTGGTGCCCTGCTGGGGGAGCCGAACTAAGCTGCCTAAGTCTTTGCATGTTCATTTG
GACAGGCTTTTAGAGCAGCTCGTTGCATCTGGGAAGCCGGTATTGGCATTTGGCGTGACT
GGTTCGGGGGACCCGAAGCACCCACTAATGCTTGGATATTCGACAAAACTTGTGCCCTGG
GGAGGAAAATAATTATGTCGATGCTTGAAGCACGTTACTTCGTGGCAAAGATAAGCGATG
CACAGGCCGTGCTCTGCGATGAGGAGCTGGCGACACTGGAAAGGCTGATCCGGAAGGTTG
ATGATGGCCGCAGAGCAAATGGCAAGAGCTCGCTTACTTGCGTTGTTGTCGAAGAAGATT
GGCCGAACTGGCAGCAAACGGTTGACTCTGTACTTTCACTTGCTGACGGGAAAGACAACG
ATTGGACCAATGCCACACCAGAACAAATCAAGGCCTTTTGGGTCGATGACTCAGTTTGGA
AAACTCTGGATGGCCGGGATAAATGGATTGAAGATCTCGACCTGCTGGTGGACGGTTCCC
CGGTGGCTTCTGAGTGGGAACCTTTTGGCTTGGAATCAGGGCAGTCTGTCGTCATACGAG
GCGGATGGATAGAAGGGAATGCCTTGAGTGATGATGGCGTACCGCTAGTGCAGGCATTCG
TCGCTTGGAAACAGGGCCAAGACAACCACTAAAGGAAAATTACTTCGGAAGGGCGGCTAT
GGCCGCTTTCTTTTTAATATTTACAGCATCACATAATGATACTATGATGTTGTTAATTTA
CTTGGGGAGGCGCTTATGCTCACTGCTAAATGTATAGGGTGCGGATGCACCGATGATCAC
GCTTGTGTTGAAAACGGTCAACCGTGTCACTGGCTTCGCGTGAATAGAGTGGAAGGTATC
GGGGTCTGCTCTTCGTGCCCGGATGCTCTCAATCAATTCGCATCGACCAGCCAGGAACAA
GCAACTGGACAGGATGACTACCGGAACAGTCCAGAATGGAAGGATTTTTCATCACGCATT
GGTAATGCACTATGCGGGGGCCGGAGCAAAGCAAAGTAAGGTAAAAACGGCTATGCCGTT
TTTTATTTGTCTATTACGGCATCACATTATAAGCCTGTAAATGCTGAATCTGTAATTGCC
CAAAATGGGCGTGAGCAGGGGCGGGGTGTGAGGTCGTTCTAAGGCAAAATACTTTGTACT
GGCAGAACAAAGAAAGAATGAACTTTCACATGTGAAAGTTTTTGGGGTACGGGATGGAAC
AAGCAATTCAAAGTTACTTGGCTGACGACCGGCAATATCAAGACAGGATTACCGCAGCTC
TCAGCCAGGTTGAAGAGAAAGGTGCAGAGTACGAAGCTCTTTGCCAACAGCGTGCTCAGT
TGGGGATCTGGCAAAAAATTATAACGTTCTGGCAGTTTCGTCGAGACATCGCCGTTATAC
GTTCAGCACTAAAAGGCCATAACAGCGATTTGCGGTATTTGCGCCGTGGGAGAGACCAGC
TCAAAGAGGGATTGGTTTCTCGGGCCGTGAAGCAAGCGATTGACGGTAGCCAGATTCTGG
AACGAATCACCCAAGCTCAAGATAGGCTTGACGCCGCATCTCGACTCCACGAGAGCAACA
AGCGACTGGTGGACATGGGGCAAAAAGCCTTACGTGAAATCAGTGAGGCCTCTTCCAGTG
TTTCATCAGCACAAACAATGGAGGTTCTCGACCTTGTGACTGACAACAAGGGCATTTCCG
TCATGTCGTCGATGTCAAACTCCTCGGCCAGCAGTGAAATAGACGATGCGAAGAGAGCTG
TGAAAGCCTTCGCAAATGCGCTGGGGGATCATCGTGACATTGTGGGCTCACTGCACCACT
CAATGGCAACTGAGTTTATCGATCTGGGTATGGACTTTGCCGGTTTGAATGATGGTTTCG
ACTTTGGGAGCGTCTTCTCGCTGTTCAGCTTGTCGTCTGCCAGTTCCTCTCTGGACAAAG
TAGAGTCTCGCGTTGAATCTCTGATGCCAGATCTAAGACGAGCCGCCTCCAATTCGGCAG
CAGAGTATGCCCGTGTTAACGAAGAGTTCTTTGGCCTGAAACAACAGGCCTGTTGCCAAG
TGCATGAGTTGCTGGTGACAAATGGTATAGACGTGTCAGTCAAGCGCGTTGAGTCCGCCG
TGAACAGCTACAGAGTTGGAAGGTGATATTTACAGCATCATAAAATGAACTTATGATGTG
AGGTGATCAACAAGTCGAAATTTATTTAAAGGATAAGCGATGAGCGATATTACACAGCAA

ATGGACAAACTTGAGATTCCAATCAAGTTGTCCTTTCCAGTCATCAACGTTAGCACTTTT
GAGCTGGGCCGCGCAGAGAGCGTTTTCTCTGACATTGCAAAGAAAGTCGGCAAGCACTTT
ATTGTAATGCCATTCAAGAAGCTGCCTGATCCAGGCACGATGAAGGCAATGGTGGACGAG
TCCAAGAAGTCCTCCAAAAACGGAGTTGTCGTGTTCGACACTTTCTTTTTCGACCGCCAA
CGTGCAAACCCGGAAACACTCCCAGCCCTCAAGTCATCACTGACCTATCTGGAGAATGAG
GGGATCAACTACATCATCGCTGGCAAAGACGTCTTCAATGAAGAGTTCGTTTATCACATC
GATCTCCCGGCTATGAGCAATCAGGAAATCCTGAAACTGCTCCAGACCTGTGAAGATAAC
GTGAAAGATGGTGGAGTCTTCGAGAGCAACGAACGTGCTGTCATCGCAAACCACGCCTTG
GGCTTGTCACACACCCAGATGAAGAACGTCTTCACCTACTCCGCTTACTTGAAATTCAAG
GGTGAAGAATACCTGGGCGAGATCCGAAAAGAAAAAGCTCACATCTTGCGTGATGTCGGC
CTCGATGTGCTTGAGGCCATTGATATTGGGAATGTCGGTGGTCTCGAAAACCTCAAGGAG
TTCCTCCAGATACGTAAAGCCGGTTGGGACAAAGACCTTCCGGTAAAAGGTGTCCTTCTG
GCCGGTGTACCTGGTGGTGGTAAATCGCTGACGGCAAAAGCCGCTGCCGGTGTACTTGGC
ACTACCTTGGTTCGCCTGGATATGGGCCGTTTCTATAGCAAGTATCTCGGTGAAACCGAG
CGCCAGTTCAATCGTGCATTGCAGACCATTGAGCAGATCGCACCCGTTGTTGTGTTGATT
GACGAGATGGAGAAGTTTTTTGGTAATGCCGATGGCGAACACGAAGTATCCAAGCGCCTG
CTGGGCTCTTTCCTCTACTGGCTTCAAGAGCGCAAGAAGAAGATCTTCATTGTGGCGACG
GCCAACCGGGTTCAGTCGCTGCCTCCTGAATTGATGCGAGCTGGCCGCTGGGACCGAGCA
TTCTTCATTGATCTGCCAAGTGTGGCTGAGCGCCAGAAGATTTTTGAGATCCACCTCGCC
AAGCAGAAGGCCAACATCGCCGCGTTCGATATGCCTACGCTACTTCGTACCACCGAGGGA
TACACCGGGGCAGAGATTGAACAGGCCGTCATTGACGCGATGTATCTGGCGAACGCTCAG
GACAAAGAGCTCAACAATGAAGCGCTGGTGGATGCGGTCACTCGCATTACCCCGACCAGT
GAAACTCGCCGGGAAGACATCAACCAGATTCGCAGTTTGCGGGATCAAGGCTTCTATCCG
GCCAATAACTTCGATGTTCAAGAGCAGAATGGCTCTGGACGCAAACTTGCCATCGAGGAC
TAAGCGATGGAGTGGATTTTTTGGACTGCTCTTGCTGTGTCATTGATATTCCACTCAGAA
GTCAGCGACTTAATCAATGCTCTAGCTGAGTGGGCTAGGGAGCGAGCTGGTGCGGCCAGG
AGAGAGCATCAAAACGGCAGAGAGGAGAATTGACCGTTATGTACAAACAAGACATTGAGA
AGGGGATTGAGCTGCTCAAGCTGTGCAGCAAACTCCAGTCAGAAAAAGATGGAGTGGATA
GACCAGAGCCTCTGGTTATCGACAAGAGTAAGGTGCTGGACCAGTTCGCCCGAGACGTTT
CCACATCGATCACCTACATGAGTTCGTTGTTCAAGCTCATCCCCATGATGGAAAACCTTA
CCGAGCTTGGCCGAAAGCTGGAGAAAGAAGGAAAGATCGAAGTTTCTCTCGGGCAAGACT
ATTCCATTGCCGCGCTTAACTTTGTTATGTCGGAACACGGCATGACACCCGAAACAACTC
AGGAGTGATGAGATGAGCCATATCGTAAAAGGCAAGGTGCAGGTCGCCTACAAAGACAAA
GAGCTGCTGTTGAAGGCTCTGGAGGGAGTTGGCGTTGTTGTTGAAAACGAAAAGCTGTAC
CGCGTGGGCGCTGGCTACACCTTCGAGAAATACCCGATTGTTCTGATCGACCAGAACAAC
AAGGAGCACCGGATTGGCTACAAGGAAAAAAACGGTGTTTGGGAGCAGTACCAGGAAAAC
TACGGCTCTTATGGCCGCTGGACGCAGCAGGCAAGCAGCAAGGTGCAGGATCGCTACATT
GCCTTCCACTACGAGCAGCAGTTGAAAGAGGAAGGTTTCAGCGTGACGGTGAAGCAGCAT
CACGATGGCACTTTGGAACTGGAAGCTGAGGAAGCTGTTTGGTAATAGCGCCAAGGCAAT
CAGATTAGTTGAACTTAACGACGAGGTGACAAAGTGAAAAAGGTAAACATCAAGATCAAG
GGCGGCAAGATTGCTGCCGATTTCACGGGGTTTCAGGGTAAGACCTGTGAAGCGCTGGAA
CAGCGTATTCGCCCGGAAGAGCTCGAAGTCGAAGAAAAAGAGCTGAAACCCGAGTACCAC
TTCAATGCTGGTCAAACCCAGCACGAAACGGAACAGAACGAATGGTGATGAGAAGGACAG
GGCGCGTCCCCCTTCTTTTGCTGGTTGGTCTCATGGTCGCAGCTCCTGCGGCCTTTGCCA
GTGACTTTGTGACTGGCGTTATGGTGGGGCAAATGCTCTCAGATGACTCAGGCAGCAAGA
AGATAGAGGACACTGGCCCGGAGAGCAAAACGGTCATTGATTACCATGACGGTCAGCCAG
TTGTGACCAAAGTGGAGGCCTTCAAAAGCAATAAATGGCGCAAACTGGATGGCCCTCAGG
GCGGCTATTTTGTTTGCCCAGGTGAATACCGTAGCTATTCGGGAAGGCTGCGATGCCGTA
TTCATGATGACGGGTTTAATGGCTTTATGGGCGGTATGACTGATGCTCAGGAGCTGCCGC
TCCAAGAAGCTCTGACCAAGTTGGAAGGTAAACCCATCTCCCTGCAAACCATTGAAGTCC
ACGGCAACAACCTGGCAGTCAAATACCAGTTAGCACCACCAGCACAAACGCACGTCAAAA
CTCAGGATCTGGGGGTGGTCAAGCAGGATGGTGAAACGCAGAAGGCTGCCAGCCAACAAT
CAGCGTCATCCATAGTGACTGCACCAAAGAGTGAACCTCCCAAGCAGATTAATGCTGCAC
AAGACACTGACCGCAATACCAGTGCGTTTCAAAGTCCGTTCGAGGAGGTAAGTGACTCCA

TGATCGGTCTAATGGATAGCTCTTTCGTCAAAGTCATAGCGGGATTGATGCTGGTGTTTG
GGGTTGCTAGTGGAATTATGAGGCAGAGCCCATCAGGAATTGTGATGGGGATAATGCCTG
CAATTATGATCATGACCGCGCCAACGGTTATTCGTACCGTATTCGATACAGGCGCGGCCA
GTACCAAGCCAGTGGAAGATAGCGGAAGCTCATTTCCTTTCTTCTTGGTGGCAATAGTAC
CGGTGCTGATTTTCTTTGCCTATAGAGCTTTTATGAATAACCGGAGCGACTCCGAAATTG
ATGAGTTGTTAAGAGAAGCCCGACGTGCAGAAAGGGCTGAACGACCAAGCAATGAGCCTC
CCTCAGTGGATGAACTCCGTGAGCGCCAGCAACAAAACACAGAAAGAGAACCGGTGGTTG
TCAGCTCATCAGCTCCGGCACCAAAGGTGCAAAAGGAAGAGCCAATCGAAGTACAGCCAG
GCAAGCGCAAAATTATTTGGATTAGTGGGGTCGGTTATGAGCAAAAAGCGCATCGTAAT
CAAAAATGGTGAGGTCTGCGGGTTTGCCGATGAGGTTTCCTTCAAAGGCCTTGAAGTGCA
GGAATACAGTAAAACAAGGGTTTCGCGCATCGTGCCGACGAGCGGCATTCTAATGATTGC
GTTCTATGTTATTCGCGGACTTTGTTCAGACGAGTCAAAGATTGCGGCATGGACTCGTGT
TTGGCGTTGCCAGTGGAAGGTGCTGATCGACGGTAAAAGCTATGGCCCATTCAGCAGTCG
TGCGGATGCTATCTCGTTCGAGAAGGACGAGATCTACAAACAAGGCAAATTCTTTGCCGA
TGCCACTCACGAGGCGGCAGTATGATGACACGGGCGGCTATGGCCGCTCTGTTATCCGCG
TTGGTAATAGGCCTTGTGTCTGATTTGGCCGCACAAGAGCTGGTGGTAAGCCCGGTGGCT
ATAGACAAGTCCACTGAGGTGGAGAAAGAAGCTACCTTCCACAACCGCAAGTGGGTGTTA
AGGACGGGGCAGGTTAGTGGATTCTTCATCTGCCAAGGAGATAACAAAGACATTTACTAC
CGGCATGACAGGGTGGGCGCTCACTGCCAGAAGACATCGACTGGGTGGCGCAATGTACTG
GGTCTCAGGGACGATGTTCCGGAAGTTGAGCTGAGCGTGTACCTGGGGACCGTTGAAGGT
GTCCCCGTGAAAGTCCTTCATCAGGAAGTGGATGGTTACGATCTGAAAGTGCAGTACAAG
GTTGCACGTAAAGGGTCAGCAAATGAGTGAGAGTGTGCGTCGGTCAACGCCGATGGTAAA
GATTAAAGTCCTGCCGAGTTTCGTAGGGGTTCCCTTCCCGTCGGTGCTACTTATCGTTGC
GGGGCTCCTTAATGGCCCTTTGTGGGGCTTTGCCGCATTCGTCTTTCATATCGTCATCAA
GCGGTACATCTACAGAGAATACCGGAGGTTGCCATATCCGATGCCAACCGGCTCAAGGCC
TATGGATGAGTTGAGCGATCTCCTTGTAAAGGGGATACCGGAGGAATTTTTCCGGCGGCT
TGACGCAGCTTGCAGCTCTGATGCGTCAGTACAAATAACCTGCCCAAAGAAATACCGGCT
GCTCAAAATGTCGTTGAATCGCTATGCCAACCGCGCTGGTTGTTACCCGCAGTTCCGGGA
GGGGGATGAAATAGCGATTGAGGTAACAGAGCAGAGACCAAGAAAAGCAGGAGCAGGGAA
GGGCTTTCCAAGTATTCAAGATCTGAAAGCGTCAGAGGGTGTTCTGAGTGAACCGGAGCA
GCCTATCGAAATTCAGAAAGATAAGCGCAAGATTATCTTGGATTGAGGTGGGTTGGAATG
CGGTCATTTTTATACAGAATATTGCTTCTTTGCTTCGTCATTATCGTCCAGATAGGGCTA
ATCGCCATTGATGCGAGCCCGTTGGTTGTTGCTATGTTCCTCTCGCTAACAGCTTTAGTC
GCTGGGGTCTGGATGGCGTACCTTCATTCCCGATTTTTGATGGCGGTGACTCCAGTAGTT
GTCTCTGTGCTAATGGCGACCCTCGCCATGAAAATGTTTGAGCCGAGACTCTTTGTTTCT
GCCAGCGAAAAAGGCTGGTTCCCTTTGTCATTGTTCGTCTCGATACCGTTTTACTTCTAC
TTGAGTTACTTGCTTGAGATAGAGGAGAAGAGACGTACATTCCGGGCCAATGTAATTGGT
TTTTTGCGTGGAGTTTCGGAAGAGATCCGTCGTTCAACTAGAGAGCATAGAGATCAAGGT
GAGATAAGACACGGTGGAACAGAGCTGGGGAGTATTGACGAACTACAAAGGGGCGAGGCC
AAAGAGCCAGAAGTCATCAGCTCTACATCGGACCAGAAAGAACAAGGCACTCCCAAGCGA
GACAAGAGAAAAATCATTCTGGACTGAACATGGAACAAATCAGAAAAGGCTTAACCCTCG
AATACGCAAAAGAGAAGCGTGAGAAGCTCTTGGCGGAGCTCAAATCAGATGAACACTACA
GCCAGACTGAAACAGTAGCCTATGGGCACCACGACCCGCTCAGCGTTCCTGTGGCAGCCT
GCGATAGTTGTCATGGCCGGGCGCAAATGCAGAAGGTTATAGGTCCTCCTGTGCGCTGGA
ATATGGTCTGCCTGGGTTGTGGGAAGGCGATCCAGCAAATCCAGAAACGACCGTGGCAAG
CAGCAATGGCGTGGAACCAAATTAACTTGGGAACACAGGACTACAGACAACTGCCCCTCT
TTGGACTTGGGAGTCTGTCTCTTGAGTCAGCAAGACAGAGAATGGTGGGAATACGCAGGA
ACCTGGAGCTGAGAAAGAGCCTCGCCGGTATCGAGAGGACGATCGCCCACAAGGAAGGTC
AACGCCCACCAGGGAAAGAGTACCAGCAACGACTGGAAGCATATCTGCAATGGGCCATGC
TGGCGCTGAGGTTGCTCAAAGTCAAAGCAAGTTAGTGGTGAGAAGGAAGAGAGAACCCGG
TACTGTAAAGCGCCGGGTTTTTTATGCCGCCCTGTTTACAGTATCAAAAAGTGATGCTAT
AAAGTGAGCAAGTCACAAGGAGAACCAACGAATGGCTGTTATCAACTCACTCCGCGCTCT
CAAAGGCGTAACCGCCCCCGAAGAACTGAAAAAAGGGGATGGATTCACTATCTCTCCTCA
GCTTCTGCTGGAGGAAGAAGGGTTTAACACCCGTGGCGCTTTCTGCGAGAACTACTACGA

GCGCCCGGACATCAAAGCTGGTATTCGAGTGCTGGCCGATGCTTACAAGCGTGGCGACTA
TGTTCCGCCGATCATCGTCAAAGTTATCGACGGAAAGGTGTATGTCCGTGAAGGTCATCG
CCGTCGCCGCGCCATCCTGCTTGCTATCGAGGAAGGTGCCGACATCCAGTTCGTGCAGGT
CGTAGAGCACAAGGGCGATGAAGCCGAACAGAGCCTTCTGATCGCCACCAGCAACGATGG
GCTCCCTCTTTCTCCACTTGAGCGAGCCGTGATCTACGCTCGGCTTGCAAACTGGGGGTG
GAGCGACCAGATGATTGCCCAACGTGTTGGCCGCTCGGCTGAGCACGTTCGTATCGCCCG
TGCCCTTCTGGAGATGCCTCTGGAACTGAAACGGATGATTCAGGAAGGCTCTGTGGCTGC
CACCTACGCTCAGGAGCTCTACAACGAGCACGGCACCAACGCTGTTGAGATCCTGAAAAA
GGCGCAGGAAGAACAGGCCAATGGCAATGACGGCAAGAAGGCCCCGAAAAAACTGACCAA
AAAGTCTGTCGAGAAAGGTCCCCGCCTGGGCAAAAAGGTGGTTGAAGCCATGCACCGAGG
CGTGAGCTCTATTACCAGTCGTCTGGACAACATCAAGCCGAACGATGACGGTGAAACGTT
CACCCTTACCCTGAGCCGGGAAGATGTTGATGCGTTTCAGGAGCTGAAAGCCAAGCTGGC
GGAGCTGGAGCCCAAAACTGACGAGTCCAATGAAGACCAGCAAGAGCTGGATTTGGCGGG
TAATCAGTAATGGGGCGCTGTACTGTTGAGGATGCCATCCAGTTACTGGAAGGGGGAACC
ATTGTTGCGGTCGTACCGGCGTCATCCGGACCAGATACGCTGGTGGTTTCCTCTATCAGG
CTTGAATCCGGACTGACAGTACAGTTTTCCCCTCCAGCGGCAGCCAGTGTCAGTGTTCAG
CTCGCGGAGCTCGATGATGAACAGTGACAAGAAATCGTCTGAACTAGCCAGGCTGAGGCG
TCAGCATCAGCTAGTTTGCTCTGTCATCGCAGCCTTGCTCATCTGTGCAGGTATATATGG
GACCGTTGCACGTTTGGGCTTCATCCTGATGGTGCCATTGTTTGTTATCGCCTTAGGTGT
GTTAGTCGATGCGTTCCTGCCAAGGGTAAGTTGGTTCTTCTGTGTGCGGGACGTGAGAGA
CATATTTGGGTGGAGAAAACGATAGTGGCGAAGTTGAAGGTTTATGGCGGGATCACTTAT
GGGGTCGAGGGGCAGTTCAGAACTGTCGTTGCGGCCACAAGTAAGAGCAAGGCTGCTTCA
ATTCTGAATATCACGATTTACCAGATGAATAGCTGGTGGACGGAGACTTTTAACAAGTAC
GAGGTCGAGGCGGCGATGTCTGAGCCGGGGGCTATTTTTTCAAAGCCTCTCGATGGCAGA
GGCCCATTTGTAAAGCAGGAAGGATAATTGTGAGAAGTGTTTTTGTATGGTTGAAGGCCG
GGGCAATACTGGCTTTCCTATTCATCGGTATTGCTGGTGCTGTCAACCGGGTCTATTGGG
TTAACAGCCAAGGTTTGAGTCAGGGGTCGATGTCATCCACATGGGTTAAAGAAAAGTTTG
GCCTAGACCTTGGTGCAATGTGCCGTTATGACAAACCCCAGCATGTCGAGTTCAAAGTCC
TTCCTGATCAGACAATCCAGTTCCGCTGCTCATGGTTTGAAGGTGGCGGATTAACCTGGT
GGCCTTTCTACACCGAGCATGATGTAAAGAGCGCCGAGGCGACCTCTGTGCTCAATGACA
TCTTCCAGGGTACGGAGGGGCATGATGAGTAGCCAGCTAGAACTTTTTCATGTCCAAGAG
GCGTATGCCAAGGCCGATAAGCCGCTATCGAATGAGGAGCTCTACGATTCAGTGGCAGAG
CTCGCTGGAATCCCGAAAAGCGCTCTGAATGAACAAAGTGAAATTGGCAAGGCAAAGATC
AAGCGGAGCAAGCTGAAAAGACAGATACGCTGGTATCAGCAAACCCTCAAGTCGATGAAC
CTTCTCCAGAAGGTGGACGGCGAGCGGGGTGTCTGGGAGCTCTCAAGCAAAACCAAGAAG
GGCCTGCATGAAGCGCTTGGTGGCATTCGACTTGTCGCCTATTCAACAAATCTTGGGCTG
GCGGTGTGGTCAAACAACAAGAGCTTTTTCTCTGACCTCGATGAGCCGGTGCATCTGTGT
GTGACATCTCCGCCGTTTCCGCTCCGAATACAGCGTGGCTATGGAAACGTTGATGAAGCC
AAATGGGTGGATTTCATAACCCAGGCGCTCGAACCGATAGTCAAGAACCTGGTGCCAGGA
GGAAGCGTTGTCCTGAATGTCAGCAATGACATCTTCGAGGCCAAGAGCCCCTCCAGATCT
CTCTATGTTGAAAGAATGGTGCTGGCGCTCCATGATCGGCTCGGGCTGTCTCTCATGGAT
AGATGGCCGTGGATCAACCTGTCAAAACCACCAAGTCCAACCCACTGGGCTTGTGTGAAC
CGTTACCAGTTGTGTGCCGGGTGGGAGCCTGTTTATTGGTTCACCAACGACCCTGACAGG
GTGCGTTCTGACAACAGAAGGGTCCTGATCCCTCATACAGAGAAACATCAGAAGCTGATG
GCTCAAGGGGGGGATAACCGAGTGGTCAGTTATGGTGACGGGGCATATCGACTGAGAGGG
AACGCATTCTCTAATGTCACAGAAGGACGGATACCGAAAAACGTTATCCAGCGCGGCCAC
CGTTGTGCGGATACGCTGGAGCTTCGGAGAATCGCTAGAGAACTGGGGTTGCCACCGCAC
CCGGCAATGTTCCCAACAGACATACCTGAGATGGCTATCCGCTTTCTGACGGAAGAGGGC
GACCTTGTTGTTGATCCGTTCAGTGGGTCGAATAAGAGCGGGTTGGCCGCAGAAAGGAAT
AACCGGCGCTGGATCGCCTGTGACATCATTCTTGAGTACATTCGTACCCAGGCGGAAATG
TTCACCGGCTTTGATGGATTTTGGATTAACCCAGCCATAGCAACAGTGGGCGGGGGAGCG
TTGAATTAAGAGAACAATACCTATGAGTTTAAGTTTTATTGCAAGAGTCTTGCGCCAATT
GGCAATTATGTTTGTCCTCTCCGTGCTTATTGTGGCCGGATATATCTACTATGCCGGTAA
ACAGCATCAGCAGGCAGCAATCAACTTTTGGGGTGAGCAATACCAGCCTGATGCCATCTC

GACGCAAATTGATTGGGGGTTTATCGGTAACTGGGTTATTCCTCGCGGGGGGCCAATTAT
TTCTCCGGGCATTGCTGGAGTGTGTCCCAATACACCACTTCCCGTTGTGCCTCTTAAAAT
TGGTCCTGATGGCCGGGGTTACGTTCTCTGTGGTATTGGAAGTGAGGCTGTCTCTACCAG
CTTTGATGTCAATGACATCCAGGATGAAGAAATACGAAACACATTAAAAACGATGTTTGA
GGAAGAATTTGAAAAAACAGTTAAGGGGGATAAATGGACGCTAAAGAACTGAACCACATG
ATAGCTGAGGCCTACAGCCGGGATTTGCAAAAGCCTGAGCTGGTATCGTTCAAAGAGGTG
AGTCGCTGGGGGCGTAAGTACGGTTTCCCCGTCGTATGCACTCTGGCCGATGAAAGTGAA
GAAAAGCAGATCCACTGGGCTGCCAGTTTGCTCATTCAAGTAGCCGGTACTTGGCCGCGA
GAAGATATGCCGGAATTGCTCACACCGGAACGGGGCTCCGCGCTGTTCAACGATGCGATG
CAGTTATTGGCGAATGGGCTTGGAGCAGCAAATCAATTGCGCTGACAGCACAAACCTTGT
CAATAAGCGGCCTTCATGGCCGTTTTTTTGTTTAAAGTTTGTTTTAACAGAACCATAATT
AGATATTGCAGTATCATTTTATGATCGCTACAATCTTTCACATGTGAAAGTTTGGGGGTG
GTCTTGATCACCTGCGAGAAACCAAAATAGAGGTGGGTATGGCAAAAGTAATCAGCTTCG
CCAACCAGAAAGGCGGAGTGGGTAAGAGTACCCTCTGTATCCAGCAGGCCTTTTATCTGG
CGTTACAGAAGAAAAAGAAAGTGCTGGTCTTGGATATGGATGGTCAGGGGAACACGTCCT
CTCGACTGGCCCCCAGACGAGAGCTTGAGGATGGTGACTACGAGCCCATCCTCACAGGAA
CCAAAACCGCAGAGCTGTTCGCTTACGAGCTGGACGGCATTGAGGTCATGCACTGCCCTT
GCGGTGCAGACCTCATTCATACGCCGAAAAACGACCCGGATCTGTTTGAGATGGAGGCTG
TGCCTCTTGACCAGGCCATGAATCCGGCTCGCCATTTGGCTGAGCTGTTTGAGAACTACG
ACTACGTGCTGATTGATTGTCCGCCTAGCCTCGGCAGAAAGCTGGTGGCAGCGTTGGTGA
TGTCTACCCATGTGGCATGTCCAGTAAAGCTCTCTGGCTTCGCTGTGGACGGCGTAGAAG
GTCTCCTGAACACGATTATTGGTGTGCGCGAGGCGTACAACCAAGATTTGGAGATCCTGG
GCATCGTGATCAACGACATGGACCGCTCTGTCAATCACGACAAAGCCCTCAAGTCGCTGG
AGAACACAGTTCCGGATCTGTTGTTTGAGAACAAAATTATGCACCGGCCTCCGCTCGATA
CGGCGACGACTGATGGCATCCCTGTCTGGGAGCTTCGCTATGGACATGTCGCGGCCAAAG
AAGTTGAGGCGGTGTTGGAAGAACTATTAGAGAAGGTGGGCTAAGCGATGGCATTGAACA
ATTTAAAAGGCCTGTCCGAACTTGCTAAAGCCGCCAAAGGCAAGAAAGGCAAAGAGGTTC
TTACCGTACCTGTTGACGACGTTGTATCCAAGGTCCAGGTTCGTAAGCGCTTCCGTAACA
TTGAAGAGCTGGCGGCAACCTTGCTGACCGAAGGGCAGCAGTCTCCGATCATCGTGTTCC
CGAAGAACGAAGAAGGCAAGTTCGTCATCCAAAAAGGGGAGCGGCGTTGGAGAGCGTGTA
AACACGCTGGTATCGAGACCATTGACTTGGTGGTGAATGATAAGGTCCAGAACAACTTGG
ACGAGACTGCTGGTGAGCTGATCGAAAACATCCAGCGGGATGACTTGACTCCGGTAGAGA
TTGCCGAGGCGTTAAACCTGTTTATTGAAGAAGGTTGGAAGCAAAAGGATATTGCTGATC
GGCTCGGTAAGAATATCACTTTCGTATCTACGCATCTGTCGTTGCTCAAGCTACCTGACT
GTGTGCGTGAGTTATACGATAATGAAGTATGTTCTGATACAGAGACCTTGAATAACCTTC
GTCTCTTGTTCGATCTGAATGAAGAAAGATGTCGCGCCGTCTGCGCGGTGGCTATGTCTG
ACGGGATTACTCGTAAACAAAGCCGTGAGCTGCTGAATGATGCCAAACGTATCAAAGACG
AAATGGAAAAAGGCCCACTGACTGGCTCCCACCAGAATGATGAGCTTGGCGCTGGCAACA
CCGACGAGCAATCCCTCAACTCGGGTGGGGATGGTACGTCGGAACAAACCGGGAATGACG
ACCTGAACCTTGCCCAGGAGGAACTGGAAGGGGGTAAAAATTCCAATGGTCAGGACGATG
ATGACGAAGACCCTTTGCGTGATGAAGAGGGTGAGCACAAAGATCCTGTCAAGCAGCCAG
ATAACAGCGGCAAGGACAAAGATGAAGAAGGCGGTGATGCTCTTCCTCCTCTGCCGAAGG
ACAAGGAATGGAAGAACGTCCGGGCTGACAGTTTGATTTTTGCTGTCAACGTTAACCTGG
ATGGCGAGACCAAACGTGGAGTCATCATGACCGACCGTGTTGCTCTGGTTCCGTCTACTG
TCTGGGTTAAAACGCTCGATGGCGAAGGCAAGGAAAAGCATGTTCATGTGCCTGTGTCAG
ACATTGAACTCCTGAGTGTCGAAGGCTAATAAAGGAGGCCCAGCCAGAGAGGTTGGGCCT
GTCAAGAACAGGTGAATATGAAGATCTCACAGGACATGAAAAGAAAATTTGCTTTGGTGA
ATGCCCTGTCTAAAACGGAGAAGCCAAGTCTCCAAGACCTTCACAAGGCAACAAATATTC
CTGAATCAACGATCAAGAGACAGTTGTCTGCCCTGCGTGATGAGTTCGGAATGAATATCT
TGTTCGTCAGGGAGTCTACCGGCGAACGAGGTGCCACCGGCTACTACATGCTGACAGACT
GGGGGATCTTAGACAGGTCTTCGTTCTTGAACCGGTACGGAAAACTGTAAGGTGCCATAG
AGCAGCGCATTGAATGCAAAACCCGCCAATCGGCTAATGCCGATCAGTTAAGACAGATCG
CTTGACGATCTCACTGAAAGGATCAAAATCCGATGACCCCATGTCATCGGATTTTTTTAT
GCAACTTTCAGAAGCTTTGGCGCGTACTCATATCAATCGTCTTACCGAATTCACCTGTCT

GGCCGATGTGCTTGAGCCTGAGTTAATTCAGTCGTGCCTTGATTCACATGGTGTTGCCAG
TTTAAGGCGACGTAAGTTACCTATGGATGCCATGATTTGGGCGGTGATTGGTATGGCTCT
GTTCAGAGGCGAGTCCGTCCGCTCTCTCATCAATAAGCTTGATATCGTCTTACCGCAAGA
GGTGGATTACGTGGCGCGCAGTGCTGTCACGCAAGCGCGCAAGCGGCTGGGCTCTGACGT
TGTTCAGGATGTGTTTAAGCGAAGTGCCAAGACCTGGCATGAGAGAGCAGAGCATCCGCA
TTGGTGTGGGCTAAACCTCTACGGTGTCGATGGTGTCGTGTGGCGAACACCTGATAGCGA
GCAAAACAATCAGGCCTTTGCCAAAACTGCCAACGCTTCTGGTGATGCAGCCTATCCGCA
AATTCGCATGGTTTGCATGATGGAGCTAAGCAGCCACTTGGTATTGAACAGTGCCTTTGA
TTCTGTTGCTGTCAGTGAAATGAATTTAGCCGCCGAATTGATATCAAGCATCCCCAATAA
CAGCCTGACGCTGTTTGACAGAGGCTTTTATTCGTTGGGACTTTTGCACGCCTGGCAACA
GGCTCAACCTAACAGTCACTGGCTGCTGCCGTTGAAGAAAGGGACACAATATGAAGTGGT
ACGCTCTTTAGGAAAACACGACCAACTGGTGCGGTTAACCACCACGCCTCAAGCCAGAAA
GAAGTGGCCTATGCTGCCTGAGAATATTGAAGCTCGCTTACTGACGAAGACGATAAAAGG
TAAGTCGGTTTCCATTTTGACGTCATTAACGGACCCATTGCGTTACCCGGGGGCAGAGAT
AGCGGATTTATATGCCCATCGATGGGAGATAGAAGTGGGCTATCGGGAGATGAAGCAGCA
TCTTCTGGAGAGTCGCTTTACGTTACGCAGTCAATTGCCAGAGCTGGTTATCCAAGAATT
GTGGGGGGTACTGTTGGCCTATAACCTCATCAGATACAAGATGGTGTTGATGGCTAAAAG
CTTACCGTCGTTACATCCGAACCAATTAAGCTTTAGAGATGCGTCGAGTTATATCATTTT
TAAACTGACTCAGCTGTCATCTCAAACGCCTGGGAATGTCCCGAGAGATGTCTTGGACAT
AGTGCGTAATGCGAGGCAGTTCAAGCTTGATGGGAAACGGGATAGAGCCTATCCACGTGC
GTTAAAAATGAGTAAAAACAAGTATCCCATTAAGCCTAAGAAAAATGCCGTTCACACTAA
GTGAACGGCATTACGCCAATCGGCGGGTTTTTTTATGCTGCCACGGTAGCGGCGGTTGCT
GGAGGTTTTTCGGCGTAGTAGGCGATGTTCTTGGTTCGTCTGCTAAGGGTGTCTATGCCG
ACGATACTCAGCGTTTTGGGAGTGGTATTGCGGGACTCGAAATAATGGAATAGCTGGATC
ATGCAGCACTTCACCATCAACGGATCGATGTCTTCCAGGTAGATTGAGGCTTCTCTCAAT
GCTTCGGGACCGTGGTTCTCGATCAGGTTTTCCATCCAGTGAATGGCGTTGATGCCAGAA
CCACAGCAGGGCTCATAAAAGTCTGCCGAAGACTGTGAGCCAACAATGAGTGACATCAAG
CGGCCAATCTCTGGAGGAGTGGGGAAGTAATTGGTGCCCTTCTTGTGGAAGCCAGACATG
CTTAGGACGTAACCCAAAACATCGCTGGTGGGGTCGCGCTTAATGGCGGCAGACAGGACG
TGAGAGAGCTCGAAGGCGACCGGCTGAAGTTCTTCCGGGATCTCCTCCTTGGCTGGGTAT
AGGCCAGTCTGCAAGTATGCCCACTGGTCAATAAAGGCCTCAACAAACCGAGATGTGCCC
ATTCTGTATCGGGACTGCTCGATGAGAGATAAAGCCTTGGATGTAAGTTGCTGTGTTTCT
GGTGACATAAGCGCTCCAGTTTGTTCTGACGAAACATTCAAGAGGTAGAAACGAAGAACC
CCGCCGAAGCGAGGTTCTTTGCTTTCCTGCCTGATCGCCTTAATGCCGAAGCAAAGACGT
CTTGATAAAGTCGGGGCAACCGGACATTTCCCGGTCGATGAAGTCCTGACGTTTCGCCAG
CGGCCATTTCATGAACGCCGCGAGCGGGATGCGCTTATTCGTGTTAGTATTGCCTTGCAG
CATAACAAAGCTCCTTAATTGCTTGATTGTGTTGTAAGGGCTGCAACGAAGGTCGAAGAG
CGATGCGGCCCTTATTTATGATTATTACAGCATCATAAAATAATATCAAGGGACAATAAA
ACGATACCAAGAAACAAAACTTTCACATGTGAAAGTCTCCCCGCTGTCCACCTCGCACAC
TCCAAACGCCCAAATAACTTGTCTATCTGCCCAAAATGGGCGCGTAAGCGTCCGATAGAA
AGTAATTGTTAGTGAGATATTGACTACACGGAATATGGCTATATCATAGAATCATAAAAT
GATACTGTAAAAGTGATATGTATGCCAAAGCAAGCAAATCATCTCCGTTTGAAGAAACCT
TGCGCCAACTGTCCATTCCGGAAGGAGGGCGCTATCGAGCTGGTCCCTGGGCGATTAGAA
GGCATCATCAACGACATCGTTGAAAACGACATGACGACGTTTCATTGCCACAAGACCGTG
CATTCAAAGTCAGGTGGTGAATGGGATGAAGAGGGTAACTATGCACCTTCGGGACAAGAG
TCGATGTGTGCCGGTGCTGCGGCTTACTTGATGAAAATAGGCAGGCCGACAGTGGCTATG
CGAATTGCCTTTGCGTTTGGTGATGCAAAGGTGTCCGACTGGGACGAAGCTCAAGAGCTG
GTTGTCGAGCCTTTGGTACAAGGGGACCGAAATGAGTAAGCGATATGCGGTAGTGCCGCA
TCCGAAACTGAAACGAGAGTACAAAGGTCGGCTGGTCAGAACTACTCGGGTACTAAAAAA
CGGCTGGGGGGTGATCCCTCTAGGGGCTGTGGCAACGGTCACGCATCAGTCTCCCAAGGG
ATCAGAACTGACCTTTGAACCATGCGACTGCTGCGGCCTGAAAGCCATTATCAGTCATGT
AAGCATGGACTCCATTGAATTTATCGAACCGATTACTGAGGAAGAAGATGGACGAGAACA
AGCTCAACATTGAAACGGTCGATGGGCACAACGAGCTGGTAGTCAGCTTTTTGTCCAGAA
TGGTAAGCCTGAGTGACGAGGAAAAGCAGACAGTATTGTCCTGCCTTCCGGGCACCGGCA

AACAAACAATCACCCAGCTCTATGAGGCGCTGCGTTCTCAAGGGCACCAGGATCTTGCAG
AGAAGGTCGAACCATACCTCCAGCAGGGGGTGTTTGGGCCGATCTTCGATAACGCCAAGT
CCAAGGTGTTCGTTCGGGACGAAGCGCCTTTTTTCCTCATGGATGAAAATCCTTTGAACT
GGGATGACGCTAAAGCATTCAATCGCCTACGTATGAGTACGACCTGTGTTCTTGGCCGTG
GCGGCTGGACTATCGGTGAGCGCTTTGATGACCGCTTTGATACCGAGGTGGGCGGAACAC
AGCTTATCGTCACCCAGTCACTCAACGAAAAAGGTGAGATTGAAGGAGGACTTCCTACCT
CGATGTCGCTGAATGACTTTGCTGAGTTCCCTAAACAGCCAAGGCCTCCTCAAATAGTCG
ATTACCAGGAAGACAAGCGATACACGCTTGAGGAAGCGGAAGCTATCCCTGAGCTCGCAC
CAGTGGTTCAGCGTCTGAAAGAGCGCATCGAAGAGTATGAGGAGAGAAGAGCCCATGATT
GACATGACATGCCGCCTCTGTGCTGGTTCCGGTGTTTATGACTTTTCCCCTTCCAAAGAA
ACCTGCGGTATGTGCCAAGGGCATGGGAAATTTGCTGACGCCAAAGCAATGCTGGTGGCT
GCCATCGAGCTGGCAAAGAAGCAACGCGAGCCGGTCATTGAAGTGGTAATGGGGTTGGTG
CTCCAGGGTGTGGATGTTAGAGGGAAGGAGCCTGGAGAGCGGTTCAAGGCTGCTCAGGCC
TTTAACGCCCCAGGGCACAACCTATTACGATACGGGTGGGATGCTTGGGAGCTCTATGCC
CTCCATGAGGGTGTAAGTCCAGAACTTGCATCGCTTGGCCGAAGTGTGATGCGCGAGTGG
CATAGCCATTCATGGGGCCGGTTCAGTGGAGAGGTAGGCTTTAACGCCGCCGAGATCATG
ATCAAACAGGCCAAAGACAACCCAGAAAAAGCAGAAGAGCGCTGGGCGTTCCTGCTTAGT
GAAGAATGGATGGTGGAGTAATTGTCGTGACAGAACAAACCAATAAGAAATTGATGCTGG
ATGAGTCTCCAGATATGCCCCTGCAATTCAAGTGGAGAAAGTTTGGTGGTGCGATTTTTA
CTGCCACGCAAACAATGGAGCACGTCAAGGAAGGACGTAAGCTCGGGCCGACTCCAGCAG
GGATGCTACCTCAAGAAGTGTGGCTGAACCGCCTGATCGCTATGGAAGAAACCCTGCCAC
GAGGGAAGTTTTTTGACAGATTCCGCCGCCGCGACAAAAACGAGCAGTATGACTTGGCCG
CCGATCATCTCCGTCTTGTCGGACAGAAAAAGCGATAGGGGGATAGATGAAAGCCCAGGC
ATACCCACCATCAGTCATTCGTAAAGGCGCTGTGTTGTACGCAGCCCTTTATTACATCTC
TGATGATGATAAAGCAAAGGTCGAGGTTACAGAGTGGATAGTTCGCTCTATTCAGAAACG
ACGCAACTCAACCAGCGATCAACGCTATGTCAATCTTGCCCAAAAGTTAGATGGGATTAC
GTGGGGGAAAAGGTCACGGAAGAATGGGGACTTTGGTTGGTTGCCATCTATACCGAGCTG
GTGTTTGAAGCAGTTCCGGGAAGGTGGCGAATTGCCTTTTGGCGTTTATACGACGCGACT
GGCCGCTCTCAAGTTTGCGAAAGTCAGCTTGCAGGAAGAGGTCCAATATTGCGAGGCCGA
GCTGAAAAAGCCTCAGACAGAAGAGGATACTCAGGAACTCCAAGAGGAGCTGGCGGAGAA
CCAGAGACTTCTGAAAGCTGCTGGAGCAATGGTGAAGCGCGAGCAAAACAAGAAGAAAAG
AGGTTGACCAATGTTACCGATCGTCTCCCCCTCCGTAGTAACCAAACAACTTGCGTTCAA
TCGAGTAGGCGATAAACGCAAGGTTAGGGTTTCGTCCAACTTTTTGGATGTTATGGGGTT
CAAGCCAGGTATGGGCATCGCCGTTGAGCCAGGGGAGGGGATGGGCGGTTTTTCGGTGAT
CCCAGCGACCGATGAACTACAGACACACCAGGTTTATCAGCGCCGGTATCAACCAAAGAG
TCGCTCCAACAACCCGCTGGAAACCGTTATTGAGTTTTCTGGACAAGGGCTCATAGATAA
GTGCTTCCCTCGCTATACAGAGCGTTTCCACGTCGAAATGCGAAAAGGCCGAGTAGTCTT
CACTCCTGTCGCAAACAGAGCCTTTGCCATTGCGGATCGGTTCAGAAAAACCAGCCCTTT
CCGTGCCTTTGTGGCATTGACTGGCGGGGTAGACATTCATGTTATGGAAGCGCTTGGCTG
GAAGGCTGAGATTGTTTTGGAGCATCGTCCAGTTGAAGCCAGAGACAGAGCATCCGGGCG
GAACCTGAGTGAAGTACATGCGCTCAATACGCTGGTGAACAGCTCCCCGCGTATCCTGCT
GAATGAAGACATTCATCACCTGGAGCTGGATCGCCTTGGAGCCTTGCTGGCGGAGTGCCC
ACCAATTGGTTTGGCCCATTACTCGTTGGGGTGTGATGACCACTCAAACGCCAAAAGTCC
AAGGGACAAAGAGCGTTCTCTTGAGGATCTCTCCACCATGCTCGACATGGTTTACCCGGC
ACTAAAACAGATCGAGGTCGTGAACCCTGCCGTCGTGCTGGTGGAAAACGTCCCGAACTT
CAAAGCATCCGGAGCCGGGGCGATGATGGGAACGACACTGCGGCGGATGGGGTACTTCCT
CACTGAGATGGTCTTGAACGGTCTGGATTTCGGCGCTTACCAGGGGCGAGAACGCTATTA
CATGGTGGCGTCGGTCTTCCCTGGGTTCGTTCCACCGAAACCTGAGCAGAGAGCTGGTGG
ACGATTGTGGCCGGTGATCGAGAAGCACCTCGGGGATTGTGCTGACGTCACAGTGTTGAA
GTCAATTCAGGCCAGAGAGTCAACTTCTCGCAGGATGCCTGCGTTCTTGACGAGAGAAAG
CACCAGTTGCCCGACCATCCTCAAGTCTCAGGATCGTGGGGTAAAGGATGCAGTGTACAT
CCAAGACGGTGGCCGCATTTACAAGCCATCGGTCGATCTGGTTCAGGAGTTGATGTCGAT
ACCTGATAGCTTTGATGTTTCGTGGATGGCAAAAGAACAAGCGACAGAAACGCTTGGGCA
GAGTGTGGATTACAGATTGCATTCAGCGGTCATGGCCGCAGTTCGGGATCACCTGAATGT

GAATTGCGGTCGCCATACCGTGGTGCAGCACGGTATCAGAAGTAAGGAAGGTAGATAATG
GCAGTCATTTACTACGGCGAGGGAACCCATGACGCCGGTTTCGTCGGGTTCCGTGTCGCA
CGAACAGTTGGGGTGGCGGATGATTACCGGCAGGAATACTTCTCCTTGAGAGAGTATTCC
TACGCAACAGCTCACCGGCTGGCCTACAGCTTGGACCGAAAGTGGGAAGCTGAGGCAGAA
GAGGTGAAGCGTCAGAATAAGACTTGTAAGCGGCGACGCAACTCCGGGCCAAACATCATT
GCTGAGGGGCTGAGGGCTTATATCAGCATTGAGAACCGGAGCCGGATGGGGGTGAAGAGA
ACCTACTTCGCGCCTTGTTTTCTCGTCACAAAGCCAGGCTACGGCAATGGGGATATTGCT
TTCAGGATTTCCACTCATGGCTACGCAGAAGCCTACGAAAAGGCGGTGGAAAAATATTGT
GAGATCCATGATTTGACGGATGAGCAGTATGTTGAGTTGCTTGACCGTATGCCGAGTACA
GAGGTGTTCACTGGATACCTGCTGAATGCTCTTTTAATGCGCGGCCATCGCGCTACAAAA
GCTGAAATACTGAGTAAGCTGGGGGCTGAGAAGAATGAAGAGGACATTGCCAATGGCAAA
GGGAAAAGCGGCCAGAACAGAGTGCGTTGCCCAGAGTATCGGTGGGCGCAATAACAGCGC
CACCAGCTTTTGGGACCGTATTTTAACAGTTGAGGAGACGGCCACACTCATTGGCGTCAG
GATCTCTGAGCTCAAAGACGCGGTGCGCTATCAGAAACTCCTGAAAGGGAAGGCAGCTCC
AGAAGTACATTCGGTATCAGGAACTGGGAAACTGTTTTTTGATGGGCGATCCATTAAATC
GTTCATAGAGGACTAACCGGTGGATCTGGATCAAAAGCAAGAACCGTGGATCAGCGTCAA
CGACAAGATGCCGGTCGTCGGTGTGCCTGTTCATTGCCAGCTTAAAGGATGTTGGTCGGG
CAAAATTGTTGAGTACGACTTGATCCATGTTCAGGAAGATGATTGTTCGTGGCGAACGGC
GGACGACAACTCGGAAGTCAGCTATGACTTCGATGTCATCACCTGGAGACCTATTTAGGT
TTGGGAGTCTTGCATTATCCGGCGGTTATTGCCGTGAAAATACGGATGTTCAAACCATGT
TTTATAGCAACACCTCCTTGGGGGCGGAATTATGTTTTTTGATAACAAAGTAGAATCTCA
CTCGCTCGTAATGGGAGCCAGCGGGAAGGGTAAATCGGTCTTGTCCGAACAAGTGCGGAA
AAATGCGAGGCTGCGCGGTGATCTGCTGGTGGATACTGAAATGTACCGTGAAGGTCGAGG
GTTGAAACCATACGAGCATGAGTACGCTCGTCGCTTAGTTCTGGGGCTAAGTGGACCGTT
GCCGCGTGAACTGCGTGGGAAGCCGGTGACGGTTATCTCTGATGTGTCCAGGCCAAAAAA
GGTAAAGCGTCAGCCAAAGCAATTTGTTAAGACCGTGAATGGCGTAACCCTTGAACGCCA
ATTGGTGGCCGATGCAAGAGATCAGTTGGAAATGCAAACAGGCGTCTGGCTCAAACAACC
ACAGCTCATCGAGCTGATGGAAGAGTCGGGCATAGACGAAACTCTGGCCGATTTTGGTGA
GGCTGAAACACAGATCCGAGAAATGCTGGCGGATGCGTTGGCGATGAAACTGGTAGGACG
CTCATGGCCCAAGTGTGGAGCCCTCTATAACGCGGCAGAAAAGTCCGACGTTAACTTCTC
CTCCGAGCTTGATGCTGCGGCCAAAAAAGCTGGATACATGGTTCGCTAAATGGGATTTGG
TGTGGACAAGATAGATCGGCAAAGTTGGCTCGTTAAATTCAGGCGAGCGAAGTGTCAGGA
CACTTTAGACACGATGAGGGATGCGGCCATTCGCAACTATGAAGGGAATATTCGTGTTAT
CGCGGATATTGTATTGGCCCATGAGGCGAGAGAGACAGAAATTGAAAAAGGGATGTTTTG
TCGAATAGTTAGATAGTCTGATGGGCTATCAGGTGGTGATTATGAAAAAGAGAATACTTC
ATCTGCCCGTAAAAAAGATTTACTTCGATCAGATCAAATCTGGAGAAAAACCAGATGAAT
ATCGCCTCGTTACAGACTACTGGATAAAAAGACTAGAGGGGCGCGAGTATGATGAAGTCC
ATGTTAAGTGTGGCTACCCAAAGGCTGGAGATATGTCCAGGATAGAGATTCGCCCTTGGC
GTGGCTTCTCAAGGAGTGTTATAACGCACCCGCACTTTGGAGATTGTCCGGTTGAAGTAT
TCGCTATCCATGTGAATTGAATGGTGTATGACAACTGTGCTTGGTAGGAAAATCAGGTAT
CAAAAGAAATTGGGTTATTAAAAATTAGGGGCCATCGGCCCCTTAATTGTTTTAGTCTGT
GAACATTGAACAATCGAGGTGGGATAGTGCTTGTTCGTAGCTGTCTTTGTTGTTCGTCTT
ACGTAGTTCCACCAGCTCAACAATCACTGGACTTAAAGCCAGAGATTGAGGCTCCCAGAC
GCGCTTGCATTTGACCGCCATGTTGGCATTTACGTCTGTAACAACGTAGAGAAACTCAGG
CGCTTCTTTCAATGTCCCCAGGCCCTTTGCCATATCCACAGCCTCAGAGTTCTTATAGCT
TCGAGTAAAGCTCAGCTCGTCTTGGGGAATGTAGCTGGCAGTCGCCGCAGCCAAACCTGA
CACCATGCCTTTACCTGCGCTTTTTGCAATGGCCGAGCTGGTAGCTAAAGTTGGCATTAC
GGCCTGCCCCATTTGGCTGGCGGATGCCGCCGCCAAGCCTGAGGCGATGTTCTTCCCTGC
ATCTTCGGCAGTGGCTGAGCTGGTGGTGAGCATCGCAGTTAAAATGATTGTTCCAAACAT
ACTTAATTCCTCTATCAATCCATAAAGTCCGACTTTTTGAAGGGCATAGTGCCTTTGCAA
TCGGGATAGGCGGTGCAGCCCCAGAACTTAGAAGCTCGCTTCTTGCCGGGCGCTTTTCCT
TTCCTGAGACGCATCGGGCTACCGCAATCTGGGCAGTCTGGACAATCCTCAGCCGCGATA
CGCTTCTCGGGTTTCCCTCGGTTGTCTGGGAACGTCTTCTTGCAAGCCTCGTTCTGGCAG
CCCCAGAAAAATCCGTTCTTCCCTTTGATCCGGTGCATCTCTCCACCGCAGTTGAAGCAT

TCGTGTGAAGGCGGCTTAGCGCCCTCGAAGGCTTTAGCCATCGCGCCACCTTCTTTGGTC
AGAACCGGTGCGGCCACTTTGAGCTGCTCAACCATTTGGCAGATCCAGGTGGAAATCTGC
TTCATGAAGACAGACATATTCCCGGAGCCGGAGGCGACTTTCTCAAGCTCCTGCTCCCAA
GCCGCAGTCATGCCGGGTGATTTGATGGCTGGTGGAAGCACTGCGATAAGCGCATGAGCC
TTATCAGTTGCAAGCAGTACCTTTTTCTGACGTTTGAAGTAGCCTTTATCAACAGCGCCC
TGGATGATGCTTGCGCGTGTTGCTGGAGTGCCTAAACCGGCTGTGTCTTTGAGAATTTGC
TTGAACTTCTCCTCAGTCACGAACCGGGCAATGTTCTCCATCGCGGCAAGCAATGTGGCT
TCGGTGAAGTGCGGTGCTGGCCGCGTCATTTTGTTTGCCAGCTCGGCACCATTAAGCAAA
GCGGGTTCGCCCTGGCTAACCCTGGGGAGCTTCTCTTGTTCAACCGGCGCGTCGGTGTCT
TCCCCCTCGTCCTTGGGACTGCTTTCACTATCAGAAGCAAACAAAACCTTCCAGCCTTGT
TTGGCAGGTGTCTTACCAGAGGCCGCAAAGAGGTGACGCCCACACTGCACCTCGATGGAG
GTTTTGGTGAACTCGAACTCGCTGTAGAACTGCGCGATGTAGAAACGACGGATGGCGTCG
TAAAGATTGAACTCGATCTCAGACATGGCACTGATGTCTGTTCTGGCCGGTGTTGGAATG
ATCGCGTGGTGCGCGGTCACTTTGGCGTCATTGAATACCCTGGCCTTGCGATGAGGATCT
GCGCCAGCCACCAGCCCAGAGACGTTCTGATCGGACAAAATGAGCGCCTGGAGAATGTCC
GGAATGTCTTCCTTTTGGCTCTCGGGTAGATAACGACTATCAGTACGAGGGTAGGTGGTC
GCTTTGTGCGTCTCGTACAGAGCTTGAGCTGCATCCAGTACCTGCTGAGCGGTGTATCCC
CATCGTTTGCTTGCGTATTGTTGCAGCGACGTTAGATCGAAGGGGAGGGGGGCTGACTCT
TTACCTGGTTTGGTTTCTGCTTTGCTGATAACAGCATTGGCACCATTGACCTGGGAGGCT
ACCTGCTCGGCATAGGCCTTATTGACGCACCGGCCTTGCTCGTCACTGCACTCTTCTGGT
GGTATCCATTGGGCGGCAAACTGTCCATTCTGTACGGACACGTTCACACCCAGAGTCCAG
TATGGTGAGGGGGTGAAACCGGCGATCTCTCGGTCCCGTTGACAAACAAGAGCGACGGTT
GGGGTGATAACCCTGCCAACGTGAAGAGTGTGGTTGAAGCCGACATCTCGGGCCAGCACT
GTGTAGAGGCGGCTCACGTTCATGCCTACCAACCAGTCAGCGCGTTGCCGTGCCAATGCG
GCGTAGTAGAGTGAGACCGTATCCTTGCCATCCTTTACGTTGTTCAGCGCTTTCTTGATG
CTTGACTCATCGAGAGCCGTCAAACAAACCCGGCGAATAGGGCCAGAGTAGCGGAATCGA
TCCAGAAGCGAACGGGCGATAGCCTCACCTTCTCGGTCGTAGTCCGTTGAGATGTAGATG
GTGCTCGCCTTTTTGACCAGTCCCTCAACAATTTTGTACTGTTTGAACGCACTCTTGCGA
ACGTTGTACCGCCATGACTCTGGTGCAATAGGGAGGGTCTCCAGTGACCATGACTTGTAG
CGTTCGTCATAATCGTCTGGCATATACAATTCCAGCAGGTGGCCGAACGCCCACGTAATA
ACGCGGTTTCCTCCATCATGGAGAAATCCATCACCTCGTTGGGAGGCCTTCATTACGCCA
GCCAAATCTTTGGCCTGAGAAGGCTTCTCGCAGATATAAAGGTCCATTAAGCAGCTCCTC
TCATCTTTGATTCCAGATTCGCCAGCTCAGCCCAGCTTTGGGCCAGACCTTTGACTTCGG
GGTAGTCTCTGGAGAGGTTTTCGAGGACTCCAGAAATGACATAGGCCGCTTCGCGGGAAT
TACCCCTCGAACCGGCCTCCTGGAGCATGATTGATAAAGCGTCGAAGAGCTCAGCCTTAC
GGCTTGGCTCTGTCTGGTTTCTTATTTCAGAGCTTTGCATGGGTTCACCACTGAATAGGG
ACGCACGGACTCGCGCACAATGCGGCGATCTGCGTCAGTTACAGCCTGGCTGGTGCAAAA
GTGCGTCCAGGCATTCAGATAATCTTGATAGGTGTGGGTAGGTGGTATGCAGCCGGTTAC
AACATCCTTGGGGATGTTTGTAGTCCCCTCGGCTTCCACAAAGCGCCAATCGTGCTCGTC
CATACCTTTGCCGCCATCGCAATAGCGTTCCCAGCGTGACAACTCGTACAGCGAGTAGCC
TTTGCCTTTATTCATGGACTTGAAGGTATTGATGGCTTCGTTCACATCCCCGTGCTGCTT
GATCCCTGCTGGAGTTTTATCTGAACCAGCGGAGTCATCTTTGACCTTGTTCATCGTTGC
GCTGACATTACCGTCGCCCAGCACATGGATATAACCAGGTTGACGTAATGCAGCACCACC
GCGCCCCATGTTGTCCCATGCCATCGACAGTTCCCGGCTAACTCGGGCAGGTTTGTCATT
TTCGGCAGGGGCAGTCTGGGGCTGAGGCTTTTGCTCGGGGGCGTTAGAGGCACACCCGGC
CAGAATTGCCACTCCCAGAATTGCTATCAGTTGTCTCATCGTGTCGTTCCTCATCAAAAA
TTGTCGATGAGCCCATTCTAAAAGGGGGGGGATGGAAAGCTGGTTTCTCAAACTGCCCAA
ATTGGACAGATAGGCGTGTGTGCGATCTAAAGGTGTTTTGAGAGCATGTAGCGACGAGAA
ATACGATAGGAATATGGTCAACCTACATGCTGAAAGCCCTTAACAAGTTATTTGGTGGGC
GAAGTGGAGTGATCGAGACCGCGCCGAGCGCCAGAGTGTTGCCGCTTAAAGACGTGGAAG
ATGAAGAAATCCCTCGATACCCACCTTTTGCCAAGGGCCTGCCAGTGGCCCCACTAGACA
AGATACTGGCAACCCAAGCTGAACTGATTGAGAAAGTGCGGAACTCTCTCGGTTTCACTG
TGGACGACTTCAACCGGCTTGTTTTGCCGGTGATCCAGCGGTATGCCGCGTTTGTTCACC
TGTTGCCAGCTTCCGAATCACACCACCACCGTGGCGCTGGTGGTCTGTTCCGACATGGGC

TTGAAGTGGCCTTCTGGGCAGCTCAGGCATCTGAGTCAGTTATCTTTTCCATCGAGGGGA
CGCCTCGGGAACGCCGTGACAATGAGCCGCGTTGGAGACTGGCGAGCTGTTTCTCTGGGC
TGCTGCATGATGTGGGTAAACCGCTCTCGGATGTGTCCATTACGGACAAAGACGGGTCAA
TCACATGGAACCCGTATTCGGAGTCACTTCATGACTGGGCACACCGTCACGAAATCGACC
GTTACTTTATCCGGTGGCGCGACAAGCGACACAAAAGACATGAGCAATTCTCGCTGCTGG
CGGTGGATCGAATTATTCCGGCTGAGACTCGGGAGTTTCTGTCCAAGTCTGGCCCGTCCA
TCATGGAAGCGATGCTGGAAGCTATCTCAGGAACCAGCGTCAATCAGCCTGTGACCAAGC
TGATGCTTCGCGCTGACCAAGAGAGCGTCTCACGGGACCTTCGCCAGAGTCGTCTCGATG
TGGACGAGTTCTCCTATGGTGTGCCCGTCGAGCGTTACGTGTTCGATGCCATCCGCCGCC
TGGTTAAAACCGGAAAATGGAAGGTCAATGAGCCAGGCGCGAAAGTCTGGCACCTCAACC
AAGGTGTATTCATTGCCTGGAAACAGCTTGGGGACCTTTATGACTTGATCAGCCACGACA
AGATCCCCGGTATCCCACGAGACCCTGACACACTGGCCGACATTCTCATCGAACGTGGTT
TTGCTGTACCAAACACGGTGCAGGAGAAGGGTGAACGTGCGTACTACCGCTACTGGGAAG
TTTTGCCTGAGATGCTCCAGGAGGCGGCGGGTTCGGTGAAGATCTTGATGCTCCGACTCG
AATCAAACGACCTGGTGTTTACGACTGAGCCTCCTGCGGCTGTTGCTGCGGAAGTTGTTG
GTGATGTTGAGGACGCTGAGATTGAGTTCGTTGATCCTGAGGAAGCCGATGACGGTGACG
ACCAAGAGGAAGGTGAAGCAGCTCTGAACGATGACATGTTGGCCGCAGAGCAGGAAGCAG
AGAAAGCTCTAGCTGGCCTTGGCTTTGGTGATGCGATGGAGATGCTGAAAAGCACCTCCG
ATGCTGTCGAGGAGAAGCCAGAGCAAAAAGATGCGGGACCAACGGAATCATCTAAGCCTG
ACGCTGGCAAGAAGGGTAAGCCGCAGAGCAAACCGGGCAAAGCAAAACCGAAGAGTGATA
CGGAGAAACAACCCCATAAACCAGAGGCAAAAGAGGATCTGTCCCCTCAGGACATTGCCA
AAAACGCACCACCTTTGGCAAACGACAATCCGTTACAAGCACTCAAGGATGTTGGGGGTG
GACTGGGGGACATCGACTTCCCGTTTGACGCATTCAACGCATCGGCAGAGACAACCAGCA
CTGACGCAACAAACTCAGAAATCCCAGATGTGGCAATGCCCGGAAAGCAAGAGGAGCAGC
CAAAACAGGACTTCGTTCCACAAGAACAAAACTCCCTGCAGGGCGATGACTTTCCAATGT
TCGGTGGTTCTGATGAACCGCCATCATGGGCGATTGAGCCGCTCCCTATGCTGACTGACG
CACCAGAACAACCAACGCACACGCCAGAAATGCCGCATACGGACAACGTTAATCAGCATG
AGAAGGACGCAAAGACCTTGCTCGTTGAGATGTTGTCTGGATACGGGGAAGCATCGGCGT
TGCTTGAACAAGCGATCATGCCTGTTTTGGAAGGTAAAACGACGCTGGGCGAAGTCCTAT
GCCTGATGAAGGGGCAAGCCGTCATTTTGTACCCGGATGGCGCTCGGTCGCTGGGTGCGC
CGTCAGAGGTTCTCTCGAAGCTGTCCCACGCCAACGCGATTGTTCCGGACCCGATTATGC
CAGGTCGCAAAGTTCGTGATTTCAGCGGAGTGAAGGCAATTGTACTGGCGGAGCAGCTTT
CAGATGCAGTCGTGGCGGCCATTAAGGATGCCGAGGCGTCAATGGGTGGATACCAGGATG
CCTTTGAGCTCGTCTCTCCCCCTGGCTTGGATGCAAGCAAAAATAAGTCTGCACCGAAAC
AACAAAGCCGAAAAAAGGCGCAGCAGCAGAAGCCTGAGGTTAATGCCGGTAAAGCCTCGC
CTGAACAAAAGGCGAAAGGTAAGGACTCCCAGCCACAGCCGAAGGAGAAGAAGGTCGATG
TTACTTCTCCGGTTGAAGAGCAACAGCGCAAGCCGGTCCAAGAAAAACAGAACGTGGCTC
GCCTTCCTAAGCGGGAGGCTCAACCGGTGGCTCCTGAGCCCAAAGTTGAGCGTGAGAAGG
AATTGGGACACGTCGAGGTGCGAGAAAGGGAAGATCCAGAGGTTAGGGAGTTTGAGCCGC
CTAAGGCGAAAACAAACCCGAAAGACATCAACGCGGAAGATTTCTTGCCGTCTGGTGTTA
CGCCTCAGAAAGCACTCCAGATGCTCAAGGACATGATCCAAAAACGCTCGGGCCGATGGC
TCGTGACACCTGTCCTGGAAGAGGATGGCTGCTTAGTAACCAGTGACAAAGCCTTCGACA
TGATTGCCGGTGAAAACATCGGCATCAGCAAACACATCCTCTGCGGGATGCTGAGCCGGG
CACAGAGACGCCCTTTGCTCAAGAAACGTCAGGGAAAATTGTATTTAGAGGTAAATGAAA
CATGACAATGAGTTATGACCCGCTCGCCTACGAGATGCCGTGGCGGCCCAACTATGAAAA
AAATGCTGTAGCAGGCTGGCTTGCCGCCTCCGGCGCGGCTTTGGCCGTAGAGCAAGTCAG
CACGATGCCGCCGGAGCCATTCTATTGGATGACGGGGATCTGTGGCGTGATGGCGATGGC
TCGTTTGCCCAAGGCTATCAAACTTCACCTGCTCCAAAAGCATTTGAAGGGGCGTGATCT
GGAGTTTATTTCCATTGCGGAGCTCCAAAAGTACATCAAGGACACGCCGGACGATATGTG
GCTTGGTAGTGGGTTCCTGTGGGAAAACCGCCATGCCCAGCGCGTGTTTGAGATCCTGAA
ACGCGACTGGACTTCCATCGTAGGGAGAGAGTCCACGGTCAAAAAGGTTGTCCGGAAGAT
ACAGGGTAAGAAAAAGGAGCTGCCAATCGGCCAGCCCTGGATTCACGGGGTAGAGCCCAA
AGAAGAGAAGCTGATGCAGCCACTCAAGCACACTGAGGGGCATTCGCTGATCGTTGGGAC
CACCGGCTCGGGCAAGACCCGTATGTTCGACATCCTGATTTCACAGGCCATTCTGCGTGG

GGAAGCCGTGATCATCATAGACCCGAAAGGGGATAAGGAGATGCGGGACAATGCCCGACG
TGCCTGTGAAGCTATGGGGCAGCCGGAAAGATTCGTCTCATTTCATCCAGCATTCCCGGA
AGAGTCGGTGCGTATCGACCCTCTGCGTAACTTCACCCGCGTGACTGAAATCGCAAGTCG
TTTGGCAGCGTTGATCCCGTCCGAAGCAGGGGCCGACCCGTTCAAATCATTTGGATGGCA
GGCACTGAACAACATCGCTCAGGGCTTGGTCATCACTCATGATCGTCCCAACCTGACAAA
GCTCCGCCGATTCCTTGAAGGTGGCGCTGCTGGCTTGGTCATCAAGGCCGTTCAGGCTTA
CTCAGAGCGAGTTATGCCCGACTGGGAGGCAGAAGCAGCGGCTTACTTGGAAAAAGCCAA
AAACGGTTCGCGTGAGAAGATCGCTTTCGCGTTGATGAAGTTCTACTACGACATCATCCA
ACCTGAGCACCCGAACTCTGACCTGGAAGGCTTGCTGTCGATGTTCCAGCACGACCAAAC
CCACTTCTCCAAGATGGTGGCGAACCTCCTGCCGATCATGAATATGCTGACGTCCGGGGA
GCTAGGCCCTCTGCTGTCTCCAGACTCATCTGATCTGAGCGACGAACGCCAGATCACCGA
TTCCGCAAAAATCATCAACAACGCTCAAGTTGCTTATCTGGGGCTCGACTCCCTGACCGA
CAACATGGTTGGTAGTGCTATGGGGTCCATCTTCCTGTCAGACCTGACAGCGGTTGCCGG
TGACAGATACAACTACGGCGTCAACAACAGACCCGTAAATATCTTTGTTGATGAGGCTGC
CGAGGTGATCAACGACCCGTTCATCCAGCTCCTGAACAAAGGTCGCGGTGCGAAACTTCG
TCTTTTCGTTGCAACTCAGACTTTTGCTGACTTCGCAGCTCGACTGGGTAGCAAAGACAA
AGCGCTCCAGGTGTTGGGGAACATCAACAACACGTTTGCTCTGCGTATCGTCGATGGTGA
AACCCAGGAGTATATCGCGGATAACCTGCCGAAGACCCGGCTCAAGTACGTCATGCGGAC
TCAAGGCCAGAACTCGGATGGCAAGGAGCCCATTATGCACGGAGGCAACCAAGGCGAGCG
TTTGATGGAGGAGGAAGCTGATCTGTTCCCAGCCCAGTTATTGGGAATGCTTCCGAACCT
GGAATACATAGCCAAAATTTCAGGCGGAACAATCGTAAAAGGCCGTCTGCCCATATTGAC
CCAGTAAGAGCAGAGCTATGTGTGACAAGAAGTATAGAGATTACGAGGTAGCCATCATGG
TCGATGTGAACCCTTTCGACAGGGTTATGAATGAATTGAAAAGTCGTGGCCGCAAGAACG
CTCACATCCTGAGCATCCTCCAATTCGACTGGCCTGCATCGGAGGCCATCATCGAGAAGC
TGAGCTGCTACATCACAGACGGGATTAAGGCTAATCAGGAGCCTGTGATTTACCCGATCA
TTGAAGAAGCTCTGCATCGCTACAGCCAGCTCGTGTTTCATGAGCAGAGAGAGAAATATG
AAGACCCGGCCAGAATTGGGGCATTTCTGGAAACCCTGATCACCGAAACCTGCCGGGCGT
TGGAAGTGCAAATTGTCGATAGTGGCGGTGATTCATGGTCTGTCGATTCAGGAGAGTCGT
TCTCACTGTGGCTTTCTTCCCATCCAGGAGAACTATCCATTAACCCGCAGCCCCATGAGG
ATGAGACCTCTTTGCGTGGCTTGCTGTATGAGCTCATCACCTGTGAGAGCGTGAAAACTG
TTTTAAGGAGAACCGACTATGAAGAAGCCGTGGTTGCTGGTCGCATGGCTGCTGGTTATT
GAGTTGCTGGCAATATTGCTGCTGATCCCTGGCGACTGGACAGACAGAGCCATCAAAAGG
GAATCCGTGCTGGTGGAACAGAGTCTTGGTGTCGAAGCAAGAGACTGGATACAGAACAAA
GCATCTACCTGGTTCAGGTCGAGCGTTATTGATTCAGGCTTCTATGAGGGGATGTACCAA
ACGCTGATCCCATCAGAAGAGGAGCGCCAGAAGTCCAAGGGGATGCAGGATATGGGCAAG
GGCTGGTTTGTGTGGGTCAAAGGCCGCATGGAAGCCTTTGTCAACGTCATTTACCAGTTC
TACACACGGTTGGCGCTGTTAGCCGCGTGGGCTCCCTATATGCTGATCCTGTTCGTACCT
GCGGTATATGACGGGATGATGACATGGCGAATTAAGCGGACCAACTTCGATTATGCGAGT
CCGGTTCTCCATCGTTATAGCGTTCGCGGAACGATGTACCTGATGGCCGGATTGTTCATC
GCGTTCTTCATCCCCATAGCGCTCGATCCGGTTGTCATCCCGATGACAATGATGACGTGC
TGTGTCCTGGTTGGCCTGACGTTCGGCAACCTCCAGAAACGGGTATAGGGAGGGAGGATG
AGCTACTCCGTTATAAATCAAGATGGGGTGCATCTGTGCGACATCCCATTGAACGTCTAC
CAGGTGATACGTCGCCAATCCTTGTCTGCGTTGTGGCTTTACTGGGCGCAGAGCTTGAAT
TTGGTGAAGGTGCTCATTGCTTGCGTCGGAAAGATGATCTTTGTGATGCCGGTAATGTGG
TTCTGGGTGCTGGTGGTGTGCCGCTTGGTTGAACCAGAACGTATCTCAGACGTGCCAGGA
CTTATCTCTCCGGACGTGATCGGGTGGGCCGCTGGTGCGGCCATCACCCTCATTATCTAT
TCCGTTTTTACACGCCCAGCTTTGTACGGATACCACAACTTTTTCAAGCAGCATATTTGC
TCAAGAGTAAAGCAAGTAACGCCGGAACTCAGAACAGTCACTGGGCCACTGTTCTTTTAC
CGAAACGAAATTGGAACGATATAGAGAGGAACCATGAAAGTAACCAATAAACTCAAACTG
CTGGCACTGGGGCTGTGTATGGCCGGTACTGCCATCGCGGCTCCTGATGTGCTCAATGAC
GACGCAAAGCTCAAGAACCTTGAGAAGGTGTGTCCCGACTGTAAGATGGTGGCAAAAGAT
GTACTCAATCTGCGGGTTGAAAACTGCCAGCTCAAAGACACTTCCAGTGCAATGATGATT
GGCACCATGCAAAATGACCCTATGTTTTCGTTTATGCTGGCAGTACACACAGCGGCAGGC
TCGGAGGCATACAAAACGGTAGTTGGTGCCGCTGGCAACCATGTTGACTGTGAAAACCCG

TTGAACTGGATCAAGCTGACCCAGCAAGCGATTAAAGGAGGCAAAGTCTAATGCGTAAGA
GAGATTTCTTTTTTGGAGAGGTGTATGAGGGGGGTGCAGGAGCCACTCTACGACTGAGTG
ATATGGAACCATTGGCAAGAAAAGTGTCGGCAGAGTTCTTCACTGCACAACTGAACCGTA
TGCTGAAAGAGCATGACGGTCAGTTGACGCTCAGCGATGGAACGTCGTACCCCAGCTTTT
GGAGCTTCATCGACAAGGTTGTTCCAGAGCAGGTTGGTTTCGTGGAGATCTACGCTCGGC
AGGATGTTAACGATAATGTTGAAGCGACACTGGCGTGTGACATCGTTTTGGTAAATGGTG
TGATCACCGTTAAACCTCACTGGTGTGCTTACAAAGACATCAGGGCTGACGAAGTGATTT
CCACCTTACTGGTGCCTTTGCATTTGAAGGCTCTCCAGGGTAAGGCTTACATTCGCTGGG
ATGATGGTGAAACCGAACCTCTGTTACAAAACGACGACTATCAGGCTGAACTTGAAAATG
TGTTTAGTGTTTCCAAGTACCCATCAGCCATGAGCTGGGGCGATACAGCAGACCAGAAGG
TTAAGCAGTACAAGATGGACCTTGAGTGCGCCACAGATGTTGGTTGTCGAGGTGTCTCAT
CAGAGCAAGCATGGGATGCTTATCGAGAACTCCGTTACAATAGAACAGTGTGAAACAAGC
GGCCATTGGCCGCTTTGAACTTTCACATGTGAAAGTTTTTCGCCTCCCGCTCTTATCATC
TCCTCGAATAAACCTCCATAAAGTGCCCAAAATGGGCCGTAACAGCACTGTTCCTAGATT
GATCAATTAAGAGACGATTACTCCGTGATGACGACAAGTTATTTGGAGGGTCTATGTCCG
CACAAGCTCACGTCAGAGAAAGCGAAAGCTCATCTGGTTCATTCATCTCCTGGCAGTTCC
TTGTCTGGCAGGTGATGTTCGTTATAGGTGTTGTTGCAGGGATGAACCTGGAACACGCAT
TCAATTTTCTAGGTTAATGGGAGGAAGTGTGAACCTTAATAAACAATTTTTCTGGTTTTT
GATGAACGCGGTGCTGATGTGCATCTTCATCGTTCCAGTGGCCGTCGCGTTTTGGCTGTC
TGCTTTTGCGGCAGGTTTTGACTGGAGTCAGTGGGTTAAGTTGGCTGCTGATACGGCCAA
CAGAGCTGCAAGTGACCCGGCCAAGGCTCTGGGCACGGTTCAGACCTACTGGGGCATTCT
CAGCTTCTTCCTGCTGGCGGCGTACAGTCTGATGTTCAAGTTTAAAGCCAATGCTAACAA
GGAAGTGAAAACTCTGGGGGTGGCCCGACCAGCCAATGAAGTTAGCGTTGCGGCTTCGGA
AAAACATTCGGAAGCGGTTCCTCAAAATCAATAACCGATCCAGTAAACGCAAAAAAGGCG
GCTTAGGCCGCCTTTTTTTATTTGTCGCTGGAAGTGCCCGAAAAGGGCGCGAAGCCGTGG
CAGAAAATCAGTGACCCTGAATGAGAATACAGGCCACTAGGAAAAGAAGAGGGTTGCTAT
GAAGCCTGTAAAGATACCGCGCCGGGTCGATGAGCCCCCGCATCTGCTGTTGTGGAGCGC
AGATGAGTTGGCCCCGATGCTTTTGGGGCTAACGATAGGGGTCATCATCGGTAAGGCCCT
GATCTGCTTTCTGGGGGGGTTACTTGTAACCAACCTTTATCGCCGATTCAGGGATAACCA
TCCGGATGGATACCTGCTCCACATGATCTACTGGGCCGGGTTCATCATGACCAAGGCCAA
ATCTCTCAAGAATCCGTTTGTCCGGAGGTATTTGCCTTGAACCTGAAAAAGTATCTCAAG
ACCTGGGAAGGGACCCAAACAGAAAATAAGTGGGGACGAATCTTCCAGGGTGGTCTTATT
GCTATCGTTTTCCTGCTGGTGGTCCAAGTATTCAGCAAGGAAACCATCGTCACTATCCAG
CCTTTCACGCTCACGGAAGAAGCCTGGGTGACGAAAAATAACGCCTCTCAGTCCTATAAA
GAGGCTTGGGGTTTCGCTTTTGCTCAGCTCCTTGGCAATGTGACGCCAGGAACCGTTGAC
TTTGTGAAAGAACGGATCACCCCGCTTCTCTCCCCGAGCATCTATCAGGACGTGATTGAT
GCCATCGAAATTCAAGCTCAACAGATCAAGAACGACCGCGTAACCATGCGGTTTGAGCCG
CGTTTTGTTGAGTACGAGCCCAAGAGCGACAAGGTGTTTGTCTACGGATATTCCTACGTC
AAAGGGGCTTCTTCTAACGAAGAGCGTAGCGAACGCTCCTACGAGTTCGCCATCAAGATT
TCAAACTACGCGCCCGTGCTGGACTACATCGACACCTATGTAGGAAAGCCACGCACCAAA
ACTGTTTTGGAGCAACTCCAGCGCAAAGAAGAAAACCGGAGAAAGCATGAAGAACAACGC
TAAACTTTCGCTCCTGGCGCTGTCCTTGGCGCTTGGAACTTCAATGGCCTATGCCTCGGA
TGACATTCCTGTTGTCCCGGCCAGTGTTATGAAAAAGGATGTTCCTGCCCCTGTGACGTC
AGGACAAAGCTCCCATGAGGTTGTGGGCAGCATGAATGAAAACCCCTTACTGACGATGAA
GCCGGGGGTTAACCAGATCATCCCGATAGCCGTTGGTCATCCGAACCGAATCGTCACGCC
TTTCAGCAATCCTGAGATCGTTTCAACATCTCTGACCGGGGCGACGGATAACGGCCAGTG
TGGTGAGGTCTGCATCAAAGAGAATGTGGTCTATGTCGCCACGGATAAGCAGTATCCGGT
GACTATGTTCATTACTGAAAAAGGCTCGGAAGCCCAAGCTCTCAGCCTGACGATGGTTCC
CCGTCGTATTCCGCCCAGAGAAGTCTTTCTCAAGCTCGATGGTGGTGTAGGTATCACTGG
TGCTTTTGCCAATACCAAGGCTGAGACCTGGGAACAGAGCCAGCCTTATGTCGAAACCAT
CCGGTCAGTATTCCGAAAGATTGCTCTTGGTGAAGTCCCTCAAGGTTACACGTTGAACCG
CATCCCTGCTGGTGCTGCGGTGCCGAGCTGCGCTCATCCTGGGGTAAAGGTGGATTTTAG
CAAGGGGCAATACATGATGGGCCACCACCTTAACGTGTTCATCGGTGTCGCCCTGAACGT
CTCTGATCAGCCTATTGAGTTCAAAGAGGCGTTGTGTGGGAGCTGGGATGTGGCTGCGGT

GACTACGTGGCCGCTTAACGTGCTTGAGCCCGGCCAGAAGACGGAGATCTATGTGGCGAA
GAAGCAGAAGCGTGGTCTCGCACCAACGTCTAAGCGTCCATCGCTGCTGGGAGGTGCCCA
ATGATTAAGCGATTTTGGACACAGTTAGACCCCAACAAGAAGCGTTGGGTGTCTATCGCT
GGCGGCGTCTTCGTTCTTTTTGCGGTCGTGACAATGTTCTCTGGTGAACCCAAGAAAGAA
GAGAAGCGCGGTCGCCAAGAAACCATCAAGCACGTTCTCACAGACAAAAACACCCGTGAG
ATCGGGATAGATTCGTTGTCTGCCGATGTGAAGATGGTGTCTCGTGAAAACTCCGACCTG
AAAAAGGAGCTGGAACGAGTCAAGAAAGAGCTGGAGGAAACCAAAACCACTGCCGGGAAA
TCCAGTGATGTTGGCCGTGAGATGACCCGCCTTCGTCAAGATTTGGACCGCCTGACCCAG
AAGAACATGGAATTGGCTAAGAAAGTCGAAACCGGCGCTGCTGGTGGAAAAACATCCTCT
TCATCAGAGGATGCCAGAGCCGATGTTAATGGTGCATCTGGTGGTGATGGTCAGTTCATG
GAGAAAAAGCTCGACTACAAAGATCCAGCATCCTTTTTTCGGGACGCACCGCTTCCTGAC
TCGAAGGGTGGGGCTCCTGCAACTGGCAAGGGAGACGGTCGTGATGCAACTAAACCAGGC
ATCCAAATAGTGAGCTACTCGCAGAAAGCGCCAGAAGTTGAAGAGAAGGACAACAAGGAT
GATGAGTCTATCTACCTACCTTCTGGCTCCATCCTGACAGGGGTGCTCATCAACGGTATG
GACGCACCAACATCTCAAGGTGCTCGTCGAGATCCGTTCCCTTCGACCCTCAGGATTCAG
AAAGAGGCTATTTTGCCTAACCGCTTCCGTGCGGATGTTCGTGAGTGCTTCCTGATTGTT
TCAGGCTATGGAGATCTCAGTTCAGAGCGAGCGTACCTGCGTGGCGAGACCTTCTCGTGC
GTTCGGGATGATGGGGGGGTCATAGAAGCGAAGCTGGATTCCTATGCAGTGGGTGAAGAC
GGTAAGGCCGGTGTCCGTGGTCGCGTCGTATCGAAGCAGGGGCAAATCATCGCCAAGAGC
TTGATGGCAGGCTTCCTTGGTGGCGTTTCCGAAGCCTTTGACGTCAATCCTGTGCCGGTC
GTCAGCACTAACCCTGGCTCAAATACCCAGTACCAGTCTGTGTTCTCCGACCAGATGTTG
CAGGGAGCAGCAGTGAAGGGGGCCAGTAAGGCGCTAGATCGCATCGCTCAGTTCTATATC
GACATGGCCGAAGGCATCTTCCCCGTTATCGAGGTCGATGCTGGCCGTCAGGTAGACATC
ATCGTGACCAAAGGAACCAAGCTACAAATTCGTTCCACCGGGGGAACCAAGAAATGAAAA
ATTTGAACATTTTGACCAGAAAGGGCAGTTCCAGAGGCGAGGCTCAAAAGGAACAGGCAG
TAAGATCCGCAAAGATGTTGGGGGTGGGTGCAGCGCTACTTATTTTGTCGGGCTGTTCGA
CGTTCAACATCGGCAAGGATGAGTATAGCTGTCCGGGAATGCCGAATGGTGTTCAGTGTA
TGTCAGCGCGAGACGTTTACGCCGCAACCAATGACGGAAATGTCCCGCGCCCAATGAAAC
CAGAGGAAGTCGAGGCCAAAGCGGAAGCGGATGGCGAAGGTTCCTCAAACGTTTCAGCGA
ACTCATCTAGCTCCGGAGACCCGGTGATTGACAACTATGTCGCACCGCGTCTTCCGGATC
GCCCGATTCCAATTCGTACACCAGCACAGGTTATGCGGATTTGGGTAGCTCCCTGGGAGG
ACACCAATGGTGATCTCATCGTGACAGGGTATGTCTATACCGAAATCGAACCGCGCAGGT
GGGTGATTGGGGATGGCACACCGCAAAGTGAGCCAGTTTTGAGACCGCTGCAAACGGTAC
AACACGAACCGAAGTCTGAAACAACCAAATAGGAGATGTTCTTGATGAACGCAAATCAGT
TGGCGAATGCCTCAAGTAAAAACAACGCACTCTTCCTCTTCCTGGGGTTGATGGTGGTAG
CCTTCCTGCTCGTGCCGGATCAGGCCCACGCTGGTACTGGTGGTACAGCGTTTGACGACG
TATGGGTAACTCTCAAGGATTGGACCCAAGGTACTTTGGGTCGAATCGTTGCGGGTGCGA
TGATCCTGGTCGGTGTTGTTGGTGGTATCGCTCGCCAGAGCCTCATGGCTTTCGCTATGG
GTATCGGTGGCGGTATGGGCCTGTACAACTCCCCGACCGTAGTGGAATCCATCATGTCTG
CTACTCTGGAACATGCAGAGAAGGTCATCCCGGCTGTTGTGCAACTCAGCAATGGCCTGG
GGGTGTAAAAGACACCTTGCCGTTAATCAACGGGTGATAGGGGCAGCCTAAAAGCTGCCC
TTATTTTTTGGCAAAAAGTCCAGTCATAACAAATTCCAACTATTTCCTTATTTGCTCAAA
AAGGGCACTTCCACAGCCCATGTTTATGCACTCCCTCCCGTAACCTCCAGCATTGGGTTC
TTGTAACTCAAAACGAATGGAGAATGCGATGATCAATTTCAAGCCTAAAATTCCGGCGAT
GCTTGGGGCTCTGGCGGTTCTTACCGCTGGTGCTGCTCATGCAGAGCTGCTGGAATACAC
CTTTAAGGCTCCAGATGGTACGCAACGGTCTCTGACTCCGAATGCCAACTATGCCAACCC
AACGGGCAATATCTCGTTTGCCTTGAGTGCCGGTATCGACCGAAAGGTAAAGATTTCGGT
GATTCGGTCGGATGGAACAGTGGTTTCGACAGCGACCAGCCACCTTCTTGGGGCTACTGA
TCGCATCACAGTGGGTGGAAAATCCTACTATGGTGCGGAACTTCAACTACCGGCTCCTTC
CGAGGGAGTCTACAAACTAAGGGCCGAAATACTGGCGTCAGACGGAAGTTCCGTTCAGAC
TGATGAATACCCGCTTCAAGTCGATACGACAGCGCCAAGTTTGGCAAACGTGACAGTAAA
AGGGGAATGGAACCGGGCGCTTTCTGATGGAACGCTACTGCGAGGTCCTAACCGATTTTC
AGGTATAGATGTGTCTGCATCTGATGCCGGTTCTTCGGTTTCCTCTATACAGGCGTATGC
TGTAGATAGCAAAGGCAAGAGGAGTCCTGTTGTCTCGGTTAATTACGCCAACGGCCAAGG

CCAGTTGTTGAACTGGAGCACTGTGTTCCCTAATGGGGAGGATCTATATACGCTTCATTT
CGAGGCCCTGGATAAAGCAGGGAACAAAGGGTCAATAGCCTATCCTATTGCTTGGGATTC
AGTAGGTGCAAAGTCTGGAGAGAATCCTGAGCCAGTAGCAGTGTATGACCCTAAGAACCC
GCAGGCCTCAACTTTTAAGGTTAATGGTCAGGCCCTATCAGGTTTTGCGCCTTATCAGAA
CGGAATGACCATTTATTCCGATACCTACAGGGTGCTCTACCGGATACCCAAAACAAACTC
CTATCCTTCTTCACCTTACGGTGCTCAAAGCGGTAACTGGTGTGATTATAAAAACTGTAT
TGAAGGCAATATCATTGCCGAAGACGGTACATATGATTATCGGCAGACGCAAGCTGATGT
ATTCCAGCAACATGGGACAAAAACTAAGTCGTTTATTATTTACGACTGGGTTATGAACTC
TCTTGGTAATACATCCGTCAGTGTGAAAGCTGATCCATCGGTTAGCTTGGCTCCCATCGG
TAAGTATGTTGAATATCTTAGGGATGATGGTCAGTGGGTCCGCGGTGAAACCATTAATAT
GAGCTCAGTTAACCATTATAAAAAACTGAGATTTCATGTTGAGCCGAGACCTTACGCTCA
GGAGTTATGGGGATCTTGGTTGCCTACTACTAAAATTCCGGCAAATGCTTCTTATGCAGA
AGTGGATACGGATATATCGTTCAGTGGGCGCTCATGTTCATGGCCTGGATATTGGTCTCG
ACCGGAAGGGAAGCCTGAACTTCCGTCTGATCGGATTGGGGCAACGTTCTGCTATGACCT
TAACCCTCCTGAAATTGCTGGGTTAGAACGAAATGGGCGGATTTTTAAAGCTCATTTCCG
TGAGCCTGATTCATTTGACGGTTGGGGTGTTAACCAGTGGGTTATTTCTAACAACTCAAG
TGCGTCTGCGATAACCTCGACTGGCGAAGAACGTCCGTTGTCTCGTACTGAAGTTCTTCG
TAGTAGCACAAATGATTGGTTTTTCACATTTTCTGCTGAGAATCTGTCAGAGGGCACATA
TACCGGCATATCTCTCGTAGCTAAAGATGCTTTCGGTAATGAGGTTAAGCAGGTATTCAC
TGGCTCCCAATATGCTATGAGCATAGATAATTCAGCGCCGACACTAACCGTATCGATCAG
TGATGGAGCACCAATCCAGTCTTTGGATGATGTTGTAATCACCTTGACTGATACTGCCGA
CCCGAGTCCTAAGCTGACCTCTATTGCCCTCGTCGGAGGCCCAGCCGATGACAAGGTGCA
GTTGTCTTGGCGTGAGGAGTCGAAAGGTCGATTCCGCCTTGAGTACCCGGTAATGTTCCC
GTCTTTGAAGGAAGGGGAATCTTACACGCTGACTGTTTCCGGTGAGGACGCACAAGGCAA
CGCGGTTCAAAAGGCGGTTGGCTTCGAGTACAAACCGCGTCAGGTGATGCTGGCGGATGG
GATGGATGGCAAGGTTATGGTCCCCGCTGTCACTCATGAATTTGTTCATGCAGATGGCAA
GCGGATCATCGAGACCAAGCCGCTGACGCTCAGTGATGGTGCTGTCGTGACAGGTTCATA
CGACGTGTTTGCGACCCTCCGTTCTGATGCGAAAGTGCCGCTGGTGGTGAATGGGGTGCG
TATCGAGCCGGGCCAGACAATGGGGATCATGAGCCAACATGATTTCGGTGCGTCAGGTGG
TCGTTTGAGCATTCCGGTTAAACCGGCTGTTCCTGATGTGGTCGGCTCTTCCAGTCTTCT
TGTCATGACCTCCGCGCCGAACTCACCCATCTTGGTTGTGGACATCAATACCTGGAAAGG
GACGGCCAAGCTCTCGGCTGAATCATGGACAATTCGCCAGGTTATTGACCCGGTGAAAAT
CTATGCCCTGCCAGAGTCGGGTGTGCCTTGCCGGTTCACCACGAAAGAGGATGTGGCTAT
GGCCGCAGACCCAATTCGTGACCCGGTGTGTTTGCTCCAATGGGACAGAACTCCGGATGA
GGCTGAACAAACTACGCAGGACAACAACGGGATGAAAGTTGCCGGGCTGGTGGGGCAGGC
TGTGAGCATTGGTGAACAACCTGTCGAATACAGCCTGTACCTGTTCAGTGGTGACGGTTC
CAAGGTAAAAGTTGGGTCTGGCTCTCAGAACCTGACGGTAACTACTGCCTATGGCTCTGT
TGGTTACACCCCGATTGATGACATTGCTCAGGTGAATCGCGTCATTGAAGACTTTGATGT
GAACTTCAAGCAGAGCAAAGGTCCTGATTGTTCAATCACTCTCAGTGCTGACCGTGCGAA
AAACGAGGCCGCGAACAAAGCTGTTGGCAGTGCAAGCCGCACCTGTCTGTTCGAGTGGCA
GCAGATCCCCGATGGACTGGTCCAAGACCCGTTATCGGAATCTCCTTCGTTGTCAGGTTC
CCTGGCCGAGAACGGTGATCACCCTCTGGGGTGGCGGGTAAGTATTTTCACCCGTAACGG
CACTCGGGTGACGTTGAACGACGAGACTTTCAATGTCGAAGCGGTTGACCCACCAGCTCC
GACCGTTGAACTAGCCTCCGACTACAACTTCAAAGACAACATCTACTTGGTGCCGATGAC
AGGTAACTACCTGGGTGATGCCATTATCAACTCTGAACGAGCTGATCTGGATATTGCCAT
ATCGCGCAACTCTGATGTTCTTGAGTCTGAGACCTTCACTCCGGGATGGGGTGCCACCAA
TAAGGTGTATCGCCGCATCAATACCGATGAGCGAGCGTTGTGGGAGGAGACCACCTACAA
GGTGAACGCGGCCTACAACAAGGTGCCTGATGTGAAGACTGAGGTTGTCTACCGGGCTAT
CTCTGCACCTTCTGACAGTATCCGTCCTATCGTTGAAGTTAAAGGTGATACCGCGATTGA
CACCCAGGCATTGCCGGTTAGGGTTCTCATCCGTGATCAGTACAAACCTGATGGTGACTA
TGACGCTAACACGATGGGGGTGTGGAAAGTACGCCTGATCCAGCAAAAGGCCTACAACGA
GACGGTTGCGCTCACTGATTATGCGGAAGCATCGAACGGTGAAGCTCAGTTCTCAGTAGA
CCTGTCTGGTGTGGATACTTCCTCCGTCCGTATCGCTGCTGAGGCTGTTCTGGAAAGCCC
TGTTGAGGGTTACAACCGCACAGAGTTGTCTATCAGACCTGCCTTCTTGACAGTGCTTCG

TGGTGGTGCCATCGGTGCTGGCGTGGAGGCTCGCAAGTTGTCTGGTGAAGCTCCGTTCAC
TGCTGTGTTCAAGCTATCTTTGGACGACCGTCAGGATCTCCGGGCTACCGGCCAGGTTGT
GTGGGAAACCAGTAAGGACGACGGTAAAACCTGGGAGCAGTTCATCCCAGAAGATCGATA
CAAGTATCAGCTTGTGAAGACCTTCGACAAGGGGGAGTACCAGGTTCGGGCCAAGGTGGT
GAACGTCAACTCAGGTGCGGAAAAGTACACCGAAGCGGTCAGTGTTGTCGCTTACGACAA
ACCTGATATTGCTGTTATCGGCCCGACCACGTTGTTTGTCGGAAGTGAAGGCAAGTACAC
AGCGAACCTGACGTTGAACGATGAGCCAATCTCCGGTGGCAATGCCATTGTTGAGTGGTC
TACTGACGGTGGCAAAACCTACGCGCAGACAGGGGATAGCATCACGCTTTCGAGCGATGA
AGAAACCCGATACCGCCTGTGGGCTCGTGTGCGCTCTGCCACTGCACCGGCTGATGACGG
CTATGCCTATGAAGTTGCGAAAACGGCAGTTGACTTCCGAGCAGTGAAGGCCCCCCGTCC
TTACGTGACAGGGCCGCGAGTCATTGAGACCGGTAAGAAGTATGTGTTCAAAGCCGAAAC
CAGCCTGCCTTACCGTGGGATGGACGTGAAGCTGAACGGGTTCTTCACGCTGCCTGATGG
CTCAATTGTGCAGGGTGATACTGCTGAGTACGTGCCTTCGGACACTGACCTCAACCAGGC
TACTGTAGAAACGAAGTACACCACCTGGATCGAAGGATACCGAGATCAGGGTGCAGAAGC
CTCGCATAGCCTACGCTCCCGCGTGTGGCAGTATGTATGGCCGAGCTTCGGAATGCAGGT
CAGGAAGAACGCTGACGTGGCTCCTGCGACGATCACCGCGTCAGTGCGGCCAATTGCCTT
CAACGGCAAGCTGGAAGAACCGACCTACGAGTGGGAGTTGCCGGAAGGCGCAGTGATTCA
GGATCAGCGGCAGGATATTGTCCGGTCCTTTGTGATCAATGAGCCGGGCGATTACAACAT
CAAGGTGACTGTCCGTGATGCTCGCGGCCATGAAACCGTGATCGAGCAACCGCTCAAGAT
CGGCCAGGCAGAGCCCTATGCTATCGACCTGCAATACTCTGGCTCGAACAAATACGAGCG
TGAGCCTCTGGATGTGCTGTTGCGACCGTACATTTCTGGCGGCCACCCACGCGACCGTAT
TTCGACTCGCGTCTACTCGGTAGATGGTACGCCGTTGGAAAGCAGCGGTTACTACGGCAG
AGCAACTCTGGGCGCTGGTGAGCACAGCATCAAGCTGAAAATAACCTCAGAAATGGGGCA
TGAAGCTGAGGGCGAGGTGAACATCAATGTGGCAGAGAACAAGTTGCCTGCATGTAGCCT
GAGCTCACGAGAGACCGTTGGGTCGTGGATCGTTTATGCGAACTGCGAAGATACCGATGG
CCGCATGAAGTCCTACGAATGGACCATTGCCGGTGAGTTGCAGAGCATCAGCTCTGATCG
AGTGACTATCAGCAAGGGCACCTATGAAACGATGCCGACCATCTCTCTGGTTGGGGTCGA
TGACTCTGGTGGCAAATCCGAAGCTGTTACCATGAACTAAGGTTCATTCCTCTCAAAGCC
CGGTTCAGACCGGGCTTTTTTTTGTATCGCGCTGGAATTGTCCAAATTGGGCTATTACAG
AGGCGAGTCTATTTCCCTCCCCCGGATACACTGCCCAGCATCCAGTAATCCAATCTTAGG
GGATAGACATGCGGACAAAATTACTCGGGGCGCTGATGGTGTTCGGGATTATTACCGGCA
CGGCTCATGCGTCATCGAAATTGGAAATCACCGATCCCAGAGCGGCGAAGATAGAGGACA
TCGTAGAGCTACCCATCAAAGGGGTTCGAGCCGTCCAAAGTGATGGGCAGATCATGTTCC
TCTCTGAAAACGGGCGATTTGTTATTTCAGGACAAATCTACGACCTGTGGAGCAAGAAGC
CCCTCAACACGATGTCCCAAATGAGGGATGTAGCGGAGCGTATCCACTTCAAGAGCATGG
GCATGGATGTGGACACGCTGAACACCGTTTCGATGGGGCGTGGTGACAAAGAGGTGGTGG
TCTTTGTCGATCCTAGATGCGCGGTTTGCCATCAGCTCATGGGTGATGCCAAATCGCTGG
TGGATGATTACACCTTTAAATTTATCGTGATTCCTGCTCTGGGTGCTGAGTCCAACCGCT
TGGCAAAGAACTTGTACTGCGCGAAAGACAAAACCCACGCGCTCGATGCGCTGATGAACA
ACACCTTGGGTTCCCTTCCTTCAAAAGAAACCTGCGACCCCGGCCAATACGATCAAACGC
TGCTGACAGCTCATTTCATTGGGATTGAGGGCGTTCCGTTCGTGGTTGCTCCGGATGGTC
GTGTCAGCAAAGGACGTCCGAAGAACCTGAAATCATGGTTGGAGAGTGTTGAATGATCGT
AACCATCAAAAAGAAACTCGAAGAAACGTTGATCCCGGAGCACTTGCGAGCTGCCGGGAT
TATTCCTGTCCTGGCCTATGACGAAGACGATCATGTCTTCCTTATGGATGACCACAGTGC
AGGCTTTGGTTTCATGTGTGAGCCCCTGTGTGGTGCCGATGAAAAAGTTCAGGAGCGAAT
GAACGGTTTCCTGAATCAGGAGTTCCCGTCGAAGACTACGCTCCAGTTTGTTCTGTTCCG
CTCCCCGGACATCAATCAGGAGATGTACCGGATGATGGGGTTGCGTGATGGCTTCCGTCA
CGAGCTGCTGACATCTGTCATCAAGGAACGGATTAACTTCCTCCAGCACCACACGACAGA
ACGCATATTTGCCAAGACCAACAAAGGTATCTACGACAATGGCTTGATCCAAGACCTCAA
GCTGTTCGTTACGTGCAAAGTCCCCATCAAGAACAATAACCCGACTGAAAGCGAACTCCA
GCAGCTCGCACAGCTTCGCACGAAGGTCGAATCATCGCTTCAAACCGTTGGTCTGCGTCC
CCGCACAATGACGGCGGTGAACTACATCCGGATCATGAGCACCATCCTGAATTGGGGGCC
GGATGCTTCATGGCGACATGACTCTGTGGATTGGGAGATGGATAAGCCCATCTGCGAGCA
AATCTTCGATTACGGCACTGATGTGGAAGTCAGCAAGAACGGCATCAGGCTGGGGGACTA

CCACGCGAAAGTCATGTCAGCGAAAAAGCTGCCTGACGTTTTCTACTTTGGTGATGCGTT
GACCTATGCCGGGGATCTCAGCGGCGGCAACTCCAGCATCAAAGAAAACTACATGGTTGT
GACCAATGTGTTTTTCCCTGAGGCAGAAAGCACGAAAAACACTCTGGAGCGCAAACGCCA
GTTCACTGTAAACCAAGCCTACGGGCCGATGCTCAAATTCGTGCCGGTGCTGGCGGACAA
AAAGGAGAGCTTCGACACTCTCTATGAGTCCATGAAAGAGGGGGCTAAGCCAGTCAAGAT
CACCTACTCGGTGGTTTTATTTGCTCCAACCAAAGAACGTGTTGAAGCGGCGGCGATGGC
CGCACGAAACATCTGGCGTGAATCTCGGTTCGAGCTGATGGAGGATAAGTTCGTTGCTCT
GCCGATGTTCCTCAACTGCCTGCCATTCTGTACAGACCGGGATGCAGTGCGAGACCTATT
CCGCTACAAGACCATGACAACCGAGCAGGCGGCTGTGGTCCTGCCGGTGTTTGGGGAATG
GAAGGGGACCGGGACCTATCATGCAGCGCTGATTTCCCGCAACGGCCAGCTCATGAGTCT
GTCTCTTCACGACAGTAATACCAACAAAAACCTGGTGATCGCAGCCGAATCCGGCTCGGG
TAAATCGTTCCTTACCAACGAACTGATTTTTTCCTACTTGTCCGAGGGTGCTCAGGTCTG
GGTTATTGATGCCGGTAAGTCCTACCAGAAGCTGTCGGAAATGCTCAATGGCGACTTCGT
TCACTTTGAAGAAGGAACGCACGTCTGCCTCAACCCGTTCGAGCTCATACAGAACTACGA
GGACGAAGAAGACGCGATTGTCAGCCTCGTTTGTGCAATGGCGTCGGCCAAAGGCTTGCT
GGATGAATGGCAAATCTCTGCGCTGAAACAGGTCCTTTCTCGCCTGTGGGAAGAGAAAGG
TAAAGAGATGAAGGTTGACGACATCGCTGAGCGCTGTCTGGAAGAAGAAAACGACCAGCG
CCTCAAGGATATTGGTCAGCAGCTCTACGCCTTTACGTCGAAAGGTAGCTACGGGAAATA
CTTCTCTCGCAAGAACAACGTCAGCTTCCAGAACCAGTTCACTGTACTGGAGCTCGATGA
ACTGCAAGGGCGTAAGCACTTGCGTCAGGTTGTACTGCTCCAGCTTATTTACCAGATCCA
GCAAGAAGTATTCCTGGGTGAACGTAACCGCAAGAAAGTCGTCATCGTGGATGAGGCCTG
GGACCTGCTCAAAGAGGGCGAGGTCTCGGTCTTCATGGAACATGCCTACCGCAAATTCCG
TAAGTACGGTGGCTCCGTTGTCATTGCAACGCAGTCCATCAACGACCTCTATGAGAACGC
AGTGGGCCGCGCCATCGCGGAGAACTCGGCCAGCATGTACTTGCTCGGCCAAACCGAAGA
AACCGTGGAATCTGTTAAACGTAGCGGTCGTCTGACCCTTTCAGAGGGCGGGTTCCACAC
CCTCAAGACGGTACACACCATCCAGGGCGTGTACTCAGAAATCTTTATCAAATCGAAGAG
CGGCATGGGCGTCGGACGCTTGATAGTGGGCGACTTCCAGAAGCTGCTTTATTCGACCGA
TCCGGTGGACGTTAACGCCATCGACCAGTTTGTGAAACAAGGCATGAGCATTCCTGAGGC
AATCAAGGCCGTGATGCGAAGCCGTCAGCAGGCTGCATAACCAGGGAGACAGTAATGGAC
ATTAAATCAATCGCAATCGCCGCCATTCTCGGTGCCGCTGGTGGCTTCGGCGGTAGCTAC
TACGTGATGAGCGAACAAACGGCAAGCATCCATCAGCGTTTGAATCAAACCCCGCCAGTG
GTCGTGGTTGACTTCGCTAAAGTGGCGTCGGCGTATCCCGCTGGTGCCTCTCAGGAGGAA
GTTGAAAGACTGATGGTCAAGACCAATGACGCAATTTTGAAGTTGAAAGACGCAGGTTAT
TTGGTCCTTGACGCAAGTGCTGTCGTCGGTGCTCCAAGTGACGTGTACCTCCCTGATGAG
GTGCTGAAATGAATTTTCCACTCAAGAAGTATTTCGTCAAAAAGGAATCCTGGAAGCGCT
TCGGGGTTAAGGCCGGTGTGACACTACTGGTTCTTTGGGCTGCTGGTGCGGCCTTTGCCA
GCCGCTACCGTATTGGCATTGATCCACAACAGGAGAAGTGCCTGCCGGGTTACACCTTCT
TCCTCATTGATCTGAACGACCAAACTCTGGAGAGGGGAGCTGTTTACGCCTTCCAAGCCA
AGAACATGCAGCCTTTCTACAAGGACGGGACTCGCATGGTCAAAATCCTCACCGGTATGC
CGGGGGATAAAGTCGAGATCAACGATAAGTGGAAGATCACCGTCAATGGTGATGTCGTCG
GAGAGGGGCTCCAGCTCGCAGGGAAACTACATCTGCCAGAGAGCCACTTTTACGGCAAGA
CCACGCTGAAAGAGAATAACTACTGGTTTATGGGCAAAAGCCCATTCAGCTTCGACTCAC
GTTACTGGGGGACTGTGAAAAATGATCAGATCATTGGCCGCGCATATCCCCTGTTCTAAG
AGCGTTCTGGTGGCGTTGATATTCTCTGTGGCTGGCGGGGCATACGCTCAAGAGTCTCCG
CTCACAGAGCAGGATAAGGCGCTTATTGAGAAAGGAAAGCAAATTGCCCAAAAGGCCCAG
AAGATGGAAATGCCATCTCTGTTGCAAAACCAACACATGGACGAGGCTCAGGCCGAAGCC
AAGGCATTTTTCAAGCAGCTCCAAACTACTAACCCAACGCTCAAGGAGATGCACCGGAAA
CAGGCTGAAAAGGGTATCTACTCTGACCATCGGATACTGGTTTTCGCCTCGTTGTCTCTT
GGCGAACAGGGGTTAGATGACGTCCTAACGGCGGTGTCAGGCCAGCCTGATTCTGTAATT
GTGTTCCGTGGCATCCCGGAAGGAATGAACTTGGGGCAGGGAGTTAAAGCTATTCAGGCG
CTCGCGGCCAAAAAAGACCCAGTGCCGAACATCATCATCAACCCTACGTTGTTCAAAACG
TACAACATCACAGCCGTTCCCACGATTGTGATGCTGGAGGATGAGCCGCTGCCTGGCGAA
CAACCAAACGTCGTCGCCCAGGTCTCCGGGTTGTCCGACCCGGTATGGTTGGCTCGGGAA
GTGGATAACGGAGAAAAAGGCGATCTCGGCGTTAAGGGGCCGGTGGAGAAAATCAGTGAG

CCAGACCTTATTGATGTTGCCAAGAAACGCCTTGCCAATATCGACTGGGAAGAGAAGAAG
AAACAGGCTATAGAGCGCTTCTGGACCAAGCAGAATTTCAATGAGCTGCCCAGAGCGCCA
AAATCTCGAACACGAGAAATTGACCCTAGCGTCATGATCACCAGTGACATCAGCACTCCG
GATGGCACTGTGTTCGCTCACGCGGGTGACGTGATCAACCCATTGTGCGATCCGAAGGAA
GTTTGCAAGCCTGGAACGCGGCCATTTACCCAAGCGGTCGTAGTTTTCGACCCGCTGGAC
AAAAAGCAAATGGAGCTGCTCGCCAAGAAGCTGCCTGAAATTAAGCAGGAACCTGGCGTA
CAACGGATTACCTATATCGCCACAGAGTTCGACAAAGACAAAGGCTGGGATTCCTACAAG
AGTGTCACCGACAACTTTGACGCGCCGGTATATCTGCTGACGCCAGATCTGATTACCCGG
TTCGAGCTGGAGCACACACCGAGCGTCATTACTGCCAGAGGCAAGAAGTTTGTTGTCCGC
GAACTTGCTGAGGAGGGCGGTGAATGATTTTTGCCCCGGCTTTCCAACCAATTAAAGACG
TCGGCACAGGTTCGTTTGTCGCTGCTGAGGTACTGGCTCGTTGGTACGACGAAGGCCGGG
TTCTTACACCATCCTCTCTGTCATCACCTCCTTATTGGGGGCTGGTGGATATGGAGATGG
CACGGTTCATTCAGGACAACCTTCATTATTGCTTGGATCTGTACCCAGCTCTTTTTCTGA
ATGTCTCTGAACATACTTTGCAATCAGACGTCATTTTCAAAGCGTGGTGGAGGGTTGTCC
GCGACATTGCTAAGAATCACTCATCACGGCTTGTCATCGAGATTACCGAGGGCATTCAGG
ATGCCTCTCTCGCATTGAGATGGGAGGCTCTTACCGAAATAGGGGTTGAGCTGGCGCTGG
ATGACTATGGAGACAAAAACTCTTCGCTGGAGCGCCTGAGTCGTTATGACTGGCACTATT
GCAAGTTTGACGCGAGAAGACTGCGGTCGCTTGAAGACTACTCGGCCATCCTCCACTGCC
GCCGAAAAGGCATACAGCTCATTGCTGAGCAGGTGGAGAGCTTCCCATTGGGGGAGAGCG
CCAAATTACTTGGACTGTCATGGCAACAAGGTTTCTATCACGGGAAGCCTGCTGTCATGG
AGAAACACTTGAATTACGTAAAGGCCTTACCATGATGCAAAAAATTCTACGGGTCATTGC
CGTTAGCGCGGTCTTTTGGGTTCGTTCGGTATCGGCTGACCCTGGGTGCCAGAATGCGGA
AGTAATCGGCGGAAAACTGATTACTGACATCTGCTGGAGCTGTATTTTTCCCATCAAAGT
AGCAGGGGTTCCTATAAGTGGTGGAGGCGGATCATTCCCGAGTGAAGCCGTAAGCAACCC
TCTGTGTATGTGCGAGGATAATCTAGGGGTCCCTCGGCCTGGAGTCACCACTTCTATGTG
GGAGCCAGCACGGCTTGTTGAATTTCAGAGAGTGCCTGGCTGCTCATCTGTCTTGAATGG
TGTCAGGTTCCCGTTTGATAGGACTAACCAAGGGCATCATGGCATGGGAGACATGGATGG
TGGTGATGGTTCTTTTATGCACTATCACTACTATGCGTTTCCTCTGTTGGTGATGCTCGA
TTTATTTATTAAGCAGACCTGTAATGCTGATGGGTATATGGATCTCGACATCATGTACAT
GTCGGAGCTCGACCCGACCTGGAACAATGATGAGCTCGCTTTTTTTACCAATCCAGAGGC
GGCGGCAGTAGCAAACCCAATTGCGGCGGCTGCGTGTACTGCTGATGCTGTCTCATCAAC
CGCTGGAAAACCTTTAAAACAGCTTTTCTGGTGTGCTGGTTCATGGGGCACCCTGTATCC
ATTTAGTGGCAACCAGAATGGTGGAAAAGGTGTTATCCGCGATAGCAGTCTTCTTAGCAC
AAGGGTTCTGGCTGCTTTGCATCGCCGGGGGTTAGCGTGGAAGACAATGGGTTCTGAGGC
TATGTGTAGAGGTGTTATTAGCCCAACACTACCCAAAACGCAGTACAAATTCACGCTATT
GCATCCGGTTCCAGAGACAAACTCATCTCACGTAATTGGCGAATCCACTCTTACGTGGGG
TCTGGCGCGAACTATACCGGCAATTGGGCAAGACCCTATTTACACCATCTGGCGATGGAA
TGATTGCTGTAACAATTGAGCAGACCCTAACGAATTTGCACGGGCATTTTAGATTATGCC
CCAAAAGGGCCACTACACGTACAGGTGGCCCTTGTCTTCTCAGATAGCATGGGAAGGTTG
AAATGGAGAACACAATGCGAAGTCATAATTACTTTATGAAAGCTGTGGCCTCGCTGTTGA
CAGTAACCATGTCTGCGCTGCCGATACATTCCTACGCTAACGGTAGCCAAGACCAGGACA
TCACAGCGGTGGGTAAAGAGGCCCAGGCTTTCGGACAAAACCTCTCAAACTCATTCAAGT
CGAGCTCGGGGACTGTGCAAGATGGCACAATCTCTATGCCGACGTTGAAAGACGGTCAAT
TCCAAATGAACGGGGGGAGTCAGATTAATGTCAATGATCTATTCCCCGGAACGAGCGGGA
CCAACAATAAACCTGATAGTTATTACTTCCCTGATGCCAATAAACCTGATGTGGGCGGTC
TGCAAGGCATTTACGACTCTGGCGATGACATGGACAGCGTGGGGAATAATGCAAAAGGGT
CGCTGTGGAGTGATGCCAATAGTGCTAATCCATCAATCTCTGGTGCGGCATACAAGGTTC
TTCTCGATGCCTCTAATCGATCACGCCCTGATTTCAGTAATGACCCTGTACTAAATCTAA
GCAAAAAGACCTATGAGGATATGGACCTCATCGCAGGTGGCTTTGGGGATTGTTCTGCCG
AAACAACCATCAATCAGAATACTATCAACGCCCACATTCCAGAGTATGAACGGTGTCAGC
GTGTTGTAGATCAAAGCGCGGACTGTGAGGTTGTCCATGACTACGATGCCTCTGTGGTGA
AGCACTATGATGGTCCATATAACCTCAAATCTTGTGGAGAAGGCTGTACTGAGTTGTGGA
TTGGCCGAGTTGGTAACGACTACTGGAGTGGCAACTGTTCGATTTATGAGGAATACACGC
GGGTCCAAGTCAGTAATCCAGACGCCATAGTGTCTGCAACTCTTGAGTACGCCAAGTGGG

ATGACTACATGCAAGTTTGGGTTGGTAAATCAGGTCAGGAAACTAAAGTATGGTCCGGCC
CTGACGGCAATTTCCCTCCAGAAACGGCTGGCCGATGTGAATTGTCAACAAGTTGGGAGC
GAAACCCTAATATTGATGTCACTCCCTATTTCAAGAATGTGAAAGATGGTGATGTTGTTA
CGTTTAAGATCCGCGTTTCAGTAACTGGCGCAGGTGAGGGTTTTGGCCGCATAAAGCTAC
GCTATGACCCATCAAAAGCCATTACCAAGGATGAGTGGGCTCCACAGAGCTGCATGGATT
CAGCCAAAGGGGTTGTAGATGGTTTTGCGGAAGGCGAGATCACATGTATAGATGACCCGA
CTGATGCTACGGGCTGCACAGTCATCAATGGGATCAAAGTTTGCGAATCTCAACTCAAGC
CGTCACCTTTGCCTGGTATTCCAAAACTATGCAAAAAGGTTCGAGTTAAAGCTGACTATG
ACTTTTATAAGGGGCAAATGGACTGCTGGACAGACCCTCAAGGTGAAACGCACTGTCCGG
TAAACACGGGCGGGAATCTCGATAGCTGTCAGAAATATGAAGAAAACCCTCAGTGCGGCT
TCATCAGTTCCAAATGTGTTGATGGTGCTCAGGGAAGTTCTGGTACGTGCTACGTCCACG
AGGATACTTATGACTGTGGTACAGATGTTTCTGTTCCGACCTTGGAAAAGGAAACTGAGT
ACCAGTGCGGTGGGCCTATACGCTGCATGGGAGATGACTGCCTTGATTTGACCAAAACAC
AAAGCACTGATTTTGCTCGCGCTACTGCGTTGCTCAATGCAGCCCAATTCATGACGCAGG
ATATGAGCTGCACAGGCCAAGATGGGGATGACAATCCTACCGGGGATGAAAACGTTATTT
GCTCTGCTTTTGCAGGGGAAGCTGGCGAATGCAAGATAGCTGTTGGGGGAGTTTCTGATT
GCTGTGAAAAGCCAACCAATATATCTCTTGCCGATTATCTGAACCTAATAATGGCCGTTC
CAAAGCTCGATGGCGCAGTGATGGGGCTGACTGATGGTAATGCGCTTAAAGGTGCTTATC
AGGTACTTAGGGAACCTGCCCTTCAAGGGTGGACAGAAGTCACAAAACCGTTCACAAGTT
ATATAGAGAACGTTTCAGGTGCTGTTGATTCGTTCTTCCAGCCTGTAGAGCAGTTTGTTG
ATCAACTCATTGACCAGCTCAAAGAGCAAGTCAAAGAAGTGATGATGGATGTCATGAAAT
CAGCAGGCCAAGATGCAGCAACAGAGCAGGCGGCTGCCGCAGCATCTGAACAAGCTGCCG
AAGCAATGATGGAGACTGCGACAACATGGCTTAGCACTGCCATGACGATATATACCGTCT
ATGTCGTTGCGATGGTGATGATCCAGATGATTTATAAGTGCGAGGAAGAAGAGTTCACTA
TGAACGCCAAAAGAGCGCTCAAGAATTGCACCTATGTAGGCTCTTATTGTAAATCTAAGG
TGTTGGGCGCTTGTATTGAAAAAAGAGAAGCGTATTGCTGCTTCAATTCTCCGCTCTCTC
GTATTATACAAGAACAGGTTCGCCCTCAATTGGGGCAGAACTTTGGAGACCCCAAAAATC
CTCAGTGTGAAGGGATTCCACTAGATAAAATTGCCGAAATTGATTGGAGCAAAATTAATT
TGGATGAGTGGCTTGGGATATTACAGCAGAACGGTAAATTCCCTGATCCGGCCTCAATAA
ATCTCGACTCGCTGACTGGAGCAGGGAATGACTTCAATATTGACGGGACGCGGAAGAATG
CTCAGGAAAGGGCGTTGGAGCGGTTGGAAGGGATTGATATTGACGCGAAGCGAAAAGAGG
CGACAAACAGCATAGACCCTCAAACAGGCGCGCCAACTGGTGGTGGTGGATAAAAAAAGG
GGCCTTTCGGCCCCTTTCTCATGTAGATTGATTACGCTTCTGGGTAAACAAATAACTCTG
CTTTTCCCTGATCAAGAAGGTAAGGGAAAGCAACTTTTTCGCCATTATCCAGCTCAACCG
ATTTTGGAGCAAGGTAGAGGTAGGCCCAAGCTGGTAGCTGTTTGCTAATAGCTGCGTAGA
TCGCTTGCTGAGGAGCATGGCTTGCTGCCCCATTACCGACATTCACAAAGTTGTATGCGG
TCTCATGGCCTGATGTGAAGGCATACGCTGTACCTTGAACGCCAGAGACAAAATTAGGAG
CTGACCCTAGGCGAGGCTCCACGATTTTCACCTTAACGGAACACATGTCAGCTACCTTAC
TTTGTCCGTTCACCCACGTCGGGCAACCCCATTCATTCTTGTAGAACTGATAGGTTAGGC
GACCATCTTTGTCATACAGAAGCGTGAAATTGGCACCCAAATCAGTCAGCGCTGACTCAA
TGGAAACTTTCACATGTGAAAGAAATTTGGCCTGAGCGTCTGCCGGTGAAACTGCTTCGT
TAGCAGGCATCCAAGCGATCAAGCTATTGCGAGCGCCATGCTGTTTGGGGCCGATAGCCC
AGTTCGCAAGGTTGACCAAGCCACCTTGCCAGTCGGTCATTCCGAGCTGAGGGGACATGT
AGCCAGACAGCACGTAGGCTGCCCCAAACGTTTTGGTGTCGGTCAGACGCTCCAGCTTGT
CACGCGGAACAGAAGCGTCTTGGATGCCTGTTACCAGGCCACCGGCCTCCGCGATGTTGT
AAGCGCGACTGTGATTGGTTTTGTACGCTGTGGGTTCGTAACTGCTGGTGGATGCACAGC
CAGATAGAGCTGCTACAGCCAGAGCCACGGTGGTGATTAAAGCCTTTTTCATGTTGTTCT
CCGTTCTTACCTGAGTTGAATCTGATTTACTTCGATAATATCACAGCATCAAAAAATGCA
ACCATAAAAGACAAAAAAAAGCCCCTCGGGCGAGGGGCAAAGGTTTGGGCTGGGTTATGC
TGCTTTTGTTTTCTCGGCTTCGTCTGCGATTTCCTTGTCGCCGGTTGCAGATTCCGGAGC
GGCTTCGGCCACTTCACCTTCTTTGCCTTTGCTGTGGGCGGAAATGCGAGCCCGTTTCTC
GATGCCGATGATTCGACCGGCCAGCTTGATGAGGCGTTGCTGCCATTGGTAAGTGGCGTC
GGTGCGCTGCTTGCTGGTCAGTACGGTGTTCAGCCAGAGAGTGTCAACGATCCCCATCAG
GGTATCCAGTTTGCGGATCAGGTGTGCAAACTGAGCAACCTGAGGAGAGTTGATCTCGAT

GGTGTATTCATTCGGGTTGGTGTAGCCGGGCATCATGTCGATGCCGTTGTCTTCCATCAG
CTTGTTGAGCTGGGCGGTAGCTTTGTCCAGGTCTTCGGAGACCTTGGAAATGTGTTCCAG
GATGACGGTTTCAACCTGGTCGATTTCGTCCTGCTCACCGATGATGCGAAGGATCACGTC
GATGGAGAACAGGGAGTTGGAAACTCGCTCAAAGCTGCGTTCCATGACGCGCTGAGCTTG
GAGGCTGTTTACTTTCAGAACTTGCTTGAAAACAGGGCGAGAGTAGTGGTTGCTGCGGTC
GTTGTTTGTGTCCAGTGCTACTTGTGCTTCTGCCATTAGAGATACTCCTAAATTTGAGGT
ACAGATAAACGCACTGGAAAGGATAAATGCGGTCAATCTGTAACCTGACCTCTCGAAGTG
CCCAAAATGGACAGTTACAGCGTTTTCTGTTTAGCAAAGGTGAGCTATATTTGAATCATC
TTAGTCGCTCTGCGGCTATAGGTGAGATAGCTGGTCTCCGAAAACAAACAGCATCTAGTC
TTATGCCGTATGGCATTACTTAACCCCAAGCCGGGGGCTTCTCCCGGTTTTCGGGTGTTG
CCCCTGTTTTTTTACATAGGAGCAACACCATGCACATCATCACTTTCATTGTAGTTCTGC
TTGCATCTCTGGGCGCACTGTACGTAGGCGCGAAGGCATTTGCTTTTTCCTTCATCGTTC
TCGCTAAAGTGTTCCAGCGTATCGGTTTGTTCCTTTGGAACAAATTCGCAGTTCCTGCAA
TGCGGTCTGTACAGGCCGTCATCAAAGAACGTGCTGCCAAGGCTGAACAAGCCAAAGCAG
TCGTTGAACCTGAGGTAACTCAAGTTCAAGAAGTCGCTAAACAAGAACCTGACTGGGATT
ATCTCGAAATCCCGACTTACCTTCGTAAGGGCAAGGAACTTGTTTGGTAATAACTGTCGC
TGAGTAATCTTCGACACTTTCAAACCCATCGGGGCAACCATACCCCGACGGGGATGTTGC
CCCTTTTTGCTTAGGAGCAACATCATGGAAATCGCTATCATCGCTTTGTGTATCCCTACT
GGTTGGGCAATCTCTCGTCCTTTCGTGAGAATCGCTGAATCGTTCGGTTACTGCTGATAG
TCAACGCAAAGGGAGGGGGCAACCCCTCTCTTTCTGGTCAAGCCTCTGTGGCTTGTCCTC
AAAGTCTTCTGGTGTCAGTTGGCTTTGACGACAAGCTACAGGGGGAATGTCGAATGCAAC
AGAATCTTCATGTTTACGACGATCACGCAGGCATCATCTATCTGGCTGATGGACGCGAGG
TGAAATTTGATCCGAAACTGTATTCCAGCTCCTACCAGGCGCACAGTGAAGCAGTGAAGT
GGGCCAAAGAAACCGGGGTCATTGGTCAAAACGACGATGTGGTGATGTTCGTCCATTGAG
GGAGTGGCCTCCAGGAGAGGCTACTCTCCTCGCTCCTTAAAAGTCTTCTTACTCATGGTG
AATACCATCAGTAAGCAGATTTTGTTCTCTTCGGAGAGCGAAAAGTGCGATAGCTGGTCG
CCAAAAACAAACAGCAAATTAACGTTAATTTACTAGCCCAACCGGGCGCATCCGCCCGGT
TCGGGACGTGGTGCGCCTGTCACAATAGGAGCGCACCTATGTCTCAATACTCTCAATTCT
CAGTAAGCAAGGTTTTCGGTATGCCCAGCATTCCGGAGAAGGTAACGGCCATCGGCTACG
CTGACGGTTCAAACCCTTTCATCCCTGCCACTGATACCAACTACGTTTTCCGCAAAGAGT
TTCTGCGAGAGGTCTTGGCCTATCTCAAAGAGCCTGGCGGTGACGCATTGTTCGTAACCG
GCCCTACCGGGTCTGGCAAAACCTCAGGTATCACCGAGATCGCTGGTCGTCTCAACTGGC
CTGTTCAGCAAATCACTGCCCACGGGCGGATGGAGCTGACAGATCTGATTGGACATCACG
CTCTGGTCGCGGAAAAGCCTGGTCAACCACCTGTCATGAAGTTCATGTATGGACCTCTGG
CAGTCGCTATGCGTGAAGGTCATCTGCTCCTCATCAACGAGGTGGATTTGGCCGACCCTG
CCGAGCTGGCTGGTCTCAACGATGTCCTTGAAGGGCGTCCCCTTGTGATCGCTCAGAATG
GCGGGGAAATCATCAAGCCGCACCCGATGTTTCGTGTGGTCGTTACTGGTAACTCTACGG
GGTCCGGTGATGCTTCTGGTTTGTACCAGGGGGTGATGATGCAGAACCTCGCAGCTATGG
ATCGCTATCGTTTCACCAAAGTAGGGTATGCGGATGAGGAAGCTGAACTCAGCATTCTTG
GCCGTGTTACTCCGAAACTTCCGGAAAATGTGCGGAAGGGAATGGTTCGGATTGCTAATC
AGGTCCGCAAACTGTTTCTTGGCGAAAACGGTGAAGATGGTCAGATCAGCGTCACCATGT
CCACTCGGACGTTGGTGCGTTGGGCGAAACTGTCTCTAGCGTTCCGTGGTGCCCCAAATG
CTCTTGAATACGCCCTGGATCAAGCTCTGCTTATCCGTGCGGCCAAAGAGGAGCGTGAAG
CCATCCTGCGTGTTGCGAAGGATGTGTTCGGGGACCAATGGCGCTAAGGGGTGACGCATG
AAGAAAAATGACTGTTTGTGCCGTCGTTACACTGCTAAAGAGTGGGGCAATGACGAAACC
ACAATAGAAGTCTTCATTGGCTACAAGTTGCTCCGGGAGCCCAGCTCCTCGGAGCCAGGC
CAATTCACGATGGTCGAGCTACGCCGAACCGTCACTGATGGGAAAGCTGAAAATTGGTCT
GAGACAAAGCTCGAAGGACCTTTTGAAGCTAACGGCCCAGACACGATCCCAATGTCCTAC
AAGGACAAAGAAAGCCAGTATGTGTCACAGTTTCTCAGCCAGGGGTACACCTTTCTGGAT
GAGGTGCTGGTAAACGCAGAGACACAGACGGTGCTGGAAGGGGGGAATGTTTCAGCCGGA
CAAACAGCCAGCTTGGGATCTCTCAACTGGCTGTTATCTCCGCCCTCTGAGTTACCTCCA
GGTGACATAAACCTCTTCAAAGGTTTTGTTGCTGGGGTTTTTGCCAAAGGAGCCGGTTTA
ATCGGCTTTGAGGTTGCTCGGAGCGAAGGCTCAAATGACTTGCTACCCAGTGTGCTGATG
CGTACTGACAGCGGTTATGAGCTTGGGGTTAGCACTGGACTAGGCGAAAACACTATCCAT

CCAGCTACGCTGGAAGGCGCTGGTGAACTCCGCCCGGAACATGGTCACAAACCCCTGCTG
ATGTTGGTTTACCTGCAACAGCGTTTCGCGGATGACTTTTCAAACGTAGAGAAGCCGCTG
GTGGCATTCTGTGATGAACAGGGTGATACCTTCGACTACGAGCGTTTTGATTCACTAAAA
CCCCTCATTGAACGGTTTGGTTTCAGCTACGACGAAGTAAGAGCAGATGCAGAAAGGCTT
GGCCTTGTATCTGAGCTGATTCGCCTGGCAGAGATCGACGCCGAACAAGAGGATCACTTT
TTTTAACCCTTACGGGGGCTTGCTCCCCGAAAGGGGCGTTGGCCCTCTCCAAATAACTTG
GAGGTCATGATGTCTAAAGGCGTCAACAAAGTAATTCTGGTCGGTAATCTCGGTTCTGAC
CCGGAAATTCGCTACATGCCAAGCGGAACTGCCGTTGCCAACTTCAACGTTGCAACAACG
GATACGTGGCGCGATAAGCAGTCTGGCGAGCAAAGAGAGCATACTGAGTGGCACCGTATT
GTGCTTAAAGGTCGTTTGGCAGAAGTCGCTGGTGAGTACCTGAAAAAGGGCTCCCAAGTC
TATCTCGAAGGGAGCAACCGCACCCGGAAGTGGACTGACAGCCAACAAATCGAGCGCTAC
ACCACCGAAGTACACTGCGTTGAAATGCAGATGCTTGGTGGTCGTGGAAATGCACCTCAG
GACAACTCTCAACGTGCAGCGCCCCAAAAAGGGCAACGTACAGGAGCCGGTACGCAATCT
GCTCCTGTGCAGCAATCAGCACCGCAAGGTGGTATGGGCGGAGGCTATGGTCCCGCTCCT
GATGGCTGGGATGATGACATCCCGTTCATGCGGCTGCACCACTTGGCTGGCGGGTAACAC
CGCCAACTCTTACTAAACCCCACGGGGGCACTCATGCCCCGAGGGGGTCATGGTGTCCTC
AAACCGTTCATTCGTGAATTGGAGAAACACCATGTCTGATAACAAATCTCTTGTAACCCG
TATCGCAAGCCGGTTTGGCGTGGACACCCGAAAGTTCTATGAAACTTTGAAGGCGACCGC
ATTCAAGCAGCGAGATGGAAGTGCCCCGACCGATGAGCAGATGATGACGCTCCTGATCGT
GGCTGAACAGTACGGTTTGAACCCTTTCACTCGGGAAATCTATGCGTTTCCTGACAAGCA
AAATGGGATCATTCCGGTAGTAGGTGTTGATGGTTGGAGCCGCATCATCAACGAGCATCC
CCAGTATGATGGCGTCGAGTTCGTGTATTCGGACAAGATGGTCAGAATGCAGGGGGCGAA
AGTTGACTGCCCTGAGTGGATTGAATGCGTGATTTACCGTAAGGACAGATCTCGCCCTAT
CCGCATCAAGGAGTTCATTGATGAGGTGTACCGTGAACCGTTTCAGGGTCAAGGTCGCAA
TGGTGCTTACACTGTTGATGGCCCCTGGCAAACGCACACCAAGCGTCAACTCCGGCACAA
GTCGCTGATCCAGTGTTCTCGTGTCGCATTTGGTTTCTCTGGTATTTATGACCAGGATGA
AGCTGAACGCATCCGTGAAATGGAGCAGGCATCGGCCATTAACCCGGCTATTGCCAATCT
CCCTTCACCATCTCAAGTTCAAAGCCAAGAGCCTTTGGCTATTGAGCACAAAGAGCTTGA
CCCGATCCTAACCAAACTCGCAAATCGCGCCATTGCTGAAAACGCATGGTCTGCGGCGCA
TGAGTATGTGAAGGGACGGTATGAAGGTTCGGAACTGCAATATGCGACTCAATTCCTTCG
TGACAAGGAGATGGATCAAATGGAGCCTCCGAAACCTGACTACCAGGAAGCGCACGAGCA
AGAGTCCGCCGCTGGTGGTTCTGCAAATGCTGAACCTGGTGCCGAAGAAATGCCGCCTTT
GAGTGACGAGGACATGATCCCTGTTACAGAAGAGGAGGGCGCGGAAGGCAGTTACTACTA
ACCCCAACGGGGGGGACTCCCCCGCTGGGGGAGATCTCCCTCCTAACCATTGGAGAGAGG
TCCATGAAAATAGTCAACCTATCGCAACGAGAGGAAGATTGGCTTGATTGGCGGCGTCAA
GGTGTAACAGCCACTGACGCCGCTATCCTGCTCAATCGGTCTCCGTACAAAACACGATGG
AGACTGTGGGCCGAGAAGACTGGGTATGCGCGTGAAGTCGATCTGAGTCTTAATCCGCTG
GTTCGCCGGGGGATAGAAAACGAAGATGCTGCAAGACGCGCTTTCGAGGAGAAGTATGAT
GACATGCTGCTCCCCGCCTGTGTCGAATCGGTTCAATACCCGCTCATGAGGGCCTCCCTG
GATGGCCTGAGAGATAACGGGGAGCCCGTCGAGCTGAAAAGCCCGAGTGCGACTGTCTGG
GAAGATGTTTGTGCTGAGAAAGCAAACAGCAAGGCATACCAGCTTTATTACCCGCAGGTG
CAACACCAGCTCCTGGTAACGGGGGCCAAGCAAGGCTGGTTAGTCTTCTACTTTGAAGGT
CAGATTCAGGAGTTTCCAATACTCCGAGACGAAGCCATGATTCAAGAAATCTTGGCCGAG
GCTAAAAAGTTCTGGCAACAGGTAGTAGACAAGAAGGAGCCCGACAAAGATCCAGAGAGA
GACCTGTACATACCGCAAGGTGAAGAGGTCAACCGTTGGATTGCTGCTGCTGAGGAATAC
CGCCTCTATGATGCTGAGATTCAGGAGCTGAAACAGCGACTGTCTGAGCTTCAAGAAAGG
CAAAAGCCTCATCTCGACACCATGAAGTCCCTCATGGGGGAATACTTCCATGCCGACTAC
TGCGGTGTGATGGTAACGAGATACAAAGCGGCTGGCCGGGTAGACTACAAAAAGCTGTTG
GCTGATAAGGCGTCAGGCGTGAAGCCTGAGGATGTTGACCAGTACAGAGAGAAGTCATCA
GAGCGGTGCCGTGTAACGGTTACTGGCTCTGTGAAGCCACGGTACATTGTTGATGAGGAC
GTGCTTGCTCCTCTTGATGATTTGCCGGAAGAAGTAGAGACGTTCTACTGGTGAGTGGGG
GATTTTCCCCCGCTTTCCCTTGGGGATTCTGGGAGTCTCCAACGGAAAGCAAGATTTTGT
TCTCTTCGGAGAGCGAAAAGTGCGATAGCTGGTCGCCAAAAACAAACAGCAAATTAACGT
TAATTTACTAGCCCAACCGGGCGCATCCGCCCGGTTCGGGACGTGGTGCGCCCCCAACTT

TTTGGAGTGCATCATGACTAAAGTGAACCATCTACAAAGCCTCTGTGTTATCCACGTAGA
CTTTGACATCTGGAGTGGACAAACCCGTTTGTCTGCATCTGATCTCAAGCTGGGCGAGGG
TGGTGAAATTCCACCTGAGAAAGTAGCTCAACTGGGAAGTAAGAAGATCTGTGATCCGGC
TAAGCTGAAAGGCTTTCATCGCCTGAAAACAGAAACTCGTCGCCTCCTGCTGAAATTCGG
TATGCCGTTCATGAACGGATTTGCCGTACCCGTCAGCAAGACCGATGAAATCTGTAACAA
GCTGAATGACATAAACTTTCAGTTTAACCAACTGAAACAGGATTTCATCAAAGGTTACAA
CAAAGCCGTGGATGAATGGTGTCAGGAGAACCCTGAGTATGAACGAGCTATCCGTGCCGG
AGCCCTTCCAAAGGAAACGGTCGAGGAGCGGATTGGCTTTGAGTACCAGGTGTTCATGAT
CCAGCCTGTGAACGAAGATGAGGCCAACGCCAAACGCCTTAACCGCAAGGTTGAGCGCTT
GGGTGACGATCTCATCTCCGAAGTGGTTCAGGAAGCGAATAAGTTCTATATGGAACGTTT
GGCCGGTCGAGACCAATGTGCGGTCACTACTCGGCAGACACTCCGTAACATCCGCGACAA
GGTGGATGGGCTTAGCTTCCTGAACAGCGCTTTTAACCCTCTGGTCAAGCTGCTCGACCA
AACCCTCCGGGGATACGAGCAACATGCCGATGGCCGAAACATCGTTGCGCCTTTCTTTTA
TCAGGTCGTGGCCGCAGTGCTGATCATGAGCGAGAGGGACCGCATCGAGCAGTATGCCAA
TGGCTCGATTACTGTAGAGGGCATGGCTAATGACATTGGCGGTTCGGGAGCCCAGATGGG
GGACCGTTCTAAAGATGAAAAGGCCGAACAGAAAAGCGATAAAGCCGGTGAGCTTATCCC
TGCAACAGAGGGTGGCGAAACCAAGCAGCAACAGGTAGGTGGTACTGAATCTGTTCAATC
AGAACAGACTAACAGCGGTGGTAACGCTGTTGACCTGGATGAAGACATCGACAACTTCTT
CAAGAGTTTTGCAGAACGAGGCGAGGGTGAATCGGAAGATGAATCCAATGCCGGTGATGT
GGTTCGAGAAGAGCGCGTTGATGTTGAGGATGAGCCGGTTTTGCCTGAGGAAACTCCGGT
AGAGCAAGAGCCTGTCCAAGAGGAGCCGACAGAGGAGCCTCTCAACCAGGAGCTGCCTAA
AACTGACGACGATGGCGACTATTTCTTCTAATAGTCACTTCAACTAAACCGACCCGGAGG
GGATAGCCATTCCCTCCAGGGGTGGACTATCTCCTTCCCTACCAGGAGAAAAGATATGTC
CAAAAAACGCACCATTTACAGCGCTCTACCAATCGTGGCCGCAGCCTATGGTGAAAAACT
CGGTGTCAAAGTCGCCATCGGTAACGATGACGCATACACCGATGGTAAGACCATCGTGGT
TCCGAATATCCCCGACGACTATCCTCACATGGATGCTGTCTGGGGGTATTTGGCCCATGA
AGCAGCCCATGTCCGGTTTACGGACTTTGGTGTTGAGCGCCGCAGAGGTCTTCATGCTGA
GTTGTCCAACGTTTTGGAGGACTGCCGCATAGAACGGGCCATGATGGAACTCTTCCCCGG
TACGTCGCAGACCCTGAATGAGGTTGCTCGCTATATGGCTCAAGCTGGTCATTACGAGCA
CGTCACAGACAAAGAGGCCCCTGCCTCCATTCTGACAGGGTTTTGTTTGTACTGGTTGCA
AACCAAGGCTGTAGGGCAATCCGTCCTTCAACCCTATCTCGATTCGGCTACCCCCGTATT
CGAGCGCGTGTTCCCTCAGGGTGTTGTTGTTCGGCTGAACGCTTTACTGCGTAAGGCTGT
GAACACTAAGTCAACCGCAGAGGTGACATCCTTGGCCGACCAAATCATCAAGATGATCGA
GGAGGAAAAGGAGAAAGAAGAGCAAAAGCCCCTGAATGGTCAGGATGGTAACAACCAGCA
GAATGCTGGTGGCAACCAACCTCAGAACAGTCAGGGCGGAAGTGGTAACGATCAAAACCA
AGGGCCTGATGCCAATGGTGGTGATCAGCAAGGTAAAGACCAGAAGCAAGACGATGCTAA
CGGGAAATCTGATCCGAAAGGACAGGGCGACCAAGGCAAGTCGGATACTGATGGTGGCAG
CAAGGCAGGACAAAGCCAGGCTGGTGGCAATTCTGACGCGGCGAAACAAGACGCGGCCAA
AATGCTCCAGCAGGTTCTGAATGCCGGTGCCGGTGATTTGCGTGGTGACGCGCATGATGC
ACTTAAAGCCGAGCTCAACCGGGTGGCTCAAGATAAGGGGGACAGTAGCTATATGACTGT
TCGCTCTGCTGTGAACACCCAGGACAACCCTGCTGTTGGCAAAAGCCTGGTAGGGGATGT
GAAGAGCACCACTTCAAAGATAAGAACGCAGCTCTACGGATTGGTCCAGGCCAGCCAGCG
AGTTGCTCACCGTAACCAACGATCAGGGAAGCGTGTGGATGCTCGGAAACTACATCGTGT
AGTGACGGGTGATACCCGCGTATTCCTCAAGCCGGAAGCCAAGAAACGCCCTAATACGGC
GGTTCACATCCTGGTTGATATGAGCTCCTCGATGGCCTACAAGGCCGCCAATGGAAAGGA
GCGTCAAGACATTGCGCGGGAAGCGTCCTTGGCTATTTCGATGGCTCTGGAAGCAATACC
CGGCGTAAACCCGGCAGTCACCTTTTTTGGTGGCAACCGGAACCAGCCAGTGTTCAGTGT
CGTGAAGCATGGAGATACGGTTCAGAATCGGGCCGGTCGGTTTGGGTTCAAAGCAACTGG
CGGTACGCCTATGGCGGAAGCTATGTGGTATGCAGCTTTTGAACTCACCAAGACCCGTGA
AGAGCGAAAAATGTTGATCGTAGTGACTGACGGGCAGCCTCAAAGCGCCCCGGCATGTCG
CTCAGTGATTGACCTCTGTGAACGAAGCGATGTTGAGGTGATCGGCATAGGGGTAGAGAC
TACCGCAGTGTCAGGACTGTTCCAAAAGAACATTGTCATTGATGATGCGGCAGCTCTGCA
ACGCACACTGTTTAAGTTGATGGAGCGGTCATTGACTGCTTTTGCAGCTTAACAGGAAGT
GAAACGACAAACGGCTATCCCTTCGGGGGTGGCCGTTTTTATTTGGAGAGTCTATGAACC

AATTCCATTCATCTTTGGATTTGTATCATCGGAACAAAGGTAGAAGGGCTACAGTGCCGG
AGACGCCTTTTTTACTGCTTGCTAAGCGCATTCCTCCGATGTACTGGAGACTGTTCCAGG
GTGTTACTTTGGATAGTCGTATGGGATACACAGGCAGGCGGCAGTTCCACAGTCTTGGGC
AAGCAATCGACTGGGCAAAGTCATCAGTTGGCGATTCCTGGTCAAATAAGCGCTTTCACA
AGCCGGTAGGCCTCGATGTATTGCTGGCCTGTACTGCGAGTAAGGTGCCTGAACATCTGG
TCGAAGAACTGAAAAGACGGGGCAGTTGATGCGTCTTTGAGCTTTGCACCCGAAAAGGGC
AGTTTGAGCGTTCCTGTTCACACAGCTCTCGTGGAACAATCAAAGTGAGGAGGGAATACC
CTCCCACTTTCTCAGAGGCCTTCATTGAGGGTTGCTGAGAAAGTGAATTTTGTCCTCTCT
GAGGGCGAAACGTGCGATAGCTGGTCGCCAAAAACAAACAGCATTCAACCTCAGCCTAAG
GGCGCACTGCGCCTGACAGGCTCTGTGTGCCCAAGTTACTTTAAGGAGCACACATGTCTC
ATCTGAACAATCTCAAATCCGTAATGATCTCTCTCGCCGCCGAACATAAGCTGCCTGAAA
TCTACCAGGATGACATCACTACCGATGTGGAGTCTCTGGATCGATTCGATGGTTTACGTC
TGGTCTGGCTGTTGCGGTCTTGCGGCAGCGTATTGGTGCCAGCGGAAGTTGGCGTTAATC
CGATCTATATCACCCATTGGTTGTGGTCTAACCACGGTCAACAGGTGGTTCCATTCTCTG
TGGATACCCGCACGGGGTTGATTGAAAAAATCGACTTCGAGCAAGCTGAAAAGCTGATCA
TGCAGATGCCTTGCAACCTCTCTTCATTGCAGAACAAGGAATACTTGGTTGACCAGGTAA
ACCGAGTGTTGCAACGAGGTTGTGAAATGCGTATCTGGGGTATCTTCGAGTCTCCAAGCT
CGGTGGAATCCGTTGGTGGTTGGAAAGAGTGGCAGAGCTATTTCAGCTCTACTGGGAATC
GACTGATGGCCGATTTCGTTGGTAAAGCCATTCGATTCACCAACCCTCGATAAATCGCCG
TTCAACCATTAACCCTAATTAACCCTATGGGGCTCCGATAGCCCTGAGGGGACGGGGCTC
CGCAATCGCAATAAGGAGTCCCTATGAAAAACCTATCAGCTTTGGAAGCAGTTCTTGACT
ACGACAAACCTTCTCGACGTTTTCTTGATGAGCTCAACGAAAACCAGATGAAGGATTTGT
CCGGGGAAATCTTCGCCAAGCTCTACTGGAGCAAGCGTAATCCCCAGTGGTACGAGAAAG
ACACCAATCGGCTGTTTGCACGACTTCGTTGGGTTCAGCGCATCATCAAGAAGCGCTTGA
AAACCGGCAAGGTTAAACCTGAACTGACAGAAAATGGTTCAGTGATGGAGCGCTTCAACT
TCCCTTATGGTGACACTCTCGATTTCTTCCATCGGTACTTGCGACATCCCAAATGGGAGG
TTGTGTATCAAGAATCGGGGTGCAGTGCCTTTTGGAAAAATGAAGCAACGCTTGAGCTAT
GCACCTACTGCGAAGGTGATGTGGTCATGATGAAGGCTCCTGATGAAGCAACCTTCTTTC
GTGATTGCAATCGCCTTAGCTGGTGGTATGCAGATAACGCTTGATCTTAATCCCTAACCA
GGGGCGAACCGGCCCCTGATGGGAGCATGGTGCGCCCCATCTATGATGGAGGACGCCATG
TTCAAATCATCAGTATGCTCCTACGAAAACAGGGGGCCTTACGGAAACAACAAGTACCGA
GGCAACTGCTCGGGCTTTATCGTTAAAGACTTCATTGAGTCTTACATGAGAAAGCCGAAC
GGGCTGGTGGCAGACCCAAGTGTTGGGGGTGGCTCTAGCATCGACGTTGCCAATGAACTT
GGTGTCCGTTTCAAAGGGACAGACTTGCATCAGGGGTTCAACCTGTTGCGGGATGACTTC
CTTTCGTTCCTCGGAGAGCCTGCACACCTGATCTGGTGGCACCCTCCTTACTGGGACATG
ATCCAGTATTCAGGTAAGCAGTGGGGCGAGCCCAACAAATGGGATATGAGCCGCATGAAT
TTGCCTGAGTTCGTTGAAGCCCTTGAGCTGGCCGTTATGAATATTCATGACGCCTGTGAG
CGAGGAGGGCATTACGGAATCCTCATGGGAAACCTGAGGCGCGATGGGGATTATTTCAAC
CTGTCCAGCCTTGTGGAACGTATCGCACCTGGGAAGCTGGTGGATGAGATCATCAAAACA
CAGCACAACTGTGTGAGTGACCGAACTCAGTATTCCGGGAAGCTGGTGCGTATCGCCCAT
GAAAAGCTGTTGGTGTTTCGTCGTAACGACGTTGCCTCATCGCTCTGTCTTCTTGCTGCC
GTACACCGTCGAGCAACCAATATGGTTTCGACTACGTGGAAAGCTGCAATACGCCGAACC
CTACAGGGGAAAACGTTGAAGCTGGAGCAGATTTACAAAGAGATTGAACCCTACGCAAAG
CATCGAGAGAACAACCACTGGCAAGCAAAGGTCAGGCAAGTTCTTCAAGATGCCAGGTTC
TTTATTCGCATCGAGGTAGGTGTCTATGCACTTGCTGAGTAGCCCAACCGGGGCGTGACA
TCCCGGAGGGGATTGGTCGCGCTCATCAACAACAAAGGAGCGTGACCATGAACGTACAAC
TTCAAGCCAGCAACAATGTGCTGTTGGCGCAAATCGAAAGCAAAGGCCTCACTGTTGAAA
CGCATTGTCGCAGTGGCTTTTGTGGCATGTGCCGGGTTCGTCTACTCGAAGGTCAAGTGG
CCTATGACGAGACGCCCATCGCATTTGTCAAAGAAGGGGAAGTGTTGGTTTGCTGCGCTA
AAGCAAAAACCGATGTGACCTTGGAAATTTAACCCATCCGGAGGAGCCTTTCCTGCGGGT
GGGGCTCCTCTATCCATTACGAGGAGAAACACTATGTCTATCAATACCAAAGTTGAACAA
ATCGCATACGGTCATGCTACCGCTCTAGTGCTCAGTGAACTGGGCCAACAAGAGAATTGG
TGCAAGGCTTATGAGTATTTGTCTGAGTGTGTCGAACGGGGAGACGAGCCTGAGGATCTG
GTCGTTTGGCAACCGTTTGAGCACTGGGAATGGAAAGACATTCTGGAGCAAATCGAGAGC

GAAGCCGAGTCTCTGCTTTCGACTATTAAGTCCGTTTTAGGCTTGGCCCACAAAGGCATC
ATTCAGTCAGCAATTGACTGCTCTCTGGATTCGGACATGACCCAGCTTGACCTGATTGGA
ATGGTCGAGCTCGGAAGTGAAATCGAAGATGGGGAGTGTGCTGGAGGTGGCTATGCAGCT
TAACCGATACACGGCCAGGGAGTCTGACAAAAGTCGGATACTTCGGACCATCGGCTGGTG
CAAGCGCAATCACCTTACACTTGCCGGTCTTCCATACGAGGACAATCTTGCAGGAAGTGA
CGGGATCAGCATAGAGATCATCACTCCTCCTGGTATGTCGCGGGAAATGTTGGAGCAAGC
CGTCAGAGAGGGGTATTCAGAGCGAGATGTCGTAAGGCATCGCATTCTGGAGTGCCCTGT
CGGTTGGTTCATGGAAGCTGATGGCAAGGCATTTGATCATGAAGTGTTCCACGATTACGT
GGTGGCTCATGGTTATGGCGAACCTTCCAGCGAAGCCTATGAGCTGGCGGAGCGGTGGTT
CTGGCAGGGCAACGATTATGCGTTGATAGCTGCGGAAATAGTTGCTCGTGATCTTTGTGT
TCGTGATGACGAAGATGAGGACTGATCCTATGGATGCTGTAGCGCATATAGCAGCAGATA
ACCTTGTACCGGCATGTGGAGGTGCAGAGAAGCCGTTTACCGTTAGTGGTAGACAGTGGC
TTTACTGCTATCACCCAGCCAGTGGGAGGCACTGCTACTTAGACCTCGATAATGACCGTC
CAGTCTGGCATCGAGGATTTCATCCTGCTTTTCATCCTGAGCTTGAGTTCGCTGATGAAG
CTGAGTTGCTTCGACCAGTTGAGCGAAAGCCTGACCATGAAGAGCTGGAGGATTTTTACT
TTTAACCCACGGGGCCGCTCTTCCTGATGGGAGACGGTCCTCCATTCTCAAAGGAGAAGA
CCATGAAAGATCACAGTCAAACGATTGTGTTCCCTGGGAACAATGTTGAATCACTTGCTG
AGGCTAACGCCATGTTGAGCGCTGTGTCGGAAGATGCACGTAAAGCCAGCAATACGGAAG
ACAAACGTGACCTTGAATCGCTTCAAGGCTGGCTGGAAGAGAACATCAATTCTCAACTGG
CAGGCGTGAAGTAACTATGACTGTAACCCCACTGGGGGCTTCATGCCCCTGTGGGGGCAT
GGTGCCCGTAAAAGGAGTCACCATGAATGCAGTGAAGATGATCCAGAAGGAAAACCAGCT
TGAGCTGCCGCTTTTCTTCCTGGATGAAGAGCCGAAGACCGCAGAAGTCATTCCATTTGA
GCCAAAGCCTGAATGGACTGATGATGAGGTAAGGCAGTTACGTGATGGGCTTTTATGGCA
TAGCCTTAGAGTCCTTGCTGATGGTCGAGCTGGAAGTGAAATCAAGCAGGAAACGATGGC
TTGGGTGATGTCCGATGAGGTACATCCATTCTCATTTGTGGTCTGCTGCGATGAAGCAGG
TTACGATCCATCTGGAGTAAGGGAAGGCGTGAAGTCTATCCTAAACCGCTTAGCGCGGGT
TAAAGCGGGGGGTTAACCCCCTGCTTTCCCAGTGGCATCGACTTTCACATGTGAAAGTTT
GTGCCCCTCGGAAAGCAAGATTTTGCTCTCTGTCGAGAGTGAATAGTGAGGTAGCTGGCC
TCCCCAAACAAACAGCATTCTACATAACCCTTGGGGGATCACTGCCCCTCGGGCTGGTGG
TTCCCTGACAAACATGGAGCCACAACTATGACTACTCTTGCCAGCTTTGCGAATCCGATT
TCTGTTATTCAACGCTCACCAACCAGTGGGGTGTTTGGCACAAATCAGTTCGTAGGAGAC
AAAGAGGGATTTGTACAAAAGGTGCTCGGAAAGTGGTACTGCAATCCAGGGAATACCATT
CCCGTTGAGAGCATGTCCCACCTTGAGCAGGCGGAGAAACTGTATGACTTCTATGTCAAG
TATCTCCAAGTGAAAGAAAAGATCTCTGTGAACCTTGGCCGGGTGCTGCATAAGCTGTAC
AGCACTGGTTCTATCAAGGAAGCAGAGATCGTGCTTGAAGCAATAAAGGCTCAGGCTGGC
GTAACAGCTTGAGCAATCTACTAACCCTAAACCGGGTGCATCCGCCCGGTTCGGGACGTG
GTGCGCCCCCAAACTATTTGGAGTGCATCATGGAAAAACATAACCTCAAATCCGGATTCA
GCATCTATTTCGCTGACGTCCATTTCGAGAAACAAGTCTACGCATTCGGCTCAGGACTTG
GCTTCACCTCTGTGATTTACGCCTACTCGCTGGGCCGAGATCCGGAAGAAGCTGAGAAGC
TGGCGCTTGAAAAGTACGACTCTGACGAAACGAAGGTGAAGAAGGTGCATGTCAATCTGG
CTCGCAGCCAGGACATCAACCGCTACACCTTCCCTGAACAAATGGCTGGCTTTGCTAATG
CCATTCAGTCTCACGGCATCGCTGTGAACTGAGAACCACTTTAAACCCATCGGGGCCATT
ACTTCCCGATTGGGACGGTAGTGGCCTCTCAACCGAAAGGAGAGAGCTCATGGCTACTAT
CGTAAACACCAAGTTAGGAGAACATCGAGGCAAGAAGCGCGTCTGGCTGGAAGGCCAAAA
GCTACTGCGTGAAGGTTACTATCCTGGCATGAAGTATGATCTGGAGCTGAAAGATTCCCA
GGTTGTGCTTCGCGTCAAAGAGGAGGGTAAGTTCACCATCAGTAAGCGCGAGCGCAATGG
CCGGGTGTCTCCAATCATCGATCTGACCGTGCAGGAGCTTGCTACCGTTTTTGACGGTGT
AGAGATGCTCCGCGTGTTCATCCGTAACGGCGCAATTGTGATCTCTGCTCACCATCAACA
AGAGCGAGTGATCGAGCGCGTCAACCGGCTTATCAGTAAGCTGGAGAATGGAGAATCGCT
TTCGGTATGCAGCCTCTTTCATGGGGGTGGTGTGCTTGATAAAGCGATTCACGCCGGTTT
TCACAAGGCGGGAATTGCCAGTGCCATATCTGTGGCCGTGGAGATGGAAGGGAAATATCT
CGATTCGTCTTTGGCAAATAACCCGGAGCTTTGGAACGAAGATTCTATTGTTATCGAATC
GCCAATCCAGGCTGTGAATCTCAGCAAACGCCCACCACAGGTGGATGTTTTGATGGGGGG
CATACCGTGTACCGGCGCGTCAAAGTCAGGTCGCAGTAAGAACAAGTTGGAGTTTGCCGA

ATCGCATGAGGCGGCAGGTGCCATGTTCTTCAACTTCCTGCAATTCGTAGAAGCGCTAAA
CCCAGCCGTTGTGCTGATTGAGAACGTGCCTGAGTACCAGAACACCGCTTCGATGGAAGT
GATTCGTTCGGTGCTCTCTTCGTTGGGTTACTCCCTACAAGAGCGCATTCTCGACGGCAA
TGAGTTTGGGGTTATTGAGCGCCGCAAGCGTCTTTGTGTTGTTGCGCTTTCCCACGGGAT
CGACGGGTTTGAACTTGAGAAGGTTCAGCCTGTTCGCACCAAGGAAAGTCGCATACAGGA
CATCCTGGAGCCAGTTCCGCTCGATTCTGAACGTTGGAAGTCATTTGACTATCTGGCTGA
GAAGGAGTTGAGAGACAAAGCTGCTGGCAAGGGGTTCTCTCGCCAGCTTCTGACTGGCGA
CGATGAGTTTTGCGGCACCATAGGTAAGGACTATGCAAAATGCAGAAGTACCGAACCTTT
CATTGTTCATCCAGAACAGCCGGAGTTGTCTCGCATCTTTACACCGACAGAACATTGTCG
AGTGAAAGGGATACCAGAGGAACTCATCCAAGGTCTGTCGGACACTATTGCCCACCAGAT
TCTCGGGCAATCGGTAGTCTTTCCCGCGTTCGAGGCTTTAGCCCTCGCATTAGGGAACAG
CCTGTGGAGCTGGGTTGGAATGATGCCAATCATGGTCGAAGTCGTGGATGAATCACAGCC
GGTGATCGGTGGTGAAGACTTCCATTGGGCAACGGCATTGGTTGACGCAAAGGGCACTCT
CAAGCTGTCACCGGCAGCGAAAAAACAGGGGATGCCCTTCAACATTATGGATGGTCAATT
GGCTGTCTATTCACCTAACGGAACTAAAAAGAGCTGCGGCCATGAGCCTTGCGAATATCT
CCCGGTAATGATGTCCGGAGACGCAATCATGGTCACTTCATCTTTGGTTCATTAGTAACT
CACCGGGGCAATGCCGAAAGGCCTTTGCTCCGGCCTATCCGAAAGGAGAGTAATCATGAA
AATCGAAACTGTTGTGCCTTTACCTCCCGAGGATTCAGGCCTTCAACACTGCATTGCCAG
GTTCCATAACCGCAACATGGATTCCAAGCGAAAAGACAAGACCCGTTTTTTCAGGAGGGA
GCCGGTTATGATCGTAAACCCAGAGACCAAAGCAAAGGTTCTCCGGTATGCGATGGGCAA
CCCTGGGAATTTATCCATCACAAAGCTGGCGGTCGCGCTGGATTACGATGCGGTAGATGC
TCTGGGTGTTCGTTTTAAAGACACTGTAAATCTTGAGGTTCGCAGAGCCAGGCGCTGGGA
AGTCTGGCAGTGGTTCTGGAACCATCCGGATCAAAGTGTCCAACTGTCAATCAAATTGGG
CGTGGTTGGCGCTGTGTTGGGAGTCATGGGCTTCCTGACTGGCGTAGCTCCGTACCTCTT
GGGATAAACCAATAAAGCCGTTAACGGGCAATGTAAGTCCGGAGACGGCTTGCATTGCTC
TCTCGAAAGTGGAGAGTCAAATGAACACATTAGAACAACTTGTAAATCAGAGAGTGCTTG
AGGAAGGTCTGAAAAAGGCGAAGCCATTGGCCGCGCAAATGGGGCTTGAATCTGTACCTG
ATGAGATGCTTGAAATGATCTGCCCAAACTTGAACTACTCTGGGCAGTTTACGCCAGCTC
TGTACGCGAGATTTCAAAGCGTAAAGCGATTACTGAAAGAGTTGTCGGAACAAACCTCAC
CTAAGCACGGGCATATTGTGAAGTTTACCAACCAATATGGGAGCTACGATGAGTCAGCTT
TGCTGTTCAAAGAAGGCGACGTCAGAAACCAGAACTTGGTTGCTTGTGTGGGCGGGAAGA
CGCTAAATCTCGGAGAGCTAGAGCTTCGAGATTATGGGCTCAATATGCACATCGGTTGCG
GTGGACCATTTGAGCCGATCTCAGAAGCGCAGATTCAGTCCTTGTCTGAGTTTGACTTCT
ATGAGACGACCTACAGATTCTGGGGCCAGAATCCATGTGGAAACGGGATGATAGACATCA
CCGTCCCTATGAAACGTTGGTTCTTGACTGTGTAGCTTCAACCTGAGTGCGCCGAGAAAT
CGGCGTACCGGCCCCTTAAAAAGGGTAATCAGCGCAGGGGGAAACCCCGCGCTTTCCCAA
GCATCTCGGTTTCCCAGGGTGCTTCGGAAAGTGCCTTAGGCGCTCAAGTGCGGTAGCTGG
CTGCCAAAAACAAACAGCAAATTATCAACCCTGACGGGAGCACCTCCTGTTAGGGGGGCT
CCGTCATAAACTGAACGGAGATATAGCATGTCTAAAAACGTCAACGATGTTTTTTCAAAC
CGAACTTCTTTGGAATGTACTGTAAATGCAGCGCTGGATCGTCGTATCGGTCTGCGTGTC
GGGTTCCCTCATTACAGAGAAGGAGCAAACGGTGTGGTTTGTGAGATCGAAGCAGAGTAC
CCATTGCTCAACACTCATTCGAGTGTTGCATGGCCCCTGCTCGATGAACTCAACAGTTAC
ACCATCCAAAAAAACCAATCGCTCATTGATCACATTGATAATGCGGTCGCTGAACTGTTG
AAACACAAGGCTCGTCTACAGCTTGATATTCAAAAGCTGCAAGACGGTGACATTGATCTG
GTGAAACAGGCCAAGCAGCAATACGAAGACAACCAGATGTCGAGTAGCAGCTAGTTCAGG
TCTGATAGCCAGGATTCAATTTTAACCCTACCGGGGTGCTTTCCCGGAAGGGATGGGCAC
CCTGCTAATCCAGGAGGCCCTATGCAACAAACATTAAACTTTGATCTATCAGAACAACCC
ATCCCAGTGCTTTTGCGTGGGGATAAGTGGGATTCTGTTTGGTCAGAACTGACTGATAAC
GAGGATTTGAACTTCGTTGATGCCAGTAGAGGAACCAGTCTGTCATCGTTGATCACGTCA
TCGGTTGAGTCCATTCACACGGCCTTAATTGATGGGTGGACCATGATGATCGGCTATTCC
AGTGGTAAAGATTCTGAAACGGTCTTGCACCTGTTCCTGATGGCCCTGATCCGGGCTGTA
AGGAGTGGGGCGACCATCAGCCAGCACCACTTCATCTTGCACACTGACACACTGATCGAA
AGCCCAGAGGTAAGGTGGCTGGCGGATCAGAAACTGGCCGAGCTGGAGCGTTTCATTGCG
AAGGAAAACCTTCCTCTGACCATCGTTCTAGCAAAGCCTGGGATCTCGCAGAGTTGGACG

GGCCGAATCCTTACTGGGAGGGGGTTGCCTACGTTTTCCAACTCCACGGCCCGCCAGTGT
AGCCAGGATTTAAAAATCACCTCTGCCAAAAGAGCGAAGGCAGCTTATATGCGAGAGCTT
CCCAAAGAGGTTCGACAGAAAGTCTGCCTTTTGCTGGGCAGTCGTGATGCTGAAAGCACT
ATTCGTGCGGCCAACATTGCAAAACAGCGAGGAAGCTCAGATCGCGTCATTAAGACGAAA
GATGGGGGTGAGCTGTATGTGGTGAAAAACTGGTTGGCGAGTGATGTCTGGGAGTTCTTA
CTATCCGCTGGCATGGGCTCAGCATATCCTCTGCCAAGTTACTTGGAGAGCAATGTCACT
ACGGCAGAGCTTTACAAGGCGGCAACAGGGGAGTGCGTGTGGTCGGCAACAGAGAAGAAG
GCCAGTGACGCCTGTGGCGCTCGCTTCGGTTGCTGGGCTTGCCAGGCTGTTGGGTTGGAT
AAGTCAATGGAGACGTTGCTTGCCACGGACCCTGAAAGGCATGGCTATATGTCGGGGCTG
AACCGCATCCAGCGCTACTTGGCAAAACGCCGGTACGCATGGGAAGACCGCCATCCCGTA
GGTCGAACGATTTACGAGGGCGGATACATCAAAATACAGCCGGACGTTTACAGTCCTGTT
TTCCTAGAACGATTGCTTCATGTCTGTTGCAGCATGGACTACATGGAGCAAAAGAGAGCT
GATGAGCTGGCATACAAGCTGGCTACTGGTCAAGCAGAGGATAACGACTGGAACCGTCGA
ATGGCAGAGCCACAATTCCGGATCATATCGGAAGAGGCTTTAGTCCATATCGACTTCATG
TGGGCCTTCCATCATTTCAATGATAAGCCTTTCCATGCCCTGGAGATTTACCACAGGGTT
TGGTCGATGGGTGATCTGGACTTGCTGGAAGACGAGCCGCAGTGTGAAACCGTTCCTCAG
TCACCAATTCCGAAGCCATTGTGGTTGAAGGTTGGTCGCTGGGGCGATGGTTCGCTTTCT
GATGGCTTGGCCGATCCGTTAGCGGAAATGGCTTACTTTGACGGCGGGGATGACCCGTTG
GCAGCGCAAGTGATCAATACCGCAGACGGAAAGAGAAGGGTGGTTTGCTTCGCAGAAGAT
GATGAAGTGAAAGTCGATCCCGACTCCGCTGCGTTCATCATCTGGAACGAATACCCGCGA
CTAAGGGAGTCTGTACTGAAGGGACATTATACGCCCGGTAGTGCAGCGCAGTTCTACCTT
CGGTTCGGAGCAATCCAGCTTGCGAAAGGAAAAGGCGCTTTGTATCACCGTATGATGCAA
AGAGGTCAAACCTACCACCAGATGGGCCTTACAGGGCTACAGACGATGGAAGGAATCCAA
CAGCGCAAGGATGTCAAAGTGCTTAGCGATGCTAAGTACAAAGATCTGGTGAAGCGAAAA
ATCAAGGGAAGACTGGCAACGGTACGCTGGTGGGTCAACTTGCACTTAACGTTCAAGTAC
CATCTGCACCACCGTACTCCGACGGGGTTGTTCATCGAGAAGCAACTTGACCAGGAAGCA
ATGGAAGAACAGAAACGGCATCAGGAGCGATGGTTTAACTACGTGACTGACGCGATGCTT
TGTTACTCCAGCGCATTCTGTATGAGCGTCATGGAAGGGCGAGAGGGGAGCGGAAACGCC
AACATTCGTCGTTACATGGCCGCGACCAGAAGGAAGGCTTATACGGCCCTCTGCGAGCTC
TTGGACAATACTGACGCCCAGTGGGTGAATGATGTGGTCCAGAGTGCCGTAGGGCAGTAT
GAGGCGATACAGGCAGCCCTAACCGAAGGTTCCGCGCTTGCTATCTATCTCGACTGGATC
AACCTACTGTCAAAACGCCATCCAGCCTCTTTGGAGCGACATGTCAGAACGATGATCAAG
GCGGTTCAACGCTTACATCGCCGCGACGACACAGAGTTGCAAAGAGGACAGCAAGGCTTG
TCCTTGGCCGCATAAAAAGCCCTCCTTCGGGAGGGCTTTTTCTTTTGCACCCCAAAAGGG
CAATTACAGCGTTTTACCCTTTTAGGTGGCTGTCATACTCTTTGTTCATTGTCAGCGCTT
GCTGGCTATAACGGTAGCTGGCCCAAAACAGCAATAACCTAACCCCTCGGGAGGAAACCC
CTCCTGGGTTCTCTCCCACAAACAAAGGAGAGAAGCATGAGCCTAGACAAGTTAAGTCGG
CTGGTGGACCAACAACGAAAGACCCAGGATGAAATCGACAATGAAATCATTCTGGCCGTC
AAAGAGGTGTTGGCTACAAACTCAGTCGGTTTAGCCCGTGAGCTTGTTGGGGGTGTCCCG
CAAGATCATCCATTCCATCCGTTTTTACTCGCTATAGCTAAGCAGCTTGACCCGAGATAA
CTAAGTAACCCAACCGGGTACACCAAGGCCCGGTCGGCTTTGGTGTACCTCCAACCAATT
TTGGAGGCACTATGTCTAATTCAAATTTCGGCTTTCTAGCTCTGGCCTTGCGCCAACGCC
TGATCAAACGCTGGTCACTGATGCACTCTGTTCAACCGGAGTCTGTGTTGGAACACAGCG
CTGTTGTTACCCTGCTTTCCTACCTGGCTGGCAATATTGCCATTCAGCAAGGGAAGTCGG
TGGATCTGGCAGTCATGCTTGCTCATGCTTCGTTGCATGATGCAGCCGAGGTTCTCTGTT
CGGATGTTGTAACACCGGTCAAGAAAGCAAACGCTGTTTTGCAGCGTGAGTTTGAACGAC
TGGAAAAGGCCGCAGAGGACAAACTCATCCAGACTCTTCCCGAAGAACTCCAGGATGCAG
TCGCCATAGCCTTCGCTCCCGGTGGCTATGAACAATCACTGGTTAAGGCCTGTGATACCT
ACTCCGCTTACATCAAGTGCAAACTTGAGGTGGCCGCAGGTAATGGCCTTGAGTTCCAGG
ATGCTCTCAGCAAAATGGAACGTGTTGTTGCTCAGGTGAAATCGGACTTCCCGGAAATCG
ACGCTCTGGACAAATGGTTTGGTAACGGACTCGGCCATTCAGTAGATAAACTGCTGGCAG
GGGGTAACGATGACTAATCCAATCCCTGGCGACATCAAGATTAAGGACTTTGGCCGCGAC
CGGAAATTCCGTTCGGTTGATGAGCTTCAAAGCACTTTGTCAGAACAGTACAAAGGTCAG
CATGTCAGCATCGTTTACCCTGCAAAGCCCAGCGGTTTACTCCGCACGGTTTTTGTCAGT

GTCGATGATGCTGGTGGCGTGAATCGAACCTATGGGGACCAGTCTCCTGTCGATTTTTCT
GCCATCAAAGATGACCTGTATGTACCAAGTGACCTGTAGGAGAGCACTAGCTATGGTGGT
GATCATCGTAAACACAGGACACTATGAGTTTATCGGCCTTGGTGAGACCCACGGACAAGC
CACAGAAGGGCTTTTGAAACGCTGGGACGAACACTGCGAACGTAATCCGGATGCTGAGTC
TGGTTACATGCAGGAGCTGATAGAAGAAGGAAGCGCACAAGTTGTCGAGATGGAGCCCGG
CTCCGCCGTAATTTACGGACTTGACGGCTAAGCCTTCTCAACTAACCCACTCGGGCGCAT
CATCCCGAGAGGGTGTGGTGCGTCCTAACCAAAAGGAATGCACTATGTCTAACTTTGAAC
AAGCTCTGGAGCGTACTGACGGTAAAACACTGATTCTCAGCAATGGGAGTAAGTGGGCAG
GCCAAGACCCGGACAGCATTCAGACTTTACTGGATGTCCTGGGTGACAACGTTCTTGACC
CGATGTTTGAGCAGTATCACTGTTATCGCCCATACCCGTTTGAACCAATGGTGAGGACGG
GGCGAAATGGCGAAATGTTCCAGCCTTGGCTTGGGGCAGCCTGCTTCTTTGGGAACTTCC
TGACCGTCTCTCATGTTTTCAACATCATCACGAAAGATGACGGTGTTGTTGAGGCCTTAA
CAGAGGCGATCCGGAAGAATATGGCGACTGAACAGTACCAGCAGAATGCCTATGAGCGTT
ATGCCGGTTGGTTCTACGCTGAAACCAGCGAAGGGTTAAGGCTAGTCTCTCCAAGTGAGG
CTGCCGACATCCGAGCTGGCGCAGTTTCAAAGCTGCGTTACCCAAGAAACTTCGAGGTTA
TGAAAACTGCGGTTCTCAAAGGTCCTCGATTTGATACTGAATTAAGCCGCAAGGCTTCTT
AACCCAAACATTAACCCCACGGGGGACATCGGTCCCTGAGGGGATGGTGTCTCCCTCTGA
AATTAAGGAGATACCAATGATTAAACAGCATTTTCAAAACGAACTGGTTAAGTGTGGATA
CCCGGATGATCTGACGATTGAGTACAGTCTCGGATACTGCCAGGGGGATGGCGTAGCCTT
CTACGGGGATTTGAGCGTTGATGACGTCAAAGCTCTCATGAATCGCCTATTCAGCACTGA
GCCCGGCCAAGTGGATGCTGTCAGCCGCGTGAAGAACCTGATGGCACAGAAAGACATTGA
GAATATGCTTTCTGTCCTCCGCGAATATGGTTCCTGTGACCTGTCCATTACTCGGAATAG
TCACGGGCATCACTACAGCCATTGGAACTGCATGAACATCGACGACAACGTGGACTTCAC
AGGGATCTTCCCTGATGATGATTCCATGATTGGCACCGGCATTGAAGGGATTAACCAGGA
TATGGTCGAGCGCTGGCAAGACCTCTGGGAACGCTTTGTGCTGGAGCTGGCGGATGATGT
AAAAAGTCTTTCCAAGAAGCTCGAAGCGGACGGGTACTCGCTGATCGAGGCCTCTCCATG
CGAAGATGAAGTGGTTTGGGAACGGGCCACTGAAAACTACCTGGTGCGTGTTACTGAACT
CCCTGAGCGGGATTTCGATATGGGCCACTGGGATGACGAAGTAAGAGACCAAACAATTTG
TTCTATCCTGGAAGGGAAAGAGCGAGTGCTTGGCCTACGTGTTGAGGTACTCTCTCGTGA
AAACGAGATTGTCCTGGGTGAAGAGAGTCTGCACGGCTTGACCGTTGCCAGTGATGACAA
GAGCTACGCTGGCTACAGACGAGAACTTCTCCGGGGCGCTATCCAGCAAACCAGGGACTT
TTTCTCTCGCCACCTCAAAGCGGCATAACAAGGCAGGGGGAAACCCCTGCTTTACTTCCC
AAGTCTTCTTACTCATGGTTAAGCCATCAGTAAGCAGATTTTGTCCTCCCTGAGGGCGAA
AGCGTGCGATAGCTGGTCGCCAAAAACAAACAGCAAATTAACGTTAATTTATTAATCCCT
GCGGGAGTCTATCTCCTGCTGGAGATACCTCCCGTTATTTCTGGAGGTATCCATGAACAA
ATTCCAAGACTTTAAGTTGGTTTATCGTCAAGGTGGTCGTCGCTTAGAGCGTGTCTTTAA
GGACACTCTTTACGCGAATGTGAAGCGTGTCGCTGACTCGTTTCCTCCGACCGTTCTTTG
GCGCATTCAGCCAGCGTAAAACTCGGAACCTTAAAGCTCGGCTTTGGCCGTGACCTTTCA
ATAACCCATGCGGGTACACTTCCCCGCACGGGGTAGGTGTACCCATTAACTTTGGAGTAC
ACCTTATGAACCAAGTAGCTAAATTGGACCTCGCCCAAATCCGTCAACAAGCCATCAATG
ATGGTCTGCTGGTGGACCAATCTTCCATCGGTAAACAAGCTGGTTTCCTGACCAATGTGG
CAGTGACCCCGGCAATTGTCGATGGAGTATTTGGTGCAGATGGTAAGCACTCGGTAGAAG
ACTTCCTTTTCATGTTTTTGCAGCTTTGTGTTGCTCAAACAAAGGTCGCTTTTACCGACA
ACAAGAATTGGGGAAAGATTCGTCTTTATTACCCCATGCCCACTGTAGATGGCTTTTTCA
AACCCACTGAGGTTGTGATTAAGTCAGATCCTGTAACCGCAGATGTCACAATCATGATGG
CCTCGGAAGATGGCACTCATTTGTGCTTGTGATATTCACAAAATTCGCCTCCGTTTCGGG
GGCGACCTTTTTAAACCCCTCGCCGGGGAGTTCTCCCCGGCTGGGGAGCCTCCTTGGCAC
ATTCTCCCGGTGTCGGGGTTTCCTTAAAACCCTGGCGACGGGGGTTTAAGGAAGCCTCGA
TGCTGGGGTTCACGCTAAGGAGAAAACCTATGTCTTACTACAAAGCTGACACCGTTCGTG
AAGCTGCAAACGGAAACTGGCTATTCATCCTGGCGGCCCTAGCGCCCCATCTTGAACCAG
CTCTCCGTAAACCTGGTCGTCATGTTTCTTGTCCTATCCACGGCGGGAAAGATGGCTTCC
GACTGTTCAAGGATGCCCACCTGACTGGTGGCGGCGTATGCAATACATGCGGTGCCAATC
ATGATGGTTTTGAGCTGCTGATGTGGCTCAATAACTGGGACTTTAAACAGTGCCTGAGCG
AGGTTGGTGATTATCTGGGCGTTGAGAAAGAGCAACCCCAGTATCAACAAGCCGCTGCAC

CGACACGAGCTCCTGTCCAGGCCAAAGCGCCTGTTCAGCAAGAGCCTATGAAGGTGAATA
ACAAGGTTCTCGATTCCAAGAATCGTAAAAAATCCATTGCCGGTACTCTGATTGCCCACG
GTAAAGCTCCTTATGAGCATAACGAAGACAACGAGCTCAGCTACTTTGCCTTCATCCGTG
ACAAGAGCGGTTTGGAACGCACCATCTGGGGCGTGGATCTTGAAAGAGCCATTGGTGAAA
GTGAAGCCAAGTATGGGGATGAGATCGTCATGACAAACCTCGGGCGCGAGCCTGTAACTG
TCGTCGTTGAAGTTAAGGACGAGCAGGGGAATGTTGTGAGAGAACAACCTATGCAAACGC
ATCGCAACACCTGGCTGGTGGAACGTCGCGGCGCTACGGTAACGCAGTTCCGCGCTCGCT
CGAACGGTGGTGTTGAGCCGGTGAGTCACCATGTTGAGTCGGCCCCTGTGGTCAACCGCA
CGGTAGAAGCTCCGGCACCGCAAGTACAACATGCGGCCACACAACCGGAAGAGCAAAGCA
GCGAAAACAAGCCGAAAGTTGTTCCGATGTTTCGTGAACAACCTAAGCCTTGGCTGCTTG
AGCTCCAAGAAGAAATGGAGAAGAGAATGGAGCGCGAACGCGCTTACAGTGCTCGTCTCC
GTGAGAAAATCGAGAAGGTATGGAACGAGTGTTTGCCGTTCTCCAGTCATGTGACTGAGC
CAATGCGTCTGTACTTCAAAAACCGCGAGCTTCTGTTCAAGGTTGATGAAGTAGAAAAAA
CAGACTGTCTGCGGTTCAATCCGGCTATGGCCTACTACGACGAAGATGGCAATGAAGTTG
GGAAATTCCCGGCTATCGTCTGCGCTATCCGAGATGTGGAAGGCAACCTGGTAACGCTCC
ACCGCACCTATCTCACCCAAAACGGTAAAAAAGCCAAGGTCGGCAACGCCAAGAAGATGA
TGCCCATTCCTGACGGTTTGGATGTCAATGGCGCGGCCATCCGCCTCGGTGAACCGACTG
AGGGTATCCTGGGTGTTGCAGAAGGGCTGGAAACAGCCCTGTCAGCTTATCGAGTCACTC
AAATCCCGGTTTGGTCAACGGTCAATGCGACCCTGATGGAGTCCTTCGAGGTTCCAGAAG
GTGTTCACACCGTACTGATCTGGGCTGACAAAGATAAGTCTGTGACTGGTGAGAAGTCAG
CGAACGTGCTGAAAGCCAAGCTGGAGAAGCGCGGCATTCGTGTGTACGTCCTGCTGCCTA
AACTCCCGATCCCGCCCAGAGCGAAAGGGATTGACTGGAACGATGTCCTGATGAGTCAGG
GAAGCCTCGGTTTCCCGAATGCTCGCTACCTGCGCGATTTCATTGCGAGAAGGAGAGCTG
AGTATGGCCGTCATTGATGTCTCGAAGGTTGATACAACGCCTGGTAACGACGCGGTGTGC
CCCTTTTCTCCCCCTGAGGGGTGGGAGGGGGACTCTGCGGCCTACGTTGAGCTTATGCGG
TCTCGGTATCGTCATCTGATGCACGGCCAGAGAATGATGGTGACAGCCTCCTTCGCAAGA
AGGGAGCCTATCCAAGTTACTGGCCCGTTTGCTGATGAAGCGACGAAGATCATTAACTCA
ATGAAGATGAACAAGGCGAAGCCAACAGCTTTGTCTGCCTAAAACTTTAACCCTGAGGGG
GTGTTCATGCCCTCTCGGGCATGATTCGCCTCCTCTTCAACTATGGAGGTAATCATGAAA
AAGTTTTTGCGTATTAAGACGTGGTTTGTGCGTCTTTTCTCTCCTGACAAGAAGACTCTG
GGAGCTATCGGTGAAGACCTGCGTAAGGTCGCCGTAACAGCCATCGGTGTCGGTATTGTA
GGATTGGCTGTATCTGGGGACACTATAACCGTCAAAGAAGCCGGTTTGGTGTTGGTCATT
GGGGTTATCCTGTGGATCTATGGTATAATCTTAACCAAGGTCAGCAATTCCTAAGGGGGT
CAAATGGACGCTTTCACATTAGGCATGTTGGGGTTGCTCATTTTTTTCACTGTCGTCACT
GGCGGCAGTCTGTATCTCTACCATGAGAAACAGAAGGAAAAAAAGCATCACAACGCCTAA
AGAGTAACTGCGATAATGAACGTAAAGGCCAGCAATAGCTGGCCTTTTTTTATACTTGCA
GTTTGAAGTGTTACATGTCACGATGAAGTTGGTGTGACATGTAACGGTAAAGGTGTTTTT
TTGAAAGTATCCAATGAAGATGCTCAGGCTACGGCGATCTATCTCCTCAGAGCTGCTTCG
CGCCCAGCTTTCTGGCGTGACGTCCCATTCGATAAGAAACTTGAAGCCGTGGACAGCCTG
AACAGCATGGGGCGATCACCATCAGAACTCACTGAATGGATTAATAAATATCTGACAGCA
GAGCAAATCAATAAACTCGGGACATCAATTAGGCAACGTCGCAGAAGGGGATATGGTGTT
GGTAAAAGCATAACTATAAGCGATAAAGCCCACAGGATTTTGAAGCGGTTGGCAGAAGTC
GATGGTTGCAATTTGTCAGAAGTGATAGAGAAACGCCTAGCCCGAGCTTATAAAAAATACT
TGGGACCACAAATAGAGTGGCACTAGGGGTTGCATTTTTAAAACCCGTGGTAGCATCTAA
ATGAACCCTAACGAATAGGGGTGCTGGCGGCGATGCCGACCATTATCCCGATAATGGAGA
AATAGACCATGATGACACTGACTACTGTCTCGAAGAAAACTTCTAATAATTCAGCCCTTG
TATTCTGGCGCGTTGGTACAAAACGGAAAGGCATCCTTGATGTTCGTATTGATTTTGACA
ACGAGGAGGCAGATCTTTTGGCTGAGCTCGTAGCCATTCGCTATCTGGCGCTGGACAAAC
AGGTTTTTTGCAGAGAGCCAGGTGCTGGTGCTGGTTACAAGCTGGTGGTATCCAAAGGTG
CGATTAAAAAGCTGGCGCTGGGCAAATCCACCAAGGAGTTTGCATTCAAGTTTGCGGCTT
GCCTCACTGGACGTTTAAAGGGCGCTACTATTGAAGTTTCGCAGAGCATGGAGTTTATGG
ATGAGCCGGGTGAGGGCAACGTTGAGCTCCTCGATGTGGACAAGCAAGCCTATACTCAAA
CCCATGACGAAATCTCTACACCGGCCATTGGTCCCGTCCTTGTTACTCAACATGCCATCG
ATCAGTATCAGGCCCGGATAACCTCTGGAGACCCTAAAAAACCGTGGGCCTCACTCGTTG

GTCGCCTCCAGCATCCAGAGTTACAGGTTCAACCCTTTGACGAGAAAGTGGCTCGCCATA
AAGCCAGAAAGTATGGCCGCGTAGATAACGTGGAAGTCTGGGGCCATAGAGATTCCAAGT
TCAAGTACCTGATGGTGATCAACGACGACAACCAAAAACGTGTTCTTGTCACAGTGTTTG
AGCGAAATGAGTAACCTGCTTCTCATTTGTTCTGAATACACAACCTCTCCAAATCACGTA
TTCATACCCTAAAAGGGGTCGTCTCAGAAAACGGAAAATAAAGCACGCTAAGCCGGTTGC
AGAGGCCGTAGCGGCCTGAACTTCCCCGCGCCGATCTTGGCGCTGCTGCGCCATAGGTAA
TCACCGGTCAGGTTGATGTGCTCCCAGCCGAGTGGCGACAGGTACTGCAATAGCGAGTCA
TCGACGGCATGACCATTGCCGCGCAACGCATGCGCCGCACGCTCCAGGTAGACCGTGTTC
CACAGCACGATGGCCGCCGTCACCAGGTTGAGGCCGCTGGCCCGGTAGCGCTGCTGCTCG
AAACTGCGGTCACGGATTTCACCAAGGCGGTTGAAGAACACGGCACGGGCCAGCGCATTG
CGCGCCTCGCCCTTGTTCAGCCCGGCATGCACGCGGCGGCGTAGCTCGACGCTTTGCAGC
CAGTCGAGGATGAACAGCGTGCGCTCGATGCGGCCCAACTCGCGCAGCGCGACGGCCAAG
CCGTTCTGGCGCGGGTAGCTGCCGAGTTTCCTGAGCATCAGCGAGGCCGTCACCGTGCCC
TGCTTGATCGAGGTGGCCAGCCGCAGGATTTCGTCCCAATGGGCGCGGACGTGCTTGATG
TTGAGCGTGCCGCCGATCATCGGCTTGAGCGCGTCATAGGCGGCATCGCCCTTCGGGATG
TAGAGCTTGGTGTCGCCCAGGTCGCGGATGCGCGGCGCGAAGCGGAAGCCCAAGAGGTGC
ATCAGGGCGAAGACGTGATCGGTGAAGCCCGCCGTGTCGGTGTAGTGCTCCTCGATCCGC
AGGTCGGATTCGTGGTACAGCAGGCCGTCGAGCACGTAGGTTGAGTCGCGCAGGCCGACA
TTGACCACCTTGGTGTGGAATGGCGCGTATTGGTCGGAGATGTGGGTGTAGAAAGTCCGT
CCTGGGCTGCTGCCATATTTTGGGTTGATGTGCCCCGTGCTCTTTGCCTTGCTAGCGGTT
CGGAAATTCTGTCCGTCCGATGATGATGTGGTGCCATCGCCCCAGTGCCCGGCAAAGGGA
TGCCGAAACTGAGCGTTGACCAGTTCAGCCAACGCTGTCGAGTACGTTTCGTCGCGGGTA
TGCCAGGCTTGCAGCCAAGCGAGCTTCGCGTAGGTCGTGCCGGGGCAGGACTCGGCCATC
TTGGTCAGGCCCAGGTTGATCGCGTCGGCCAGGATCGTGGTCAACAACAGGTTCTTGTCC
TTGGCCAGATCGCCCGATTTCAAGTGCGTGAAGTGCCGGGTGAAGCCCGTCCACTCATCG
ACTTCGAGCAGCAGTTCGGTGATCTTGACGTGCGGCAGGACCATGGCTGTCTGGTCTATC
AGCGCCTGCGCGGTGTCGGGCACCGCCGCATCCAGCGGCGTGATCTTCAAGCCCGACTCG
GTGATGATGGCATCCGGCAGGTCGTTGGCTGCCGCCATGCGGTTGACGGTGGCAAGTTGT
GCTTCCAGCAGCGTCAGCCGCTCATGCAGATATTGTTCGCAGTCGGTGGCCACGGCCAGC
GGCAATTCGCTGGACTGCTTGAGGCTGGTGAACTTCTCGGGCGGTACCAGGTAGTCCTCG
AAGTCCTTGAACTGGCGTGAACCCTGCACCCAGATGTCGCCCGAGCGCAGGGAGTTCTTC
AACTCGGACAGCGCGCACAGTTCGTAGTAGCGCCGGTCGATGCCGGCGTCGGTCATCACC
AGTTTCTGCCAGCGCGGCTTGATGAAGCCGGTCGGTGCATCGGCTGGCAGCTTGCGGGCG
TTGTCGGTGTTCATGCCGCGCAGCACCTCAATGGCATCAAGCACGTTTTTGGCGGCGGGC
GCGGCCCGCAGCTTGAGCACGGCAAGGAATTCCGGTGCATAGCGGCGCAGGGTGGCGTAG
CTCTCGCCGATGCGATGCAGGAAATCGAAGTCATCGGGTTGCGCGAGCTTCTGCGCCTCG
GTGACGCTCTCGGCAAAGGAATCCCAGGACATGACGGCCTCGATGGCGGCAAACGCATCG
CGGCCTGATTGCTTGGCGTCGATCAGCGCCTGACCGATGCGCCCGTACAGACGTACCTTG
GCGTTGATGGCCTTGCCTGACGCCTGGAACTGCTGCTGATGCTTATTCTTGGCAGCGTTA
AACAGCTTACCCAGGATGCGGTCGTGCAGGTCGATGATTTCGTCGGTGACGGTGGCCATG
CCCTCGGTGGCCAGCGCCACGAGAGTGGCGTAGCGCCGTTGCGGCTCGAATTTGGCCAGG
TCGGCGGGTGTCATCTGGCCGCCCTCGCGGGCAATCTTGAGCAGGCGGTTCTGGTGAACC
AGCCGCTCGATGCCGGTAGGCAGATCGAGTGCCTGCCATGCCTTGAGGCGTTCGATGTGT
TCCAGCATATGCCGCGAATTTGGCTTGGCCGGAGACTGGCGCAACCAAGCCAACCAGGTC
GTCTTGCCGTTGTCCCGGCGCTTGAGCAGATCGTCGAGGCGGCGGCGATGCGCGTCCGCC
AGTGGTTCGGCCAAGGCGTCGTAGATGCGCCGGTTAGCACGGGTGATCGCCTCGGCACTC
GCCCGCTCGACGGCGTTGAGGGCGGGCAGAATGACCGACTGCCGCCGCAGGTGCCCGATC
AAGGCGCTGGCCAGCACGATGCCTTTGTCGGTTTGCATCGCCAGCTCGGTCAGCATCTGG
ACGGCCTGCCGGTAATGGCTCATGGTGAAGGGCCGGAAACCGAACACGGTTTGCAGCTCG
CTCAGGTGCTCGCGCCGGGTCTGCTCCCGCTGGCCGTACTCGTTCCAGCTTTCGACGCCG
ACCTTGAGCTGGTCGGCGACCAGCTTCAACAAGGGCGGGAACGGTAGTTCATCGACGCCC
AGGATGACGCCGGGAAAGCGCAGGTAACAGAGCTGCACCGCGAAGCCCAGCCGATTGGCT
GGCCCGCGCCGCTGTCGGATGATCGAGAGGTCGGTATCGTTGAATGTGTAATGTCGGATC
AGGTCGTCCTTGGAGTCCGGCAACGCCAGCAGGCTTTCCCGCTCGGCGGCGGACAGGATG

GAACGACGTGGCATATTTACTGATCCGTTCTCAAGTATTGATACAGGGTTTCGCGACTGA
TTCCGAATTCACGAGCAAGCTTGGTCTTTTGCTCGCCAGCCTCGACACGTTGGCGCAGTT
CGGCAATACGCTCAGACGACAGGGATTTCTTCCTGCCACGGTAAGCCCCGCGTTGCTTGG
CGAGCGCAATACCCTCGCGCTGACGCTCGCGGATCAGGGCGCGCTCGAACTCGGCGAACG
CGCCCATCACCGAGAGCATCAGGTTCGCCATCGGAGAGTCTTCGCCAGTAAAACTGAGGT
GTTCCTTGACGAATTCGATATGCACGCCGCGTTGTGTCAGCGTTTGCACGATCCGGCGCA
AATCATCGAGATTGCGCGCCAGGCGATCCATGCTATGCACCACCACGGTGTCGCCGGTGC
GGGCGAAGCTTATCAGCGCTTCCAGTTGCGGACGCTTGACATCCTTGCCGGATGCCTTGT
CGCTAAAAGCGCGATCAACCTTGACGCCTTCCAGTTGCCGTTCCGGGTTCTGGTCGAAGG
TGCTGACCCTGATATACCCAATGCGCTGTCCAGTCATGGAATTCCCTGCAAAATGTCAGG
GAAGACTCTATGACCTTCAACGAGATATGTCAATAAATTCAAAATTCAATCCTATCCTGA
CGCAATTTACACATGGCATCTGACATCAGGTTAGGGTATGCCTCAACCTGACGGCGGCGA
ATCACAAGCGTCCGGTTTGACGCTGGTTTCGGTCGCAGTGCTGGCCCGCGCCTGGAAGCG
CTGATAGCACTCCAGCCCGCAGAAATGCTCCACGTACTCGGCCCCTTCCGGCGTGAAGGC
GGCATCGAGCGGGATTTCCTTGCAGCACACGCAGCAACTGGTGCTCGGTTCATTGGCGTT
CATGGTGGTGTTCCTCCATTGGTTGACGAAGCCGACGAAGGCCGCCGCCGGCATCGGCCT
GGCGAACAGGAAACCCTGCACCGTGTCGCAACCCGCCTGCCGCAACCACGCAAGGCAGTC
GGGTGTTTCCACACCCTCGGCTACCACCTCCATTGTCGTTTTCAGAAGACGGCTGCACTG
AACGTCAGAAGCCGACTGCACTATAGCAGCGGAGGGGTTGGATCCATCAGGCAACGACGG
GCTGCTGCCGGCCATCAGCGGACGCAGGGAGGACTTTCCGCAACCGGCCGTTCGATGCGG
CACCGATGGCCTTCGCGCAGGGGTAGTGAATCCGCCAGGATTGACTTGCGCTGCCCTACC
TCTCACTAGTGAGGGGCGGCAGCGCATCAAGCGGTGAGCGCACTCCGGCACCGCCAACTT
TCAGCACATGCGTGTAAATCATCGTCGTAGAGACGTCGGAATGGCCGAGCAGATCCTGCA
CGGTTCGAATGTCGTAACCGCTGCGGAGCAAGGCCGTCGCGAACGAGTGGCGGAGGGTGT
GCGGTGTGGCGGGCTTCGTGATGCCTGCTTGTTCTACGGCACGTTTGAAGGCGCGCTGAA
AGGTCTGGTCATACATGTGATGGCGACGCACGACACCGCTCCGTGGATCGGTCGAATGCG
TGTGCTGCGCAAAAACCCAGAACCACGGCCAGGAATGCCCGGCGCGCGGATACTTCCGCT
CAAGGGCGTCGGGAAGCGCAACGCCGCTGCGGCCCTCGGCCTGGTCCTTCAGCCACCATG
CCCGTGCACGCGACAGCTGCTCGCGCAGGCTGGGTGCCAAGCTCTCGGGTAACATCAAGG
CCCGATCCTTGGAGCCCTTGCCCTCCCGCACGATGATCGTGCCGTGATCGAAATCCAGAT
CCTTGACCCGCAGTTGCAAACCCTCACTGATCCGCATGCCCGTTCCATACAGAAGCTGGG
CGAACAAACGATGCTCGCCTTCCAGAAAACCGAGGATGCGAACCACTTCATCCGGGGTCA
GCACCACCGGCAAGCGCCGCGACGGCCGAGGTCTTCCGATCTCCTGAAGCCAGGGCAGAT
CCGTGCACAGCACCTTGCCGTAGAAGAACAGCAAGGCCGCCAATGCCTGACGATGCGTGG
AGACCGAAACCTTGCGCTCGTTCGCCAGCCAGGACAGAAATGCCTCGACTTCGCTGCTGC
CCAAGGTTGCCGGGTGACGCACACCGTGGAAACGGATGAAGGCACGAACCCAGTTGACAT
AAGCCTGTTCGGTTCGTAAACTGTAATGCAAGTAGCGTATGCGCTCACGCAACTGGTCCA
GAACCTTGACCGAACGCAGCGGTGGTAACGGCGCAGTGGCGGTTTTCATGGCTTGTTATG
ACTGTTTTTTTGTACAGTCTATGCCTCGGGCATCCAAGCAGCAAGCGCGTTACGCCGTGG
GTCGATGTTTGATGTTATGGAGCAGCAACGATGTTACGCAGCAGGGCAGTCGCCCTAAAA
CAAAGTTATGCCGCACCCACCCCTATGGAGTCTTGATGTTAAAAGTTATTAGTAGTTTAT
TGGTCTACATGACCGCGTCTGTCATGGCTGTCGCAAGTCCGTTAGCCCATTCCGGGGAGC
CGAGTGGTGAGTATCCGACAGTCAACGAAATTCCGGTCGGAGAGGTCCGACTTTACCAGA
TTGCCGATGGTGTTTGGTCGCATATCGCAACGCAGTCGTTTGATGGCGCGGTCTACCCGT
CCAATGGTCTCATTGTCCGTGATGGTGATGAGTTGCTTTTGATTGATACAGCGTGGGGTG
CGAAAAACACAGCGGCACTTCTCGCGGAGATTGAAAAGCAAATTGGACTTCCCGTAACGC
GTGCAGTCTCCACGCACTTTCATGACGACCGCGTCGGCGGCGTTGATGTCCTTCGGGCGG
CTGGGGTGGCAACGTACGCATCACCGTCGACACGCCGGCTAGCCGAGGCAGAGGGGAACG
AGATTCCCACGCATTCTCTAGAAGGACTCTCATCGAGCGGGGACGCAGTGCGCTTCGGTC
CAGTAGAGCTCTTCTATCCTGGTGCTGCGCATTCGACCGACAATCTGGTTGTATACGTCC
CGTCAGCGAACGTGCTATACGGTGGTTGTGCCGTTCATGAGTTGTCAAGCACGTCTGCGG
GGAACGTGGCCGATGCCGATCTGGCTGAATGGCCCACCTCCGTTGAGCGGATTCAAAAAC
ACTACCCGGAAGCAGAGGTCGTCATTCCCGGGCACGGTCTACCGGGCGGTCTAGACTTGC
TCCAGCACACAGCGAACGTTGTCAAAGCACACAAAAATCGCTCAGTCGCCGAGTAGCAGA

TGCGGCATAACAAATCGTTGGAGCGGGACTTTTGCTACGCAGGCTGCGCCTACTCCGCAA
AAGCCCCTCAACTCAGGCGTTAGGCACCAATGGATAGTTCGCCGCTCGTCAGGCCTGTTG
AAACTACCGATTCGGCCAGTTGGCTAAGCATGCGCTGTGAGCTGTGGCCAGATGGCACAT
GTCAAGAGCACCAGTCAGAGATCGCAGAATTTCTGTCCGGAAAAGTCGCCCGGCCTGCTG
CTGTCCTCATTGCTGTAGCACCCGACGGAGAAGCACTAGGGTTTGCCGAGCTTTCGATCC
GCCCGTATGCGGAGGAGTGCTACTCCGGCAACGTTGCGTTCTTGGAGGGTTGGTACGTTG
TGCCAAGTGCGCGGCGTCAGGGCGTAGGTGTAGCTCTGGTAAAAGCCGCCGAGCATTGGG
CTCGTGGTCGCGGATGCACCGAATTCGCCTCCGACACTCAACTTACCAACAGCGCAAGCA
CCTCGGCGCACCTGGCGGCTGGATTCACGGAGGTTGCTCAAGTACGCTGCTTCCGGAAAC
CGTTGTGAGGGGCGCCGCGTTGGTGCCTAACAATTCGTTCAAGCCGAACTTGCTTCGTTA
CACCAAAGCCATGGCAGAAAGAGCTTGCCATGGCTTTGGCTCCACTACGCAAGTCGGCTT
AACTCAGGTGTTAACCTCTGAGGAAGAATTGTGAAACTATCACTAATGGTAGCTATATCG
AAGAATGGAGTTATCGGGAATGGCCCTGATATTCCATGGAGTGCCAAAGGTGAACAGCTC
CTGTTTAAAGCTATTACCTATAACCAATGGCTGTTGGTTGGACGCAAGACTTTTGAATCA
ATGGGAGCATTACCCAACCGAAAGTATGCGGTCGTAACACGTTCAAGTTTTACATCTGAC
AATGAGAACGTAGTGATCTTTCCATCAATTAAAGATGCTTTAACCAACCTAAAGAAAATA
ACGGATCATGTCATTGTTTCAGGTGGTGGGGAGATATACAAAAGCCTGATCGATCAAGTA
GATACACTACATATATCTACAATAGACATCGAGCCGGAAGGTGATGTTTACTTTCCTGAA
ATCCCCAGCAATTTTAGGCCAGTTTTTACCCAAGACTTCGCCTCTAACATAAATTATAGT
TACCAAATCTGGCAAAAGGGTTAACAAGTGGCAGCAACGGATTCGCAAACCTGTCACGCC
TTTTGTACCAAAAGCCGCGCCAGGTTTGCGATCCGCTGTGCCAGGCGTTACGACGTTGCT
GGCCGTACATTTGTACGGCTCCGCAGTGGATGGCGGCCTGAAGCCACACAGTGATATTGA
TTTGCTGGTTACGGTGACCGTAAGGCTTGATGAAACAACGCGGCGAGCTTTGATCAACGA
CCTTTTGGAAACTTCGGCTTCCCCTGGAGAGAGCGAGATTCTCCGCGCTGTAGAAGTCAC
CATTGTTGTGCACGACGACATCATTCCGTGGCGTTATCCAGCTAAGCGCGAACTGCAATT
TGGAGAATGGCAGCGCAATGACATTCTTGCAGGTATCTTCGAGCCAGCCACGATCGACAT
TGATCTGGCTATCTTGCTGACAAAAGCAAGAGAACATAGCGTTGCCTTGGTAGGTCCAGC
GGCGGAGGAACTCTTTGATCCGGTTCCTGAACAGGATCTATTTGAGGCGCTAAATGAAAC
CTTAACGCTATGGAACTCGCCGCCCGACTGGGCTGGCGATGAGCGAAATGTAGTGCTTAC
GTTGTCCCGCATTTGGTACAGCGCAGTAACCGGCAAAATCGCGCCGAAGGATGTCGCTGC
CGACTGGGCAATGGAGCGCCTGCCGGCCCAGTATCAGCCCGTCATACTTGAAGCTAGACA
GGCTTATCTTGGACAAGAAGAAGATCGCTTGGCCTCGCGCGCAGATCAGTTGGAAGAATT
TGTCCACTACGTGAAAGGCGAGATCACCAAGGTAGTCGGCAAATAATGTCTAACAATTCG
TTCAAGCCGAAGCCGCTTCGCGGCGCGGCTTAATTCGGGCGTTAGATGCACTAAGCACAT
AATTGCTCACAGCCAAACTATCAGGTCAAGTCTGCTTTTATTATTTTTAAGCGTGCATAA
TAAGCCCTACACAAATTGGGAGATATATCATGAAAGGCTGGCTTTTTCTTGTTATCGCAA
TAGTTGGCGAAGTAATCGCAACATCCGCATTAAAATCTAGCGAGGGCTTTACTAAGCTTG
CCCCTTCCGCCGTTGTCATAATCGGTTATGGCATCGCATTTTATTTTCTTTCTCTGGTTC
TGAAATCCATCCCTGTCGGTGTTGCTTATGCAGTCTGGTCGGGACTCGGCGTCGTCATAA
TTACAGCCATTGCCTGGTTGCTTCATGGGCAAAAGCTTGATGCGTGGGGCTTTGTAGGTA
TGGGGCTCATAATTGCTGCCTTTTTGCTCGCCCGATCCCCATCGTGGAAGTCGCTGCGGA
GGCCGACGCCATGGTGACGGTGTTCGGCATTCTGAATCTCACCGAGGACTCCTTCTTCGA
TGAGAGCCGGCGGCTAGACCCCGCCGGCGCTGTCACCGCGGCGATCGAAATGCTGCGAGT
CGGATCAGACGTCGTGGATGTCGGACCGGCCGCCAGCCATCCGGACGCGAGGCCTGTATC
GCCGGCCGATGAGATCAGACGTATTGCGCCGCTCTTAGACGCCCTGTCCGATCAGATGCA
CCGTGTTTCAATCGACAGCTTCCAACCGGAAACCCAGCGCTATGCGCTCAAGCGCGGCGT
GGGCTACCTGAACGATATCCAAGGATTTCCTGACCCTGCGCTCTATCCCGATATTGCTGA
GGCGGACTGCAGGCTGGTGGTTATGCACTCAGCGCAGCGGGATGGCATCGCCACCCGCAC
CGGTCACCTTCGACCCGAAGACGCGCTCGACGAGATTGTGCGGTTCTTCGAGGCGCGGGT
TTCCGCCTTGCGACGGAGCGGGGTCGCTGCCGACCGGCTCATCCTCGATCCGGGGATGGG
ATTTTTCTTGAGCCCCGCACCGGAAACATCGCTGCACGTGCTGTCGAACCTTCAAAAGCT
GAAGTCGGCGTTGGGGCTTCCGCTATTGGTCTCGGTGTCGCGGAAATCCTTCTTGGGCGC
CACCGTTGGCCTTCCTGTAAAGGATCTGGGTCCAGCGAGCCTTGCGGCGGAACTTCACGC
GATCGGCAATGGCGCTGACTACGTCCGCACCCACGCGCCTGGAGATCTGCGAAGCGCAAT

CACCTTCTCGGAAACCCTCGCGAAATTTCGCAGTCGCGACGCCAGAGACCGAGGGTTAGA
TCATGCCTAGCATTCACCTTCCGGCCGCCCGCTAGCGGACCCTGGTCAGGTTCCGCGAAG
GTGGGCGCAGACATGCTGGGCTCGTCAGGATCAAACTGCACTATGAGGCGGCGGTTCATA
CCGCGCCAGGGGAGCGAATGGACAGCGAGGAGCCTCCGAACGTTCGGGTCGCCTGCTCGG
GTGATATCGACGAGGTTGTGCGGCTGATGCACGACGCTGCGGCGTGGATGTCCGCCAAGG
GAACGCCCGCCTGGGACGTCGCGCGGATCGACCGGACATTCGCGGAGACCTTCGTCCTGA
GATCCGAGCTCCTAGTCGCGAGTTGCAGCGACGGCATCGTCGGCTGTTGCACCTTGTCGG
CCGAGGATCCCGAGTTCTGGCCCGACGCCCTCAAGGGGGAGGCCGCATATCTGCACAAGC
TCGCGGTGCGACGGACACATGCGGGCCGGGGTGTCAGCTCCGCGCTGATCGAGGCTTGCC
GCCATGCCGCGCGAACGCAGGGGTGCGCCAAGCTGCGGCTCGACTGCCACCCGAACCTGC
GTGGCCTATACGAGCGGCTCGGATTCACCCACGTCGACACTTTCAATCCCGGCTGGGATC
CAACCTTCATCGCAGAACGCCTAGAACTCGAAATCTAACGTCCGTTCGGGCATCGAGGTC
CATGTCGGGGTGGGACGGGCCCGTGGCTTCAAGATCACTTGCAGTCCGACCGCGATGTCT
TGGTTGCGCGAGAGGTTGTCGATATCTGTTGATTTGCACCCAAATTTGACCCGGGATTTG
CATTGAATTTTGACCCACCCCTTGTTGTCAGAATTATGTCTCGATTTTCAGTTTGCGGGT
CTGTTTTTCCTCCTGCTTATTCTGAGTTGAACTGTGTTTGAAGCGGTAACTTTCATTGCC
GGTTTCCAGGATGTGGCAGTGGTGGGTTAGTCGGTCCAACAACGCTGTTGTCATCTTTTC
ATCGCCAAACACTCGGCTCCATTCCGAGAAGCTCAAGTTGGTGGTCAGTATCACGCTGGT
TTTTTCGTACAGCTTTGAGAGCAGGTGAAACAGCAGTGCCCCACCGGTTTGGCTAAAAGG
CAAATATCCCAGCTCATCCAGAATCACCAAATCGGCATACAACAGACGGTTTGCGATTTG
TCCCTGACGCCCAGATGATTTCTCTTGCTCCAGTGCATTGACCAAATCCACGGTGGAGAA
GAAACGCACCCGTCGGTTCAAGTGCATCACTGCTTGTGTACCAATGGCTGTGGCCAGGTG
AGTCTTGCCTGTGCCTGGCCCACCAATCAGCACCACGTTCTGGGCTTGTTCCATGAAGTC
GCACCGGTGCAATTGTTTGACCGTGGCCTCATTAACCAGGCTTTGACTGAAGTCAAAGCC
CACCAAGTCCCGATACACGGGGAACTTGGCCACCCGCAATTGATAGTTCACCGAACGTAC
TTCACGCTCTGCCACTTCAGCTTTAATCAAGCTGTCCAGCATGGGCAAGGCTTGATTAAA
TGCTGGTGAATTCTGATTGCCCAACTCCTCAATGGCGTGTGCCATGCCAAAGAGTTTCAA
GGATTTGAGGATTCTCACATGGCCTTCATGCTGCATCATGGGCTCTCCTTAAACTGTCAT
AGCGGTTCACGTTGGCCTGTGGTTCCAATGTCAGCCTTAACCCCTTGGGAATTGGAATCG
GTTTGGGTGGAGGTTCTTCGGTCAAACGTCCCAACAGATTAAGCACATGCTCCTTCGATG
GCTTGCCACACTCCAATGCCAATTCCACAGCACTGAGTACCGCACCTTCATCGTGGTGCA
ATACAAGGGCCAGAATTTCCACCATGTCACGGTCACCGCCGGGGCGTTGCAGCAAGATGG
ATTGAAGCTTCTTGAACGCGGGTGGCAATTCAGCAAATGGCGCACCATTGCGCAACGCCC
CAGGTTTCTTCTGAAGCACAGACAAGTAATGGTGCCAGTCGTATTGTGTGTGGCCACGCC
GAGCGTGGCCACTGCCAAACAATCTTGGATGCTCGGCAATGTGTTGGCCTTCGGCAGCCA
TCACCAGCTTGTCTGCATAAATCCGAAGGCTGATGGCCCTGTTGGCGTAACTGGCAGGAA
CGCTGTAGCGATTGCCCTCGTGGTGAACAAGGCAGGTTGAAGTGACTCGCTTGGTTTGCT
CCACGAATGCATCAAAGGCATTGGGTAGCGCCATCAACTCGCCTTGTTCATCGGCAAAGG
CCTCTTGCACGGTTTGGTCCAATTCGGGGTGGCGCAGCTCAGACCACAGCGCTTTGCAGC
GATGCTCAAGCCACACATTCAAATCAGCAAGGCTTTGAAAGTCTGGTGCCCCTTGCCACA
GGCGTTGGCGGGAATCCTGCACGTTCTTCTCAATCTGGCCTTTCTCCCAACCCGATGCTG
GATTACAGAACTGCGCATCAAACAGGTAGTGGCTGACCATGGCAGTGAACCGCTGATTGA
CCCTGCGCTCTTTGCCACGCCCCACCGAATCCACAGCGGTCTTCATGTTGTCGTAGATGC
CGCGCTTGGGAATGCCACCGAAGATTTGAAAGGCATGCCAGTGGGCATCAAACAGCATTT
CATGTTTTTGCTGGTAGTAAGCCCGAAGCACAAAGGCCCGGCTGTGGGCCAACTTAAACT
GGGCAATCTGAAGTTTGACCTGTTTGCCCGCTATGCGGGCAAAGTCCTCACTCCAATCGA
ATTGGAAGGCTTCGCCACAAGCAAAGCGCAAGGGGATGAAACAACCCTTGCCCGAGGTTT
GCGCCTTGAACTGTTCGGAATCTTTCCACTGTCGGGCAAAGGCACACACTCGGTCATAAG
ACCCGGTAAAGCCCAAAGCGACCAAATCCCGGTACATGCTGCGCAGGTTTCTGCGCAGCT
TCTTTGTCTTTTTGTGCTCGGTGGAGAGCCACTGCCTTAACTTGGGCTCAAAAGGACTTA
ACTTGCCAACGCTGTCTCGCGCTGGGTACTGCGGTTCAACCACCTTGCTTTGCAAATACT
TGCGAACGGTGTTCCTGGACAGGCCGCTTCGTCGGGCTATTTCCCGAATCGACGCACCAT
CGCGAAAATGCCAGCGTCGAATTGCGCTCAATATCGCCACGTTTATCACTCCTTGATTTC
TCCCGCCATATCCAGACGGGAAACAGTGTCATACGTGGGTCAAATTTCGACGCAAATCTT

TACCCTAAGTGGGTCAATTTTAGATGCAACTCAACAGGCCATGCTGAGTGTGCGATGGTT
GATCGCTTCCTCGCCGCTCTCCACGGCGACGATGGCCGCCGCCATCAGCAAGTGCGCCAG
TTCCCCTATGGTGCCCTCGCTGCGTGTGAGCAGGTAGCGAGCCATGTCCAGCGTGGCAAT
TGGGGAAGGCCGGCGCAGCGGGAGCGAAGCGGCGAAGCTGGCCAGCAGTGAGCAGCAATC
GTCGTTGGCCTCCCATACCGGCAGCATCATCGGCTCGAAGCGATTTTCCAACTGGTCATC
GGAGCGGATGGCTAGGTAGGCGTCGCGCGTGCCTACCCCAACCAACGGGATGCGCAGTTC
GTTGCCGAGGAAGCGCAGCAGGTTGAGGAATTCCCGGCGGTTGACGCTGTTGCCGGCCAG
CACGTTGTGCAGCTCGTCGATCACCAGCAGCGCGGCGCTGTCGACCATGCGCCGGACGCC
ACAGCTGGGACAGAACCCTCGGCGTTTACAGGAGAAGGCCACCAGCTTCTCGTGGTGGCA
GGATTGGCAGCGTACGCGCAGAAAGCCGTGTTCCAGGCGGCCGCACTTGAGGTAGTCCTC
GAACTCGCGGGCGACATAGCCTGGCAGGGGTGAGCCTTGGGCGGCCATGAGCCGGGCAAA
CTCGGGGTAATGACGCTCAACCAGTTGGTACAGCAGGGTCTGCTCGGGCCGGTGGCGGAC
ATGGGTGGCCGGCCGAGGCGCTTGGGCGGTAAGAGACATGACGGCATCCGTTCCATCGGT
ACTGGTGTGTGGATGCCTTCAGGGTAGTGCAGCTTGGGGAAATGTGCGGAGATTGATGCT
GTGGGTGACAGCAAAAAGGCACTGGCAGAGCTGGAACAGAAGGAGTTTCCCCAGGTGGGT
CAGGTCACCTGTAGTATCGGTTTTGCGGCTGTGGACCCTGCCCAGCCACCGGCCAATATC
CTCGACAACGCCGACCAGGCCCTGTATTACGCCAAGGGCAATGGTCGCAACCGGACCTGC
CACTACGAAAGTCTGGTGGAAACCGGGCAGATAGCTAGGCGAGACAATATGGAAGGCAAC
GCCAGCGTCGATTTTGATATTGATGCACTGTTTGGGTAATGCCGTTTTATTGCGAGCGCA
AAGTGTCCAGCAGCTCCCTGTTCATCAATATCCATATGCCGGCAGCGGCAATAAACGGCA
ACATCAGATACCCGAACTGATAGCCCAGGGCAATGGCATTCATTTGCCAGGGGGCAAAAT
CGAGCTGAGTGGTGACATCGCTGCCGGTTTCAAAGGCCAGGGTTTTGACTGTGTCGAGCG
CAATACCAAACGCCTGAAAAGGCAGCAGCAAGGTTATCTCAAGGATATATAACAGCATAC
GGTAGTGCACTTCCTTGGTCAAATCTTGAACAAATGTTTGCTGGTGCTGGAGGAGTAATT
TCAACAGCCGCGGATATGGGAAAATGGCTTTCTATGCACACTAACGAGGGCAAAAATATA
AATGGAGAAAGATTACTATCAAAATCATTGCTAGAAGAATCCTATTCACCTCTACCTGGA
AGTCCTAAATATGGTCTTGGATGGTCTTTAAGTTCTGCAAATGTTAAACCAGCACGTATT
TCTCATAGTGGGGCGTTATCTACAATTCAAGCGCAACAAGATATTGTACCTTCAAGTGGA
TATGCTGTAGCCGTAATGCTTAATAGCTTTACGACAACTTTTGAACATGCTTATGAGATA
AGTTCGGGTATTATAAAATTGACCGAAGGACAAAAACCTAATATTAAAGTTCCTATGCCT
AAAATAATTGATTTATTTCTTGGTCTAATGACATTAATATACTTATTCTTAGGTATAAAA
GGTATTTTGCGAAGTAAGGAGTGGTCGAATAGAAGAAAACTTCATCCTACTTTGAGATAT
TATCTTCGTCTAATGCCTCAAATAATTCCAGTTTTGTTTATAGGGTGGTTGTTTTTTATT
GTTCCAAACCTACAAAATAATAGTTCTACAATTAAGGATGCTATTGGTATTTGGCCAGCT
GCAATGTTATTTTTGGCAGTAGTTTTCTTAATTGGAACTATAGTTACGGTTAGGAGAGTA
TATTATAGAGTAAGATTAAATAGAAATTGATAGATTTAGATATTACCTACTTGTGCTAAA
CACTTTGTTTATATTCCGCCGTCATGCAGCATAGTTTACGGAACGTGGGCTGATAATTTG
AAACAATGGGAGTATACTAATTACGAGGGTGTAGGGAGTCGTTATGGGTTACGTTGCAGA
GGAATCAAAGCCGGTGACAATGTACGTAATCTCTTGAATTTTCAGTTTAATATTTCATGA
CAACTTTTTGGGTACAATATCAATGGAATTGATAATGACGAGTACCTGTGGAAACCCTGA
GAAAAAGGATTGCATATCCATAACGAAGATGGCACATGGCACGCCGACTGGCCAGATTCA
GAATCATACGATATTGGTCCAGCTAGTATTGCCTGGCTGACATGGCATATCATATTTTGG
AGGTCAATGGTATTCGATTACTTTTTTGGAAATGGTACGCTGACTCACGAAGAGGCTCAA
TGGCCCGGAAATATAACAGCTGTAAGAGAAATGAACAATTGAACAATAATCTTCGAGACA
AATGGAGAACCTTACTGGTACGGCATATTTTTTATCTCCTCAAGGCACGTTGAGAGTATT
GTGTTGATAACAAGAAACTGAAGTCTGGGAAAGCCTTGAAAATACTAAGCTTAATAAAAT
GAAAGTAAGTCTGTCCACTCGACCTAAACCCTTGATGTGTGTTTTAGAGAACATGTCGAG
GGTGTTTTTTTTGACAAGAATGGGGTCAGGTTGAGGAGATAAATTGGATATCCACAACGG
TTTTTTTGATAAACTAACGTTGAATTAAATTCAGGCCTTCAAATAGACATAAAATATAAC
TTTTGTATCAATCAAAATTTAATTTTCTTGATTTTTACATATATTCATGTTAGAATGAAA
ACAAATAAACAATTTGTTTTTAAGTTCTCGTATGGAGGTGTTTTATGTTGTGGCTAAATT
TACAGAGTTAGAAAAAGAAAAAAATTCGTGAAGAATTGCTTAAAGTTGCATATCGATTTTT
TGTAGATAAAGGCTTTAAGAATACTTCTCTCGAGGATATTACTTCATCAGTTGGCATTGC
AAAGAGTTCTTTTTATATATTTTTTGAATCAAAAGAAATACTATATATGGAATTGTTAGC

ACATGAGGGAGAGCAAATTGAAAAGCAGGTTTGGCCAAAGGTTATAGCTGCAAAGGATAT
ACGTTCAGCTATAAAGATATACTTAAATGAGATGGCTTTGGAACTAGAGGATAAGATTTT
AACACAAAGGCTGGTTTACGACCTTGAGGAATACAAGATTGTATCTAGAAAGCTAAATCC
AGATTATGTTGGCTCAGAGCATCTAAGAAGCATCGTTCCTCTTGTGGAGTTTATAAAGTT
ACGTCAAGACTCTAATGAAATTATCGATGAAGAGCCAGGCATTATAGCTGGAGTTTTAAG
AGCAGCTTGGTTAATAGGTTCTCAAAAGGGAGATTTGCAGCAATACAATTATGAAAGAAT
TAAAGAGTTATTATTCGAAGCTGTTGCTGATCGAGTCACTCGATTTTAATTTAGACTAGA
TAAAGATATATGAAGGTGGTTATCTATGACAGAAAGAATAATTCGTTTTAATGATGTTTA
TATTTGCACTGAGAGTTTTGGAGATACTAATGATCCAACTATCTTATTAATAATGGGAGC
TACTTCATCAATGATTTGGTGGGAAGAAGACTTTTGTAGGAAGCTAAGCAATCAAGGGTT
TCATGTTATACGTTATGATAATCGGGATGTAGGCAGATCAATTACTTACGAGTATGGTCA
TCCAGAATATACCTTTGAGGATTTGGCTGATGATGCAATTCAGGTACTAGATGCGTATAG
GGTTGATAAGGCTCACATAGTCGGTATGTCGATGGGTGGAATTATTACGCAGATAGTAGC
TCTTAAACATCCCGATAGAGTTCTTACAATTTCACTAGTTATGACATCGAATTATGATTC
TAGTCTTCCTAAGAAGGATAGCAAAGTAACAGAAGTTTTTGCCAAAATTAACATCAATAA
CTGGGAAGATAAAGAGGAAGTGATCAAGTATGTTATGGATAGGAGTAAAGTCCTCGTAGG
AACTAAGCATCCATTTAATGAAGAGAAAATAAGGAAATTAGCTGGGAAGGATTTTGATCG
TGCTAGTAATATGGAGAGCATGGAAAACCATGAATATGTAAAAGGATGGGGATTATATTT
ATCTAGAACCCATGAAATTAATGTTCCTACACTAGTTATTCATGGGACTGATGATCCTAT
TATACCTCATGAGCATGGAGCGCATCTTTCTAAAATTATATCTAATGCTGTGCTAGTTAC
CCTGGATGGTGCTGGGCATGAGCTACACTATGATGATTGGGATGAAATTATTAATGCTTT
ATCCAAGCATGCTGCAAGTTTATAATTGCTATATAAAAGCTAAAGTCGGGAGTGATTTTT
AATGAGTAAAGATATTAAACAAGTAATCGAGATAGCAAAAAAACACAATCTTTTTCTAAA
AGAAGAAACGATACAGTTTAATGAATCAGGGCTTGATTTTCAAGCTGTTTTTGCACAAGA
TAATAATGGAATTGATTGGGTTCTAAGATTGCCTAGACGGGAAGATGTGATGCCTAGAAC
AAAGGTAGAAAAACAAGCTTTGGATTTGGTAAATAAGTACGCTATATCCTTTCAGGCACC
AAACTGGATCATTTACACAGAGGAACTAATAGCTTATAAAAAGTTAGATGGTGTGCCAGC
AGGTACGATAGATCATAACATAGGTAACTATATTTGGGAGATAGACATAAATAATGTTCC
AGAATTATTTCACAAGTCGCTAGGCAGGGTGTTAGCAGAGCTTCATAGCATACCTAGTAA
TAAAGCCGCAGCGCTTGATCTTGTAGTACACACACCAGAAGAAGCAAGAATGTCAATGAA
GCAGCGTATGGATGCAGTAAGAGCAAAGTTCGGAGTAGGTGAGAATCTATGGAACAGATG
GCAAGCGTGGTTGAATGATGATGATATGTGGCCTAAGAAAACTGGACTGATTCATGGAGA
TGTACATGCCGGACATACTATGATTGATAAGGATGCCAATGTGACTGGATTAATCGATTG
GACTGAAGCGAAGGTTACAGATGTTTCGCATGACTTTATTTTCAACTATAGAGCTTTTGG
GGAAGAAGGGTTAGAAGCTTTAATTCTCGCTTATAAGGAAATTGGTGGATATTACTGGCC
TAAAATGAAAGAGCATATTATCGAACTTAATGCAGCATACCCAGTTTCAATCGCTGAGTT
TGCATTAGTGTCTGGAATTGAGGAATATGAGCAGATGGCAAAGGAAGCATTGGAAGTACA
AGGTTCGTAATCGGTATAAAGTAGTTATCGATATGTTCCCGCCTACCACTATGGTAAATA
TTGCAACGTAATAAGTAAATAATTAGGTCTTAAGTTTCCAAAAATTATATTTGCGATATG
AATGATGAATAGGTTTTTGACTTTATTGGCTATTGAAGGGTATACGCAGCGTGAAATTGC
AAGAAAGATGCACTGTTCACAAAATACCATATTTAAAAGACTAATAAAAATAAAATATTT
TTCAAAAATGGTTGTCAAAAAGATCTTCTCCTTGCCTACGCATTGTTTAAGGATAGGGAA
TATAAACAGGTATTCCATTGTCGTCGAGCCTGAAATGCTTGCTCGTTACGGAGCCTATTT
CTCCGCCAAAGACCCCTCGAAACCCTGAAATGTCCATGAATTGCGCCAAATGCACCCACC
TTGCTGGCTGCTCTCCCATCAAGCCTCTCCTGCATCCGCACACTCCCCTGCAATCCGTGT
AGGTCGCATCGTCCGTGATGTCTGTTGGCGTGCTTGCTGTATGTTGGTTCAATCCGCCAG
TCCCTGCTCCCACCCAGGCAGCAAGGGTGGAGCTCGTGGTGGCGGCAATTGGGTGGATGG
AGAAACTTGTTTGGCTTCCTTGGCCTTGAGGTGGTTGAGGATCTTGTCGATGACCACAGG
GTCCTCGATGCAGGCAATCACCTTCACCGGCCCCTGGCACTTTTCGCATTCGGTAATGTC
GATGTTGAAGACGCGCTTCAGGCGTCTGGCCCAGGTCATGGCGGCCCGCTGCTCCAGCGG
TGTGCGTTCCTCGGGCGCAAGGTGCACGGTACCGACCTTGCCTGCCCCCTTGCCACGTTT
GGCCGGGGTGATCGTTACACGGTAGTGGCTGTTGGGGGCGAACACGCCGTGGAAGCGGGT
CAGGTTCACTCTAGGTTTGGGCACCAGCGCCGCCAGCTTCGCCATGAAGTCCAAGGGCTC
GAAGATCACATGGGTGGTGCCGTCCCGGTACGGTGTCTTGAGTTCGTAACGTACCCGGCC

GTCTCTTGTCAGCGCCAGACGCTGCTCCGAGATCGCCGGGCGCGCCACATAGCGGCACAG
GTGCTCCAGTTTCTGTCTTTCATGCGCCTGCACTGCCACGCCGGCGTGCAGGGAGAAGCC
GCCGGCCTTGGCCAACTGAGTCAATCTGTCGTCTTCCGCTGCTTTGGGGAGCAGGGTTTG
CAGGGTGAAGACCTTCTTGCCCTGCTGCGGGCCGAGGGCGATGCGGTAGGTAATGCTGTG
CCCGTGCACCTGCTGCATGGGCTCGTCTGCCAGGTGATCCAGAGTCAGGTAACTGTTCTC
GCTGTCCTGGGTCAGCACGCCCTCCTTCACGAGGAAACGGGCCAACCGGGTGCTGAGCTG
CTGCAGTACCGCCTCCAGATCGCCCTTGGTAGGCGCATTGACCCGGCGGAACACCTGGCT
GGTGTTGTTGTCGTCGCTGTTGCCGTCGTGGCACCGCGTCACATACACCCCGTCCAGGAA
CAGCATATGAAAGTGGACATTCAGGTTCAGGGCCGAGCCGAAGCGCTGGATGAAGGTCAC
CGCCCCGGTGCGGGCGGTGTCGTGAGTGAAGCCAGCCTGCCGGAGCAGCCAGGTGGCAAT
CGAGCGATAGACGATGCCCAAGGCCTTGCCCATCAGGTCCGGGTAGCTGGCAAACAGGAA
GCGTAGCTGGAAGGGGACACTGAGCACCCACTGTCTGAGTGGTACCTTGGGCAAGACCTC
ATCGACCAGCAGCGCGGCGCTGTCGACCTTGCGCAGCAGTGCCAGAGCCAGTTGCTCCAT
TTCCGGCAACCGTGGGCGTGGGCGCAGCGGCGCGCCCATCGCGGCGAGCAGCGCGACGTA
GAAGCGGATCACGGACGGCTCGGACGGCATCTGCACGACCAACACCGGGATGTGCTCCTG
GTCGGCGTCGGAGCTGGCCGGGTGGGTGCGGCGGAACTTCTCGACGATCATCGACTTGCC
ATTGTTGGTCGGGCCAACCAGCAGCAGGTTGGGCATGCGTTGCTTGTTTGGCCACGCATA
AAGGGCTTCCAGCCGGTTCAGCGCCTCGACTGCGCGCGGATAGCCGATCCAGCGGTCGGC
GCGAAGGCGCTGGATGCGCTCGTCCGCCGGAAGACGGGCCAAGCCCTGGGCCGCCGGCAG
CAGGTGGGACAGGTCGATGATGGGATATTCGTCCACGGCTACCACTCCTCAATCTGGTCG
AACGGTTTGGCGGGTGGCAAGTTGTCTGCCTGCGGGTCGGCAATATCCGTATCCGGCGGA
ACGGGCTTGTCCGGCCGAGCTGATGTCTTGAGGTGCTGGCGGCGATCCGCGTCACGCCGC
GCCTTGCGTGTGGCCTTCTGCGCGCTGGTCACAATCTCACGCATCTGGCCGATCATGCGG
AACAGCGCCGACTCATCCACCTGTTCGCGCCCTTGCTGCCGCAGTTTCGCCAGCGCCTGC
CGTTGTTCCCAGAGGGTGACAGCCGGATGCGACAAGGTACGGTAGGGAATTTCCAGGTAA
TGCTGTCCCTCCGGTTCCAGGACCCAGATACGGCTGATGTCGCGCGGATCGCGCCGGATC
AGAAAGGACGGCCAGCGTTCACGCCGCGCAATCCACGGCTTGAGCGCATCGGCGTAGTAG
TGGATGTGGTCGATGACAAAGCCGGTGCGGGTCAGCGTGCGCCGGAGGATCGGCAGAAAA
TCGACCAGGAACGAAGTAGCGCGTGTGACGACGGCCGGTACGCCGACACGCGCCACGGCC
TCGGCCCAGCGCGCGGCCGGCGGTTGGAGCAGGCCGTTGTGCACCGAACCGTGGTAGGTG
CCGACCGCCAATGTGAGCCAGCGCTCTAGCTCGCGCAGCGTCAGGGCGGCCTTGTTTTCG
GAATCGTAGTCGCCGCGCTGGTCAGGGTTGGAGAAGGTCGTTCCCGGCAGTTCGTCGTGA
ATCATCTGCATCGCCGTGCCGATGATCCGTTCCACGATGCCGCCATAGTGCGGCTGTCCC
AGCGGGCGATAGTCCAGCCGGATGCCATGCTGCTCGCAACCCCGGCGCAGGGCCTCGCTC
TTGAACTCGGCCGCGTTGTCTAGGTAGAGCAGCAAGGGCTTGCCGCTCATCTGCCAATCC
ATTTCCACGTTCAGTCCTTCCAGCCAAGGGCGCTTGTCGCAGGCGACATGCACGAGGCAC
AGGCCAACCGAAACGGCAGACGGCGCTTCCAGCGTGACGACCATGCCGAGCACGCAGCGG
GTGAACACGTCGATGGCGAGGGTCAGGTACGGGCGGCCAATAGGTTGCCGGTCGCGGTCA
TCGACCACGATCAGGTCGATGACCGTATGGTCTATCTGCACCTGCTCCAGCGGCGCGGTC
ACGGCAGGAGGCTCGCCGCCCACACCTTGTAGGTCACGAGCGGCATCCTGGCCTTCCCGC
CGGCGGATGACCTTGCGCGGGTCAAGGCTAGCGATCCGTAAGGCCACGGTATTGCGCGCC
GGCACTCGCAGTTTTTGAGCCTTGCACACCTGAGTGACTTCGCGGTGAAAGGCCGCTAGG
CTGCGCTTCTGCTTGGTCAGGAACCGCTTTTGCAGTAGCTCGTGGATGACGCGCTCGACC
GGTTCCGGCAAGCGCCCCTTACCTTTACCTCCACCGGACTGGCCGGGCACCAGATCCGTC
ACGAGGCCGCTGCCTTGCCGGGCACGCCGGATCAGAACGTATACCTGGCGCCGAGACAAG
CCCAGCGCCTGAGCCGCCATATCGGCCGCTTCGTGCCCGACCGTCTCCGACTGCGCCAAC
GGACTGATGATCTCCGCACGACGGCGCGCACGCTCCCAAGCCTCATCAGGCAGAGTGGCC
ACGCCTTGTTCTGGAATCCGTGGGGTGTCCGTCGCCATGCTCACCTCGCTTTGGTGCACA
CGAGTATTGAGCATAGTCGAGATTGGTGCAGATCACTTCTGATATTGAACTGTCAGGAGC
TGGCTGCACAACAGCCATTACGCCCAATCAACTGGTGCAGTCGTCTTCTGAAAATGACAT
CCATGCCCAGCCCGTGCGCGAGCTGGATCACCGCCCGCACGATAGTTTGGTCACGGGCAT
CATCCGGGAGCCTGGCGACAAAGGATTGGTCGATTTTCAATGTGGTGATGGGGCAGCATT
TCAGATGTTGCAGGCAGGAATAGCCGGTGCCGAAGTCGTCGGCGGCGAAGCGCACGCCGA
TGGCGCGCAAGGCGTCGAAACTGGCGAACAGGGCTGGATTGCCGAATGCGACCGATTCGG

TCAGTTCGATCTCCAGAAGCTCGGCGGGCAGGGCCATATCGGCCAGCACCCGCTTTACCT
CGTCGTCGAACGTTGGCCCAACCTGGCTGGCGGACACATTGATGGCAAGACGGAACGGTT
GCCATGCCGGTCCTTGCCACTTGTGCATCTGGCGACAGGCCTCGCCCAGCACCCACGCGC
CTATTTCCGGCATCAGGCCGAACGACTCGGCCAGCGGCAGGAACTGGCCGGGCGGCAACA
GGCCAAGCCTCGGATGCCGCCAGCGCATCAACGCTTCCGCGCCAGCGATCCGGTGATCGC
GCAGATCGACCAGCGGCTGGTAATGCAGGTCAAGCTGTCCGCGCGCCGCCGCCTGCGCCA
ACTCGGCCGCCGTCCATCCGGCGGGCTGCGAACTCGTCATGATCCGCCCCGGAAGGCGCG
CAGCAGCCGCGTTACGGCCAGAACGAACAAGCCGGTCAGCGCGAGCGCGGCAACACCCCA
ATGCTCGCCAAGGAAGGCACCGGCGGTCGTCCCGGCCAGCACGGCGGCGAGAATCGGCAG
ATGGCAGGGGCAGGTCAACACGGCCAGCGCACCCCACAGGTAGCCGGAAACGGGTTGGCG
CGTCTCGGGCGGCAGTTTGTCAGGGGCGTTCACGGCAATGCCTCCTCGTGCGCCCGCTCG
GCTGGCATGGAGGCCAGTTGCGCGTCCAGATGGGCCAACGCCGCGCGCCGCCGCTCGACC
AACTGGCGCAGCACGGCAAGCTGCGCTGCGGCTTGTGCGCCGTCCGCTGCGTCGAGCGCA
CGGCACAGCCGCGCCAGGGCATCCAGGCCGATACCCGCCTCGAAGGCCGCGCGCACGAAG
CACAGCCGTTGCAAGGCCGCATCGTCGAACACGCCGTAGCCGCCCGTGGTGCAGGCCACC
GGCCGTAACAAGCCGCGCACCAGGTAGTCGCGCACGATATGTACGCTCACCCCAGCGTTA
TGGGCCAGTTGCGATACCGTGTAGGCGCTCATCGCACACCTCCTTGTCCTCACCCGGCGC
AGCAGGAAAGCTGCTTCACATCCTTGTTGAAGGTCTGCGCCGCGAGCTTCAACCCTTCGA
CCATCGTCAGGTAGGGGAACAACTGGTCGGCCAGTTCCTGCACCGTCATCCGGTTGCGAA
TCGCCAGTGCGGCCGTCTGGATCAGTTCGCCCGCTTCCGGGGCCACTGCCTGCACGCCGA
TCAGTCGTCCGCTGCCTTCTTCAACCACCAGTTTGATGAAGCCGCGCGTGTCGAAGTTGG
CGAGCGCGCGCGGCACGTTGTCCAGCGTTAGCGTGCGACTATCAGTTTTGATGCCGTCAT
GGTGCGCTTCCGCCTCGCTGTAGCCTACGGTCGCCACTTGCGGGTCGGTGAACACCACGG
CCGGCATCGCGGTCAGGTTCAGGGCCGCGTCACCGCCGGTCATGTTGATCGCGGCGCGAG
TGCCGGCCGCTGCCGCCACATAGACGAACTGCGGCTGGTCGGTGCAGTCGCCTGCGGCGT
AGATGTGTTCCACGCTTGTACGCATGCCGGGGTCGATGACGATAGCGCCTTGCGGGGTGA
GCGTGACGCCCGTCGCATCCAGTGCCAGCTTGCGTGTGTTGGGCGCGCGGCCGGTGGCGA
CCAGCAGCTTGTCGGCGCGCAGTTCGCCGTGCGCCGTGGTGAGCACGAATTCGCCGTCCC
CTTCACCATTGATATACGCGACCTGGCTGGCCTGGGTGTGTTCCCTCACCTCGATGCCCT
CCATGCGGAATGCGGCCGTGACGGCTTCGCCTATAGCTGGGTCTTCGCGGAAGAACAGCG
TGCTGCGAGCCAGGATCGTCACCTTCGCTCCGAGTCGGGCGAACGCCTGCGCCAGCTCCA
GCGCCACCACTGATGAGCCAATCACGGCCAGGCGCTTAGGAATCGTCTCGCTGACCAGCG
CTTCAGTGGAAGTCCAGTACGGAGTGTCTTTCAGGCCGGGAATCGGCGGCACGGCCGGGC
TCGCGCCGGTGGCGATCAGGCAGCGGTCGAATGCCACCACGCGCTCGCCGCCGTCGTTGA
GTTGCACGATCAGGTTGCGATTGTCCTTAAAGCGGGCGGAGCCGTGCAGCACAGTGATCG
CCGGATTGCCCTCCAAGATGCCTTCGTACTTGGCGTGGCGCAGTTCATCGACGCGGGCCT
GCTGCTGGGCCAGCAGCGCCGTGCGCTGGATGGTCGGCGTGGTAGCGGCGATGCCGCCAT
CGAACGGGCTTTCCCGGCGCAGATGGGCGATATGGGCGGCGCGGATCATGATCTTGGACG
GCACACAACCGACATTGACGCAGGTGCCGCCGATGGTGCCGCGCTCGATCAGCGTGACAC
GTGCGCCTTGCTCGACGGCCTTCAGCGCCGCTGCCATCGCGGCCCCGCCGCTGCCGATGA
CGGCGATATGCAATGCGCCGCTGCTACCCGTCTTGTCGTTTCTGCCCAGCAGATCGCGCA
TCTTGTCGAGCAATCCGCCCGGCGTCGAAACTGAGGGGGCATCGGCCAGCGTGGCCCGAT
AACCGAGTCCAGCTACAGCGGCCGTCAGCGCGTCGGGTGACGTGCCGACCTCAATGGCGA
GCTTGGCGCTGCCCTTGGCGTAGGAGACATCCGCTGATTGCACGCCGGGCACTTTCTCCA
GGGCGTCCTTGACATGCACTGCGCACGAGTCGCAAGTCATGCCGGTGATTTTGAGAGTGC
TCATACCATCGTTCCTTATTCGTGTGGGCCGCCGTGTCGCACGGTCAGCCGTCTTTCACA
AGCGCTTGGCGGGGAGTTCGCAGCCGTCCGGTCCGCAACGGCGATGCGCCGGCGACACGA
AGTCCCAGATCGACACCCCAATCATCAAGGCCAGGCCGACGTACATCAGGTTCGCCGTCC
ACCAGTTGCCGAGCAGCCAGACCGTGGCCGCAAACACGATGGCCGGGCCGATCATGCCGA
GCAGACTGCGCAGCCATTGCCGATGACTGAACCAACCCAGCGCGTTCGCCAGGAAGGCCA
GCGCGGCAAACAGCGGCAGCAGGCGGCTGATGAACAGTCCCTCGTACTGGCTCAAGAAGC
CCAGCCCGATGGCCGCGCCGAAGCTGGCGAGGGCTGGAAAGCAGGCGGCGCAGCCCATCG
CGGAAACGACGCTGCCGAGCGCGCCGGTTTTATCGGCAATGCGTGTCATCAGTCCCATGA
AGCGGCTCTCGCTGTTGTCGTTGGCTTGCTGGCTCACTGCTTGACGCTGGACGGATAGCC

GGCGTCTGCGGTGGCCTTGGTCAGCTTCTGTACGCTGGCCTTGGTGTCGTCAAAAGTGAC
GACGGCCTCGCGCTTCTCGAAGCCCACATCGACCTTGCTCACGCCTTCGACCTTGGAGAG
CGCTTTCTTGACTGTGATCGGGCAGGCGGCGCAAGTCATGCCGGGAACCGCTAGCGTGAC
GGTCTGGGTAGCGGCCCACACCGGGGCAACAGCGGCGGCGAGGGCAAGGGAGGCAAACAG
TTTCTTCATGATGAACTCCTGGTTAATAGAAAAATGGAACGACATAGGGAAATCCAAGCG
CGACCAGGACCAGCACGGCCACGATCCAGAAAATCAGCTTGTAGGTGGCGCGCACCTGCG
GAATCGCGCAGACCTCACCTGGCTTGCATGCCTGCACGGGCCGGTAAATCCGCTTCCAGG
CGAAGAACAGCGCCACTAGCGCCGCGCCGATGAACAACGGTCGATAGGGTTCCAGCACCG
TCAGGTTGCCGATCCAAGCACCGGAGAAGCCCAGGGCGACCAGTACTAGCGGCCCCAGGC
AGCAGGTCGATGCAAGAATGGCGGCCAGCCCGCCGGCGAAGAGCGCACCGCGCCCGTTTT
GTGGTTCAGACATACGTTGGCCCTTTTGAATTTGGATTGGATAGCGTAACCTTACTTCCG
TACTCATGTACGGAGTCAAGCGATATGGAAAATAATTTGGAAAACCTGACCATTGGCGTT
TTTGCCAAGGCGGCCGGGGTCAACGTGGAGACAATCCGCTTCTATCAGCGCAAGGGCCTG
TTGCGGGAACCGGACAAGCCTTACGGCAGCATCCGCCGCTATGGGGAGGCGGACGTGGTT
CGGGTGAAATTCGTGAAATCGGCACAGCGGCTGGGGTTCAGTCTGGACGAGATTGCCGAG
CTGTTGCGGCTCGACGATGGCACCCACTGCGAGGAGGCCAGCAGCCTGGCCGAACACAAG
CTCAAGGACGTGCGCGAGAAGATGGCCGACTTGGCGCGCATGGAAACCGTGCTGTCTGAA
CTCGTGTGCGCCTGCCATGCACGAAAGGGGAATGTTTCCTGCCCGTTGATCGCGTCACTA
CAGGGCGAAGCAGGCCTGGCAAGGTCAGCTATGCCTTAGCGTGCTTTATTTAATGAGATG
GTCACTCCCTCCTTCCCGGTACTATGCTGAGGACAGGCTTTCATTCGGAGAACCATCATG
GAAAACATTGCGCTTATTGGTATCGATCTGGGTAAGAACTCTTTCCATATTCATTGTCAG
GATCATCGTGGGAAGGCCGTTTACCGTAAAAAATTCACCCGACCAAAGCTAATCGAATTT
CTGGCGACATGCCCGGCAACAACCATCGCGATGGAAGCCTGTGGCGGTTCTCACTTTATG
GCACGCAAGCTGGCAGAGTTAGGGCATTTTCCAAAGCTGATATCACCGCAATTTGTCCGC
CCATTCGTTAAAAGCAACAAAAATGACTTCGTTGATGCTGAAGCTATCTGTGAAGCAGCA
TCACGTCCATCTATGCGTTTCGTGCAGCCCAGAACCGAATCTCAGCAGGCAATGCGAGCT
CTGCATCGTGTCCGTGAATCCCTGGTTCAGGATAAGGTGAAAACAACTAATCAGATGCAT
GCTTTTCTGCTGGAATTTGGTATCAGCGTTCCGCGAGGTGCTGCCGTTATTAGTCGACTG
AGTACCCTTCTTGAGGACAGTAGTTTGCCTCTTTATCTCAGCCAGTTACTGCTGAAATTA
CAACAGCATTATCACTATCTTGTTGAGCAGATTAAAGATCTGGAATCTCAGTTGAAACGA
AAGTTGGACGAAGATGAGGTTGGACAGCGCTTGCTGAGTATTCCCTGCGTTGGAACGCTG
ACTGCCAGTACTATTTCAACTGAGATTGGCGACGGGAAGCAGTACGCCAGCAGCCGTGAC
TTTGCGGCGGCAACAGGGCTGGTACCCCGACAGTACAGCACGGGAGGTCGGACGACATTG
TTAGGGATTAGCAAGCGGGGCAACAAAAAGATCCGAACTTTGTTGGTTCAGTGTGCCAGG
GTATTCATACAAAAACTGGAACACCAGTCTGGCAAGTTGGCCGACTGGGTCAGGGAGTTG
TTGTGTCGGAAAAGCAACTTTGTCGTCACCTGTGCTCTGGCAAACAAGCTGGCCAGAATA
GCCTGGGCACTGACGGCGCGACAGCAAACTTACGAAGCATAAAGGCAGAAATACACCAGT
TTAAACAATCATTCATCTGGTTTTGCGAATACTGATATTGATGATACTAACGGCCCACCG
GCCTGTTGAGGAACCTGTAAAACGGAAAGGCTCATTGAAGCCGTATATTTTCTGGAGGTT
CATCAGGCGCGGAACTCATCGAGGCGCGGGAATAAAATCCCATTCAGACGCCGGATAGAT
TCAAGCAAGCCAACTTGTCGTCAAAATCGGTGTTGCAAAAACGGGAGTGACCATAGATTC
CGTTTTCTGAGGTGCCCCCTAAAAGGGCAAAAACGCAGTCGTTGATAACGACTCTCCCAC
CTGAAAATAGCTCCAGCATAAAAACTGGAGCTGTAAATGAGATCGAGACCTTCCCTTTTA
CTGATGCGAAACCTGCGGTCATTGGCCGTTGTGGTGCTCGCAACTTTGCCATGCGTTGCT
TTTGCACAATGGCGGGTTGTAGCCGTGAGCACCGAAGTAGACAAAATGCGCTTCAACACC
ATCATAGACGCGCATTCCTTCATGAAGAATTATCGCTCTGGTGAGGAGCAAGGGAAGGGA
ACGCCAGTTGGGGATGCGCTTTATCCGGTTGCCAGTTATGGTGATGGCCGTTATGTCTCC
AGGATCTGTTTTAAGTATCTAGGTGCTACTGGCGATTATGACCCCACTACCTGCACTGGC
GACCCAGCTACGGTCTACTGGCGCTCAACCTATGTTCTGCCGGGTGAGATGGATAAAACA
CCGTTCCTGGACAGGGATCTCGGCTTACCAACAACGACCATGTGTGTAGGGAACCCTATC
CATCTTGGCACCGGCAACAAGTTCCAGGCTGAACTGGATTATCAAAGCGGAGGCTCCGAC
CCTTTCACATTCACCAGATACTACAATAGCCACCTTCCTGATGAAGAGCTCGGCGGCTGG
CGGCATACCTACTCGCGGAGCGTCGAGGTCAACGCCTCGAAGTACGGTGAGAACATGGTT
GTCCTGCACCGGCCAGAAGGGCAACAACTCGACAAGAGAGTGGTCACATAGCTCTCCATA

ATAGATGAGCGACAAAATCTCTTGCTCGTTGATTTTTCCCACCTTAAAAAGGTAATTGAT
TATTGCTGTATCCAGTAGGTCGTGACACCCATTAAGCAGATCCACGATATTGGCCGCAGC
TTCATTTTCCTCTCCGTCATCTCGACCGGCTAATACTGATTCAACCCAAGCCTCAAGGCT
CGGATGTCCCTCAGCGAGCTCTATTTGACGGCACTCGTTCTCGATCAACCCTGTGATTGC
TTCGTTGAGCGTTCCGCCATAAGTCGGCATGTTGAGAACAACTCCAAATCCGGGGATTTC
TTTTTGGATTGGCTTCGCATCCTTGGGAGCTGATTTCAGCCACAGGAAAAACCAGATCCC
AGAGCTTATGTCTTCCGGCTTGACCTGGTTGGCCGCAAGGAATTTGGAAACCTCGTTACA
GATGATGCCATCTGGCGCTGCCATCTCGTAAATACCCGCCATCAGATCTTCAAGGCTCTT
ATACATATTATCTCTCGCACCTGTACATGATGTTTTCACTATCGTTGTTAAGCGTTTTTT
CAGCAAGTATGTTCCAGCGAGACATCTTATACCATTCCATCCAAATCTGTAAAATTACCG
TATTACCGTAATTATGATAATTACGGTAAATAAATTTGAGTTCCTCTTGCTGTTGTTCTA
CTTATTAAAATGTATCTTTGAAAAAGTAGAGTTTTTGGAATAGGATAGGTGGTGCTGAAT
GCCCACTTTTTACAAGGAGCGCGACCAGTGATTTTTGATGTAAGAGCGACCTTTGAAGTG
GCACTCCAGACGGACACCCACCTGGTACTTATCGATCTGGATCAGGGAGCATCAGTTACG
AACGATGCTGATGCAGTGATCGCCTGGTTGGCCGCAAACCTCGAAGGTGGAATTGGAAAA
CGTAAGGTGTACTACCGGGACACCGATGGACGCTTTGATGAGCTCAAGGTTAATGCCGGT
GCTTTTGCCGGATTTGCACCTTGTAGCGAAGGCCAGCAGACCACCTTGGCTGGGATGCTC
GGCCAGTAACTTTGTGTGGAGAACTCGATGTTAAACAAAAACAAATTTGAGAAAGTTTTA
AAGAGAATCCTCGATAAGAACTTTGAGCGCTGCTCTATTTGCAGGAAACCATTCCCTGGA
CCGTGCCATACTTTTGCTGGTTTGGATTCGGACAATAAAGTTCAGAACGTAGGTTCTTGC
TGTCGGACCAGTATTGTGGACTTGCGGCATGGAGGTGTGTATACGACCGCTCCTGTAGAT
ACTCAAGAGGGGCAAAGTCAGGCGCGTGAGCTACTCGCAACACATCCTTGTAAGGGGATG
ATGGGACATGCTTAACAAAAAAGTCTTTTTCATTAGTCAAGCGGAGGCCGAAAGACTGGA
GCCGGTTCCCGGTGCGGCCATGATCTCCATAACTGACCCAGACAAGTCCCCTGCCGCTCT
CGGACAGTGGGGGCAGTTGTACCGCGATAGCTTCTATGACGGCGGCTATTCTGAGAACAC
CATCCACACGATGAAGGCAGCGTTCCGGATGAATTACGCCTCTTACATTGACTCATCCCA
GGCTGAAAAGCTGTCCACCTTCCTGGATGGATTGGTTGGTAGCGGTATCGATCAGATCTT
CGTTCATTGCTATTACGGAGAATCCAGAAGTGGCGCGGTAGCGCTGTACCTCCAGAACAA
ACATGGATTCACTCCGAATAAGCCGATCACCAAGCCCAACCGAACAGTTTACGAATTGCT
GTGCAATCCAACCAAGTTTGAACCACTGATGCAAAGCTACGAAACGCAACATATGGAGGA
GGAACTACCTCTTCACCTCAAAATATGGGATTTTCTTCTCGTTGCGGTCGGGCTTAGGAG
GTAACTTTGCACGACACAAACAATGACAAGGAAGAGCTAGTTTCACATGCGAAAGTAAAT
GTTCCGGCAGAACAAAGCATTCGCGGTGAACTGTTGCCGTCATCGAGCTCGCTCCGGAAC
ATCCAGGACAACCCCGCCGTCGCCTATCTGGTGAGCCTCGGGTCAAAGCGAAGCAGGCAG
ACCATGAGCTCATTCCTCAACATTGTCGCCAAGATGATCGGGTTTCAGAACCTTCGTGAC
TGTGCATGGAGCTCAATGAGGCGGCACCACATATTGGCGGTGCTGGAAATGCTGGGGGAT
GCAGGGAAGGCTCCGGCAACGATCAACACCTACCTGTCAGCGCTCAAAGGGGTGGCTCTT
GAAGCCTGGACGATGAAGCAAATTGATACGGATAGCTTCCAGCACATTAAGCAAGTCCGT
TCAGTACGTGGATCTCGACTTCCTAAAGGGCGGGCACTTGAACGCCATGAGATTCGCAGC
CTCTTTTTCACATGTGAAAGTGATTCAAGCGCCAAAGGGCTTCGGGATGCGGCCATTCTC
GGGGTGCTCCTCGGGTGTGGCTTGCGCCGCTCGGAAATCGTTGCGCTGGACATGGGAAGC
ATGATCTACAAGGACCGCGCTCTCAAGGTTCTTGGCAAGGGCAACAAAGAGAGAATGGCG
TATGTGCCTGGTGGCGCATGGAAAAGACTGGATAAGTGGGTTGAAGAGGTTCGAGGAACG
CATGAAGGGCCTTTATTCCCGAGGATCAGGCGGTTTGATGATGTGACTGGAGAGCGGATG
TCGGATCAGGCCATCTACCACATCTTGGAGACCAGAAGAGTTGAAGCCGGTCTGGAGATG
TTTGCGCCCCATGACCTGCGACGCACCTTTGCCTCCTCGATGCTGGATAACGGTGAGGAT
ATTGTGACCGTGAAGGACGCAATGGGCCACTCAAGCATTGCAACTACCCAGAAATATGAC
AGACGCGGCGATGAGCGTTTGAAGAGAGCGAGCCAACGCCTCGATATAGCGGATTAACAG
CATGAATGATGAAGAACTCGAACTTGCCAGAGCCGAAGCCATGAAAGCCGATAGGTGCTT
TTCAAAAGGACGGCTCAGGGACGAATTTCGGATGAAGCCTAAGCCAGGAGTAGAGCCAGT
TTCGTTCTACAAAAACGGGTATGGTGGCCAATTTGGAGTGTACCGGATAGCAGATTGCCA
ACCAATGCGGAGAAGGGGTTGTTCACCGGCATCACAAAAGCAAATAAGGGCGCAATCCAT
CCTTTCGGTCAAGGCAAGAATGCGTAGCAACCTGGCAAAGGCCTCTGTCATGGCACAAAG
GTGGGTCGCTCTGGAACCTCTGGTTCTCGATACCGAGACGACCGGACTTGGTGAAAGAGA

CCAGGTGATCGAGCTGGCTGTTACTGACATCAGAGGCGCGGTTCTCCTCTGCACCAGATT
ACGGCCAACCGTGGAAATAGACCCTCAGGCAATGGGTGTTCATGGCATCACCGAGACTGA
GCTGTCGAATGAGCCTACGTGGACTCAAGTTGCGCCAGCTCTCGCCCGGCTTTTGTCGGG
CCGTCATCTGGTGATATTTAACTCCAGTTTCGACAGTAGGATGTTGAGGCAAACAGCCAG
TGCATTTGGAGACCAACTCTCTTGGTGGCAAGAGCAGAACTGTCTGTGCGCGATGAAGCT
GGCGGCTGATGCCTTTGGCTCAACCAACCGGCACGGCACAATCTCACTTGCGGATGCCAC
CTGCGAGGCAGGTGTGAGCTGGAAAGGTCGAGCCCATTCAGCAGCGACCGACGCTATTGC
CACCGCCGACTTGGTGACAGAGATAGCCAAAGTCCAACGTGACCTCGTGGTCCAGCTCCA
GGAGCTTCAAAGCAAAGGTAATTTGGAATGACAGAACAATCCTACGGCGAAAGTCTCAAA
TTTTTCTCAGACTGGCAGAAGGACCCCGCGAAACGCACCGGCCTGAATGTGCAACACACG
CTAACCAGAGGTGAATATCCTACGGTCAGCATCGAGATTGCGCCGATCAGGGCATCGGGG
TCCAGCCCAGACTGGAAAAGCAAGATCACGGTGCAGCTCACTCGCGGCGAACTGACGGCG
TTTTGCAGCGTTCTCTTTGGTTTGCGTAGCAAGGCTGAGGGTTCCTATCACGGCGACGCG
AAGAATAAGAGCTTTGCGGTTTACAACAACGGCAAGGCCGGTGTTGCGATCATCCTTAGT
GAGCGAGGAAACCAGCTCCAGAATTTCATCAATGATGACGACCGAATGGAGCTCGCAGTC
TTCGCAGTTCGCCAGCTTTCCAACGCATGGAAAGTTACCCCTTCTGGGGGTCGTCTCAGA
ATTCGGAAAATAAAGCACGCTAAGGCGTAGTCACCCCGTGACTCCCCCGCGCCGATGCAG
CGAGCTTCGTTCCGTCTTGCAGTGACGCAATCAGCGGGCAGGAAACGTTCCCTTTCCGCG
CATGGCAGGCGCACACCAGTTCAGACAGCACGGCCTCCATGCGTGCCAAGTCGGCCATCT
TCTCGCGCACATCCTTGAGCTTGTGCTCGGCCAGGCCGCTGGCTTCCTCGCAATGGGTGC
CATCCTCCAGCCGCAGTAGCTCGGCGATTTCGTCCAGGCTAAAGCCCAGCCGCTGGGCCG
ATTTCACGAACCGCACTCGTGTTACATCCGCCTCGCCATAGCGGCGAATGCTGCCATAGG
GCTTGTCTGGCTCCGGCAGCAGGCCCTTGCGCTGGTAGAACCGGATGGTCTCCACATTGA
CCCCGGCCGCCTTGGCAAAAACGCCAATGGTCAGATTCTCAAAATTAATTTGCATATCGC
TTGACTCCGTACATAACTACGGAAGTAAGCTTAAGCTATCCAAACCAAATTTGAAAGGAC
AAGCGTATGTCTGAACCACAAAAGTCTGAACCACAAAACGGGCGCGGCGCGCTCTTCGCC
GGTGGGCTGGCCGCCATTCTTGCGTCGGCCTGCTGCCTGGGGCCGCTGGTTTTGATCGCC
TTGGGGTTCAGCGGGGCATGGATCGGCAACCTGACGGTGCTGGAACCCTATCGCCCGATC
TTCATCGGCGCAGCGCTGGTCGCGCTGTTTTTCGCCTGGCGGCGCATCTACCGCCCGGCG
CAAGCCTGCAAACCGGGTGAGGTCTGCGCGATTCCCCAAGTGCGAGCTACTTACAAGCTC
ATTTTCTGGATCGTGGCCGCGCTGGTCCTGGTCTCGCTCGGATTTCCCTACGTCATGCCA
TTTTTCTATTAATCACAGGAGTTCATCATGAAAAAACTGTTTGCCGCCCTCGCCCTCGCT
GCCGTTGTTGCCCCCGTGTGGGCCGCCACCCAGACCGTCACGCTGTCCGTGCCTGGCATG
ACCTGCGCCTCTTGCCCGATCACTGTCAAGCACGCGCTTTCCAAGGTTGAGGGCGTGAGC
AAGACCGACGTAAGTTTCGACAAGCGCCAGGCCGTCGTCACCTTCGACGATGCCAAGACC
AACGTCCAGAAGTTGACCAAGGCGACCGAGGACGCGGGCTATCCGTCCAGCCTCAAACGC
TGATCCGTTAACCGAACTCGGGAGCGACACATGGGACTCATCACGCGCATCGCTGGCAAA
ACCGGCGCGCTCGGCAGCGTCGTTTCCGCGATGGGCTGCGCCGCCTGTTTTCCTGCCATC
GCCAGCTTTGGCGCGGCCATCGGACTGGGCTTCTTGAGCCAGTACGAGGGGCTATTCATT
GGCATCCTGCTGCCGATGTTCGCCGGCATCGCGTTACTCGCCAATGCTATCGCTTGGCTC
AATCATCGACAGTGGCGACGCACGGCGCTCGGCACGATAGGCCCGATCTTGGTGCTGGCA
GCGGTGTTTTTAATGCGGGCTTACGGCTGGCAGAGCGGTGGACTGCTCTATGTCGGCCTG
GCCTTGATGGTTGGGGTGTCGGTCTGGGATTTCATCTCGCCAGCACATCGCCGCTGCGGG
CCGGACAGCTGTGAATTGCCAGAACAACGTGGCTGACGGCAACAGCCGTAGCCACCACAG
AAAAGGAAAAATACATGACCACCCTGAAAATCACCGGGATGACCTGCGACTCGTGCGCGG
CTCACGTCAAGGAAGCCTTGGAGAAAGTGCCCGGCGTGCAATCGGCGCTGGTGTCCTATC
CGAAGGGCACAGCGCAACTCGCCATTGAGGCGGGCACGTCATCGGATGCGCTGACTACCG
CCGTGGCCGGACTGGGCTACGAGGCAACGCTTGCCGATGCGCCACCGACGGACAACCGCG
CCGGCCTGCTCGACAAGATGCGCGGCTGGATAGGGGCCGCTGATAAGCCCAGTGGCAACG
AACGCCCGTTGCAGGTCGTCGTCATTGGTAGCGGTGGAGCCGCGATGGCGGCAGCACTGA
AGGCCGTCGAGCAAGGCGCGCAGGTCACGCTGATTGAGCGCGGCACCATCGGCGGCACCT
GCGTCAACGTCGGTTGTGTGCCGTCCAAGATCATGATCCGCGCCGCCCACATCGCCCATC
TGCGCCGGGAAAGCCCATTCGACGGCGGCATGCCACCCACACCGCCGACGATCTTGCGCG
AGCGGCTGCTGGCCCAGCAGCAGGCCCGTGTCGAAGAACTCCGTCATGCCAAGTACGAAG

GCATCCTGGACGGCAATTCAGCCATCACCGTTCTGCACGGTGAAGCGCGTTTCAAGGACG
ACCAGAGCCTTATCGTTAGTTTGAACGAGGGTGGCGAGCGCGTCGTGATGTTCGACCGCT
GCCTGGTCGCCACGGGTGCCAGCCCGGCGGTCCCGCCGATTCCGGGCTTGAAAGAGTCAC
CCTACTGGACTTCCACCGAGGCCCTGGCGAGCGACACCATTCCCGAACGCCTTGCCGTAA
TCGGCTCGTCGGTGGTGGCGCTGGAGCTGGCGCAAGCCTTTGCCCGGCTGGGCAGCAAGG
TCACGGCCCTGGCGCGCAATACCTTGTTCTTCCGTGAAGACCCGGCCATCGGCGAGGCGG
TGACAGCCGCTTTCCGTGCCGAGGGCATCGAGGTGCTGGAGCACACGCAAGCCAGCCAGG
TCGCCCATATGGACGGTGAATTCGTGCTGACCACCACGCACGGTGAATTGCGCGCCGACA
AGCTGCTGGTCGCCACCGGCCGGACACCGAACACGCGCAGCCTGGCATTGGAAGCGGCGG
GGGTAGCCGTCAATGCGCAGGGGGCCATCGTCATCGACAAGGGCATGCGCACCAGTAGCC
CGAACATCTACGCGGCCGGCGACTGCACCGACCAGCCGCAGTTCGTCTATGTGGCGGCAG
CGGCCGGCACTCGTGCGGCGATCAACATGACTGGCGGCGATGCGGCCCTGGACCTGACCG
CAATGCCGGCCGTGGTGTTCACCGACCCGCAGGTCGCCACCGTGGGCTACAGCGAGGCGG
AAGCACATCACGACGGGATCGAGACCGACAGTCGCCTGCTAACACTGGATAACGTGCCGC
GTGCGCTTGCCAACTTCGACACACGCGGCTTCATCAAGCTGGTCATCGAGGAAGGTAGCG
GACGGCTCATCGGCGTGCAAGCGGTGGCCCCGGAAGCGGGTGAACTGATCCAGACGGCGG
TGCTCGCCATTCGCAACCGTATGACCGTGCAGGAACTGGCCGACCAATTGTTCCCCTACC
TGACCATGGTCGAAGGGCTGAAGCTCGCGGCGCAGACCTTCAGCAAGGACGTGAAGCAGC
TTTCGTGCTGCGCCGGATGAGGAAAAGGAGGTGTTCAATGAGCGCCTACACAGTGTCCCG
GCTGGCCCTTGATGCCGGGGTGAGCGTGCATATCGTGCGCGACTACCTGCTGCGCGGATT
GCTACGGCCGGTCGCGTACACCACGGGCGGCTACGGCTTGTTCGATGACACCGCGTTGCA
ACGGCTGCGCTTTGTACGGGCTGCCTTCGAAGCGGGTATCGGCCTGGACGCACTGGCGCG
GCTGTGCCGGGCGCTGGATGCTGCGGACGGTGACGGTGCGTCTGCGCAGCTTGCCGTGTT
GCGGCAACTCGTCGAGCGTCGGCGCGAGGCCCTGGCCAGCCTCGAAATGCAACTGGCCGC
CATGCCAACCGAACCGGCACAGCACGCGGAGAGTCTGCCATGAACAGCCCAGAGCACTTG
CCGTCTGAGACGCACAAACCGATCACCGGCTACTTGTGGGGCGCGCTGGCCGTGCTCACC
TGTCCCTGCCATTTGCCGATTCTCGCCATTGTGCTAGCCGGCACGACGGCCGGCGCGTTC
ATCGGGGAGCACTGGGGTATTGCAGCCCTCACGCTGACCGGCTTGTTTGTCCTGTCTGTG
ACGCGGCTGCTGCGGGCCTTCAAGGGAAGATCATGACCGCTTCCCAGCCAGCCGAGAGTG
GGCAGCTTTGAGCTTCGCTACCAATCTGGAGGAGTACCACCATGAACGCAAACGCCCCGA
ACACTGCCAGTTGCACCACCTGCTGCGTATGCTGCAAAGAAATTCCGCTCGATGCCGCCT
TCACCCCGGAAGGCGCGGAATACGTCGAACATTTCTGCGGGCTGGATTGCTATGAACGCT
TCCAGGCACGCGCCAAGGCCGCGACAGAATCTGACATTGCGCCTGTCCCTGGCGGTTCGC
AGCCGTCAGATTGAGGCATACCCTAACTTGATGTCAGATGCCATGTGTCATTTTCAGAAG
ACGACTGCACCAGTTGATTGGGCGTAATGGCTGTTGTGCAGCCAGCTCCTGACAGTTCAA
TATCAGAAGTGATCTGCACCAATCTCGACTATGCTCAATACTCGTGTGCACCAAAGCGAG
GTGAGCATGGCGACGGAGGCTCTGTTGCAAAGATTGGCGGCAGTCAGAGGTAGGCTGTCG
CTCTGCGCCGATCAGGCGGCTGCTGCGAAATGGTGGTTGAGCATGCCCATGGCCTCCGTC
AGCGCCGAGGGCCCAATGCCAAAAGCTCTCTCCACAAGGCGCACCTCGCCCCTGATGCCG
GGCTGCAGGCACCAGGGGCGAGCCTGTCCTTTGCGCAGGGCTCGCATGACTTCGAATCCC
TTGATCGTGGCATAGGCCGTGGGGATCGATTTGAAACCGCGCACCGGCTTGATCAGTATC
TTGAGCTTTCCGTGATCGGCCTCGATCACGTTATTGAGATACTTCACCTGCCGGTGGGCC
GTCTCCCGGTCCAGCTTTCCTTCGCGCTTCAATTCGGTGATCGCTGCACCATAGCTCGGC
GCTTTGTCGGTATTGAGCGTGGCAGGCTTTTCCCAGTGCTTCAGGCCTCGCAGGGCCTTG
CCCAGGAACCGCTTCGCTGCCTTGGCGCTGCGGGTCGGCGACAGGTAGAAATCGATCGTG
TCGCCCCGCTTGTCGACTGCCCGGTACAGGTAGGTCCACTTGCCCCGCACCTTGACGTAG
GTTTCATCCAGGCGCCAGCTCGGATCAAAGCCACGCCGCCAGAACCAGCGCAGCCGCTTC
TCCATCTCCGGGGCGTAGCACTGGACCCAGCGATAGATCGTCGTATGGTCGACCGAAATG
CCGCGTTCCGCCAGCATTTCCTCAAGGTCGCGATAGCTGATCGGATAGCGACAATACCAG
CGCACCGCCCACAGGATCACATCACCCTGGAAATGGCGCCACTTGAAATCCGTCATCGTT
CCGTCCGTCCAATCTCCGCCAAGCATGCTCAAGCTTCACGATTTTTGCAACAGAGCCCAC
ACGGGCACTGTTGCAAAGTTAGCGATGAGGCAGCCTTTTGTCTTATTCAAAGGCCTTACA
TTTCAAAAACTCTGCTTACCAGGCGCATTTCGCCCAGGGGATCACCATAATAAAATGCTG
AGGCCTGGCCTTTGCGTAGTGCACGCATCACCTCAATACCTTTGATGGTGGCGTAAGCCG

TCTTCATGGATTTAAATCCCAGCGTGGCGCCGATTATCCGTTTCAGTTTGCCATGATCGC
ATTCAATCACGTTGTTCCGGTACTTAATCTGTCGGTGTTCAACGTCAGACGGGCACCGGC
CTTCGCGTTTGAGCAGAGCAAGCGCGCGACCATAGGCGGGCGCTTTATCCGTGTTGATGA
ATCGCGGGATCTGCCACTTCTTCACGTTGTTGAGGATTTTACCCAGAAACCGGTATGCAG
CTTTGCTGTTACGACGGGAGGAGAGATAAAAATCGACAGTGCGGCCCCGGCTGTCGACGG
CCCGGTACAGATACGCCCAGCGGCCATTGACCTTCACGTAGGTTTCATCCATGTGCCACG
GGCAAAGATCGGAAGGGTTACGCCAGTACCAGCGCAGCCGTTTTTCCATTTCAGGCGCAT
AACGCTGAACCCAGCGGTAAATCGTGGAGTGATCGACATTCACTCCGCGTTCAGCCAGCA
TCTCCTGCAGCTCACGGTAACTGATGCCGTATTTGCAGTACCAGCGTACGGCCCACAGAA
TGATGTCACGCTGAAAATGCCGGCCTTTGAATGGGTTCATGTGCAGCTCCATCAGCAAAA
GGGGATGATAAGTTTATCACCACCGACTATTTGCAACAGTGCCGCCTGGGACGTCGCGCG
GATCGACCGGACATTCGCGGAGACCTTCGTCCTGAGATCCGAGCTCCTAGTCGCGAGTTG
CAGCGACGGCATCGTCGGCTGTTGCACCTTGTCGGCCGAGGATCCCGAGTTCTGGCCCGA
CGCCCTCAAGGGGGAGGCCGCATATCTGCACAAGCTCGCGGTGCGACGGACACATGCGGG
CCGGGGTGTCAGCTCCGCGCTGATCGAGGCTTGCCGCCATGCCGCGCGAACGCAGGGGTG
CGCCAAGCTGCGGCTCGACTGCCACCCGAACCTGCGTGGCCTATACGAGCGGCTCGGATT
CACCCACGTCGACACTTTCAATCCCGGCTGGGATCCAACCTTCATCGCAGAACGCCTAGA
ACTCGAAATCTAACGTCCGTTCGGGCATCGAGGTCCATGTCGGGGTGGGACGGGCCCGTG
GCTTCAAGATCACTTGCAGTCCGACCGCGATGTCTTGGTTGCGCGAGAGGTTGTCGATAT
CCTCCACTTCCATCATCAACCCTGGATAATGCCGCCGCCGTCATCGCCGCCGACGCCCGT
GCCGGGCTTTTCGGGCCTGTCAGGCTTGCTCGGCCTTCAGCCGGCACTGTTGCAAATAGT
CGGTGGTGATAAACTTATCATCCCCTTTTGCTGATGGAGCTGCACATGAACCCATTCAAA
GGCCGGCATTTTCAGCGTGACATCATTCTGTGGGCCGTACGCTGGTACTGCAAATACGGC
ATCAGTTACCGTGAGCTGCAGGAGATGCTGGCTGAACGCGGAGTGAATGTCGATCACTCC
ACGATTTACCGCTGGGTTCAGCGTTATGCGCCTGAAATGGAAAAACGGCTGCGCTGGTAC
TGGCGTAACCCTTCCGATCTTTGCCCGTGGCACATGGATGAAACCTACGTGAAGGTCAAT
GGCCGCTGGGCGTATCTGTACCGGGCCGTCGACAGCCGGGGCCGCACTGTCGATTTTTAT
CTCTCCTCCCGTCGTAACAGCAAAGCTGCATACCGGTTTCTGGGTAAAATCCTCAACAAC
GTGAAGAAGTGGCAGATCCCGCGATTCATCAACACGGATAAAGCGCCCGCCTATGGTCGC
GCGCTTGCTCTGCTCAAACGCGAAGGCCGGTGCCCGTCTGACGTTGAACACCGACAGATT
AAGTACCGGAACAACGTGATTGAATGCGATCATGGCAAACTGAAACGGATAATCGGCGCC
ACGCTGGGATTTAAATCCATGAAGACGGCTTACGCCACCATCAAAGGTATTGAGGTGATG
CGTGCACTACGCAAAGGCCAGGCCTCAGCATTTTATTATGGTGATCCCCTGGGCGAAATG
CGCCTGGTAAGCAGAGTTTTTGAAATGTAAGGCCTTTGAATAAGACAAAAGGCTGCCTCA
TCGCTAACTTTGCAACAGTGCCAATTCATTTTCTTCCGGGCAAAAATGCCATTTCACACA
TCAGCTTTGCAGGACGACCTGCAAACGCTTCATTCACCGGGGACGGCCCCATAGTTCTGG
AGCCTGATATGTCCTGGATCGTTTTATTAATTGCAGGTTTGCTCGAAGTCGTATGGGCGA
TTGGCCTGAAATACACCCACGGTTTTACGCGTCTTACGCCAAGCATTATCACTATTGCGG
CGATGATCGTCAGTATCGCCATGCTCTCCTGGGCAATGCGCACGTTGCCTGTAGGAACCG
CTTATGCGGTCTGGACCGGTATTGGCGCTGTTGGGGCGGCTATTACAGGGATTTTGCTGC
TGGGTGAGTCTGCCAGCCCGGCACGTTTGTTGAGCCTTGGGCTGATCGTTGCTGGTATTA
TTGGTCTGAAGCTGAGCACTCACTAAGCGCCAGGCTGTTTCACCCAAATCAGTTTGTTCA
CTTCAAACCCTTCCCGGGTCGCGATGGCTAACATTTGCTGTTTCATTTCATCTGAAATAG
TAGGGGTCCGTGAAAGGATCCACAGGTAGTCACGATCCGGGCCACAAACCAGCGCGTGAC
GATATTCCCGGTCGAGTGCAATTACGTTATACCCGCCATAGAAGGGGCCGAAAAAAGAAA
CCTTAAGCGCGGCTCTGCCTGGGTCGCCGGTGAAATAGGCTTTCCCTTCCGTTTTCTGCC
ACATCTCCCTGTCAGGGTTATAGCCCTTGTTAATAACGTTGATCCCGCCGTCGTCGCGCA
AGCTGTATGTTGCGGTCACTTTATCCAATCCGCGCTCGAAACGATGGTCGAAGCGCGCAA
TTTCATACCAGGTTCCCAGATAGCGTTTGGCATCAAAGTTATTTACCACGGTAACGCCTT
TCAGCGGTGTCGGGGAGCTACACGCGACAACCAGGAATGCAGCCGTAACTGCAGCAACGA
CGGGCAAAATGCGCATGGGATTTTCCTTGCTGTATTTTTGTTAAGTGTAGATGACAACAG
GAAAAAAAGAGAAAGAAAGGAGGCCCAATATCCTGGGCCTCATCGTCAGTTATTGCAGCT
TTTCAAGAATGCGCCAGGCCGCCTCGACACGGACAGGGTTAGGATAGCTTTTGTTTGCCA
GCATCACGATGCCAAGGTTTTTTTCTGGAACGAAGGCTACGTAGCTGCCAAATCCTCCAG

TGGATCCCGTTTTATGCACCCATGAGGCTTTCACTGCCGGGGCGGGCGGGTTTACCTCAA
CGGCGGGAAGCGCTGCCAATGCCACTTTGCTGTCGCTACCGTTGATGATCGAATCAGCTT
TCAGCGGCCAGTTCAGCATCTCCCAGCCTAATCCCTGGTACATATCGCCAATACGCCAGT
AGCGAGACTGCGCAAGCGCAATGCCCTGCTGGAGCGTTTTCTCCTGAACGCGGCTGGCGT
CCATGTTGACCTGAACCCAGCGTGCCATATCGGTAACGTTGGATTTCACGCCATAGGCTT
CGGCGTCAAGTTGTCCCGGAGAAACGTGTACAGGTTTCCCTTCGCGATAGCCCCAGGCAT
AATCTTTTTGTTCGTTCTGCGGAACTGTAATCCAGGTATGCGCCAGTTTTAATGGTTGCA
GGACGCGTCTGGTCATTGCCTCTTCGTAACTCATTCCTGAGGGTTTCACCGCCAGCGCGC
CAAACAGACCAATGCTGGAGTTAGCGTAAAGACGCTTAGCGCCCGGGGCCCATTGCGGCT
GCCAGTTTTGATAAAAACGCAGTAATGCGGCTTTATCAGTAACGTCATCGGGGATCTGCA
GCGGTAGGCCGCCTGCCGTATAGGTGGCTAAGTGCAGCAGGCTGATACCCTGCCACTGTT
TGCCTGTCAGTTCTGGCCAGTATTTCGTGACCGGATCGCTGAGCTTAATTTCGCCGCGGG
CGATAGCATCGCCGCCCAACACGCCGTTAAACGTCTTACTGACCGACCCTAGCTCAAACA
GCGTTTGCTGCGTGACTGGGTGGTTATTGGCGATATCGGCTTTACCCCAGGTGAAATAAT
AGGGTTTTCCCTGGTAGATAATGGCAACGGCCATACCCGGAATAGCCTGCTCCTGCATCA
ACGGGGTGATGGTGCGATTAACGATATCGGCAATCTGTTGTTCTGTTTTGGCGGAGGCAA
ACGTGGAGAAAGAGGCTGTCAGCAGCAGAGCGCAGCATAACGATTTTTTCATCATGAAAT
CAGTTCCGTAATTAAAAGCAAAAGGGTGTCCGGGCCCGTCAGACGCAATCAGTGTGTTTG
ATTTGCACCGTGTTGACAAACGGTTAAATTTAGCAGCAGATATAAGTTTTTCTAACAGGC
CGGATGATGACGCGTAGCTATATCCCTCTTAACTCGCTGCGGGCTTTTGAAGCCGCGGCC
AGACATCTCAGTTTTACCCGCGCCGCGATTGAGCTCAACGTGACGCATTCTGCCATCAGT
CAGCATGTAAAAGCGCTGGAGCAGCAGCTAAATTGTCAGCTTTTTGTGCGCGGTTCTCGT
GGATTAATGTTGACGACTGAAGGGGAAAGTCTGTTACCTGTACTTAATGACTCTTTCGAT
CGTATGGCGGGGATGCTGGATCGTTTTGCCACCAAACAGACCCAGGAAAAACTTAAAATT
GGCGTGGTGGGGACCTTTGCGGTTGGGTGTCTGTTTCCTCTACTGGGCGATTTTAAGCGC
AGTTACCCGCATATTGATTTGCATATTTCTACCCATAATAACCGCGTGAATCCCGCTGCC
GAAGGGCTGGATTATACCATTCGCTACGGAGGGGGAGCCTGGCACGATACCGACGCGCAA
TATTTATGTAGCGCGTTGATGTCTCCACTGTGTTCGCCAACATTGGCTTCGCAGATTCAA
ACGCCTGCGGACATCCTGAAATTTCCGTTATTACGATCGTATCGGCGGGATGAATGGACG
CTTTGGATGCAGGCCGCCGGAGAGGCGCCCCCGTCACCGACGCACAACGTGGTGGTGTTT
GATTCGTCCGTCACCATGCTGGAAGCCGCGCAGGCGGGGATCGGTGTGGCAATTGCACCA
GTCAGAATGTTCACGCATTTACTCAGTAGGGAACGAATCGTTCAACCGTTTTTAACGCAG
ATTGATTTGGGAAGTTACTGGATAACGCGTTTGCAATCGCGTCCGGAGACACCCGCGATG
CGTGAATTTTCCCGTTGGTTGACAGGAATGCTGCACAAATAAAAAGGCCCGCCAATGGCG
GGCCTGATAATTAACGCATGTACTTAGATGGTCAGAATGCCAATAGCCGTTACGACGGTC
AGGATTGCTGCCAGTCCGTAGAATACCCATTTGCCAGCAGGCACGTGGATTTTCAGGTCA
TGCATCGCGTGGTGCATACGGTGTAAACCACACCACAGCGGCAGAACGATCATCAGGAAC
AGAAACGCACGTCCGATGAAGCTCTGAGCGAATGCCAGTACGCGTTCATAGCTGAAAGCA
TCAGGAACAAAGCCCAGCGGCAGCATAATGCCTACCAGCAGGATCATTACTGGCGCAATG
ATTGCGCTCCACATACCGCCTGCACCAAACAGGCCCCAGAATACGGGTTCGTCAGAGCGT
TTTGGATTTGGATTAATCATTCCGGACTCCTTACCAGAACAGGGCAACAAACAGTATGAC
TACTGTCGCAACCACAGTAACTGCCCAGAGACCTTTGATGATCGGCTCTGGCCCCATTTT
TTCGTCTTTTACGATGATGTTGGCTGCTTTTGGCGCCAGCTCAAACCAGGTTTTGGTATG
CAGCAGCGCTGCTGCCAGGGCGATAATGTTAAGGATTACCACCACCGGGTTTTGCAAAAA
GGCGACAAAGCCCATCCAGGACTCTGCACCATGCTTGAGTGCAAACAGGCCGATAATCAG
TTCAATGCTGAACCAGACTGCCGGAACTGCCGTGCCTTCACGCAGCATGTAAAAACGATA
AAATGGCAGCTTTTTCCACCAGGTGGACGTCATTGGCCGCACATAGGCTTTACGTTTAGT
CGTCATCGTGCACTCCTTAGCGTGGTTTCAGGGTAGCGATAAGAAAGTCTTTTGAACTTT
CAACTTTACCCTGCTGAATGGCCGCAGCCGGATCGACGTGTTTCGGGCAGACTTCGGAGC
AGTAGCCCACGAAAGTACAGGTCCAGACGCCGTTCGGGCTGTTCAACTGAGCCATACGCT
CCTTCTTACCGTGGTCGCGGCTATCTTCGTTGTAACGATGCGCCAGCGTAATCGCAGCCG
GTCCAATGAACGGCACTGTTGCAAATAGTCGGTGGTGATAAACTTATCATCCCCTTTTGC
TGATGGAGCTGCACATGAACCCATTCAAAGGCCGGCATTTTCAGCGTGACATCATTCTGT
GGGCCGTACGCTGGTACTGCAAATACGGCATCAGTTACCGTGAGCTGCAGGAGATGCTGG

CTGAACGCGGAGTGAATGTCGATCACTCCACGATTTACCGCTGGGTTCAGCGTTATGCGC
CTGAAATGGAAAAACGGCTGCGCTGGTACTGGCGTAACCCTTCCGATCTTTGCCCGTGGC
ACATGGATGAAACCTACGTGAAGGTCAATGGCCGCTGGGCGTATCTGTACCGGGCCGTCG
ACAGCCGGGGCCGCACTGTCGATTTTTATCTCTCCTCCCGTCGTAACAGCAAAGCTGCAT
ACCGGTTTCTGGGTAAAATCCTCAACAACGTGAAGAAGTGGCAGATCCCGCGATTCATCA
ACACGGATAAAGCGCCCGCCTATGGTCGCGCGCTTGCTCTGCTCAAACGCGAAGGCCGGT
GCCCGTCTGACGTTGAACACCGACAGATTAAGTACCGGAACAACGTGATTGAATGCGATC
ATGGCAAACTGAAACGGATAATCGGCGCCACGCTGGGATTTAAATCCATGAAGACGGCTT
ACGCCACCATCAAAGGTATTGAGGTGATGCGTGCACTACGCAAAGGCCAGGCCTCAGCAT
TTTATTATGGTGATCCCCTGGGCGAAATGCGCCTGGTAAGCAGAGTTTTTGAAATGTAAG
GCCTTTGAATAAGACAAAAGGCTGCCTCATCGCTAACTTTGCAACAGTGCCGGGAGGACT
TTCCGCAACCGGCCGTTCGATGCGGCACCGATGGCCTTCGCGCAGGGGTAGTGAATCCGC
CAGGATTGACTTGCGCTGCCCTACCTCTCACTAGTGAGGGGCGGCAGCGCATCAAGCGGT
GAGCGCACTCCGGCACCGCCAACTTTCAGCACATGCGTGTAAATCATCGTCGTAGAGACG
TCGGAATGGCCGAGCAGATCCTGCACGGTTCGAATGTCGTAACCGCTGCGGAGCAAGGCC
GTCGCGAACGAGTGGCGGAGGGTGTGCGGTGTGGCGGGCTTCGTGATGCCTGCTTGTTCT
ACGGCACGTTTGAAGGCGCGCTGAAAGGTCTGGTCATACATGTGATGGCGACGCACGACA
CCGCTCCGTGGATCGGTCGAATGCGTGTGCTGCGCAAAAACCCAGAACCACGGCCAGGAA
TGCCCGGCGCGCGGATACTTCCGCTCAAGGGCGTCGGGAAGCGCAACGCCGCTGCGGCCC
TCGGCCTGGTCCTTCAGCCACCATGCCCGTGCACGCGACAGCTGCTCGCGCAGGCTGGGT
GCCAAGCTCTCGGGTAACATCAAGGCCCGATCCTTGGAGCCCTTGCCCTCCCGCACGATG
ATCGTGCCGTGATCGAAATCCAGATCCTTGACCCGCAGTTGCAAACCCTCACTGATCCGC
ATGCCCGTTCCATACAGAAGCTGGGCGAACAAACGATGCTCGCCTTCCAGAAAACCGAGG
ATGCGAACCACTTCATCCGGGGTCAGCACCACCGGCAAGCGCCGCGACGGCCGAGGTCTT
CCGATCTCCTGAAGCCAGGGCAGATCCGTGCACAGCACCTTGCCGTAGAAGAACAGCAAG
GCCGCCAATGCCTGACGATGCGTGGAGACCGAAACCTTGCGCTCGTTCGCCAGCCAGGAC
AGAAATGCCTCGACTTCGCTGCTGCCCAAGGTTGCCGGGTGACGCACACCGTGGAAACGG
ATGAAGGCACGAACCCAGTTGACATAAGCCTGTTCGGTTCGTAAACTGTAATGCAAGTAG
CGTATGCGCTCACGCAACTGGTCCAGAACCTTGACCGAACGCAGCGGTGGTAACGGCGCA
GTGGCGGTTTTCATGGCTTGTTATGACTGTTTTTTTGTACAGTCTATGCCTCGGGCATCC
AAGCAGCAAGCGCGTTACGCCGTGGGTCGATGTTTGATGTTATGGAGCAGCAACGATGTT
ACGCAGCAGCAACGATGTTACGCAGCAGGGCAGTCGCCCTAAAACAAAGTTAGGCATCAC
AAAGTACAGCATCGTGACCAACAGCAACGATTCCGTCACACTGCGCCTCATGACTGAGCA
TGACCTTGCGATGCTCTATGAGTGGCTAAATCGATCTCATATCGTCGAGTGGTGGGGCGG
AGAAGAAGCACGCCCGACACTTGCTGACGTACAGGAACAGTACTTGCCAAGCGTTTTAGC
GCAAGAGTCCGTCACTCCATACATTGCAATGCTGAATGGAGAGCCGATTGGGTATGCCCA
GTCGTACGTTGCTCTTGGAAGCGGGGACGGATGGTGGGAAGAAGAAACCGATCCAGGAGT
ACGCGGAATAGACCAGTTACTGGCGAATGCATCACAACTGGGCAAAGGCTTGGGAACCAA
GCTGGTTCGAGCTCTGGTTGAGTTGCTGTTCAATGATCCCGAGGTCACCAAGATCCAAAC
GGACCCGTCGCCGAGCAACTTGCGAGCGATCCGATGCTACGAGAAAGCGGGGTTTGAGAG
GCAAGGTACCGTAACCACCCCAGATGGTCCAGCCGTGTACATGGTTCAAACACGCCAGGC
ATTCGAGCGAACACGCAGTGATGCCTAACCCTTCCATCGAGGGGGACGTCCAAGGGCTGG
CGCCCTTGGCCGCCCCTCATGTCAAACGTTAGGTGGCTCAAGTATGGGCATCATTCGCAC
ATGTAGGCTCGGCCCTGACCAAGTCAAATCCATGAGGGCTGCTCTTGATCTTTTCGGTCG
TGAGTTCGGAGACGTAGCCACCTACTCCCAACATCAGCCGGACTCCGATTACCTCGGGAA
CTTGCTCCGTAGTAAGACATTCATCGCGCTTGCTGCCTTCGACCAAGAAGCGGTTGTTGG
CGCTCTCGCGGCTTACGTTCTGCCAAAGTTTGAGCAGGCGCGTAGTGAGATCTATATCTA
TGATCTCGCAGTCTCCGGCGAGCACCGGAGGCAAGGCATTGCCACCGCGCTCATCAATCT
CCTCAAGCATGAGGCCAACGCGCTTGGTGCTTATGTGATCTACGTGCAAGCAGATTACGG
TGACGATCCCGCAGTGGCTCTCTATACAAAGTTGGGCATACGGGAAGAAGTGATGCACTT
TGATATCGACCCAAGTACCGCCACCTAACAATTCGTTCAAGCCGAGATCGCTTCGCGGCC
GCGGAGTTGTTCGGAAAAATTGTCACAACGCCGCGGCCGCAAAGCGCTCCGGCTTAACTC
AGGCGTTGGGCAACAAGAAAACCGATATGAACGTACGCACTTGCACTGAATCTGACGTCG
CCTCTATCGCAGTCGTATTTACTGAGTCTATTCATGTACTTGGAGCGTCTCACTATGACG

CTTCGCAAAGGAATGCGTGGGCACCGCGTCCCGCAGATATAGAGGCTTGGTCAGCTCGCT
TATCTGGCCTACAGACTCTTCTAGCAATTGAGGGAGATGCGGTTATCGGGTTCATCTCTT
ACGAGCTTAGCGGCCACATCGAGTTTCTTTACACCGCACCGGGTTCCGAGCGTCGGGGCG
TCGCGTCTGTTCTGTACCGTGAGGTTGAGAAAGCCCTCCCAGGTGTTTCGCTCTTCACAG
AAGCCAGTCTGGTCGCCAAGCCCTTTTTCCTGCGGCATGGTTTCAGTGTAGTTGAGGAGC
AAAATGTCTCCCGTGGAGGCGTCATGTTCCGTAGGTATGCAATGCGCAAGGCAGTTGTCG
CCCAACATGGCGCTCAAGCCGACCGGCCAGCCCTGCGGGCTGTCCGTCGGCTTAGCTAGG
GCGTTAGAGCAAAAATGAACTGCGCACGCTGTGGCCGCCAGAATTTCGTAGATGCACATC
GTTGCGCTCACTGCGGCCACCACTTCTTTGACGGTAGTGACGTGGTGAATCTTCTAGACA
AGGCTCAGAAGCGCACTTGGTTAAGGCCGCGAAACATCGCCATATTCCTTGCGGCCTGGG
CGCTACTGGTGGTGGAGATCGTCACCTACGCGGGTAAAGCGCCAAGTTCTCCTCAGGGCT
GGGCGGCGCTCATACTGCTCGGGCCTATTGTGTACGTCGTACTAGCAACCTGTGCAGATA
CAATTTTTCGAAAGCCATGAATACAGTAAATCGCTCATGCTCTAACCAATGCTTCCAGCC
GACGCTACGGCCTTACGGCCTCCGCGCGTCTGAAGCTGGGCGTTAAACATCATGAGGGAA
GCGGTGATCGCCGAAGTATCGACTCAACTATCAGAGGTAGTTGGCGTCATCGAGCGCCAT
CTCGAACCGACGTTGCTGGCCGTACATTTGTACGGCTCCGCAGTGGATGGCGGCCTGAAG
CCACACAGTGATATTGATTTGCTGGTTACGGTGACCGTAAGGCTTGATGAAACAACGCGG
CGAGCTTTGATCAACGACCTTTTGGAAACTTCGGCTTCCCCTGGAGAGAGCGAGATTCTC
CGCGCTGTAGAAGTCACCATTGTTGTGCACGACGACATCATTCCGTGGCGTTATCCAGCT
AAGCGCGAACTGCAATTTGGAGAATGGCAGCGCAATGACATTCTTGCAGGTATCTTCGAG
CCAGCCACGATCGACATTGATCTGGCTATCTTGCTGACAAAAGCAAGAGAACATAGCGTT
GCCTTGGTAGGTCCAGCGGCGGAGGAACTCTTTGATCCGGTTCCTGAACAGGATCTATTT
GAGGCGCTAAATGAAACCTTAACGCTATGGAACTCGCCGCCCGACTGGGCTGGCGATGAG
CGAAATGTAGTGCTTACGTTGTCCCGCATTTGGTACAGCGCAGTAACCGGCAAAATCGCG
CCGAAGGATGTCGCTGCCGACTGGGCAATGGAGCGCCTGCCGGCCCAGTATCAGCCCGTC
ATACTTGAAGCTAGACAGGCTTATCTTGGACAAGAAGAAGATCGCTTGGCCTCGCGCGCA
GATCAGTTGGAAGAATTTGTCCACTACGTGAAAGGCGAGATCACCAAGGTAGTCGGCAAA
TAATGTCTAACAATTCGTTCAAGCCGAAGCCGCTTCGCGGCGCGGCTTAATTCGGGCGTT
AGATGCACTAAGCACATAATTGCTCACAGCCAAACTATCAGGTCAAGTCTGCTTTTATTA
TTTTTAAGCGTGCATAATAAGCCCTACACAAATTGGGAGATATATCATGAAAGGCTGGCT
TTTTCTTGTTATCGCAATAGTTGGCGAAGTAATCGCAACATCCGCATTAAAATCTAGCGA
GGGCTTTACTAAGCTTGCCCCTTCCGCCGTTGTCATAATCGGTTATGGCATCGCATTTTA
TTTTCTTTCTCTGGTTCTGAAATCCATCCCTGTCGGTGTTGCTTATGCAGTCTGGTCGGG
ACTCGGCGTCGTCATAATTACAGCCATTGCCTGGTTGCTTCATGGGCAAAAGCTTGATGC
GTGGGGCTTTGTAGGTATGGGGCTCATAATTGCTGCCTTTTTGCTCGCCCGATCCCCATC
GTGGAAGTCGCTGCGGAGGCCGACGCCATGGTGACGGTGTTCGGCATTCTGAATCTCACC
GAGGACTCCTTCTTCGATGAGAGCCGGCGGCTAGACCCCGCCGGCGCTGTCACCGCGGCG
ATCGAAATGCTGCGAGTCGGATCAGACGTCGTGGATGTCGGACCGGCCGCCAGCCATCCG
GACGCGAGGCCTGTATCGCCGGCCGATGAGATCAGACGTATTGCGCCGCTCTTAGACGCC
CTGTCCGATCAGATGCACCGTGTTTCAATCGACAGCTTCCAACCGGAAACCCAGCGCTAT
GCGCTCAAGCGCGGCGTGGGCTACCTGAACGATATCCAAGGATTTCCTGACCCTGCGCTC
TATCCCGATATTGCTGAGGCGGACTGCAGGCTGGTGGTTATGCACTCAGCGCAGCGGGAT
GGCATCGCCACCCGCACCGGTCACCTTCGACCCGAAGACGCGCTCGACGAGATTGTGCGG
TTCTTCGAGGCGCGGGTTTCCGCCTTGCGACGGAGCGGGGTCGCTGCCGACCGGCTCATC
CTCGATCCGGGGATGGGATTTTTCTTGAGCCCCGCACCGGAAACATCGCTGCACGTGCTG
TCGAACCTTCAAAAGCTGAAGTCGGCGTTGGGGCTTCCGCTATTGGTCTCGGTGTCGCGG
AAATCCTTCTTGGGCGCCACCGTTGGCCTTCCTGTAAAGGATCTGGGTCCAGCGAGCCTT
GCGGCGGAACTTCACGCGATCGGCAATGGCGCTGACTACGTCCGCACCCACGCGCCTGGA
GATCTGCGAAGCGCAATCACCTTCTCGGAAACCCTCGCGAAATTTCGCAGTCGCGACGCC
AGAGACCGAGGGTTAGATCATGCCTAGCATTCACCTTCCGGCCGCCCGCTAGCGGACCCT
GGTCAGGTTCCGCGAAGGTGGGCGCAGACATGCTGGGCTCGTCAGGATCAAACTGCACTA
TGAGGCGGCGGTTCATACCGCGCCAGGGGAGCGAATGGACAGCGAGGAGCCTCCGAACGT
TCGGGTCGCCTGCTCGGGTGATATCGACGAGGTTGTGCGGCTGATGCACGACGCTGCGGC
GTGGATGTCCGCCAAGGGAACGCCCGCCTGGGAGGCACTGTTGCAAAGTTAGCGATGAGG

CAGCCTTTTGTCTTATTCAAAGGCCTTACATTTCAAAAACTCTGCTTACCAGGCGCATTT
CGCCCAGGGGATCACCATAATAAAATGCTGAGGCCTGGCCTTTGCGTAGTGCACGCATCA
CCTCAATACCTTTGATGGTGGCGTAAGCCGTCTTCATGGATTTAAATCCCAGCGTGGCGC
CGATTATCCGTTTCAGTTTGCCATGATCGCATTCAATCACGTTGTTCCGGTACTTAATCT
GTCGGTGTTCAACGTCAGACGGGCACCGGCCTTCGCGTTTGAGCAGAGCAAGCGCGCGAC
CATAGGCGGGCGCTTTATCCGTGTTGATGAATCGCGGGATCTGCCACTTCTTCACGTTGT
TGAGGATTTTACCCAGAAACCGGTATGCAGCTTTGCTGTTACGACGGGAGGAGAGATAAA
AATCGACAGTGCGGCCCCGGCTGTCGACGGCCCGGTACAGATACGCCCAGCGGCCATTGA
CCTTCACGTAGGTTTCATCCATGTGCCACGGGCAAAGATCGGAAGGGTTACGCCAGTACC
AGCGCAGCCGTTTTTCCATTTCAGGCGCATAACGCTGAACCCAGCGGTAAATCGTGGAGT
GATCGACATTCACTCCGCGTTCAGCCAGCATCTCCTGCAGCTCACGGTAACTGATGCCGT
ATTTGCAGTACCAGCGTACGGCCCACAGAATGATGTCACGCTGAAAATGCCGGCCTTTGA
ATGGGTTCATGTGCAGCTCCATCAGCAAAAGGGGATGATAAGTTTATCACCACCGACTAT
TTGCAACAGTGCCGTGGAAGCTTATATCGAACGCGTCAGCCGGGCCAACCGCTTTGAATA
TGGCAAAGTGCGGGTCGCAGGCTGCGTCGGCTGGGCGCTATGCGCCTCGATAACCGGCGT
GCTGTTCGGCATCGATCCCAATATCACCTTCTGGATCGCCTCCGGCTTTGCGCTGGTGCT
CGGTCTGCTGCTCTGGCTGTCGCGGCCGGAAAGCAGCAACAGCGCCCAGGTTATCGAGGC
GCTGGGCGCCAATCGCCAGGCCTTTTCGCTGCGTACGGCGGCAGAGCTGCTGCGTATGCC
GCGCTTCTGGGGCTTTATCGTCTATGTGGTGGGTGTCGCCAGCGTCTACGACGTGTTTGA
CCAGCAGTTCGCCAACTTTTTCAAAAGCTTTTTCGCCAGTCCGCAGCGCGGTACCGAAGT
GTTTGGCTTCGTCACCACCGGCGGCGAGTTGCTCAACGCGCTGATCATGTTCTGCGCGCC
GGCCATCGTTAACCGCATCGGCGCCAAAAACGCCCTGCTGACCGCGGGGATGATCATGTC
GGTGCGTATTCTCGGGTCGTCCTTCGCCTCATCGGCAGTCGAGGTGGTGATCCTCAAGAT
GCTGCATATGTTTGAGATCCCCTTCCTGCTGGTGGGAACTTTTAAATATATCTCTTCCGC
CTTTAACCCGCGCCTCTCGGCCACCCTGTTCCTGATCGGTTTTAATCTGTCAAAACAGCT
GTCCGGGGTGGTGCTTTCCGCCTGGGTGGGCCGGATGTACGACACCGTCGGCTTCCATCA
GGCCTATCTGATCCTCGGCTGCATAACCCTGAGCTTCACCCTGCTCTCGTTCGTCACCCT
ACGCGGCGGCAACCGTCTGCTGCCGACCGCAGAGACGCAGAGCCCCGCCTGACTCCAGCG
CCCCCGTCAGGGATGACGGGCTTCACTCGACAATCCGCGTCTCACCGCCGCGATTCTCCA
GCGCATAGCGCTGACAAGGCGCCCCGGCGGCGATAAGCTCCGCCAGCTCCGCCGAGTGGC
TGGTCAGCCAGATCTGGCTGTAGCGCGAGGCCTCGATAATCAGCCGGGCCAGCGCGGGCA
ACATGTCGCGATGCAGGCTGTTCTCCGGTTCATTGATCGCCAGAAACGCCGGCGGGCGCG
GACTGAGCAGCGCGACGGCGAGGCACAAAAAGCGCAGCGTACCGTCGGACATCTCCGCCG
CCAGCAGCGGGCGGCGGATCCCTTCGCGACGCATTTTCAGAGCGAAACGCGAGTGCTCGT
TTTCACAATAAAACTGGCAGCCGGGAAAGGCGTCGGCAAGGATTTCATGCAAAATCTCCT
CCGCGCCGATCTCGACAATGGTCTGAAAGGCGGCGGCGAGATTCTGGCCATCGCTGTCGA
GGACTGGCGAACGGTAACCCACGGCCGGCTGACGCAGTGGCGAATGACGGCCAATCGCGA
ATTCATGATAAAACCGCCAACGGCGCAGCGTCTCCCGCACTCGCGATACTTCGGGAAAAC
GGTGCGGCTCGCCAAGCTGACCGAACACCGACTCATTTTCATAAATACTCTCGGTGAAAG
TACTTTTCTCCCCGGTGACATCGACGAGAAACGCCGCCTGATTCTTGCGCTGCAGTACGC
GGGAGGAAGGCCGGCGGGAGTAGCCGGCCAGCCAGATATTCTCTTCTTTAACGATAGGAT
CGAGCATGAATTGCGTCGGATAGGGCAATTTTTCAGGGAAGCCTATCTGCAGTTCATAAT
CGAAGCTGTCGGTCCGGCAGGCGATTTGCAGCCGGCGGGGGTGACGATCGAGGGGCGAAC
GCTCCCCGGACCACATCATATTCTCCAGACCGCCCTCTTCGCTGATAAACCCCGAAAGCC
TGCCCTCAGCGGCAGCGGTCAGCAGGTGGATGGCGTTATAAATATTGGATTTACCGCAGC
CGTTAGGGCCGAAGACAATGTTCAGCGGCCCGAGCTCCAGCGCGATATCCTTTACTGAGC
GAAAATTTTGAATACGAATGTACTGAATCATTATGCGTCCGGCCCTGGAAATAGAAGGCT
GCCACAGTAGCACAGCGGCGCCCGGCATACCCTCTTACGCCCCCTTTCGGGCAGGGTTTA
ACAGAACATTTTTTTCATTCCACGGGTCAGGGCGACCCGCGTCAGCTTATCGCTATCCTG
ATGGGCGTCGCTGAAAACGCCAAAAAAATAGCGTTCATCGTCAATGGTTTGGCTAACAAT
TCGGTAGCTCAACGATTGCCCGGATAGCGCACCGTACTGCGCAGGCGTCACCGAGACTTC
GGTCGCGTCATCGGCGACGCTGACCAACAGCCCGTCCGCCTTACCGCCCACCAGCATGAC
CTTAATTATCGGGTGCACCAGTTCCTCCCGGGATGGCATATAGCGTTGGCGAACCGCAGG
GACCGGCGGGCGTGGTGATCGGCAAACAGCGACGCCCGACAGAGTGCGGTATTTAGTACT

TTCTTATAGTTCATCACGGCCTTGAGTCAAAAAATAGCGTGCTTAGGCAGGGCTAGATAT
TGATTATTCGAAATAAAAGATGAAAAATGATGAAGGAAAAAAGAGGAATTGTGAATCAGC
AAAACGCCGGGTTATTCTTATTTGTCGCTTCTTTACTCGCCTTTATCGGCCCTCACTCAA
GGATGTATTGTGGTTATGCGTTATATTCGCCTGTGTATTATCTCCCTGTTAGCCACCCTG
CCGCTGGCGGTACACGCCAGCCCGCAGCCGCTTGAGCAAATTAAACTAAGCGAAAGCCAG
CTGTCGGGCCGCGTAGGCATGATAGAAATGGATCTGGCCAGCGGCCGCACGCTGACCGCC
TGGCGCGCCGATGAACGCTTTCCCATGATGAGCACCTTTAAAGTAGTGCTCTGCGGCGCA
GTGCTGGCGCGGGTGGATGCCGGTGACGAACAGCTGGAGCGAAAGATCCACTATCGCCAG
CAGGATCTGGTGGACTACTCGCCGGTCAGCGAAAAACACCTTGCCGACGGCATGACGGTC
GGCGAACTCTGCGCCGCCGCCATTACCATGAGCGATAACAGCGCCGCCAATCTGCTACTG
GCCACCGTCGGCGGCCCCGCAGGATTGACTGCCTTTTTGCGCCAGATCGGCGACAACGTC
ACCCGCCTTGACCGCTGGGAAACGGAACTGAATGAGGCGCTTCCCGGCGACGCCCGCGAC
ACCACTACCCCGGCCAGCATGGCCGCGACCCTGCGCAAGCTGCTGACCAGCCAGCGTCTG
AGCGCCCGTTCGCAACGGCAGCTGCTGCAGTGGATGGTGGACGATCGGGTCGCCGGACCG
TTGATCCGCTCCGTGCTGCCGGCGGGCTGGTTTATCGCCGATAAGACCGGAGCTAGCAAG
CGGGGTGCGCGCGGGATTGTCGCCCTGCTTGGCCCGAATAACAAAGCAGAGCGCATTGTG
GTGATTTATCTGCGGGATACCCCGGCGAGCATGGCCGAGCGAAATCAGCAAATCGCCGGG
ATCGGCGCGGCGCTGATCGAGCACTGGCAACGCTAACCCGGCGGTGGCCGCGCGCGTTAT
CCGGCCCGCAGCACCTCGCAGGCGTGCCGGGCGATATGACTGGCGGCGGCATCGGAAAGA
TGCCGGTCGGTAATGATGGTGGTGAACCGGGTCAAAGGTAACGCCATAAACGTGGCCACC
TGATTGTATTTCGAACTGTCGCACAGCAGGATGCTTCTCGCGCTGACCTGGCTGACGGTC
TCCTTGACGGTAACCTTGTTCTCATCAGGGGTGAATATCCCGCGACTGTCCCAGCCGCTG
GCGGAGATAAAGGCCGTATCGATAGCCAGGTGGCGTAACGTACGCGCCGCCGATTCGCCC
ACGCAGGAGCGGTTCTCCCGGCACAGAGTGCCGCCGGTGTGGATCACGCCGCACTGGCTG
GCATCGATCAGCAGCTGGGTTATCTCAAAATCATTGGTGACCACCTGGAGATCGTTCCGG
TCGAGGATCGCCCGCGCCAGCGCCAGGGTGGTAGTCCCGGCATCCAGATAGATGCAACTG
TTTTTAGCGATATGACTCGCCGCCAGCGCGCCGATCGCCTGTTTCTCCTCACTCTGCAGC
GTGCTTTTCACCAGATGACTGGGTTCCGCAGCCAGCCGGCTGACGGCGCGCACGCCGCCC
GAGACGCTGACCAGCAGCCCCTGCTCCTCCAGTTTACTGACGTCCCGACGGATGGTCATA
TGGGACACGCCGAGGATCTCCGTCAGCTCGTTAATGCTTACCGCCCCACGCTGCTCCACC
AGGGCTAAAATACGCTGATGTCGTTCTATTGGAATCACCGCTCTCCCCTTACCATTTTTT
CACACCAGGCGTCACCACCCAGGCTACGGCCCTGGCGACACCCGGTGTTGTTATCGCGCT
TTGCCGCTGTTTTTAGGCTATTTTAGGGCAAGAATCGCCGTTGTGCAGCCTCTTTCCGCC
TGTGAATTTTTTATATTCATGTGGGTTATTCGTGATAACTCTCACATAAATTCACATGAT
AACGCTTTATTCTATCATTAAATCACATTAATTAACTAATGTTCACAAGGAGACCAGCAT
GGCTGCACACACTAACGTCTGCGTGATTGGACTGGGTTCAATGGGCATGGGCGCCGCCCG
CGCCTGCCTGCAGGCGGGCCTGAACACCTGGGGCGTTGACATCAATCCCGACAACTGTCG
CGCACTGCTGGCGGCGGGCGCCAATGGCGCGGGCCCCAGCGCGGTGCCGTTCGCCGCGGA
ACTGGATGCAGTTGTGCTGCTGGTGGTCAATGCCGCCCAGGTGCGGGGGATCCTGTTCGG
CGAGAGCGGCCTCGCCGCCCATCTGAAGCCGGGCACCGTCGTGATGGTGTCGTCCACCAT
CGCTTCCGCCGATGCTCAGGCCATTGCCGAGGCGCTGGCGGAGTACCAGCTATTGATGCT
CGACGCGCCGGTATCGGGCGGCGCCGTGAAAGCGGCCGCCGGCGACATGACGGTGATGGC
CTCCGGGAGCGATGCCGCCTTTGCCCGCCTCGCGCCGGTGCTGGACGCCGTGGCCGGCAA
AGTCTACCGCATAGGGAGCGACATTGGTCTTGGCTCGACGGTAAAAATTATCCATCAGCT
GCTGGCCGGGGTGCACATCGCCGTTGCCGCCGAAGCGATGGCGCTTGCCGCCCGCGCCGG
GATCCCACTCGAAACGATGTATGACGTGGTCACCCACGCGGCGGGTAATTCATGGATGTT
TGAGAATCGCATGCAGCACGTCCTGGATGGCGATTACTCGCCAAAATCCGCTGTCGATAT
TTTTGTCAAAGATCTCGGGCTGGTGAATGACACTGCCCGGGCGCTGACCTTCCCGCTGCC
GCTCGCTACCACCGCGCTGAATATGTTCACCTCCGCCAGTAATGCCGGATTCGGTCGGGA
AGATGACAGCGCGGTGATCAAGATTTTCAACGGCATCACCCTGCCGGGCCATAAACAGTG
AGGAGAGACAACATGCAGCTTGGTGTCATTGCCGATGACTTCACCGGCGCCACGGATATT
GCCAGCTTCCTCGTGCGCAACGGCATGCCGACGGTGCAACTGAATGGCGTGCCGACCCGC
GATCTTCCGCTGACCAGCGAGGCGGTGGTCATCAGCCTGAAAACTCGCTCCTGCCCGGCG
GAAATGGCCGTCAGCCAGTCGCTGGCGGCCCTGCGCTGGCTGCAGGCCCAGGGCTGTCAG

CAGTTTTATTTCAAGTACTGTTCCACTTTCGACAGCACCGCGCAGGGCAACATTGGCCCG
GTGCTGGATGCCCTGCTGGCCGAGCTGGGTGAGACGCGGACGGTGATTTCCCCGGCGCTG
CCGGTTAACGGCCGCACGGTCTATCAGGGATATCTGTTCGTCGGCGAGCAACTGCTGAAT
GAGTCCGGGATGCGCCACCATCCGGTGACGCCGATGGAGGATGCGCACCTGGGCCGCTTA
ATTGAGCGCCAGGGGCGCGGAAAAGCCGCGCTGATTGCCTGGCCGATTGTCGACCGGGGG
CCGGAGGCGGTCGCCGCCGCGCTGGCGGCAGTCAACGATCCGGCGGTGCGCTATGTGGTG
CTCGACGCCCTCAGCGAACAGGATCTGCTCACCCAGGGCGTGGCGCTGCGGGAGATGAAG
CTGGTCTCCGGCGGTTCCGGCCTCGCCATCGGCCTCGCCCGCGACTTGGCGCAGCGCCAT
GGCGCCCGGGGTGAAAGCGCTCAGGCCGGCATGCCGCTGGTCGGCCCGGCGGTGGTGCTC
TCGGGCTCCTGCTCGGTGATGACCAACAGCCAGGTGGCGGCCTATCGTCAACAGGCCCCC
GCCCGCGCCGTCGACTTAAGCGCCTGCTTTACCGATCTGGAGAGCTACGTCAGGACGCTG
ACTGACTGGGTGGACGCGCAGCGCGATGCGCCGCTGGCGCCGATGATCTATGCCACCACC
GAGCCGCAAACGCTGCAGCGGATCCAGGCGCAGTATGGCGACAAGGCCAGCAGCGAACGG
GTGGAACAGCTGTTTGCCGCTCTTGCCGCCGCCCTGAAGGCGAAAGGATTTACCCGCTTT
ATTGTGGCCGGAGGGGAAACGTCGAGCATTGTGGCGCAGACCCTGGGGGTTGAGGCGTTC
CATATTGGGCCGACCATCTCCCCTGGCGTGCCCTGGGTGCGTGACACCCGCCAGCCGCTC
TCCCTGGCGCTGAAGTCAGGTAACTTCGGCGATATCCAGTTCTTTGCCCGTGCCCAGCAG
GAGTTTCGTCATGACTGAGCAACAACTGCGAGAGGAAATGGTGCAGATTGGCGCCTCGTT
ATTTAGCCGCGGCTATGCCACCGGCTCCGCTGGCAATCTGTCGCTGCTGCTGCCGGACGG
CAACCTGCTGGCGACGCCGACCGGCGCCTGCCTCGGCGAACTGCAGGCTCAGCGGTTGTC
GGTGGTGACGCTGCAAGGAGAATGGATCTCCGGCGACAAACCGTCGAAAGAGGTCACTTT
TCACCGGGCAGTCTATTTGCACAACCCGGCCTGCAAGGCGATCGTCCACTTGCACAGCCA
CTATCTGACCGCGCTCTCCTGCCTGCAGGGGCTCGATCCGCACAACTGTATCCGCCCCTT
TACCCCCTATGTGGTGATGCGCGTCGGCGACGTCCCGGTGGTTCCCTACTACCGGCCGGG
CGATGACCGTATTGCCCAGGCGCTGGCCGGGCTGGCGCCCCGCTATAACGCCTTTTTACT
GGCCAACCACGGACCGGTGGTTACTGGCTCATCGCTGCGCGAAGCCACCAACAATACCGA
GGAACTGGAAGAGACCGCACGGCTGATATTTACCCTCGGCAACCGCGAGATCCGCTACCT
GACCGCTGACGAAGTAAAAGAACTGAGATAAAACCATGCCAAAATTCGCTGCAAATTTAT
CCATGCTGTTTACCGAACTGCCGTTCCTGGAACGCTTTGCCGCCGCTGCCCGGGCGGGAT
TTGAAGCCGTTGAGTTTCTGTTTCCCTATGAGTATGCCGCCGGGGAAATCAGACAGCGCC
TGCAGGAGAACCAGCTGCAGCTGGTGCTGTTCAATACCCCACCCGGCGACGTCAACGCCG
GAGAATGGGGGCTCGCCGCCATCCCCGGGAGAAGCGCGGAGGCCCGACGCGATATTGAGC
TGGCGCTGGAGTATGCCTGCCAGCTCGGTTGCCCGCAGGTGCATATCATGGCCGGCGTGG
TGCCGCCGGGAGCGGATCGCGCAGCCTGCGAGGCCGTGCTTATCGATAATCTGCGCTACG
CGGCGGAGTGCTTTGCCCGCCACGACAAGCGGATACTCATCGAAGCGCTGAATCCGCAGA
CCAAACCGGGCTATCTCTACCACAGTCAATATCAGACGCTGGCGATGGTGAAACGGGTCG
ACAGGCCGAACCTGGCGGTGCAGTTAGATCTGTTTCACGCGCAGAAGGTGGACGGCAATT
TAAGCCATCTTATTACCGAATATGCCGGCCAGTATCGCCATATTCAGATTGCCTCCCTGC
CCGACCGTCATGAACCGGATGAGGGCGAAAGGCACTGTTGCAAAGTTAGCGATGAGGCAG
CCTTTTGTCTTATTCAAAGGCCTTACATTTCAAAAACTCTGCTTACCAGGCGCATTTCGC
CCAGGGGATCACCATAATAAAATGCTGAGGCCTGGCCTTTGCGTAGTGCACGCATCACCT
CAATACCTTTGATGGTGGCGTAAGCCGTCTTCATGGATTTAAATCCCAGCGTGGCGCCGA
TTATCCGTTTCAGTTTGCCATGATCGCATTCAATCACGTTGTTCCGGTACTTAATCTGTC
GGTGTTCAACGTCAGACGGGCACCGGCCTTCGCGTTTGAGCAGAGCAAGCGCGCGACCAT
AGGCGGGCGCTTTATCCGTGTTGATGAATCGCGGGATCTGCCACTTCTTCACGTTGTTGA
GGATTTTACCCAGAAACCGGTATGCAGCTTTGCTGTTACGACGGGAGGAGAGATAAAAAT
CGACAGTGCGGCCCCGGCTGTCGACGGCCCGGTACAGATACGCCCAGCGGCCATTGACCT
TCACGTAGGTTTCATCCATGTGCCACGGGCAAAGATCGGAAGGGTTACGCCAGTACCAGC
GCAGCCGTTTTTCCATTTCAGGCGCATAACGCTGAACCCAGCGGTAAATCGTGGAGTGAT
CGACATTCACTCCGCGTTCAGCCAGCATCTCCTGCAGCTCACGGTAACTGATGCCGTATT
TGCAGTACCAGCGTACGGCCCACAGAATGATGTCACGCTGAAAATGCCGGCCTTTGAATG
GGTTCATGTGCAGGGTGATGCTGCCAACTTACTGATTTAGTGTATGATGGTGTTTTTGAG
GTGCTCCAGTGGCTTCTGTTTCTATCAGCTGTCCCTCCTGTTCAGCTACTGACGGGGTGG
TGCGTAACGGCAAAAGCACCGCCGGACATCAGCGCTATCTCTGCTCTCACTGCCGTAAAA

CATGGCAACTGCAGTTCACTTACACCGCTTCTCAACCCGGTACGCACCAGAAAATCATTG
ATATGGCCATGAATGGCGTTGGATGCCGGGCAACCGCCCGCATTATGGGCGTTGGCCTCA
ACACGATTTTCCGCCATTTAAAAAACTCAGGCCGCAGTCGGTAACCTCGCGCATACAGCC
GGGCAGTGACGTCATCGTCTGCGCGGAAATGGACGAACAGTGGGGATACGTCGGGGCTAA
ATCGCGCCAGCGCTGGCTGTTTTACGCGTATGACAGGCTCCGGAAGACGGTTGTTGCGCA
CGTATTCGGTGAACGCACTATGGCGACGCTGGGGCGTCTTATGAGCCTGCTGTCACCCTT
TGACGTGGTGATATGGATGACGGATGGCTGGCCGCTGTATGAATCCCGCCTGAAGGGAAA
GCTGCACGTAATCAGCAAGCGATATACGCAGCGAATTGAGCGGCATAACCTGAATCTGAG
GCAGCACCTGGCACGGCTGGGACGGAAGTCGCTGTCGTTCTCAAAATCGGTGGAGCTGCA
TGACAAAGTCATCGGGCATTATCTGAACATAAAACACTATCAATAAGTTGGAGTCATTAC
CCGCTTGAGTTAAGCCGCGCCGCGAAGCGGCGTCGGCTTGAACGAATTGTTAGACATTAT
TTGCCGACTACCTTGGTGATCTCGCCTTTCACGTAGTGAACAAATTCTTCCAACTGATCT
GCGCGCGAGGCCAAGCGATCTTCTTCTTGTCCAAGATAAGCCTGTCTAGCTTCAAGTATG
ACGGGCTGATACTGGGCCGGCAGGCGCTCCATTGCCCAGTCGGCAGCGACATCCTTCGGC
GCGATTCTGCCGGTTACTGCGCTGTACCAAATGCGGGACAACGTAAGCACTACATTTCGC
TCATCGCCAGCCCAGTCGGGCGGCGAGTTCCATAGCGTTAAGGTTTCATTTAGCGCCTCA
AATAGATCCTGTTCAGGAACCGGATCAAAGAGTTCCTCCGCCGCTGGACCTACCAAGGCA
ACGCTATGTTCTCTTGCTTTTGTCAGCAAGATAGCCAGATCAATGTCGATCGTGGCTGGC
TCGAAGATACCTGCAAGAATGTCATTGCGCTGCCATTCTCCAAATTGCAGTTCGCGCTTA
GCTGGATAACGCCACGGAATGATGTCGTCGTGCACAACAATGGTGACTTCTACAGCGCGG
AGAATCTCGCTCTCTCCAGGGGAAGCCGAAGTTTCCAAAAGGTCGTTGATCAAAGCTCGC
CGCGTTGTTTCATCAAGCCTTACGGTCACCGTAACCAGCAAATCAATATCACTGTGTGGC
TTCAGGCCGCCATCCACTGCGGAGCCGTACAAATGTACGGCCAGCAACGTCGGTTCGAGA
TGGCGCTCGATGACGCCAACTACCTCTGATAGTTGAGTCGATACTTCGGCGATCACCGCT
TCCCTCATGATGTTTAACGCCTGGCACAGCGGATCGCAAACCTGGCGCGGCTTTTGGTAC
AAAAGGCGTGACAGGTTTGCGAATCCGTTGCTGCCACTTGTTAACCCTTTTGCCAGATTT
GGTAACTATAATTTATGTTAGAGGCGAAGTCTTGGGTAAAAACTGGCCTAAAATTGCTGG
GGATTTCAGGAAAGTAAACATCACCTTCCGGCTCGATGTCTATTGTAGATATATGTAGTG
TATCTACTTGATCGATCAGGCTTTTGTATATCTCCCCACCACCTGAAACAATGACATGAT
CCGTTATTTTCTTTAGGTTGGTTAAAGCATCTTTAATTGATGGAAAGATCACTACGTTCT
CATTGTCAGATGTAAAACTTGAACGTGTTACGACCGCATACTTTCGGTTGGGTAATGCTC
CCATTGATTCAAAAGTCTTGCGTCCAACCAACAGCCATTGGTTATAGGTAATAGCTTTAA
ACAGGAGCTGTTCACCTTTGGCACTCCATGGAATATCAGGGCCATTCCCGATAACTCCAT
TCTTCGATATAGCTACCATTAGTGATAGTTTCACAATTCTTCCTCAGAGGTTAACTTTGT
TTTAGGGCGACTGCCCTGCTGCGTAACATCGTTGCTGCTCCATAACATCAAACATCGACC
CACGGCGTAACGCGCTTGCTGCTTGGATGCCCGAGGCATAGACTGTACCCCAAAAAAACA
GTCATAACAAGCCATGAAAACCGCCACTGCGCCGTTACCACCGCTGCGTTCGGTCAAGGT
TCTGGACCAGTTGCGTGAGCGCATACGCTACTTGCATTACAGCTTACGAACCGAACAGGC
TTATGTCCACTGGGTTCGTGCCTTCATCCGTTTCCACGGTGTGCGTCACCCGGCAACCTT
GGGCAGCAGCGAAGTCGAGGCATTTCTGTCCTGGCTGGCGAACGAGCGCAAGGTTTCGGT
CTCCACGCATCGTCAGGCATTGGCGGCCTTGCTGTTCTTCTACGGCAAGGTGCTGTGCAC
GGATCTGCCCTGGCTTCAGGAGATCGGAAGACCTCGGCCGTCGCGGCGCTTGCCGGTGGT
GCTGACCCCGGATGAAGTGGTTCGCATCCTCGGTTTTCTGGAAGGCGAGCATCGTTTGTT
CGCCCAGCTTCTGTATGGAACGGGCATGCGGATCAGTGAGGGTTTGCAACTGCGGGTCAA
GGATCTGGATTTCGATCACGGCACGATCATCGTGCGGGAGGGCAAGGGCTCCAAGGATCG
GGCCTTGATGTTACCCGAGAGCTTGGCACCCAGCCTGCGCGAGCAGCTGTCGCGTGCACG
GGCATGGTGGCTGAAGGACCAGGCCGAGGGCCGCAGCGGCGTTGCGCTTCCCGACGCCCT
TGAGCGGAAGTATCCGCGCGCCGGGCATTCCTGGCCGTGGTTCTGGGTTTTTGCGCAGCA
CACGCATTCGACCGATCCACGGAGCGGTGTCGTGCGTCGCCATCACATGTATGACCAGAC
CTTTCAGCGCGCCTTCAAACGTGCCGTAGAACAAGCAGGCATCACGAAGCCCGCCACACC
GCACACCCTCCGCCACTCGTTCGCGACGGCCTTGCTCCGCAGCGGTTACGACATTCGAAC
CGTGCAGGATCTGCTCGGCCATTCCGACGTCTCTACGACGATGATTTACACGCATGTGCT
GAAAGTTGGCGGTGCCGGAGTGCGCTCACCGCTTGATGCGCTGCCGCCCCTCACTAGTGA
GAGGTAGGGCAGCGCAAGTCAATCCTGGCGGATTCACTACCCCTGCGCGAAGGCCATCGG

TGCCGCATCGAACGGCCGGTTGCGGAAAGTCCTCCCTGCGTCCGCTGATGGCCGGCAGCA
GCCCGTCGTTGCCTGATGGATCCAACCCCTCCGCTGCTATAGTGCAGTCGGCTTCTGACG
TTCAGTGCAGCCGTCTTCTGAAAACGACACCATGTGCAAACGATGTCAGAATAGAGTTAA
ATTTCCTATTGATTGACATATTCCGTCAAAGGTAATAGATTTCATCCTGACACTTTTGCC
TTTGGAGGCATCTTGCAAGGTCAACGCATCGGCTATGTCCGCGTCAGCAGCTTCGACCAG
AACCCGGAACGGCAATTGGAGGGTGTTCAGGTGGCGCGGGTGTTCACCGACAAGGCTTCT
GGCAAGGACACCCAGCGTCCCGAGCTGGAAAGGCTGCTGGCCTTCGTCCGCGAGGGCGAC
ACCGTGGTGGTGCATAGCATGGACAGGCTGGCACGCAACCTTGATGACCTGCGCCGCATC
GTCCAAGGGCTGACACAACGGGGCGTGCGCATGGAGTTCGTCAAAGAAGGGCTGAAGTTC
ACCGGCGAGGACTCACCGATGGCCAATCTGATGCTGTCGGTCATGGGAGCCTTCGCTGAG
TTCGAGCGCGCCCTGATCCGCGAACGTCAGCGCGAGGGAATCGTGCTGGCCAAGCAGCGC
GGTGCCTACCGGGGACGAAAGAAATCGCTGAACAGCGAACAAATTGCCGAGTTGAAACGG
CGAGTTGCGGCAGGCGACCAAAAAACCTTGGTGGCCCGTGACTTCGGCATCAGCCGCGAA
ACCTTGTACCAGTACCTGCGGGAAGACTGACCATGCCACGCCGCTCAATCCTGTCCGCCA
CCGAGCGCGAAAGCCTGCTGGCACTGCCAGATGCCAAAGACGAACTGATACGGCACTACA
CGTTCAACGAAACCGACCTGTCGGTGATCCGTCAGCGTCGCGGCGCCGCGAATCGATTGG
GCTTCGCTGTGCAGCTTTGCTACTTGCGATTCCCTGGCACCTTTTTGGGCGTCGATGAGC
CTCCGTTTCCGCCCCTGTTGCGCATGGTGGCCGCGCAACTCAAGATGCCAGTGGAAAGTT
GGAGCGAGTACGGCCAGCGCGAACAGACACGGCGGGAGCACTTGGTCGAGCTGCAAACGG
TTTTTGGGTTCAAGCCCTTCACCATGAGCCACTATCGGCAAGCCGTGCATACATTGACCG
AGCTGGCCTTGCAGACCGACAAAGGCATCGTGCTGGCGAGCGCACTTGTCGAGAATCTGC
GGCGGCAGAGCATTATCCTGCCCGCCATGAATGCCATCGAGCGCGCAAGCGCCGAGGCCA
TCACCCGTGCCAACCGACGCATTTACGCGGCGCTGACCGATTCTTTGTTATCACCCCACC
GTCAGCGCCTGGACGAACTTCTCAAGCGCAAGGACGGCAGTAAAGTGACGTGGCTGGCAT
GGCTGCGCCAGTCGCCTGCCAAACCGAACTCTCGCCACATGCTCGAACATATTGAGCGCC
TGAAATCCTGGCAAGCACTTGATCTGCCCGCAGGCATCGAGCGGCAGGTTCACCAGAACC
GCCTGCTCAAAATCGCTCGTGAAGGTGGCCAGATGACGCCTGCTGATCTGGCAAAGTTCG
AGGTGCAACGACGCTATGCCACGCTGGTAGCGCTGGCCATCGAAGGCATGGCCACCGTCA
CCGATGAAATCATCGACCTTCACGATCGCATCATCGGCAAGCTGTTCAACGCGGCCAAGA
ACAAGCATCAGCAGCAGTTCCAGGCTTCCGGCAAGGCGATCAACGACAAGGTGCGGATGT
ATGGGCGCATCGGTCAAGCGTTGATTGAGGCCAAGCAAAGCGGCAGCGATCCGTTCGCCG
CCATCGAGGCCGTTATGCCCTGGGACACCTTCGCCGCCAGCGTCACCGAAGCGCAAACAT
TGGCGCGGCCTGCCGACTTTGATTTCCTGCACCACATCGGTGAAAGCTATGCCACGCTAC
GCCGCTACGCGCCGCAGTTCCTGGGCGTGCTCAAATTGCGGGCTGCGCCCGCCGCCAAGG
GTGTGCTCGATGCCATCGACATGCTGCGCGGCATGAACAGCGACAGCGCGCGCAAGGTGC
CCGCCGATGCGCCAACCGCATTCATCAAGCCGCGCTGGGCAAAGCTGGTTCTGACCGACG
ACGGCATCGACCGGCGTTACTACGAGTTATGCGCCCTGTCGGAGCTGAAGAACGCGCTGC
GCTCCGGTGATGTCTGGGTGCAGGGTTCTCGCCAGTTCAAGGACTTCGACGAATACCTGG
TGCCGGTCGAGAAGTTCGCCACTTTGAAGCTGGCCAGCGAATTGCCGCTGGCAGTGGCCA
CCGACTGCGACCAATACCTGCATGACCGGTTGGAATTGTTGGAGGCGCAACTCGCCACAG
TCAACCGCATGGCTGCGGCCAACGACTTACCGGATGCCATCATCACCACCGCGTCAGGCC
TGAAGATCACGCCGCTGGACGCGGCAGTACCAGACGCCGCGCAAGCCATGATCGACCAGA
CAGCTATGCTGCTGCCGCACCTCAAAATCACCGAGTTGCTGATGGAGGTCGATGAATGGA
CGGGCTTCACCCGCCACTTCACACACCTGAAGACCAGCGACACGGCCAAGGACAAAACCT
TGCTGTTGACGACGATCCTGGCCGACGCGATCAACCTGGGTCTGACCAAAATGGCCGAGT
CCTGCCCTGGCACCACCTACGCCAAGCTGTCTTGGCTGCAAGCCTGGCACATCCGCGATG
AAACCTATTCGACGGCGCTGGCCGAGCTGGTGAATGCGCAGTTTCGGCAACCCTTCGCCG
GCAACTGGGGTGACGGCACCACGTCATCGTCGGACGGCCAGAACTTCAGAACCGGCAGCA
AAGCAGAAAGCACTGGTCATATCAACCCGAAGTATGGAAGCAGTCCAGGACGGACTTTCT
ACACCCATATCTCCGACCAGTACGCGCCCTTCAGTGCCAAGGTGGTCAACGTGGGCATTC
GTGATTCAACTTACGTGCTTGATGGCCTGCTGTACCACGAGTCGGACTTGCGCATCGAGG
AACACTACACCGACACGGCAGGCTTCACCGATCACGTGTTTGGCTTGATGCATTTGCTGG
GATTTCGCTTCGCGCCGCGTATCCGTGACTTGGGCGAAACCAAGCTATTCATCCCCAAGG
GCGATGCCGCCTATGACGCGCTCAAGCCGATGATTAGCAGCGACAGGCTGAACATCAAGC

AAATACGCGCCCATTGGGATGAAATTCTGCGGCTGGCCACCTCCATCAAGCAAGGCACGG
TAACGGCTTCGCTGATGCTGCGCAAACTCGGCAGCTACCCGCGCCAGAACGGCTTGGCCG
TGGCGTTGCGCGAGCTGGGGCGCATCGAGCGCACGCTGTTCATTTTGGATTGGCTGCAAA
GCGTGGAGCTGCGCCGCCGCGTCCATGCGGGGCTGAATAAGGGCGAGGCGCGCAACGCGC
TGGCCAGGGCGGTCTTCTTCTACCGATTGGGTGAAATCCGCGACCGCAGTTTTGAGCAGC
AGCGCTACCGGGCCAGCGGCCTCAATCTGGTGACGGCGGCCATCGTGTTGTGGAACACGG
TATATCTGGAGCGTGCCACCAGTGCTTTGCGTGGCAACGGCACGGCGCTGGACGACACAT
TGTTGCAATATCTGTCGCCGCTGGGGTGGGAGCACATCAACCTGACCGGCGATTACCTAT
GGCGCAGCAGCGCCAAGGTCGGTGCGGGGAAGTTTAGGCCATTGCGACCGCTGCCACCGG
CTTAGCGTGCTTTATTTAATGAGATGGTCACTCCCTCCTTCCCGGTACTATGCTGAGGAC
AGGCTTTCATTCGGAGAACCATCATGGAAAACATTGCGCTTATTGGTATCGATCTGGGTA
AGAACTCTTTCCATATTCATTGTCAGGATCATCGTGGGAAGGCCGTTTACCGTAAAAAAT
TCACCCGACCAAAGCTAATCGAATTTCTGGCGACATGCCCGGCAACAACCATCGCGATGG
AAGCCTGTGGCGGTTCTCACTTTATGGCACGCAAGCTGGCAGAGTTAGGGCATTTTCCAA
AGCTGATATCACCGCAATTTGTCCGCCCATTCGTTAAAAGCAACAAAAATGACTTCGTTG
ATGCTGAAGCTATCTGTGAAGCAGCATCACGTCCATCTATGCGTTTCGTGCAGCCCAGAA
CCGAATCTCAGCAGGCAATGCGAGCTCTGCATCGTGTCCGTGAATCCCTGGTTCAGGATA
AGGTGAAAACAACTAATCAGATGCATGCTTTTCTGCTGGAATTTGGTATCAGCGTTCCGC
GAGGTGCTGCCGTTATTAGTCGACTGAGTACCCTTCTTGAGGACAGTAGTTTGCCTCTTT
ATCTCAGCCAGTTACTGCTGAAATTACAACAGCATTATCACTATCTTGTTGAGCAGATTA
AAGATCTGGAATCTCAGTTGAAACGAAAGTTGGACGAAGATGAGGTTGGACAGCGCTTGC
TGAGTATTCCCTGCGTTGGAACGCTGACTGCCAGTACTATTTCAACTGAGATTGGCGACG
GGAAGCAGTACGCCAGCAGCCGTGACTTTGCGGCGGCAACAGGGCTGGTACCCCGACAGT
ACAGCACGGGAGGTCGGACGACATTGTTAGGGATTAGCAAGCGGGGCAACAAAAAGATCC
GAACTTTGTTGGTTCAGTGTGCCAGGGTATTCATACAAAAACTGGAACACCAGTCTGGCA
AGTTGGCCGACTGGGTCAGGGAGTTGTTGTGTCGGAAAAGCAACTTTGTCGTCACCTGTG
CTCTGGCAAACAAGCTGGCCAGAATAGCCTGGGCACTGACGGCGCGACAGCAAACTTACG
AAGCATAAAGGCAGAAATACACCAGTTTAAACAATCATTCATCTGGTTTTGCGAATACTG
ATATTGATGATACTAACGGCCCACCGGCCTGTTGAGGAACCTGTAAAACGGAAAGGCTCA
TTGAAGCCGTATATTTTCTGGAGGTTCATCAGGCGCGGAACTCATCGAGGCGCGGGAATA
AAATCCCATTCAGACGCCGGATAGATTCAAGCAAGCCAACTTGTCGTCAAAATCGGTGTT
GCAAAAACGGGAGTGACCATAGATTCCGTTTTCTGAGACGACCCCTTCTGATGCCATCGC
GCTGCTACGCCAATCAGCGTGGATGGACCGAAATTTGTCCTAATCGAGCAAATCTAGGCA
CTCTAAAAGTCGTCCTATAACCACAAAGTTAGACCATCATGCAAAGTGGGGTATAGAGGG
TGCCAGCCCCACCCCAGCAGTCCCCCTCAGCCACATTTCTGGCTTGCCCCGCGCTTTTCT
TCTTTCTCTCTTTTTCTCTCTTTTTCTCTCTCTTTCTCAATCTAATCTCACACACGAAGG
TCAAACAGGGCACCCGCCGACGCGAAGCGGAGGCGTATTCTTGCCCCTCCCCCTCGTTGC
ATGATGGTCTAAATCTGTCTACTATGTAACCAATGAACTGTTAGAACAAACCCACACATA
GGCGGCAGCATGGCTAAAAACTTCATATCAGAACAATTCTCGGCCATGTGCCGCGACTTA
ACGAACCTGTCCTCTCTTATCAAACGACTACCGCCACGGTACGCAAAAGTTGCGGCCATC
CCTCCTACCGGAAAAGGAATGGAGAACGAGCCCGTCAACAAGATTGTCGTAACTGAACAA
ACTGGGCGCGAAGCTCTTGAGCTGGCAGCGCACAGCTACAGAGATCTTCACATCAACCCA
GACTATTCGCAGAAGTCTGCCAGGCGCACCGTGGGGGTACTCTGGTTCTCCCCTTCCAGG
ATTGGTGTAGCTGACGAAATTGCGGCGACGGTTGAACGCATCAACGCGGCCAAAGCTGGT
ATCGAGGAGTTCATTATCTCGACGTACCCCACCAGGCAAGAGAGGTTTGAAGCACTTCGA
GCGGACTGCCCTGGTGTCATGACTCTCCACCTTTACAGACAAATTCGGTGCTATACCAAT
GGCGACATCGACTCTATCCGCTTCACCTGGCAACGGAAGGACTCCTTGAAGAAGCCCGTA
AAAGAGGAGCTTTTGCAACGCATCAGAGAGGAGCTGGAGCGATCTGGGCCAGACTATCAA
CTTCCCCTGGAGCAGCTTATCCAGAAGATCGCCAGCACCCCGGAGCCTTATCTCCGGGAG
CGAAGGGAAGTAAAGGTTCAACCGGTGGCAAACGTCATGGCCGCAGGGGTACTCAAAACC
GTTACCGCGCCAATGCCCCTGATCGTGCTCCAGGATAAGGATGTTCAGCTCAAATTGCTT
CGTAACTTTGACGCCTCAGAACAACGCAAAACTCGATCTGATAAAGCGGCATCGGAAATC
CTTGGCACCTTCGGTGGAGTCACTATCGAATCCTTCCCTGGATGAATCAGCGTGACCGCG
TAACAAGGCTTTTCCGCAGCCGGAAGGGCTTTGTCCCACACAAACAATTCCAATCGACAG

CAGCATGATATATAATTATGATAATTACCGTATTATCGTAATTATCGTAAACATTACAAA
TTAAAAGGAACGTTCTCATGGCAAGAAAATTGGTTGAGTTTGACGATGTAGCGGCTGCGG
CCCAAAAGCTCAAGGACGCCGGTAAACGCCCAACGGTCATCGCTATCAGAGACATCATTG
GCAAGGGGAGCTTTACCACCATTTCAACGTATCTCAAACAGTGGTCAGAGGAACACTCCC
TCGATGAAGAGCTGGTAGAGGTAGTCCTTCCAGAATCGGTTATGAGCGATGCTGAGCTCT
TCCTACAGAAGATCTATACGGTAGCCAAAGCAAGCGCCGATGAACAGCTTGAGCGCGAGC
GTGAACTGCTACGACAGAAAGAAATTGAGTACCAGGAAGATATGCAGCAAGCCGTGGACA
TGGCAAATGATGCCACCGAACGGGCTGAGCTACTGGAGGAACAACTTGAAGCTCTCACCA
ACAAAAAATCAGAGCTCGATGCGGCCCTTGGTAAGGCAGAAAACTCGCTATCCCTCAAGT
CTGCGGAGCTAGAACGCTCACTGGCTGACATTGATAAGCTGGAAAAGCGGATCGTTGAGT
TGGAGGGCAAGCTGGAGGCGAAAGCCGCAGATCTGACCCGAGCACAGGATCACCTGGAGC
AAGCTAAAAGCGAAAATCGCTCTTTGAGCCAAAAATTGGCAACTACAGAGGGTGAGTTGG
AGGAGCAAAAAGGCAAGAGTATAGAGCAGACAGAAAAGCTCCGGGCCGCTCAGGAACAAA
AAACATCGTTGAAAGAGCAGCTCGAAAATGTACAGGCCAAACTGGCCCAATCACAGGACT
CCCTTGCCACAGCCAAAGCTCATGGCGAATCCGCTGAACGAGAGTGCCAGCGCCTATCTG
GAGAGGTAGAAAAGCTGGACGCCAAATTATCCAGCGCCGAAGCAGAAGCTCGCGCTCTTG
TTCAAGACAAAGGCATGATGGCTGGTCAGTTGCAGGAAAAAGACCAGCAGGCCAAGAGCC
TCGAACAAAGACTCAATGATGCACTGACCAAAATTTCTGGGTTGGAGCAAGAGCTCGCTA
AAGCTGGTAAGGGAGGGAAAAAGAAAGAGGAAAACTGATGGCGCGAAAGGCTAAATATTC
AGAAGAGTGGCGACACAGAGCTGCGGCTCTTCAAACCAAGATTGAGGAGGCTATGACACT
AGCCACCTCATCTATTGGCGACTATCGCTGGTTACACCGTCTCCATAGTTGGGTTACGGA
GGTGGCTCAAGGTAAAGCCCCAGACTGGTGGACAGATCTGGATTGTGAAGTATCCCTCCC
CCGAGAAGAAAAGCGGATCAGTACGTTCCTCTCGACACAAAAGAAGCGCATCACTCTCCA
GATGTGTTTGTCGTAGGGGGCCTCATGAAACAACTTCCTCCTGACACACCAGAACAATCA
CTGATCACTCAGTACAAAGGGCCTCGCCTTGTCGTTAAGGCCTACGCTGGAACGGGTAAA
ACCACGACACTGGTGAAGTACGCCCACAACAACCTCGATTCACGTATCCTCTACTTGGCA
TACAACAGGGCTATCCGCGACGAGGCAAGAGAAAAGTTTCCTGCAAACGTAGACTGCAAA
ACGTCCCACCAGCTTGCCTACGCCACTATAGGAAGGGGCTACCAGCACAAACTCTCCGGC
AACCTAAGGCTCACCGATATTGCCCAAGCGGTGAATACCAAGAACTGGACGTTTGCCAAA
GATATTCTCGATACGCTCAACGCCTTTATGTGTAGTGCAGACATGCGGATTCTTTATACG
CATTTTGCTCGCGCCGATACGGGTAAAGTGCTTACGTCCAAACAGGAGAGATACCAAATC
CAGGTGGTCGAAGGTGCTGAGCTCATATGGAAACGGATGACAAACGTTCAAGATCCGTTC
CCGACCGTACACGATTGCTACCTCAAACAGTATCAGCTCGGGATGCCGAATCTGTCTCGC
CGGTACACCACCATTCTTTTTGATGAAGCACAAGACGCTAACCCCGTAACAAGTAGCATC
GTCCTACAGCAGAACTGCAAGGTAATCCTGGTTGGAGATCGCCACCAGCAGATCTATAGG
TTCAGAGGCGCAAACAACGCCCTTGATAGCAAAGAGCTCATGAACGCCGACCAACTCTAT
CTCACTCATAGCTTCCGCTTTGGCCCCAACGTTTCGCTGGTGGCAAACGCCCTTCTTGAA
CTCAAAGGTGAAACACGACCTGTTGTTGGCCGGGGACCAGCAGATCAGGTACTCATGTTT
TTACCAGGTGACGTGGGCCACCGCGCAATACTTCACCGAACCGTCATGGGGGTTATAGAG
ACGGCGCTCTCTGCGACCGAATCCGGAGCGCAGGTATTCTGGGTCGGTGGAATCGACGCT
TACCAGATCAATGAGCTCCAGGATTTGTACTGGTTTTCGATGGCAGAGCCAGACCGGGTA
AAGAATAAGAAACTGCTTGATGAGTATGAAGACTACTTCGAGTATCAAGAAGTAGCGAAG
GCGACCAAAGACCCTGAGATGATGAGGGCTGTCAAGATCATCAACAGCTACGATGAAATC
CCTGAACGACTCACCACTCTACGACGCAATACAGTCAAAGAAGAGTTTGGGGCTGACATT
ACGGTCTCAACAGCTCATCGGTGCAAAGGGTTGGAGTGGGACTTTGTTCAGCTCTATGAC
GACTTTCCGGATGTCCTGGACCCAGAGCTCGACCCAATGGCCCGTGACGATGAAATAAAC
CTGCTCTACGTTGCATCCACCAGAGCGATGCGAATCCTTGCGTTGAACAGCGCTGTCGAG
ATGGTTATCCGCTACATCACCCAAAAACGCATGGTCGAGAAGCAGATGAAGATGGCCGCA
GAAGCGACAGAAGTTGAAGAGGACACGACCAAATAGTTTGGTCAATTCTTTCACATGTGA
AAGTTGACAAATAAAACGCTCTAAGCGCCCTAAATGGGCGCTTACACCTGCCTAATTTCA
CGCCTCCCACCTCTACCATATCGAGCATGGGAAAATACGTGCGTGAGACTATGAAAAAAT
CACCTTTGAATTTACTGCTCCTTGCAGCGCTCACGCTGGGGGCATCACACCAGGCTTGGG
CTCAAGATGGCACAAGACCTGGTTTTTATGAGCGAAAGGAAGAGGGTTGGTTCTGGTACA
AGGAAGAGCCCAAAGAACCAGAGAAAAAACCCGAAAAGCCCAAACCAAAGCCTGTGGCAG

AAGCGAAGCCTACACAGCCTAAGCCTGCTGCTCCGCTTCCGAGCGGCCCAGAAATGTTCT
CAGCGGAATGGTTCCGGGAAAACTTACCCAAGTACAAAGACCTTGCTTGGAACAATCCTA
CCGTTGAAAACGTCAGGACGTTTCTCTACTTGCAACGATTTGCGATAGATCGCTCTGAAC
AATTTTCCGATGCTACAGAGCTGGCGGTCGTAGGTGATCCTTTCTTGGATGAAATTACTC
GACGTCCTGCTGCCACGTTTGCCTCACAACAAGTTGATCGTGACGCTGGTAACGCCAAAA
ACATGCTACTCAAAAGCGTAGCCGAACGCGTAGGGATATTCTTCTTCTACAAGTCCGACG
ATGACTACAGTGACTTGCAAGCACCGCTCATCAAGATGTTGGAACAAGGAGAAGGATTCT
CGATCATTCCTGTATCTATGGACGGCAAACCACTCCCCAGTGGGCTTTTCCCCCATTACA
AAACCGATGAAGGCCATGCCAAACAACTTGGTATCGTAACTTTCCCTGCTGTTTACCTCG
CATCTCCAGACGGTCAGTTCGCTCCTATAGGACAAGGGCCAATGTCTCTTCCTGAGCTGA
ATCACAGGATTTTGGTCGCAGCAAAACGCAATGGTTGGGTCACAGACGAAGAGTTTAACC
GTACACGTCCGGTACTCAACCTGGAAAACAACATAGCCGAACGCTTGGCCTCACCAGAGC
TGGGCTCTGACCTCAAACAGCTATCTCAAGCGAGCGGTGATAAAGACAACTTTGTGCCAC
CGGAACAACTCATGAAGTACATCCGGGACAAATTACAGGAGAACTAAGATGGTCACGCAC
AAGACATTAAAAAGGAGCCTGCTTGCCCTGAGTGTGGCGGCCAGTCTCGTCATGGCACCG
ACAGGGGCGATAGCCGCTAACGGCCTCCAATCACAGATGGACAAACTCTTCAATGAAATG
AGCAACACAACACCACCTGGGGTTTATGAAAACCAACGACGTGGCGTTTTAGCTGGTGGC
CGGTTCACTGCAAAGACACGAATCTTCGACGAGAACCTGGTGAGCTTTGCCCCTCCATCA
TGGAAAGCTGGTTGCGGTGGTGTAGATCTGTTCGGCGGTTCGCTTTCCTTCATTAACGCG
GATCAAATTGTCCAACTCCTTCGGGCTGTAGCAGCCAACGCCAAGGGCTATGCCTTCCAG
CTTGCGCTCGATAACGTTTTCCCGGACGGAGCGAAGTGGATAGAGAACTTCCAGAAGAAA
GTGCAAGCGCTCAACCAACATCTGGGCAACTCCTGCCAGCTCGCTCAAGGTTTCGTGAAC
GACCTAACCAGCGGCATGGACCTTAAACACAAAACTGATGCTTCTATAACCGCGACAACT
TCCGGCCTGTATGAAGACTTCTTCGGGTCCAAGCAGGAAACCAGCGGCAAGAGTCCTCTG
GAAGAACTGAAAGCCAACAAACCTGACGAATACAACAAGATGATTGGCAACATCGTCTGG
AAGCAACTCAAGAGCAACAACGCCAACACCTGGTTCCAGTACGGGGATAACACCCTTCTT
GAAGCGATCATGTCTTTAACCGGCACAGTCATCATTGGTGATCTGGTAAACGACCCGAAC
TCAACCGGCACTGGTGCGAAAACAACCCCTCTGACGACCCTACCAGGTAACAAGATCACC
TTGTCAGACCTGATTTCAGGCGGTTCTGTTGAGATCTATTCCTGTGATTCTGATACGACC
AACTGCCTGAGTGCTGGCTCCAGCAATAAAACTGTCGTGCTCAAAGGTATCAAGAACCAG
ATCACCGATATGCTGTTAGGAACAAGCTCTACACCTGGTGTGATCTACAAATACGCAACG
AACTCTGGAACCTTAACCGACCCAGAAAAGGCCTTTGTTTCTAACCTCCCCGGAGGGATT
GGCACCATTGTTCGTAACTTGTCTGTCCTTTCACAGGACGGCGCTAACCTGTTCGCAACA
GAGTCATCAGGAGCGATAGCCCTGACCATGATGTATAGCTTCTCGGAAGAGTTCTTCCGC
GCAGCTCGCATTGCGATGGCTAACAGCAAATCACCCTACAAGAAAGAGGCACTAGAGCTT
CTCGCGCAATCGCAACAGCAAATCCGTGCTGAATACACAATCCTGTCCTCTCAATACGGC
GATCTGGCAAGCCAAATTGAGAAATACAACAACCTACTGGACAACATCCGCAAGCAAAAA
TACATGCTGGCAACTTTGTCCAATCCTCCTAGCACGAACTAAGGAGCTATTGGAATGGGA
TCTTTTTCAATCCACTCTATCGGTGACTCTGCTTTTCTGGAGCAAATCCTGATTGCAGTA
TCAATGATCACCGGCACCGGGGATTTCGAGAAGATGGTCAGTATTGGCCTGCTTCTTGGG
GTCTTGATGATCTGTATTCAGTCCGTCTTTCAGGGCGCAAAGCAAATCAACCTCCAGCAA
GTGCTGGTAGGTTGGATTCTATATGCCTGTTTCTTCGGCCCAACCACAACTGTGACTATC
GAGGACGCTTATACAGGGCAGGTTCGAGTCGTCGCCAATGTCCCTATTGGGGTAGGCTTT
GCTGGAGGTGTCATATCCAACGTGGGATACACCATCACCAATTTGTTTGAAACTGGATAT
GGGGTAATCGTACCCAATGTCACGGAAAGCCACTTTTCCGAAACACTGAAACTGTTGAAT
GACGTCAGACGGCGAGCCTATGACACAGGAGTTTTTACTGCGCTTAACTCAGCAAATGGA
GGCGGCTATGTTGACGTGAGGCGTTCCTGGAACAATTACATTCGGGAATGCACGTTAACC
AAAGTCGATCTCAACCTAATGTCCCTTGATGAGTTGATGAACCGTTCAACTGACTCAGCT
TTGCGATTCAACTCACAGCTCTACGGAACTAGGTTGTATTTGTCTACAGCAAACCCTGAC
GGCGCTGACTACACATGTACTGACGGATGGGTGGCTATTAGCACTGCAACCGCCAACCTA
AGCAGCCCGGTTGTTGTTGATGCTCTTAACAGCCTACTGGGTATTGACACGTCAACTGGA
GACAACGCTCTAACGAAGCTGACCGATTCGCTTCAAGCGATGGGTGCCACAACTACGTCA
TCAATCGACTATCTGAAAGCCGCCGTTCTGGAGCCCCTCTATTATGAAGCCGCAGCAGGA
CGTTATCAGGACCTCCAGGATTATGGCTCTGCATTGATGGTCAACCAGGCTATTCAGCAA

CGGAACACACAGTGGGCCGCAGAGCAGTCGATGTTCATGACCGTCGTCCGACCAATGCTG
ACGTTCTTTGAAGGCTTTATTTATGCTATAACCCCGATCATTGCTTTTATTATCGTGATG
GGCAGCTTCGGCCTCCAGTTAGCCGGGAAATATGTACAAACCATCCTCTGGATTCAGCTA
TGGATGCCAGTCCTCTCAATTATAAACCTGTTTGTTCATACCGCCGCGTCAAATGAGATG
TCTAGCCTCAGTGCTGGTGGTCTCAACTCCATGTACGCTCTTTCCTCAACTGGAGATGTT
CTGCAACACTGGATTGCAACCGGCGGCATGTTGGCTGCGGCCACTCCGGTGATTTCCCTG
TTTATCGTCACAGGTAGCACCTACGCCTTCACCAGCTTGGCATCGAGAATAAGTGGTTCT
GACCACGTTGACGAAAAGATGCAAACGCCAGATCTACTCAAGCAAGGTCCGGTTATGCAA
AGTCAGCCAGCGTACAATCACAACCAGTTCAGTGGTGCGATTGCAAACGGCGCAGAAAGC
ATGATCAGTACCTTCTCGCTTGGCTCCACCTTGGCATCAGGCGTGAGCTCCGCACAGGCA
TTACAAAGTCAGAAATCGGAGGCTTTCCAAAGCACTCTTGGTCGAGGTTTTTCTGATGGA
GTAAGTCAGGATCAAGCCTATTCAAGACTCTCCAATGTCGGGCGCAACGTTTCGTCGCAA
AACACAGCTCAAAGCCAATTGATCAACCAGCAAGCCAAGAACTTCATGGATAAGTTCCAG
GTGGATGATAGCCACTCTGATGCTGTCAAAGGTGCTTTTGCCATGCAGGCTATGGGCACA
CTCGATGTTGACGAAGCTGCGTCCATGCTTATGCCTATGGTTGGCAAGGCCAGGGCAGCA
ATGAAGGCCGCTGCTGGTGTGAAATCAAACAGTACAGCCCTAGTTCCTGCTGGTGGTAAT
GGCGAATCAGGCGGCGGCAGTGATGTCCTGGACATCAAAGCGCAAGCGAAGGGAGCAACA
GAGTCATCAACTCAAGACTCTTCAAGCTGGTCAGCGAGTGATGTGTCCCAGTTCATGAAG
GGTGTGAGCTATTCGCAAACCGATAGCCAGGCGTTGACAAATCAATTAGCGCAGGGTTTC
AGCCGTTCTGGAAGCGAGTCATTCAAGCAAACCTGGGGCGATAGCTTATCCCAGAACCTA
TCCAAGTCCGCTTCTGAACTGGTGTCTGCATCGGACACCTTCACAACAATGAGTCAGCTC
CAAAACCAAATGGGCTCCATGACTAATACCGACTTTAAAACTCTCGGTGGTGCAGTAGCA
CAAACCCCTGCGGCCATGAACCAACTGAATGACTATTTCCGAAATGCCGCGCCGCAATCG
GTTAAAGACGAGGCGGCTTCACTACAGCAAAGATACCAAGCCTACGGAATGTCTCCTCAA
GTGGCCCAGGCAGCAGCGCGAATGACAGCAATGACCAACTCCAAAAATTACGAACAGGGT
AAGGAGCTTGGCGGGTATCAAGCCGCACTACAGGCGATCAATACCGCGTCCGGTCGCAAC
GGAGCATTTAGTGGTGATGCTTACGGAAATAACGGCATTGAAGGCCCGAATGTTCAAGGC
CTACCAGGTCAGGTTCAAGGGGCTGTAGGCAGTGGACCTAACATCCCAACCGGATTCCGG
GAGAATGTGGCTGGGATGGCTGGAACTAATCCGGCATCAGAAGCTGGGCAGTTACCAACG
AATAGCCCCCTTGTTCAAAATGAACATGCAGCCGGTACGTCAGCTCTTCATAACCAAGCA
CAGCAAACAGAGCGAAATGTATCTGCTCCTGAACTGAAAAAAGCCCAAGATAACCTTATG
AACTCGCTTCCGGAAATGTCTTGGAGCGCTTCGGCGTGGGGAGCGTGGGACAACTCCAGT
GATTGGATGGGCCGCAGAGCTGAACAAGCAGGTGGAGCTCTCATTGCTGGTGGTCAAGCT
GGCGCTGATGCGTTCTCAAGAGCGATGGATCAAATGAGAACGATGACACCTGAACAACGC
GACCAATTCATCGCGGCCACTCAACGCGGCGACCAGGCCGTGCAAGAGGAGTTTGGCTGG
GCCGGTGATGCGATGGTCGGTATGGCTAAACTTGGCCGCAACGTCATGGGAGCTGCTGCA
AGCGGCTATGATGCAGCGAAGGAGTGGTTAACTGGTAAATCTGATCTATCGGAAGCCGCT
AAAGGGATGAGCATTGAGGAACGCGGCGCGTTCTATGCAGCAGCGCTATCCTCTGCCGCA
GAAGCTGGTGGTGGAGCCGCGCAGCAGTTTATGAACCAGTACGGTGATGAGTTCAAAGAA
ACGATGCAGTCTATCGCTCAAAGCCGTTATGGGCTGACTGAATCCCAAGCCGCTGTTTAT
GCCGAATCGTTTGACACAAACGAAGGTCGCATGAACCAGGCTGTTCAGAATCTGAAAATG
GAGTATGCAGAACGTAACCCGGATGGCTCACCGATGATGCAAGGAGGTCAGCCTGTTCTT
TCTCAGCAAAACGAAGAGTTTACGGACAAGCTGGTAAACGTATTGCAGAACTCGACGGAA
GCTGGAGACCGTTCAGGTAGCTATTTGACTGCCGTCAGGGGGTACAACATAGCAAATCAA
AGGTTCTAAAAGACCAACCAAATAATAAGGGGGCCAGCAGGCCCCCTTATTCGTCTCCAG
CTCCAATATGGAAGCGCTGGTCAAAATCATAGGAATCTTGAAACCCACCAGTTTGTTTTT
TTCTGCGCCGGGTAGGTGGTGCCTCTTTATATGCTGACCGCGCAACGCGGATAAAGCACG
TTAGTCCAAACAGAGCCCCGACAACCAAAGCAACTTGCCACTGGAAGTAAGCGATCCCTC
CCCAAAAGATAACAGCAGCCCAAAGGCTTTTGTGAACCAGAAATAGGAAACGAGCACCCC
AAACGCCGAAGGGAGCCAGCGCCACACCAAGTCCCCAAGGTGTGTCAGCCATCATGGCTA
TTCCCAGAAGCACCATTCCCAGCAGAAGAATATTGACCACATGTTTCATATCAACCCCCT
TACTCTTTACCTCCAGTTTACCACGGCATCAAAAAATGCAACTGTGAAAGTTTGACTATT
TTTTGCTAAACAACCATTCCATTGGCAACTTCTTCGATTAGAATTTGCCCTCAAACTTTC
ACATGTGAAAGTTTGCCCTTACGGAACAAGGCGTTTATTGACAAAGCATTACATAGTTGA

ACATGTCAAACAAATCGCATACACTATGGAATAGGCCATTGCTAACGCTTTGGTCCCCAT
CTGCAACACCGCAGAGTTCTCTTTCTCAAATATCTCTTTCTGTAAACACCATCATCCTTG
TGTGGTCGGGATGCGCCTTTGCCTTCCCTGCCCTCCTCAGCTCCTGCTGTATTCGCAATT
TCAGCCCTCGCTGTATCCCTAAAATCACTTATACCTAAACGCTATTTAATATCACAAAAT
ACCGCACTCCCCGTGCGCTATTTCCGCCATTCTTTTGGCGCACTTTCCATTACTATCGCA
AAAATATCCGTATCCCATTGATTACTAAAAGTATTGTAACTTTGTTCCGTCGGTACAAAG
TTAAGTGTATGTTTTTAAAGGTTTTTTATTGTTTACAGCATCATTATTTGGGCTTATAAA
GGCGCAAAATGTGTCAGTTTGTGACAATAGAAGAACTCTAAAAGCGTTCATCGAATAACG
CACTTTTTGTATGGCTAACAACGCAGTTTAAAGGGTGGCAAACAATGGATAAATGCCAAT
TGATAGACATCCCAAGCGACCCAGAGAAGAAACGTGAGTGGATCAAGTACAAACTCAAGA
TCCAGGGGCTTTCTCTGGCCGCATTGGGCAGAAAACACAAAACATCTCGGCAGGTGGTGT
CTACGGCACTCTATAAGCCCAGTCCACGCTGGGAACATGAGATAGCTACAGCTTTGGGTG
TGAAGCCGTCTGAGATTTGGCCGGAGCGGTACGACGAAGAACACGAAATACCCCTCAGAC
ATAAGGAGGCAAGCTGATGAAGAACAAAGCCAAGGCGCTAGTTCTGTCTGCGGCTCTCCT
TTCATCAACAGCGAATGCTATTGACCTGAGCGGAACCATCTTCGACAAAGCAGCGAAAGC
ATATAACCTCGACCCTCTTCTAGTGTATTCGGTCGCATTGGCCGAATCTGCATCAGGGAG
AGGTAATGGCTCTATAAGTCCTTGGCCTTGGACGCTTCGCGTTCCTGGGCTTCCTTTCTA
TGCTAAGTCGGAAGATCAGGCAAAGGCTAAGCTCGCTGAGTTTCAGCAGCAGTACGGTCG
TGCCATTGATGTCGGGTTTATGCAAGTGAGCATCCGGTGGAATGGTCATAGAGTTTCTTC
TCCAGCAGATCTTCTCGACCCAGAGACCAACGTCATGGTTGGGGCAGAGGTGCTATCAGA
AGCCATTCAGTCATCTCCAAATGACTTGGAGCTTGGCGTTGGCCGCTATCACGCCTGGGA
AGACGAAATCCGAGCCAGAAACTATGGTAGCCGAGTCTTGGCTATCTATCGCAACCTTCG
TGATTTGTGAGGGGGGCGGAATGTTGGAACTGGATATTATTGGTGCGTGGGATGCAAGAG
CCGTCAACCTCGATCAAGAAGAAGCTGATAGAAACGTCTACGAGTTCGATCTGACATTGT
GGAACCTGCTATCCACTCTGGCAAAAGAACGTCCAGATGATGCGGCCTCACAATTTTCTT
TGGGCATGGACACCGTTCAAAAGCTGTCACTGGCAACACCTTCCCAATTGGAAGCTCTGG
CCTCTGGCGTGTTGATCTCTTTCAAACTCGAAACAGCAGAGCAGAACATCATCACGCGAC
TCTCTGGCGACTACGACCCTGTAGTTTTTATCAACCATAGTGTTGATGAATTTGATGCTG
CCTACTGGTTGCTATTTAACCGCGTCGCATCGAGAGACCCGGAGATGGCAAAGGAAGTTT
TCGGGGTTTCGAGAGAGCTTGCGGAGCTGGTGGCTAAGGCAACAGACAGCCAGTTGCGCC
ACATGTCTGGAACAACGGTTACGCATTTTACGCTTCGTTTTGCTCCGAGCATCATTGAAG
AAATTCTCGATGACAGCCGGGAAGAGTTAACACACCCGGTATTGAAAAAACTGCAACAGT
CTCTACAGGGACGTGGGAGGTGGAGATGAACATTGGCAACTCTGGTACATTGGGTCGCTG
GGTTACAGCTCGACACATGGCCCTTGCTGGGTACATCACAAAAATCATCATGATCGAGAC
TGGTCTGACCTACAAACAGGTCAGACGGCTTTACCAGGATCTGGAGAGGGACGGATATAC
TCTGGAACGAAAATCCAGAACTTTCCGGGGTGGTGCGACACTGATTCATAGTCACACATC
CAAGATACAGGCCTCTCTTCTAATGCAGCTCTACTTCAACATTGGTGGAGAAGCCGTGTT
GCGGTCTGTGAACATCAAAGCCTTGAACAAGGCATTTAGAATGTATCACGCAATCCGCAA
AGAAGTGCCCGGAATGAAAGGTGCTCGGTGGGCTCCGTTTGATATTACTGATGCCTGGTG
TCTTGCTTCGGAGCTGAGAAGTGGGGACGCAATGCTGGAGGTGTGCGACAACTGCAAGTG
TACGTACTTCACCTCTGTTAATCAAAGAACCTGCGTTGAATGTCCGTTCTGCAAAGAACA
AGGAAGGCATGGTGGTGGGGAGAAAGAGTGTGCTTGAGTAGACTATGACATTTCGGACCA
GAAGATGAGCGCCCAAACTTTGCGGCGCTCATTTTTTTACTTCTTCTGTGGGATACGGTA
GCGCTCCAGCTCGACAGCTCCCAACCCCTTGAAAGCATCCGGGCGACGTCCTTGCCCACT
CCACGAAACTCCGTCTTTTGAATATTTAGCATTATCAGGTTCTGATCTGCTCGTGAACAT
TTCGTTGAGCAAGCCGATGTCCACACCGCAGGATTCCATGTCGCTCATTATTCGCTCAGC
CTGAGCTCGCTTTTCTTTTTCTTCTTCTTCTCGCTTTTTGTACTCTTCCTCCAGTTCATT
GAGAACGCCCTTCATTCTGTCAATGATCTCCCGAACTTCATCTAACGGGAGTCCTCGCAA
CAGGGTGCGAATGCGGCTTTTACGCTTTAACTCTGCGATTATTAACTCTCTACGTTCAGC
CGCTGATAGCGTAGAGAACTCCTCGTGGTCTTTCATGTCATACGGAGCCCATCAGTTTTC
CTTTAAGTGAATCACGTTGAGAGTTCAGTTTTGCGTCAGTCTGCACTGGCGAATTTTGAT
GCTGTGCAGGTTGACTCTCTGAACTCTGTTGCTCAAAAGACCCAATGTTGCCAGAAAATC
GAAGAGAATAAAGACCCAATAGAGCAAGGGCGCGGAGCCGATCACTACGCGCCTTATGCT
GCATCTGGGACAGTTCACGATAGAGCTCCGGAAAGGCTTGCTCCGATATGTTCAGATTAG

ATATTTTTCCATCCCATTTGCTACCAGCCACAACGACCTCCTATCTATCAGCCGCAGAAC
CAGAAGCCCCTGGCATTGGATGCCACGGATTCGTTGGGCAGTACGATCCTGCTCTTAGGG
AAAAGCTCCTTGGCCGCATCTTGGTAAGCCTCGGCACCGCCGCCAGCCAGCAGAACTACG
TCAGCATCCATCCCGTCCTCACGCATTGACTTCCGCATAGGGATCAAGGCGTTTTGAGCG
ACTTTGGTTGAGGCTTTCTTGAAGTAGTCTTTGATCGATACCTTTTCACCGTAGAGGAAG
ATTTCGGCCTTACCGGCACGAATAGCTTTCTCGATCTTTTCGATGCCAGGGGCACCGCCG
TGGTCTTCCTGAATTAGCCGGTCCGTTTCCTGTAGCAACACCGACATCGCCTTGAGGCTG
GTGCCAGATGAGTGATAGCGGACCTCTCCCTCTTCAAGAGCTACCCAGTCTACAGAAAAG
AACCCAGGGTCAATTACAACGGTTTTTCCTCCCTGGATAATCTCCAGGAGGTCTTCATCT
TTGGTTGAACTTACAACATCCATGTAAGCACCGGCAGGTTGAGGTACAACCACGACAGAC
TTAACCGCTACCGATCGTTTTGGCGTGATCTGGTGTTCACCCTCAAGCCGAGATTTCAAC
GCCTCTCTGCGCTCTACGTCCATGTACTGACTAACAGGCAGGCCAGTCACCAGCACATCG
ATCTCCTTCTGCTCGGACATCAGGAGTGCAGCGTAGAAAAGAGCCTTGTATGGATTGGTC
GAAGGATAGTCGCCGTGAAGCTCACGCTCCCATCCTTGCAATCGGTCAGGCTCGACGCCT
GCAACCCATTTCTCTCCATCAATCACAACCTGAATGCAGGTCCCTGCACCGCCAGTTAAC
TGTTGTGGCATCAGTTCCAATGGACCTGCCCCCACCGGCATGACGACTGTGCGAGCTTCC
TCACCTTTATACCCCATTGCCATTTTCAGGTTGGAGTAACCAATATCCAAACCCAGAACA
AATTGACTCATGAAATCCTCCAAAGATTGCTTTTAGATTGCTTTTCGATTGCTTGCCGCG
TGTTGGATAGAGAATAAGGCTAAAAGCGGGGACAGATGGCCTGTGTAATTGCCCAAAAGG
GGCATATAGGTTGCTTTTCGGTGGCTTTTTGATCGTTGAGTAAGGGAACCTATGGGAACG
CTGCACGGATAGGCTCAGATAAACAGACCTTACCCTCGCATCGAGAACCGCTTGCCCTCC
AGCATCGAGAGACGGTGGTAAAGAGGCATTTGGAATCTTTGATGCCATATCCAATATATT
TGGAATCTTTAAATATAGATTCATATGTAAAGAGGCTGTGAAAGAATAAGAGCATCAAGA
TTCCAGATAGATAGAGGGAAATTTGACAAATTCCAAAGATGGGTTAGCCTAGTGACAGAA
CTAGATTCCAGTATTGGAATAATCAGCTTTAAATTCCAGATAGATAGTTATGTGGATAGG
AATTGGATAGGAATTGGGAGGGTATTGAGGTGAGTCTACCAACAGAGCGATGGCTAGATC
TGCTAGATCCGAAGCTCGAAGAAGAACGATCCGAGATAACAGAGGATCTGCTAGAGGCAG
AGGGACGAGAGTTTGTTGCAGAGGTACGGAGCAAACTGGATCACGCCTTAGCGGTTCTTG
CTGTCGAGGCGCAGCAGGAAGCGGACATGTACTGGAACGCGCACAAATCAGCGCGTGAAG
AAGCGTCAGAGGACGAACAAGGGCGTGTCGGTACACGGGTTCGCATTCTAGGCGTATCAC
TCGTTGCAGAGTGGTATCGCAACAGATTTGTCGAACAAGTTCCCGGACAAAAGAAAAGGG
TTCTATCGACACATATCAAGAAGGGTCGCGGTCATGCCTACAGCATGTCGCACTTCAAGA
AAGAGCCTGTCTGGGCACAAGAGTTGATCCAGCAAGTTGAAACCAGGTATGCCGTGTTAA
GACAACGCGCCACTGCCTTAGCAAAAATTCGCCGGGCGCTAAACGAGTACGAGCGCCAGC
TAAACAAGACACATAGCGACGAGGTGTGACAACATGACAGCATCTGTAGCGGCCACTGAA
TTGGCGAAACTGGGGAAATGTGAAGCGATGATCAAGAAAGTCGCCAGCCATCCTCGCCCT
GCCCTGTCAAAGCGCCCACAATCGCCACAGGGGACAGATAGCACCCTTCGGGGTGAGTTC
GCTCATTTCCGATATGAAGCGGCAGCCCTGCGTTTTATGAGCGGCACTGCGGGGGCTAAG
CGTCGCATCTACCAGCTAGTGTTTTCAGCGACGGTAGCAGCGGGAGCATTGACAATGCTG
GCGGC
